# Hand, Foot, and Mouth Disease in China: Modeling Epidemic Dynamics of Enterovirus Serotypes and Implications for Vaccination

**DOI:** 10.1371/journal.pmed.1001958

**Published:** 2016-02-16

**Authors:** Saki Takahashi, Qiaohong Liao, Thomas P. Van Boeckel, Weijia Xing, Junling Sun, Victor Y. Hsiao, C. Jessica E. Metcalf, Zhaorui Chang, Fengfeng Liu, Jing Zhang, Joseph T. Wu, Benjamin J. Cowling, Gabriel M. Leung, Jeremy J. Farrar, H. Rogier van Doorn, Bryan T. Grenfell, Hongjie Yu

**Affiliations:** 1 Department of Ecology and Evolutionary Biology, Princeton University, Princeton, New Jersey, United States of America; 2 Division of Infectious Disease, Key Laboratory of Surveillance and Early Warning on Infectious Disease, Chinese Center for Disease Control and Prevention, Beijing, China; 3 Woodrow Wilson School of Public and International Affairs, Princeton University, Princeton, New Jersey, United States of America; 4 WHO Collaborating Centre for Infectious Disease Epidemiology and Control, School of Public Health, Li Ka Shing Faculty of Medicine, The University of Hong Kong, Hong Kong Special Administrative Region, China; 5 Oxford University Clinical Research Unit, Hospital for Tropical Diseases, Ho Chi Minh City, Viet Nam; 6 Centre for Tropical Medicine, Nuffield Department of Medicine, University of Oxford, Oxford, United Kingdom; 7 Fogarty International Center, National Institutes of Health, Bethesda, Maryland, United States of America; George Washington University, UNITED STATES

## Abstract

**Background:**

Hand, foot, and mouth disease (HFMD) is a common childhood illness caused by serotypes of the *Enterovirus A* species in the genus *Enterovirus* of the Picornaviridae family. The disease has had a substantial burden throughout East and Southeast Asia over the past 15 y. China reported 9 million cases of HFMD between 2008 and 2013, with the two serotypes Enterovirus A71 (EV-A71) and Coxsackievirus A16 (CV-A16) being responsible for the majority of these cases. Three recent phase 3 clinical trials showed that inactivated monovalent EV-A71 vaccines manufactured in China were highly efficacious against HFMD associated with EV-A71, but offered no protection against HFMD caused by CV-A16. To better inform vaccination policy, we used mathematical models to evaluate the effect of prospective vaccination against EV-A71-associated HFMD and the potential risk of serotype replacement by CV-A16. We also extended the model to address the co-circulation, and implications for vaccination, of additional non-EV-A71, non-CV-A16 serotypes of enterovirus.

**Methods and Findings:**

Weekly reports of HFMD incidence from 31 provinces in Mainland China from 1 January 2009 to 31 December 2013 were used to fit multi-serotype time series susceptible–infected–recovered (TSIR) epidemic models. We obtained good model fit for the two-serotype TSIR with cross-protection, capturing the seasonality and geographic heterogeneity of province-level transmission, with strong correlation between the observed and simulated epidemic series. The national estimate of the basic reproduction number, *R*_0_, weighted by provincial population size, was 26.63 for EV-A71 (interquartile range [IQR]: 23.14, 30.40) and 27.13 for CV-A16 (IQR: 23.15, 31.34), with considerable variation between provinces (however, predictions about the overall impact of vaccination were robust to this variation). EV-A71 incidence was projected to decrease monotonically with higher coverage rates of EV-A71 vaccination. Across provinces, CV-A16 incidence in the post-EV-A71-vaccination period remained either comparable to or only slightly increased from levels prior to vaccination. The duration and strength of cross-protection following infection with EV-A71 or CV-A16 was estimated to be 9.95 wk (95% confidence interval [CI]: 3.31, 23.40) in 68% of the population (95% CI: 37%, 96%). Our predictions are limited by the necessarily short and under-sampled time series and the possible circulation of unidentified serotypes, but, nonetheless, sensitivity analyses indicate that our results are robust in predicting that the vaccine should drastically reduce incidence of EV-A71 without a substantial competitive release of CV-A16.

**Conclusions:**

The ability of our models to capture the observed epidemic cycles suggests that herd immunity is driving the epidemic dynamics caused by the multiple serotypes of enterovirus. Our results predict that the EV-A71 and CV-A16 serotypes provide a temporary immunizing effect against each other. Achieving high coverage rates of EV-A71 vaccination would be necessary to eliminate the ongoing transmission of EV-A71, but serotype replacement by CV-A16 following EV-A71 vaccination is likely to be transient and minor compared to the corresponding reduction in the burden of EV-A71-associated HFMD. Therefore, a mass EV-A71 vaccination program of infants and young children should provide significant benefits in terms of a reduction in overall HFMD burden.

## Abstract 2

Using time series susceptible–infected–recovered (TSIR) epidemic models, Saki Takahashi and colleagues investigate hand, foot, and mouth disease dynamics and the projected effect of vaccination in China.

## Editors' Summary

**Background:**

Hand, foot, and mouth disease (HFMD)—a common ailment that mainly affects young children—is caused by a group of enteroviruses (*Enterovirus A* species), which are close relatives of polioviruses (*Enterovirus C* species). Enteroviruses are divided into various viral serotypes (variants defined by molecules on their surface that are recognized by the immune system), and the most common serotypes that cause HFMD are Enterovirus A71 (EV-A71) and Coxsackievirus A16 (CV-A16). Enteroviruses spread from person to person through contact with the mucus or saliva produced when an infected individual coughs or sneezes, with the feces or the fluid from vesicles of an infected individual, and through contact with contaminated surfaces. Frequent handwashing and good hygiene practices can reduce the spread of HFMD. Symptoms of HFMD include fever, sore throat, a non-itchy red rash with small blisters on the hands and feet, and painful mouth ulcers. HFMD is usually a self-limiting illness, and most children recover within 7–10 days. A small proportion of patients infected with EV-A71 develop severe complications such as meningitis (infection of the membranes around the brain and spinal cord) or encephalitis (infection of the brain). Currently, there is no specific treatment for HFMD, and vaccines are not yet available for routine use.

**Why Was This Study Done?:**

HFMD is increasingly common in East and Southeast Asia. China, for example, reported 9 million cases of HFMD between 2008 and 2013. Vaccination is a specific and effective way to reduce the burden of HFMD in China. In three clinical trials, inactivated monovalent EV-A71 vaccines made in China were highly efficacious against EV-A71-associated HFMD but provided no protection against CV-A16-associated HFMD (an inactivated monovalent vaccine contains a single virus strain that is unable to replicate; exposure to the vaccine “primes” the immune system to respond quickly when challenged with live virus, thereby preventing infection with that virus). So, before these vaccines can be used for routine vaccination of infants, it is important to know whether vaccination with EV-A71 will alter the burden of HFMD caused by other enterovirus serotypes. In particular, it is important to know whether infection with EV-A71 provides cross-protection against CV-A16 and whether infections with CV-A16 or other serotypes might increase following vaccination against EV-A71 because of increased circulation of these viruses in the population (serotype replacement). Here, the researchers use mathematical models to assess the effect of vaccination against EV-A71-associated HFMD in China and the potential risk of serotype replacement by CV-A16.

**What Did the Researchers Do and Find?:**

The researchers used weekly data on HFMD incidence collected in 31 Chinese provinces between 2009 and 2013 to develop a two-serotype time series susceptible–infected–recovered epidemic model (a model in which individuals are born, become susceptible to a disease, become infected and infectious with the disease, and recover). The model accurately simulated the epidemic cycles of EV-A71- and CV-A16-associated HFMD for the 31 provinces and the seasonal transmission patterns in both northern and southern Chinese provinces. It provided an estimate of cross-protection following infection with EV-A71 or CV-A16 of ten weeks in 68% of the population (an average duration of cross-protection of 6.77 weeks). The estimated basic reproduction number (the average number of additional cases one case of an infectious disease generates in an otherwise uninfected population) across China for both serotypes was 25, which means that vaccination coverage levels of above 96% are required to achieve population-level immunity. Finally, the model predicted a decrease in EV-A71-associated HFMD incidence with higher rates of EV-A71 vaccination and suggested that CV-A16 incidence following EV-A71 vaccination would be comparable to or only slightly higher than its incidence before vaccination.

**What Do These Findings Mean?:**

These findings suggest that herd immunity (indirect protection from infectious disease that occurs when most of a population has become immune to an infection, thereby providing some protection for the rare individuals who are not immune) is driving the dynamics of the HFMD epidemic caused by multiple enterovirus serotypes in China. Moreover, they suggest that the infection with EV-A71 or CV-A16 serotype can provide temporary immunity against each the other serotype and that serotype replacement by CV-A16 following EV-A71 vaccination is likely to be transient and minor compared to the reduction in the burden of EV-A71-associated HFMD produced by vaccination. The accuracy of these findings depends on the assumptions included in the model and the quality and quantity of data used to run the models. However, the researchers suggest that a mass EV-A71 vaccination campaign targeted at infants and young children should greatly reduce the burden of HFMD in China, provided a high vaccination uptake level is achieved.

**Additional Information:**

This list of resources contains links that can be accessed when viewing the PDF on a device or via the online version of the article at http://dx.doi.org/10.1371/journal.pmed.1001958.

- The US Centers for Disease Control and Prevention provide information on hand, foot, and mouth disease (in English and Spanish), including a podcast on the condition
- The UK National Health Service Choices website provides detailed information on hand, foot, and mouth disease
- Further information about hand, foot, and mouth disease is provided by the World Health Organization (including up-to-date HFMD surveillance reports from China), the Nemours Foundation (in English and Spanish), and MedlinePlus (in English and Spanish)
- The Government of the Hong Kong Special Administrative Region Department of Health Centre for Health Protection provides information on hand, foot, and mouth disease

## Introduction

Hand, foot, and mouth disease (HFMD) is a common childhood illness caused by serotypes of the *Enterovirus A* species in the genus *Enterovirus* of the Picornaviridae family [1,2]. HFMD predominantly affects children younger than 5 y of age [3], and most patients exhibit a self-limiting illness that typically includes fever, skin eruptions on the hands and feet, and vesicles in the mouth. However, a small proportion of infections lead to the development of neurological and systemic complications that can be fatal, particularly in cases associated with the Enterovirus A71 (EV-A71) serotype [4]. Since 1997, EV-A71-associated HFMD epidemics have been increasingly reported across the Asia-Pacific region, chronologically in Malaysia [5], Taiwan of China [6], Japan [7], Singapore [8], Viet Nam [9], Mainland China [10,11], Hong Kong Special Administrative Region of China [12], South Korea [13], and Cambodia [14]. China reported 9 million cases of HFMD between 2008 and 2013, with the two serotypes EV-A71 and Coxsackievirus A16 (CV-A16) being responsible for 73% of these cases [15]. As such, HFMD is a growing public health concern that poses a considerable disease burden and economic impact in affected areas [16,17]. Recent concerns with disease due to outbreaks of Enterovirus D68 in the US and other countries have only increased the urgency to understand the local viral community dynamics of this group of infections [18].

Transmission of enteroviruses occurs by direct contact with the mucus, saliva, or feces of an infected individual, or through indirect contact via contaminated surfaces [19]. HFMD infection characteristically causes acute illness with a duration of approximately 1 wk [20–22]. There are no established antiviral treatments for HFMD, but three recent phase 3 clinical trials of inactivated monovalent EV-A71 vaccines manufactured in China were found to have high efficacy (90.0%–97.4%) against EV-A71-associated HFMD in infants and young children [23], and two of these vaccines were licensed in China in December 2015 [24]. However, these vaccines did not offer protection against HFMD caused by the CV-A16 serotype [25].

There is empirical evidence that infection with one serotype of a multi-serotype viral pathogen such as influenza or dengue virus can lead to transient immunity against other serotypes of the same infection [26–28]. There is also some historical and more recent evidence of interference between polioviruses (*Enterovirus C* species) and other enteroviruses [29–31]. For HFMD specifically, co-infection of a single individual with both serotypes is rarer than expected by chance [32,33], suggesting the existence of at least short-term cross-protection, and neutralization assays have shown partial cross-reactivity between the EV-A71 and CV-A16 serotypes [34,35], indicating a potential competitive interaction. However, the effects of multi-serotype interactions remain poorly understood to date. Mathematical models can be used to bridge this gap, allowing for the estimation of key parameters governing cross-protection and the evaluation of the epidemiological impact of vaccination. Previous modeling studies have used variations of the continuous-time susceptible–infected–recovered (SIR) model to study the seasonal patterns of HFMD and its basic and effective reproductive numbers [36–39]. These analyses have focused on estimating epidemiological parameters of either EV-A71-associated HFMD alone or all-cause HFMD, and have not accounted for the potential cross-protective effects of infection with a particular serotype on the dynamics of other serotypes.

To our knowledge, two important aspects of HFMD dynamics are yet to be addressed: first, the evaluation of the duration and strength of cross-protection between different serotypes and, second, potential serotype replacement by CV-A16 or other serotypes (e.g., CV-A6 or CV-A10) following vaccination against EV-A71, due to decreased EV-A71 incidence and an accompanying reduction in cross-immunity against CV-A16 and other serotypes, as well as potential increased circulation of these viruses in the population. Such serotype replacement has been observed, for example, for pneumococcal disease, with increases in the prevalence of non-vaccine serotypes following use of the pneumococcal heptavalent conjugate vaccine [40–42]. Vaccination against EV-A71 may potentially have important effects on the burden of disease caused by CV-A16 and other serotypes, and robust predictions of these effects are necessary before introduction of an EV-A71-containing vaccine into a population.

In this analysis, we adopted a mathematical modeling approach to study the multi-serotype transmission dynamics of HFMD in the Asia-Pacific region. We linked models closely to surveillance time series, using a multi-serotype time series epidemic model to examine the dynamics of HFMD caused by EV-A71 and CV-A16 in the 31 provinces of Mainland China between 2009 and 2013. We evaluated the potential existence of cross-protection between the two serotypes and the risk of serotype replacement by CV-A16 following prospective EV-A71 vaccination. We also extended the model to assess the co-circulation of additional non-EV-A71, non-CV-A16 enteroviral serotypes.

## Methods

### Data

We used three sources of data in this analysis. First, weekly reports of HFMD incidence between 1 January 2009 and 31 December 2013 (a total of 262 wk) were obtained from a national surveillance system maintained by the Chinese Center for Disease Control and Prevention (China CDC) in Beijing, China. These reports were available at two spatial scales: the seven administrative regions and 31 provinces of China.

**Fig 1:**
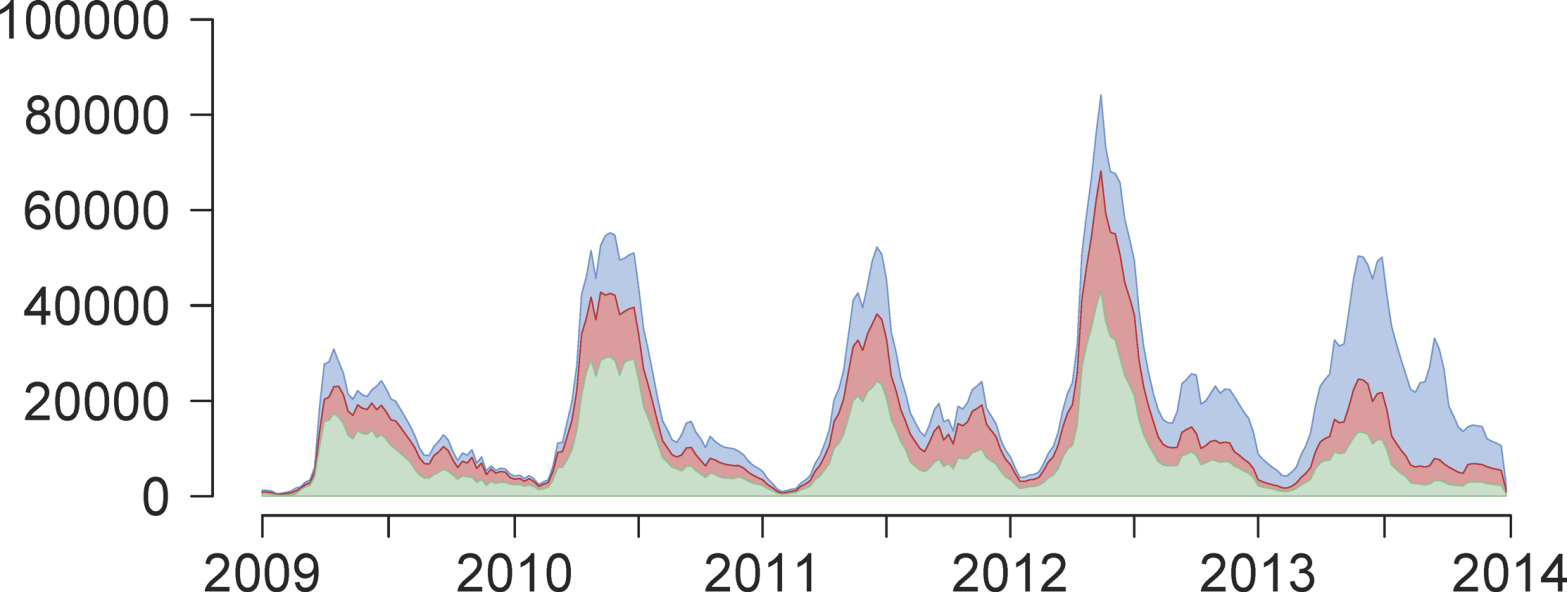
Weekly reported time series of HFMD cases between 1 January 2009 and 31 December 2013. Weekly reported time series of number of HFMD cases (*y*-axis) across time (years 2009–2013, *x*-axis), aggregated across all 31 provinces of Mainland China, not adjusted for estimated reporting rate and stratified by serotype: EV-A71 (green), CV-A16 (red), or other non-EV-A71 and non-CV-A16 serotypes of enterovirus (blue).

Second, we obtained time series of weekly laboratory-confirmed HFMD for a sub-sample of cases from each province, with virological test results classified as EV-A71, CV-A16, other non-EV-A71 and non-CV-A16 serotypes of enterovirus, or negative [15]. This information was aggregated to the regional scale on a monthly basis to reduce potential biases from the sampling scheme (S1 Fig). The proportions of each serotype were estimated, with all positive and negative tests included in the denominator. If there were no virological test data available for a given month, the proportions from the virological tests of the previous month were substituted. We applied these proportions to the reported case counts from the surveillance registry to estimate serotype-specific weekly incidence by province (Fig 1; S2 Dataset). Since infection with EV-A71 or CV-A16 accounted for the majority of total laboratory-confirmed HFMD cases between 2008 and 2013 (73%), we limited the scope of the main analysis to infection with either of these two serotypes. We used a time step of 1 wk: the incubation period of HFMD is 7 to 14 d and viral excretion persists for about 2 wk after symptom onset, so the generation time would be contained in this time frame. It has previously been shown that the estimation of seasonality is robust to the length of the chosen time step [43].

Third, we obtained yearly birth rates and population sizes between 2009 and 2013 by region and by province from the National Bureau of Statistics of China (S1 Dataset) [44]. Although reports of HFMD cases were available from 2008, the time frame of this analysis was limited to the period between 1 January 2009 and 31 December 2013 because of the sparsity of laboratory-confirmed cases during the first year of surveillance.

### Host and Transmission Dynamics

The time series susceptible–infected–recovered (TSIR) model is a discrete-time version of the continuous-time SIR model in which individuals are born and enter the susceptible class of individuals, become infected and infectious with a disease, and recover and are removed thereafter [45]. Here, we used a multi-serotype extension of the TSIR model that allows for a cross-protective effect between serotypes [46]. The susceptible compartment of the TSIR model is defined by:

$$S_{t+1,i}=S_{t,i}+B_{t}-I_{t,i}^{\left(r\right)}\;/\;\rho_{i}-{\text{CP}}_{t,i}$$ (1)

where at each time step *t*, *S*_*t*,*i*_ is the number of individuals that are susceptible to serotype *i*, *B*_*t*_ is the number of births (known from demographic data), $I_{t,i}^{\left(r\right)}$ is the reported number of individuals infected with serotype *i*, ρ_*i*_ is the reporting rate of serotype *i* (assumed to be constant over the entire time period), and CP_*t*,*i*_ represents the effect of cross-protection following infection with serotype *i* against all other serotypes. We assumed that there is no maternal immunity period for HFMD. Assuming that all individuals are born susceptible to HFMD and eventually become infected, we rearranged Eq 1 as follows:

St,i=S¯i⋅N¯+D0,i+∑k=0t−1Bk−∑k=0t−1Ik,i(r)ρi−∑k=0t−1CPk,i (2)

where ${\bar{S}}_{i}$ is the mean proportion of individuals that are susceptible to serotype *i*, $\bar{N}$ is the mean number of individuals in the population over the time period of interest, and *D*_0,*i*_ is the unknown deviation around the mean number of individuals in the population that are susceptible to serotype *i* at the beginning of the time series. We reconstructed the time series of susceptible individuals by first extracting *D*_*t*,*i*_, the residuals from the following linear regression:

∑k=0t−1Bk=∑k=0t−1Ik,i(r)ρi+∑k=0t−1CPk,i+Dt,i−D0,i (3)

The ρ_*i*_ values were estimated as the slope of this regression (using the ρ_*j* ≠ *i*_ parameters as iteratively estimated offset terms as per [46], since the *I*_*t*,*i*_ and CP_*t*,*i*_ terms both depend on ρ_*i*_) and are quite low because of the mild nature of the infection [47], though the reporting rates of EV-A71 are generally higher than those of CV-A16 due to the severity of symptoms. The *D*_*t*,*i*_ values were then added to the mean number of susceptible individuals in the population (${\bar{S}}_{i}\cdot \bar{N}$, with ${\bar{S}}_{i}$ estimated using marginal profile likelihoods and its 95% confidence interval [CI] derived from the χ^2^ distribution with 1 degree of freedom) to yield the complete time series of susceptible individuals:

$$S_{t,i}={\bar{S}}_{i}\cdot \bar{N}+D_{t,i}$$ (4)

In the multi-serotype TSIR model framework, HFMD transmission is characterized by the following stochastic frequency-dependent dynamics from the Poisson distribution:

It,i=It,i(r)ρi (5)

$$I_{t+1,i}∼Pois\left(\lambda_{t+1,i}\right)$$ (6)

$$E\left[I_{t+1,i}\right]=\lambda_{t+1,i}=\beta_{s,i}\cdot I_{t,i}{}^{\alpha }\cdot S_{t,i}/N_{t}$$ (7)

where *I*_*t*,*i*_ is the number of infected individuals adjusted for reporting rate, λ_*t*+1,*i*_ is the expected number of individuals infected with serotype *i* at time *t* + 1, β_*s*,*i*_ is a seasonally varying transmission rate, α is a correction parameter accounting for nonseasonal heterogeneities in mixing [48,49] as well as for time discretization [50], and *N*_*t*_ is the total population size at time *t*. We linearized Eq 7 and estimated β_*s*,*i*_ with the following regression model:

$$\text{log}\left(\lambda_{t+1,i}\right)=\text{log}\left(\beta_{s,i}\right)+\alpha \cdot \text{log}\left(I_{t,i}\right)+\text{log}\left(S_{t,i}\right)-\text{log}\left(N_{t}\right)$$ (8)

Extensive simulation studies indicate that the log-linear model is a better regression model than the log-transformed linear regression model (S31 and S32 Figs). Additionally, the log-linear model is a tractable approach to approximating the negative binomial distribution, which is formally a more satisfying representation of the underlying Reed–Frost epidemic model [51]. Because weekly case counts are relatively high at the province level (and define the over-dispersion of the negative binomial distribution), we can closely reflect these dynamics using the log-linear model and avoid the need to explicitly account for over-dispersion.

Predictions for *S*_*t*+1,*i*_ and *I*_*t*+1,*i*_ were generated using Eqs 1 and 6, respectively, performing separate estimation on each of the 31 provinces of China and setting *I*_*t*+1,*i*_ = λ_*t*+1,*i*_ in the deterministic simulations. We used α = 0.95 throughout this analysis for consistency in comparing seasonal patterns across provinces; a sensitivity analysis varying α values between 0.91 and 0.99 is provided in S22–S30 Figs. Co-infection was omitted from the two-serotype model due to its relatively rare occurrence in practice (S4 Table). Model fit was assessed by comparing observed incidence against simulated incidence, and calculating the coefficient of determination (*R* ^2^) and absolute yearly prediction error (PE):

$${\text{PE}}_{\text{year}\;m}=\left|\left(\frac{\sum \text{predicted cases in year}\;m-\sum \text{observed cases in year}\;m}{\sum \text{observed cases in year}\;m}\right)\right|$$ (9)

### Cross-Protection, Epidemiological Parameters, and Vaccine Simulation

The multi-serotype TSIR model allows for a cross-protective effect between serotypes. Supposing that infection with serotype *i* is fully immunizing against that serotype (e.g., exhibits SIR epidemic dynamics rather than susceptible–infected–recovered–susceptible [SIRS] epidemic dynamics) and that a proportion of those infected individuals also gain transient immunity against all serotypes *j* ≠ *i*, a fixed duration of cross-protection can be modeled as in [46]:

$${\text{CP}}_{t,i}=\delta \cdot \sum_{j\neq i}\left(I_{t,j}-I_{t-k,j}\right)$$ (10)

where δ is the proportion of those infected with one serotype (adjusted for reporting rate) who are cross-protected against infection from all other serotypes for a fixed duration of *k* time steps, and CP_*t*,*i*_ represents the effect of cross-protection following infection with serotype *i*. The product *k* ⋅ δ represents the average duration of cross-protection, incorporating individuals who do and do not experience cross-protection [46]. After the specified length of cross-protection, we assumed that individuals who had experienced cross-protection subsequently become re-susceptible to all other serotypes. 95% CIs for *k* and δ were derived from the profile likelihood using the χ^2^ distribution with 1 degree of freedom.

We estimated two time-varying epidemiological parameters for each serotype: *R*_0_ (basic reproductive number) and *R*_E_ (effective reproductive number), obtained by rearranging Eq 7 and defined as:

$$R_{0}=\beta_{s}$$ (11)

$$R_{\text{E}}=I_{t+1}/I_{t}=\beta_{s}\cdot I_{t}{}^{\alpha -1}\cdot S_{t}/N_{t}$$ (12)

*R*_0_ is the average number of secondary infectious persons resulting from one infectious person following his/her introduction into a completely susceptible population. *R*_E_ is the average number of secondary infectious persons resulting from one infectious person into a population that is partially immune; it is above 1 when incidence is increasing and below 1 when incidence is decreasing.

There is an inherent trade-off between having sufficient data to estimate the cross-protection parameters and having sufficient data to estimate the epidemiological parameters for the multi-serotype model. We chose to set aside the first year of the full laboratory-confirmed time series (2009) to back-fit the cross-protection parameters *k* and δ, and the remaining 4 y of data (2010–2013) were used for fitting the epidemiological parameters ρ_*i*_ and β_*s*,*i*_. Thus, our estimate of *k* was necessarily restricted to be less than 1 y long, and we parsimoniously assumed the values of *k* and δ to be the same for infection with any serotype. Log-likelihood surfaces of the cross-protection parameters were generated by the following modified log-linear regression model, including the reporting rate as an additional offset term:

$$\log E\left(I_{t,i}^{\left(r\right)}\right)=\log\left(\rho_{i}\right)+\;\text{log}\left(\beta_{s,i}\right)+\alpha \cdot \text{log}\left(I_{t,i}\right)+\text{log}\left(S_{t,i}\right)-\text{log}\left(N_{t}\right)$$ (13)

While our primary model is the two-serotype TSIR model for EV-A71 and CV-A16, we also explored a three-serotype model including the potential epidemic series encompassing non-EV-A71, non-CV-A16 serotypes of enterovirus (S6–S8 Tables).

To simulate the effects of introducing monovalent EV-A71 vaccination in the population, we assumed (based on findings from recent clinical trials) that vaccine-induced immunity against EV-A71 is narrow and has no immunological effect against CV-A16 infection [52]. In line with the high efficacy levels found in clinical trials, we also assumed a perfect vaccine efficacy of 100%. To capture the temporal dynamics in the simplest way, seasonality in transmission was omitted from vaccine simulations [53]. To model the effects of vaccination (assumed to be administered near birth), the size of each weekly birth cohort was reduced by the vaccine coverage. For example, if the achieved vaccine coverage was 90%, then 10% of the children born in a week would be susceptible to HFMD. We also assessed the impact of a broad monovalent EV-A71 vaccine that confers the same duration and strength of cross-protection against CV-A16 as natural infection with EV-A71, as well as the impact of a narrow bivalent EV-A71, CV-A16 vaccine that is equally protective against the two serotypes. All analysis was conducted using R version 3.0.2 (http://cran.r-project.org).

### Ethical Approval

In May 2008, HFMD was added to the list of notifiable diseases in China. According to China’s law on the prevention and treatment of infectious diseases, personal identifiers should be collected for individual cases with diagnosis of a notifiable disease, for the purposes of public health surveillance and response. The National Health and Family Planning Commission of China decided that the collection of individual data for all notifiable diseases, including HFMD, according to the national surveillance protocol was part of an ongoing public health response and was thus exempt from institutional review board assessment.

The China CDC has strict regulations on how to protect patients’ privacy. The National Center for Public Health Surveillance and Information Services at the China CDC is responsible for the management of all disease surveillance data, and it anonymized the individual HFMD data by deleting the personal identifiers (such as patient name, parent name, home address, and telephone number) before the China CDC co-authors of this article, in the Division of Infectious Disease, were given access to the surveillance data for the purposes of research. The co-authors of this article did not participate in de-identifying the data and do not have the personal identifiers of the HFMD cases. Data were also aggregated by week, enterovirus serotype, and province by the China CDC co-authors before analysis.

## Results

### Model Fit

The two-serotype TSIR model provides a good fit to patterns of HFMD incidence in the 31 Chinese provinces. The median proportion of individuals susceptible ($\bar{S}$) to EV-A71 was 0.067 (interquartile range [IQR]: 0.051, 0.153), and the median $\bar{S}$ for CV-A16 was 0.061 (IQR: 0.051, 0.070); the mean reporting rate of both serotypes (ρ) was approximately 3% (S1 Table). Our national estimate of *R*_0_, weighted by provincial population size, was 26.63 for EV-A71 (IQR: 23.14, 30.40) and 27.13 for CV-A16 (IQR: 23.15, 31.34), with considerable variation between provinces (Table 1). In comparison with separate one-serotype models (e.g., with no cross-protection) (S5 Table), the two-serotype model fit as well or better in terms of the mean absolute yearly PE in 20 of the 31 provinces for EV-A71, and 23 of the 31 provinces for CV-A16 (S7 Table).

**Table 1:** Median and interquartile range of *R*_0_ by serotype and by province.

Province	*R*_0_ of EV-A71 (IQR)	*R*_0_ of CV-A16 (IQR)
Beijing	16.42 (13.68, 18.40)	34.26 (28.54, 37.27)
Tianjin	7.64 (6.60, 9.82)	20.28 (17.70, 26.95)
Hebei	23.67 (20.65, 27.50)	27.31 (23.08, 31.48)
Shanxi	34.96 (28.80, 42.05)	34.91 (28.53, 42.27)
Inner Mongolia	1.61 (1.32, 2.09)	25.13 (19.56, 30.37)
Liaoning	33.76 (27.42, 45.24)	29.67 (23.70, 39.00)
Jilin	3.39 (2.95, 4.36)	38.33 (32.64, 47.55)
Heilongjiang	16.79 (14.68, 22.00)	55.87 (46.21, 70.06)
Shanghai	19.96 (16.34, 22.44)	26.33 (21.92, 29.99)
Jiangsu	26.13 (20.72, 28.41)	30.11 (24.74, 33.17)
Zhejiang	28.33 (23.91, 31.96)	21.80 (18.69, 24.39)
Anhui	27.70 (24.25, 31.45)	22.48 (19.74, 25.70)
Fujian	29.34 (25.87, 32.30)	24.68 (22.09, 27.21)
Jiangxi	19.44 (17.09, 22.25)	16.30 (14.80, 18.37)
Shandong	7.01 (6.01, 8.86)	10.16 (8.72, 12.06)
Henan	9.26 (8.09, 10.18)	25.37 (22.45, 27.72)
Hubei	11.85 (9.84, 13.76)	27.58 (23.44, 31.23)
Hunan	27.56 (23.43, 30.19)	26.68 (22.87, 30.09)
Guangdong	44.91 (40.13, 48.66)	32.31 (27.48, 35.04)
Guangxi	27.21 (24.13, 29.87)	15.87 (14.62, 18.31)
Hainan	11.08 (10.42, 12.45)	21.17 (19.86, 23.53)
Chongqing	80.81 (69.14, 91.82)	22.51 (18.87, 25.49)
Sichuan	41.31 (37.66, 47.22)	38.30 (34.02, 42.82)
Guizhou	26.86 (24.08, 32.46)	24.48 (22.28, 28.61)
Yunnan	61.47 (57.91, 65.91)	26.48 (23.51, 29.16)
Tibet	30.88 (22.85, 38.39)	16.75 (13.19, 23.93)
Shaanxi	53.09 (45.31, 63.09)	40.84 (33.66, 49.20)
Gansu	20.29 (16.08, 24.02)	32.66 (25.79, 42.67)
Qinghai	6.98 (5.18, 9.03)	4.41 (3.18, 5.63)
Ningxia	4.12 (3.09, 4.70)	9.33 (7.67, 11.99)
Xinjiang	4.17 (3.34, 5.43)	24.49 (20.50, 30.63)
**National Average**	**26.63 (23.14, 30.40)**	**27.13 (23.15, 31.34)**

Shown by province and national average, weighted by provincial population size, for EV-A71 and CV-A16 from the two-serotype model with α = 0.95 and province-specific maximum likelihood estimates of cross-protection. Point estimate shown is the median ${\hat{\beta }}_{s}$, and the IQR is the 25th and 75th percentiles of ${\hat{\beta }}_{s}$.

In 27 of the 31 provinces, the maximum likelihood estimates from the two-serotype model indicate the existence of a transient cross-protection between the EV-A71 and CV-A16 serotypes (Table 2). The population-weighted national average suggests that infection with one serotype yields *k* = 9.95 wk (95% CI: 3.31, 23.40) of protection against infection with the other serotype in δ = 0.68 of the population (95% CI: 0.37, 0.96), resulting in an average duration of cross-protection of 6.77 wk (95% CI: 2.50, 10.03). We estimated likelihood surfaces of the cross-protection parameters by province to obtain 95% CIs (S15–S21 Figs).

**Table 2:** Cross-protection parameter estimates by province.

Province	$\hat{k}\;(95\%\;\mathrm{CI})$	$\hat{\delta }\;(95\%\;\mathrm{CI})$	$\hat{k}\cdot \hat{\delta }\;(95\%\;\mathrm{CI})$
Beijing	21 (9, 25)	1.00 (0.65, 1.00)	21.00 (8.25, 25.00)
Tianjin	22 (2, 31)	1.00 (0.40, 1.00)	22.00 (1.80, 31.00)
Hebei	4 (3, 4)	1.00 (0.80, 1.00)	4.00 (2.85, 3.80)
Shanxi	0 (0, 52)	0.05 (0.00, 1.00)	0.00 (0.00, 5.20)
Inner Mongolia	2 (0, 52)	0.05 (0.00, 1.00)	0.10 (0.00, 33.80)
Liaoning	4 (3, 8)	1.00 (0.70, 1.00)	4.00 (3.00, 8.00)
Jilin	2 (0, 52)	0.95 (0.00, 1.00)	1.90 (0.00, 3.20)
Heilongjiang	52 (0, 52)	0.10 (0.00, 1.00)	5.20 (0.00, 13.00)
Shanghai	9 (6, 18)	1.00 (0.60, 1.00)	9.00 (5.20, 18.00)
Jiangsu	8 (1, 14)	0.40 (0.10, 1.00)	3.20 (0.55, 7.65)
Zhejiang	8 (3, 9)	0.65 (0.40, 1.00)	5.20 (2.55, 8.00)
Anhui	9 (1, 16)	0.30 (0.05, 1.00)	2.70 (0.05, 7.65)
Fujian	6 (3, 8)	1.00 (0.80, 1.00)	6.00 (3.00, 8.00)
Jiangxi	0 (0, 52)	0.05 (0.00, 1.00)	0.00 (0.00, 4.50)
Shandong	4 (3, 7)	1.00 (0.65, 1.00)	4.00 (2.25, 7.00)
Henan	6 (4, 6)	1.00 (0.60, 1.00)	6.00 (3.00, 5.70)
Hubei	2 (0, 52)	1.00 (0.00, 1.00)	2.00 (0.00, 9.00)
Hunan	6 (5, 8)	0.35 (0.15, 0.55)	2.10 (0.90, 3.30)
Guangdong	11 (11, 12)	1.00 (1.00, 1.00)	11.00 (11.00, 12.00)
Guangxi	9 (8, 14)	1.00 (0.95, 1.00)	9.00 (8.00, 14.00)
Hainan	0 (0, 52)	0.05 (0.00, 1.00)	0.00 (0.00, 3.25)
Chongqing	3 (2, 52)	1.00 (0.05, 1.00)	3.00 (1.20, 6.90)
Sichuan	6 (5, 8)	1.00 (0.30, 1.00)	6.00 (1.80, 8.00)
Guizhou	52 (2, 52)	0.15 (0.05, 1.00)	7.80 (1.20, 15.60)
Yunnan	26 (6, 32)	0.20 (0.15, 0.55)	5.20 (2.25, 7.80)
Tibet	3 (2, 31)	1.00 (0.40, 1.00)	3.00 (1.40, 20.15)
Shaanxi	1 (0, 52)	0.75 (0.00, 1.00)	0.75 (0.00, 1.50)
Gansu	0 (0, 52)	0.05 (0.00, 1.00)	0.00 (0.00, 31.20)
Qinghai	26 (0, 52)	0.05 (0.00, 1.00)	1.30 (0.00, 13.75)
Ningxia	4 (1, 29)	0.75 (0.20, 1.00)	3.00 (0.40, 13.05)
Xinjiang	52 (0, 52)	0.15 (0.00, 1.00)	7.80 (0.00, 52.00)
**National Average**	**9.95 (3.31, 23.40)**	**0.68 (0.37, 0.96)**	**6.77 (2.50, 10.03)**

δ is the proportion of those infected with one serotype who are cross-protected against infection from all other serotypes for a fixed duration of *k* time steps, and the product *k* ⋅ δ represents the average duration of cross-protection. 95% CIs for the individual parameters are derived from the profile likelihood using the χ^2^ distribution with 1 degree of freedom.

**Fig 2:**
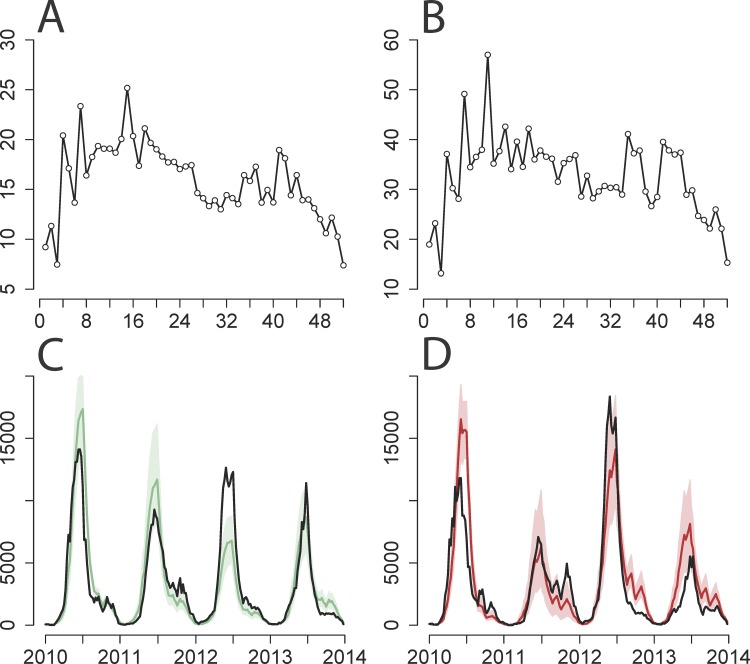
Two-serotype TSIR model fit for Beijing province. (A and B) Estimated ${\hat{\beta }}_{s}$ (*y*-axis) by week (*x*-axis) for (A) EV-A71 and (B) CV-A16. (C and D) Observed number of cases adjusted for reporting rate (*y*-axis) by week (years 2010–2013, *x*-axis) (black line) against predictions from 1,000 stochastic simulations of the entire time series for (C) EV-A71 and (D) CV-A16, showing median value (solid colored line) and 5th and 95th percentiles of the simulations (shaded area). Calculated with α = 0.95 and province-specific maximum likelihood estimates of cross-protection (*k* = 21 wk and δ = 1).

The two-serotype model performed well in predicting weekly incidence of both EV-A71 and CV-A16 in the representative province of Beijing, capturing both the shape and scale of incidence from 2010 to 2013 (Fig 2). A sensitivity analysis varying α values in the two-serotype model for Beijing suggests that 0.95 is a feasible estimate of α (S22–S30 Figs) and that it is able to capture the yearly epidemic cycles while maintaining spatial stochasticity [54]; the actual value of α has quantitative implications for the impact of vaccination, though the qualitative conclusions given below are robust. As α approaches the value of 1, the model behavior becomes erratic because the underlying Reed–Frost epidemic model is neutrally stable and the TSIR approximation breaks down [50]. Two-serotype model fits of the observed and simulated time series for the remaining provinces are provided in S8–S14 Figs.

The time series were too short to do out-of-sample cross-validation, but the robustness of our inference procedure was tested by stochastically simulating incidence data, fitting the model, and recovering parameter values. The log-linear regression model recovered more reliable estimates of $\bar{S}$ and β_*s*_ than the ordinary least squares (OLS) regression model with log-transformed incidence (S31 and S32 Figs). The estimated $\bar{S}$ in the OLS regression is biased high, and the likelihood surface is flattened; the generalized linear models (GLMs) can recover reliable estimates of $\bar{S}$, and the 95% profile likelihood intervals are more reasonable than those estimated from the OLS regression. The log-linear model was also able to robustly recover parameter values from simulated data incorporating various levels of cross-protection (S33 Fig).

### Geographic Variations in Transmission

There are two distinct geographic trends of HFMD in China: in the north, there is an annual peak in incidence in June, and in the south, there are bi-annual peaks in May and in September–October [15]. The models captured these variations and accurately predicted the annual peaks in incidence in the northern provinces (e.g., Beijing [S11A Fig] and Tianjin [S11B Fig]) and the bi-annual peaks in the southern provinces (e.g., Chongqing [S13A Fig] and Sichuan [S13B Fig]) for EV-A71 and CV-A16. We also explored the seasonal patterns of *R*_E_ along a latitudinal gradient, which suggest that in the north both serotypes tend to have one period each year when incidence is increasing and *R*_E_ is above 1, while in the south there tend to be two distinct periods within a year during which incidence is increasing in the population (S3 Fig).

Based on the two-serotype model, we found considerable seasonal and geographic heterogeneities in the estimated values of *R*_0_ (S2 and S3 Tables). We also disentangled the different sources of spatial and temporal variation in the transmission rate: within-week variation by province was estimated by the standard errors of the log(β_*s*_) terms from the log-linear regression in Eq 8 and was found to be generally low, between-week variation by province was assessed by the coefficient of variation in ${\hat{\beta }}_{s}$, and between-province variation was found to be generally high for both EV-A71 and CV-A16 (S1 Table; S2 Fig).

### Impact of EV-A71 Vaccination

Using our estimate of *R*_0_ of approximately 25 and the basic equation for calculating the critical vaccination threshold *p*_*c*_ = 1 − 1/*R*_0_ [55], we estimate that a majority of provinces would require vaccine coverage levels upwards of 96% of infants and young children to end ongoing transmission of EV-A71-associated HFMD. We simulated the effects of nationally introducing three types of EV-A71-containing vaccine on future EV-A71 and CV-A16 incidence (assuming *R*_0_ equals 25 for both serotypes and 90% vaccine coverage at birth as a base case): a broad monovalent EV-A71 vaccine (*k*_infection_ = *k*_vacc_ = 22 wk and δ_infection_ = δ_vacc_ = 1, the highest province-specific estimates of cross-protection from natural infection), a narrow monovalent EV-A71 vaccine conferring no cross-protection, and a narrow bivalent EV-A71, CV-A16 vaccine.

**Fig 3:**
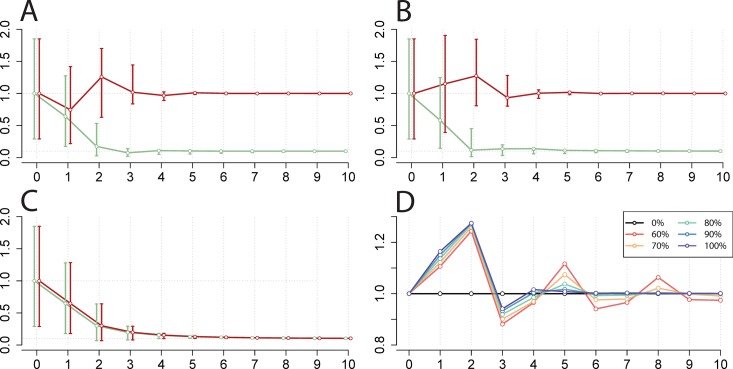
Simulation of national EV-A71 vaccination and corresponding change in CV-A16 incidence. (A–C) Relative change in incidence of EV-A71 (green) and CV-A16 (red) (*y*-axis) by year (*x*-axis) for 10 y following vaccine initiation compared to pre-vaccination equilibria (shown at year 0) in the two-serotype model, ignoring seasonality in β_*s*_. The circles indicate the output from the deterministic simulation, with error bars indicating the 5th and 95th percentiles of 500 stochastic simulations. Vaccine scenarios explored: (A) broad monovalent EV-A71 vaccine (administered at birth) achieving 90% coverage, (B) narrow monovalent EV-A71 vaccine achieving 90% coverage, and (C) narrow bivalent EV-A71, CV-A16 vaccine achieving 90% coverage. (D) Duration and magnitude of change in CV-A16 yearly incidence compared to pre-vaccination equilibria, as a function of narrow monovalent EV-A71 vaccine coverage (0%, 60%, 70%, 80%, 90%, and 100%). Calculated with α = 0.95 and the highest province-specific maximum likelihood estimates of cross-protection (*k*_infection_ = 22 wk and δ_infection_ = 1).

For all three vaccines, vaccination provides both a short- and long-term reduction in national EV-A71 incidence. While EV-A71 vaccination would not affect overall CV-A16 incidence in the long term, we found that a broad monovalent EV-A71 vaccine could cause a transient benefit in terms of reduced CV-A16 incidence due to cross-protective immunity (Fig 3A). The transient increase in CV-A16 in the second year following vaccine deployment reflects the end of a phenomenon known as a “honeymoon period,” during which susceptible individuals have slowly accumulated.

However, we found from our simulations that a narrow monovalent EV-A71 vaccine could cause a transient increase in national incidence and lead to a potential competitive release of CV-A16 (Fig 3B). This increase is due to the reduction in EV-A71 incidence that reduces the population levels of cross-immunity against CV-A16. The magnitude and duration of this increase following vaccination will depend on various factors including the achieved EV-A71 vaccine coverage (Fig 3D). Lastly, the deployment of a bivalent vaccine would lead to reductions in both EV-A71- and CV-A16-associated HFMD (Fig 3C).

Across individual provinces, we found that EV-A71 incidence decreases monotonically with higher vaccination coverage, and ongoing EV-A71 transmission approaches zero in the range of the critical vaccination threshold (S5A Fig). We also found that post-vaccination CV-A16 incidence increases slightly or remains comparable to its pre-vaccination levels with use of a narrow monovalent vaccine, and that the magnitude of this transient increase in CV-A16 incidence scales with EV-A71 vaccine coverage (S5B Fig). Further investigation of other combinations of cross-protection parameters led to the same qualitative conclusions (S6 and S7 Figs.).

### Additional Serotype Circulation

We extended the two-serotype model to address the co-circulation of non-EV-A71, non-CV-A16 serotypes of enterovirus as an additional third epidemic series, though it is likely to be a combination of two or more enteroviruses including CV-A6 and CV-A10 [56–58]. In comparison with the two-serotype model, the three-serotype model (S6 Table) fit as well or better in terms of the mean absolute yearly PE in 20 of the 31 provinces for EV-A71, and 22 of the 31 provinces for CV-A16 (S7 Table). We did not see qualitative differences in our results with the inclusion of this additional serotype epidemic series. Multi-serotype models generate more multi-collinearity among parameters and introduce statistical identifiability issues, since a number of parameter value combinations are similarly supported by the likelihood. There is an inverse relationship between $\bar{S}$ and $\bar{\beta }$ (and therefore incidence) that is mediated by cross-protection (S4 and S34 Figs). Therefore, we parsimoniously used province-level cross-protection estimates from the two-serotype model to parametrize the three-serotype model.

## Discussion

### Findings

The two-serotype TSIR model captured the observed dual epidemic cycles of EV-A71- and CV-A16-associated HFMD for the 31 provinces in China and the varying seasonal patterns of transmission in the northern and southern provinces of the country. Comparing values of the mean absolute yearly PE, we found that the two-serotype model with cross-protection fit the incidence data better on average than the separate one-serotype models; the public health message of HFMD as a highly transmissible infection remained consistent between models. Additionally, estimates of the cross-protection parameters across provinces suggest that HFMD serotypes provide a temporary immunizing effect against each other. However, our results suggest considerable geographic variability in this effect, pointing to the substantial value of further study. Our estimated national *R*_0_ of approximately 25 is consistent with the relatively low median age at HFMD infection [15], though any loss of immunity (see below) could also keep the age at primary infection low.

The simulations of EV-A71 vaccination showed that incidence would decrease monotonically with higher vaccine coverage, and suggest that a mass EV-A71 vaccination program targeted at infants and young children in China could greatly reduce the burden of HFMD caused by EV-A71. The high *R*_0_ values of HFMD serotypes imply that disease elimination is unlikely and translate to necessary coverage levels of above 96% to achieve population-level immunity. Additionally, by assuming 100% vaccine efficacy, this estimate of required vaccination coverage is yet an underestimation. A considerable increase in the prevalence of non-vaccine serotypes has been observed in other pathogens such as pneumococcal disease [40–42], but our two-serotype model results suggest that the incidence of CV-A16 following narrow monovalent EV-A71 vaccination would be expected to remain comparable to its pre-vaccination levels. However, a bivalent or polyvalent vaccine would be desirable in light of the co-circulation of several enteroviruses associated with HFMD [59,60] and because the extent of indirect protection afforded by vaccination would not be major at these relatively high levels of *R*_0_.

This analysis is novel in that it evaluates the existence and magnitude of cross-protection between different HFMD serotypes in a mathematical modeling context. Our framework allowed us to look for potential circulating serotype replacement by CV-A16 following vaccination against EV-A71. However, further studies are required to better understand the strength and breadth of immunity conferred by the EV-A71 vaccine relative to the levels of immunity following natural infection and to test for potential loss of immunity over time. While we acknowledge that 5 y may be too short a time series to definitively understand serotype interactions, based on our findings we are cautiously optimistic that monovalent EV-A71 vaccination is sufficient and will not significantly increase the risk of serotype replacement by CV-A16 in the population.

### Caveats and Directions for Future Work

Using this relatively simple mathematical model, we were able to detect a robust signature of herd immunity driving the outbreak dynamics of HFMD. Although unlikely to dominate the observed incidence dynamics, significant evidence for antigenic drift in EV-A71 indicates some potential loss of immunity, which could affect subsequent immune escape, particularly in the context of vaccination [61,62]. In general, protection (via a rise in neutralizing antibody titers) against a wide spectrum of EV-A71 sub-genotypes is observed shortly after vaccination regardless of whether children already had antibodies [63–65], but longer follow-up is required to assess whether immunity against the sub-genotypes other than the vaccine serotype wanes (more rapidly) over time, as seen in influenza and dengue virus. The current time series were too short to evaluate the extent of loss of immunity following infection (captured, for example, by SIRS epidemic models [66,67]); however, the quality of the fit underlines that our assumption of strong immunity is reasonable as a first step.

The scope of the data also did not allow us to explicitly disentangle infection with alternative serotypes such as CV-A6 and CV-A10, which are known to be currently co-circulating in China and throughout the Asia-Pacific region [68–70] and might show further interactions. The increasingly multi-collinear nature of cross-protection parameters when additional serotypes are included in the model poses an analytical challenge. However, if cross-protection were substantially stronger than our estimates, we might expect to see the epidemics be out of phase. Understanding the interactions between co-circulating serotypes would allow for more nuanced estimates of the risk of serotype replacement by non-vaccine serotypes. In this analysis, we assumed stationarity in the mean transmission rate between years because of the short time series, but there may be confounding effects of year-to-year variation in transmissibility on estimates of cross-immunity. However, our results are conservative in terms of assessing potential serotype replacement because such exogenous forcing would increase correlations in the incidence of different serotypes, which would in turn reduce apparent cross-protection.

Additionally, the absence of age structure in this formulation of the TSIR model did not allow us to assess the degree of loss of homologous and heterologous immunity over time or the age-focusing of vaccination. Subsequent models could be further refined to allow for heterogeneity in mixing and transmission by age, and to model different vaccination implementation strategies. Furthermore, serological surveys would be useful to better understand population-level susceptibility to HFMD.

The richness of the spatial scale of these data allow for future work to better understand spatial correlations in incidence and synchrony in the timing of epidemics [71]. These data will also be useful for evaluating the spatial scale (national, regional, provincial, etc.) at which vaccination efforts should be coordinated and deployed. A better understanding of the dynamics of serotype invasion and interaction is necessary for accurately predicting the effects of introducing a new HFMD vaccine formulation with another serotype of enterovirus in China.

Since the recent outbreaks of HFMD have been exclusive to the Asia-Pacific region, it will also be crucial to understand the potential competitive exclusion and invasion dynamics of HFMD and other enteroviral diseases in other parts of the world due to cross-protective immunity from other enteroviruses. Recent concerns with disease due to outbreaks of Enterovirus D68 in the US and other countries only increase the urgency of understanding the spatial and viral dynamics of this group of infections. A more accurate reflection of the sero-epidemiological and antigenic landscape [72] across HFMD and other enteroviral diseases will be a key component of efforts to reduce the clinical burden of HFMD and its associated economic costs in affected areas.

## Supporting Information

S1 Chinese Abstract Chinese translation of the abstract by QL and HY. (DOCX)

S1 Dataset Demographic data of China. Province identification number (ID) (for linking to S2 Dataset), yearly population size (in 10,000s), and yearly crude birth rate (per 1,000) by province and national total between 2009 and 2013.
(DOCX)

S2 Dataset HFMD case data. Estimated weekly case counts of EV-A71, CV-A16, and other non-EV-A71 and non-CV-A16 serotypes of enterovirus, for each province (linked to S1 Dataset via ID), from 1 January 2009 to 31 December 2013.
(CSV)

S1 Fig Aggregated proportion of laboratory-confirmed HFMD by serotype. Classified as proportion of EV-A71 (green), CV-A16 (red), or other non-EV-A71 and non-CV-A16 serotypes of enterovirus (blue) (*y*-axis) between 1 January 2009 and 31 December 2013, every 4 wk (*x*-axis), on a national scale (A) and by region: east region (B), south region (C), central region (D), north region (E), northwest region (F), southwest region (G), and northeast region (H).
(TIFF)

S2 FigSeasonal heterogeneities in *R*_0_.Range in estimated ${\hat{\beta }}_{s}$ values (*y*-axis) for each of the 52 wk of the year (*x*-axis) across all 31 provinces for (A) EV-A71 and (B) CV-A16 in the two-serotype model, using province-specific maximum likelihood estimates of cross-protection. Each data point represents a province.(TIFF)

S3 Fig Seasonal and geographic heterogeneities in *R*_E_. Estimated *R*_E_ values (*y*-axis) for each of the 52 wk of the year (*x*-axis) (each grid cell represents the median value of the 4 y from 2010 to 2013) by province for (A) EV-A71 and (B) CV-A16. Calculated with α = 0.95 and province-specific maximum likelihood estimates of cross-protection, with provinces ordered along the latitudinal gradient (north to south).
(TIFF)

S4 FigRelationship between epidemiological parameters in Beijing province.Estimated $\bar{S}$ (*x-*axis) against $\bar{\beta }$ (*y-*axis) for (A) EV-A71 and (B) CV-A16 in the two-serotype model for α = 0.95, under varying cross-protection levels (blue–red spectrum, where “None” represents *k* = 0 wk and δ = 0, and “Max” represents *k* = 52 wk and δ = 1).(TIFF)

S5 Fig Change in EV-A71 and CV-A16 incidence following narrow monovalent EV-A71 vaccination by province. (A) Relative change in yearly incidence of EV-A71 (deterministic simulation) by province (*y*-axis) 60 y following vaccine initiation compared to pre-vaccination equilibria, ignoring seasonality in β_*s*_, as a function of narrow EV-A71 vaccine coverage (*x-*axis). Calculated with α = 0.95 and province-specific maximum likelihood estimates of cross-protection, with provinces ordered by degree of cross-protection (high to low). (B) Maximum transient increase in yearly incidence of CV-A16 (deterministic simulation) by province (*y*-axis) following vaccine initiation compared to pre-vaccination equilibria, ignoring seasonality in β_*s*_, as a function of narrow EV-A71 vaccine coverage (*x-*axis). Calculated with α = 0.95 and province-specific maximum likelihood estimates of cross-protection, with provinces ordered by degree of cross-protection (high to low). Note that there is no change in the yearly incidence of CV-A16 after vaccination in provinces where the maximum likelihood estimates of cross-protection are zero.
(TIFF)

S6 Fig Simulation of national EV-A71 vaccination and corresponding change in CV-A16 incidence under the upper extreme cross-protection scenario. (A–C) Relative change in incidence of EV-A71 (green) and CV-A16 (red) (*y*-axis) by year (*x*-axis) for 10 y following vaccine initiation compared to pre-vaccination equilibria (at year 0) in the two-serotype model, ignoring seasonality in β_*s*_. The circles indicate the output from the deterministic simulation, with error bars showing the 5th and 95th percentiles of 500 stochastic simulations. Vaccine scenarios explored: (A) broad monovalent EV-A71 vaccine (administered at birth) achieving 90% coverage, (B) narrow monovalent EV-A71 vaccine achieving 90% coverage, and (C) narrow bivalent EV-A71, CV-A16 vaccine achieving 90% coverage. (D) Duration and magnitude of change in CV-A16 yearly incidence compared to pre-vaccination equilibria, as a function of narrow monovalent EV-A71 vaccine coverage (0%, 60%, 70%, 80%, 90%, and 100%). Calculated with α = 0.95, *k*_infection_ = 52 wk, and δ_infection_ = 1.
(TIF)

S7 Fig Simulation of national EV-A71 vaccination and corresponding change in CV-A16 incidence under the no cross-protection scenario. (A–C) Relative change in incidence of EV-A71 (green) and CV-A16 (red) (*y*-axis) by year (*x*-axis) for 10 y following vaccine initiation compared to pre-vaccination equilibria (at year 0) in the two-serotype model, ignoring seasonality in β_*s*_. The circles indicate the output from the deterministic simulation, with error bars showing the 5th and 95th percentiles of 500 stochastic simulations. Vaccine scenarios explored: (A) broad monovalent EV-A71 vaccine (administered at birth) achieving 90% coverage, (B) narrow monovalent EV-A71 vaccine achieving 90% coverage, and (C) narrow bivalent EV-A71, CV-A16 vaccine achieving 90% coverage. (D) Duration and magnitude of change in CV-A16 yearly incidence compared to pre-vaccination equilibria, as a function of narrow monovalent EV-A71 vaccine coverage (same value for all vaccine coverage levels). Calculated with α = 0.95, *k*_infection_ = 0 wk, and δ_infection_ = 0.
(TIF)

S8 Fig Two-serotype TSIR model fits in the east region. Observed number of cases adjusted for reporting rate (*y*-axis) by week (years 2010–2013, *x*-axis) (black line) against predictions from 1,000 stochastic simulations of the entire time series for EV-A71 (green) and CV-A16 (red), showing median value (solid colored line) and 5th and 95th percentiles of the simulations (shaded area). Showing individual provinces comprising the east region: (A) Shanghai, (B) Jiangsu, (C) Zhejiang, (D) Anhui, (E) Fujian, (F) Jiangxi, (G) Shandong. Calculated with α = 0.95 and province-specific maximum likelihood estimates of cross-protection.
(TIFF)

S9 Fig Two-serotype TSIR model fits in the south region. Observed number of cases adjusted for reporting rate (*y*-axis) by week (years 2010–2013, *x*-axis) (black line) against predictions from 1,000 stochastic simulations of the entire time series for EV-A71 (green) and CV-A16 (red), showing median value (solid colored line) and 5th and 95th percentiles of the simulations (shaded area). Showing individual provinces comprising the south region: (A) Guangdong, (B) Guangxi, (C) Hainan. Calculated with α = 0.95 and province-specific maximum likelihood estimates of cross-protection.
(TIFF)

S10 Fig Two-serotype TSIR model fits in the central region. Observed number of cases adjusted for reporting rate (*y*-axis) by week (years 2010–2013, *x*-axis) (black line) against predictions from 1,000 stochastic simulations of the entire time series for EV-A71 (green) and CV-A16 (red), showing median value (solid colored line) and 5th and 95th percentiles of the simulations (shaded area). Showing individual provinces comprising the central region: (A) Henan, (B) Hubei, (C) Hunan. Calculated with α = 0.95 and province-specific maximum likelihood estimates of cross-protection.
(TIFF)

S11 Fig Two-serotype TSIR model fits in the north region. Observed number of cases adjusted for reporting rate (*y*-axis) by week (years 2010–2013, *x*-axis) (black line) against predictions from 1,000 stochastic simulations of the entire time series for EV-A71 (green) and CV-A16 (red), showing median value (solid colored line) and 5th and 95th percentiles of the simulations (shaded area). Showing individual provinces comprising the north region: (A) Beijing, (B) Tianjin, (C) Hebei, (D) Shanxi, (E) Inner Mongolia. Calculated with α = 0.95 and province-specific maximum likelihood estimates of cross-protection.
(TIFF)

S12 Fig Two-serotype TSIR model fits in the northwest region. Observed number of cases adjusted for reporting rate (*y*-axis) by week (years 2010–2013, *x*-axis) (black line) against predictions from 1,000 stochastic simulations of the entire time series for EV-A71 (green) and CV-A16 (red), showing median value (solid colored line) and 5th and 95th percentiles of the simulations (shaded area). Showing individual provinces comprising the northwest region: (A) Shaanxi, (B) Gansu, (C) Qinghai, (D) Ningxia, (E) Xinjiang. Calculated with α = 0.95 and province-specific maximum likelihood estimates of cross-protection.
(TIFF)

S13 Fig Two-serotype TSIR model fits in the southwest region. Observed number of cases adjusted for reporting rate (*y*-axis) by week (years 2010–2013, *x*-axis) (black line) against predictions from 1,000 stochastic simulations of the entire time series for EV-A71 (green) and CV-A16 (red), showing median value (solid colored line) and 5th and 95th percentiles of the simulations (shaded area). Showing individual provinces comprising the southwest region: (A) Chongqing, (B) Sichuan, (C) Guizhou, (D) Yunnan, (E) Tibet. Calculated with α = 0.95 and province-specific maximum likelihood estimates of cross-protection.
(TIFF)

S14 Fig Two-serotype TSIR model fits in the northeast region. Observed number of cases adjusted for reporting rate (*y*-axis) by week (years 2010–2013, *x*-axis) (black line) against predictions from 1,000 stochastic simulations of the entire time series for EV-A71 (green) and CV-A16 (red), showing median value (solid colored line) and 5th and 95th percentiles of the simulations (shaded area). Showing individual provinces comprising the northeast region: (A) Liaoning, (B) Jilin, (C) Heilongjiang. Calculated with α = 0.95 and province-specific maximum likelihood estimates of cross-protection.
(TIFF)

S15 FigLog-likelihood surfaces of cross-protection parameters in the east region.Estimated log-likelihood values over a range of the cross-protection parameters *k*, from 0 to 52 (in weeks; *x-*axis), and δ, from 0 to 1 (as proportion; *y-*axis), in the two-serotype model. Calculated with α = 0.95, showing the maximum likelihood estimate (grid cell with “X”) and 95% confidence region (grid cells with asterisks) in individual provinces comprising the east region: (A) Shanghai, (B) Jiangsu, (C) Zhejiang, (D) Anhui, (E) Fujian, (F) Jiangxi, (G) Shandong. Grid cells highlighted pink are where $\bar{S}$ for either EV-A71 or CV-A16 is estimated to be 100%.(TIFF)

S16 FigLog-likelihood surfaces of cross-protection parameters in the south region.Estimated log-likelihood values over a range of the cross-protection parameters *k*, from 0 to 52 (in weeks; *x-*axis), and δ, from 0 to 1 (as proportion; *y-*axis), in the two-serotype model. Calculated with α = 0.95, showing the maximum likelihood estimate (grid cell with “X”) and 95% confidence region (grid cells with asterisks) in individual provinces comprising the south region: (A) Guangdong, (B) Guangxi, (C) Hainan. Grid cells highlighted pink are where $\bar{S}$ for either EV-A71 or CV-A16 is estimated to be 100%.(TIFF)

S17 FigLog-likelihood surfaces of cross-protection parameters in the central region.Estimated log-likelihood values over a range of the cross-protection parameters *k*, from 0 to 52 (in weeks; *x-*axis), and δ, from 0 to 1 (as proportion; *y-*axis), in the two-serotype model. Calculated with α = 0.95, showing the maximum likelihood estimate (grid cell with “X”) and 95% confidence region (grid cells with asterisks) in individual provinces comprising the central region: (A) Henan, (B) Hubei, (C) Hunan. Grid cells highlighted pink are where $\bar{S}$ for either EV-A71 or CV-A16 is estimated to be 100%.(TIFF)

S18 FigLog-likelihood surfaces of cross-protection parameters in the north region.Estimated log-likelihood values over a range of the cross-protection parameters *k*, from 0 to 52 (in weeks; *x-*axis), and δ, from 0 to 1 (as proportion; *y-*axis), in the two-serotype model. Calculated with α = 0.95, showing the maximum likelihood estimate (grid cell with “X”) and 95% confidence region (grid cells with asterisks) in individual provinces comprising the north region: (A) Beijing, (B) Tianjin, (C) Hebei, (D) Shanxi, (E) Inner Mongolia. Grid cells highlighted pink are where $\bar{S}$ for either EV-A71 or CV-A16 is estimated to be 100%.(TIFF)

S19 FigLog-likelihood surfaces of cross-protection parameters in the northwest region.Estimated log-likelihood values over a range of the cross-protection parameters *k*, from 0 to 52 (in weeks; *x-*axis), and δ, from 0 to 1 (as proportion; *y-*axis), in the two-serotype model. Calculated with α = 0.95, showing the maximum likelihood estimate (grid cell with “X”) and 95% confidence region (grid cells with asterisks) in individual provinces comprising the northwest region: (A) Shaanxi, (B) Gansu, (C) Qinghai, (D) Ningxia, (E) Xinjiang. Grid cells highlighted pink are where $\bar{S}$ for either EV-A71 or CV-A16 is estimated to be 100%.(TIFF)

S20 FigLog-likelihood surfaces of cross-protection parameters in the southwest region.Estimated log-likelihood values over a range of the cross-protection parameters *k*, from 0 to 52 (in weeks; *x-*axis), and δ, from 0 to 1 (as proportion; *y-*axis), in the two-serotype model. Calculated with α = 0.95, showing the maximum likelihood estimate (grid cell with “X”) and 95% confidence region (grid cells with asterisks) in individual provinces comprising the southwest region: (A) Chongqing, (B) Sichuan, (C) Guizhou, (D) Yunnan, (E) Tibet. Grid cells highlighted pink are where $\bar{S}$ for either EV-A71 or CV-A16 is estimated to be 100%.(TIFF)

S21 FigLog-likelihood surfaces of cross-protection parameters in the northeast region.Estimated log-likelihood values over a range of the cross-protection parameters *k*, from 0 to 52 (in weeks; *x-*axis), and δ, from 0 to 1 (as proportion; *y-*axis), in the two-serotype model. Calculated with α = 0.95, showing the maximum likelihood estimate (grid cell with “X”) and 95% confidence region (grid cells with asterisks) in individual provinces comprising the northeast region: (A) Liaoning, (B) Jilin, (C) Heilongjiang. Grid cells highlighted pink are where $\bar{S}$ for either EV-A71 or CV-A16 is estimated to be 100%.(TIFF)

S22 FigSensitivity analysis varying α values in the two-serotype TSIR model for Beijing province: α = 0.91.(A and B) Estimated ${\hat{\beta }}_{s}$ (*y*-axis) for (A) EV-A71 and (B) CV-A16 by week (*x*-axis). (C and D) Observed number of cases adjusted for reporting rate (*y*-axis) from 2010 to 2013 (black line) by week (*x*-axis) against predictions from 1,000 stochastic simulations of the entire time series for (C) EV-A71 and (D) CV-A16, showing median value (solid colored line) and 5th and 95th percentiles of the simulations (shaded area). (E and F) Output from deterministic simulation of incidence (*y*-axis) of (E) EV-A71 and (F) CV-A16 by year (*x*-axis) for 20 y before to 30 y following vaccine initiation (dotted black line, at year 0), normalized by serotype-specific yearly incidence in year −20 and ignoring seasonality in β_*s*_. Vaccination assumed to be narrow monovalent EV-A71 vaccine (administered at birth) achieving 90% coverage. $\bar{S}$ for EV-A71 = 0.112 and $\bar{S}$ for CV-A16 = 0.052. Calculated with province-specific maximum likelihood estimates of cross-protection (*k* = 21 wk and δ = 1).(TIFF)

S23 FigSensitivity analysis varying α values in the two-serotype TSIR model for Beijing province: α = 0.92.(A and B) Estimated ${\hat{\beta }}_{s}$ (*y*-axis) for (A) EV-A71 and (B) CV-A16 by week (*x*-axis). (C and D) Observed number of cases adjusted for reporting rate (*y*-axis) from 2010 to 2013 (black line) by week (*x*-axis) against predictions from 1,000 stochastic simulations of the entire time series for (C) EV-A71 and (D) CV-A16, showing median value (solid colored line) and 5th and 95th percentiles of the simulations (shaded area). (E and F) Output from deterministic simulation of incidence (*y*-axis) of (E) EV-A71 and (F) CV-A16 by year (*x*-axis) for 20 y before to 30 y following vaccine initiation (dotted black line, at year 0), normalized by serotype-specific yearly incidence in year −20 and ignoring seasonality in β_*s*_. Vaccination assumed to be narrow monovalent EV-A71 vaccine (administered at birth) achieving 90% coverage. $\bar{S}$ for EV-A71 = 0.107 and $\bar{S}$ for CV-A16 = 0.051. Calculated with province-specific maximum likelihood estimates of cross-protection (*k* = 21 wk and δ = 1).(TIFF)

S24 FigSensitivity analysis varying α values in the two-serotype TSIR model for Beijing province: α = 0.93.(A and B) Estimated ${\hat{\beta }}_{s}$ (*y*-axis) for (A) EV-A71 and (B) CV-A16 by week (*x*-axis). (C and D) Observed number of cases adjusted for reporting rate (*y*-axis) from 2010 to 2013 (black line) by week (*x*-axis) against predictions from 1,000 stochastic simulations of the entire time series for (C) EV-A71 and (D) CV-A16, showing median value (solid colored line) and 5th and 95th percentiles of the simulations (shaded area). (E and F) Output from deterministic simulation of incidence (*y*-axis) of (E) EV-A71 and (F) CV-A16 by year (*x*-axis) for 20 y before to 30 y following vaccine initiation (dotted black line, at year 0), normalized by serotype-specific yearly incidence in year −20 and ignoring seasonality in β_*s*_. Vaccination assumed to be narrow monovalent EV-A71 vaccine (administered at birth) achieving 90% coverage. $\bar{S}$ for EV-A71 = 0.102 and $\bar{S}$ for CV-A16 = 0.049. Calculated with province-specific maximum likelihood estimates of cross-protection (*k* = 21 wk and δ = 1).(TIFF)

S25 FigSensitivity analysis varying α values in the two-serotype TSIR model for Beijing province: α = 0.94.(A and B) Estimated ${\hat{\beta }}_{s}$ (*y*-axis) for (A) EV-A71 and (B) CV-A16 by week (*x*-axis). (C and D) Observed number of cases adjusted for reporting rate (*y*-axis) from 2010 to 2013 (black line) by week (*x*-axis) against predictions from 1,000 stochastic simulations of the entire time series for (C) EV-A71 and (D) CV-A16, showing median value (solid colored line) and 5th and 95th percentiles of the simulations (shaded area). (E and F) Output from deterministic simulation of incidence (*y*-axis) of (E) EV-A71 and (F) CV-A16 by year (*x*-axis) for 20 y before to 30 y following vaccine initiation (dotted black line, at year 0), normalized by serotype-specific yearly incidence in year −20 and ignoring seasonality in β_*s*_. Vaccination assumed to be narrow monovalent EV-A71 vaccine (administered at birth) achieving 90% coverage. $\bar{S}$ for EV-A71 = 0.098 and $\bar{S}$ for CV-A16 = 0.047. Calculated with province-specific maximum likelihood estimates of cross-protection (*k* = 21 wk and δ = 1).(TIFF)

S26 FigSensitivity analysis varying α values in the two-serotype TSIR model for Beijing province: α = 0.95 (used in main text).(A and B) Estimated ${\hat{\beta }}_{s}$ (*y*-axis) for (A) EV-A71 and (B) CV-A16 by week (*x*-axis). (C and D) Observed number of cases adjusted for reporting rate (*y*-axis) from 2010 to 2013 (black line) by week (*x*-axis) against predictions from 1,000 stochastic simulations of the entire time series for (C) EV-A71 and (D) CV-A16, showing median value (solid colored line) and 5th and 95th percentiles of the simulations (shaded area). (E and F) Output from deterministic simulation of incidence (*y*-axis) of (E) EV-A71 and (F) CV-A16 by year (*x*-axis) for 20 y before to 30 y following vaccine initiation (dotted black line, at year 0), normalized by serotype-specific yearly incidence in year −20 and ignoring seasonality in β_*s*_. Vaccination assumed to be narrow monovalent EV-A71 vaccine (administered at birth) achieving 90% coverage. $\bar{S}$ for EV-A71 = 0.094 and $\bar{S}$ for CV-A16 = 0.046. Calculated with province-specific maximum likelihood estimates of cross-protection (*k* = 21 wk and δ = 1).(TIFF)

S27 FigSensitivity analysis varying α values in the two-serotype TSIR model for Beijing province: α = 0.96.(A and B) Estimated ${\hat{\beta }}_{s}$ (*y*-axis) for (A) EV-A71 and (B) CV-A16 by week (*x*-axis). (C and D) Observed number of cases adjusted for reporting rate (*y*-axis) from 2010 to 2013 (black line) by week (*x*-axis) against predictions from 1,000 stochastic simulations of the entire time series for (C) EV-A71 and (D) CV-A16, showing median value (solid colored line) and 5th and 95th percentiles of the simulations (shaded area). (E and F) Output from deterministic simulation of incidence (*y*-axis) of (E) EV-A71 and (F) CV-A16 by year (*x*-axis) for 20 y before to 30 y following vaccine initiation (dotted black line, at year 0), normalized by serotype-specific yearly incidence in year −20 and ignoring seasonality in β_*s*_. Vaccination assumed to be narrow monovalent EV-A71 vaccine (administered at birth) achieving 90% coverage. $\bar{S}$ for EV-A71 = 0.091 and $\bar{S}$ for CV-A16 = 0.045. Calculated with province-specific maximum likelihood estimates of cross-protection (*k* = 21 wk and δ = 1).(TIFF)

S28 FigSensitivity analysis varying α values in the two-serotype TSIR model for Beijing province: α = 0.97.(A and B) Estimated ${\hat{\beta }}_{s}$ (*y*-axis) for (A) EV-A71 and (B) CV-A16 by week (*x*-axis). (C and D) Observed number of cases adjusted for reporting rate (*y*-axis) from 2010 to 2013 (black line) by week (*x*-axis) against predictions from 1,000 stochastic simulations of the entire time series for (C) EV-A71 and (D) CV-A16, showing median value (solid colored line) and 5th and 95th percentiles of the simulations (shaded area). (E and F) Output from deterministic simulation of incidence (*y*-axis) of (E) EV-A71 and (F) CV-A16 by year (*x*-axis) for 20 y before to 30 y following vaccine initiation (dotted black line, at year 0), normalized by serotype-specific yearly incidence in year −20 and ignoring seasonality in β_*s*_. Vaccination assumed to be narrow monovalent EV-A71 vaccine (administered at birth) achieving 90% coverage. $\bar{S}$ for EV-A71 = 0.087 and $\bar{S}$ for CV-A16 = 0.044. Calculated with province-specific maximum likelihood estimates of cross-protection (*k* = 21 wk and δ = 1).(TIFF)

S29 FigSensitivity analysis varying α values in the two-serotype TSIR model for Beijing province: α = 0.98.(A and B) Estimated ${\hat{\beta }}_{s}$ (*y*-axis) for (A) EV-A71 and (B) CV-A16 by week (*x*-axis). (C and D) Observed number of cases adjusted for reporting rate (*y*-axis) from 2010 to 2013 (black line) by week (*x*-axis) against predictions from 1,000 stochastic simulations of the entire time series for (C) EV-A71 and (D) CV-A16, showing median value (solid colored line) and 5th and 95th percentiles of the simulations (shaded area). (E and F) Output from deterministic simulation of incidence (*y*-axis) of (E) EV-A71 and (F) CV-A16 by year (*x*-axis) for 20 y before to 30 y following vaccine initiation (dotted black line, at year 0), normalized by serotype-specific yearly incidence in year −20 and ignoring seasonality in β_*s*_. Vaccination assumed to be narrow monovalent EV-A71 vaccine (administered at birth) achieving 90% coverage. $\bar{S}$ for EV-A71 = 0.084 and $\bar{S}$ for CV-A16 = 0.042. Calculated with province-specific maximum likelihood estimates of cross-protection (*k* = 21 wk and δ = 1).(TIFF)

S30 FigSensitivity analysis varying α values in the two-serotype TSIR model for Beijing province: α = 0.99.(A and B) Estimated ${\hat{\beta }}_{s}$ (*y*-axis) for (A) EV-A71 and (B) CV-A16 by week (*x*-axis). (C and D) Observed number of cases adjusted for reporting rate (*y*-axis) from 2010 to 2013 (black line) by week (*x*-axis) against predictions from 1,000 stochastic simulations of the entire time series for (C) EV-A71 and (D) CV-A16, showing median value (solid colored line) and 5th and 95th percentiles of the simulations (shaded area). (E and F) Output from deterministic simulation of incidence (*y*-axis) of (E) EV-A71 and (F) CV-A16 by year (*x*-axis) for 20 y before to 30 y following vaccine initiation (dotted black line, at year 0), normalized by serotype-specific yearly incidence in year −20 and ignoring seasonality in β_*s*_. Vaccination assumed to be narrow monovalent EV-A71 vaccine (administered at birth) achieving 90% coverage. $\bar{S}$ for EV-A71 = 0.081 and $\bar{S}$ for CV-A16 = 0.041. Calculated with province-specific maximum likelihood estimates of cross-protection (*k* = 21 wk and δ = 1).(TIFF)

S31 FigSimulated time series for TSIR simulation studies with no cross-protection.Representative stochastically simulated, single-serotype 40-y time series data of incidence (*y*-axis) by week (*x*-axis) ($\bar{S}=0.128$) (A) with weekly varying (*x*-axis), stationary β_*s*_ ($\bar{\beta }=\;9.407$) (*y*-axis) (B).(TIFF)

S32 FigResults of TSIR simulation studies with no cross-protection.Calculated with α = 0.95, *k* = 0 wk, and δ = 0, using data from S31 Fig. (A–C) Estimated β_*s*_ (*y*-axis) by week (*x*-axis) for this single time series with three regression models: (A) OLS regression with log-transformed data ($\bar{\beta }$ estimated to be 3.352), (B) Poisson GLM with log link ($\bar{\beta }$ estimated to be 11.224), and (C) quasi-Poisson GLM with log link ($\bar{\beta }$ estimated to be 11.122). (D–F) Estimated $\bar{S}$ for this single time series (*x-*axis) and 95% CIs (derived from the profile likelihood [*y*-axis] using the χ^2^ distribution with 1 degree of freedom) with three regression models: (D) OLS regression with log-transformed data (0.360, 95% CI: 0.088, 1.000), with negative log-likelihood on the *y-*axis; (E) Poisson GLM with log link (0.109, 95% CI: 0.095, 0.127), with negative log-likelihood on the *y-*axis; and (F) quasi-Poisson GLM with log link (0.109, 95% CI: 0.099, 0.121), with deviance on the *y-*axis. (G–I) Distribution of recovered $\bar{S}$ values (*x*-axis) from 1,000 stochastically simulated time series ($\bar{S}=0.128$ and seasonally varying, stationary β_*s*_, with $\bar{\beta }=\;9.407$ as in S31 Fig) with three regression models: (G) OLS regression with log-transformed data, (H) Poisson GLM with log link, and (I) quasi-Poisson GLM with log link. (J–L) Distribution of recovered $\bar{\beta }$ values (*x*-axis) from 1,000 stochastically simulated time series ($\bar{S}=0.128$ and seasonally varying, stationary β_*s*_, with $\bar{\beta }=\;9.407$ as in S31 Fig) with three regression models: (J) OLS regression with log-transformed data, (K) Poisson GLM with log link, and (L) quasi-Poisson GLM with log link.(TIFF)

S33 Fig Results of TSIR simulation studies with cross-protection. Estimated log-likelihood values over a range of the cross-protection parameters *k*, from 0 to 52 (in weeks; *x-*axis), and δ, from 0 to 1 (as proportion; *y-*axis), in the two-serotype model for α = 0.95, showing the maximum likelihood estimate (grid cell with “X”) and 95% confidence region (grid cells with asterisk) for simulated two-serotype data with true cross-protection parameter values of (A) *k* = 0 wk and δ = 0 (no cross-protection), (B) *k* = 12 wk and δ = 1, (C) *k* = 24 wk and δ = 1, and (D) *k* = 48 wk and δ = 1.
(TIFF)

S34 FigScatterplot matrix of demographic and epidemiological parameters.Each data point represents a province; from top left: mean population size from 2009 to 2013, mean births per week from 2009 to 2013, ratio of total reported EV-A71 cases to total births from 2009 to 2013, ratio of total reported CV-A16 cases to total births from 2009 to 2013, estimated $\bar{S}$ for EV-A71 and CV-A16, estimated reporting rate ρ for EV-A71 and CV-A16, and estimated $\bar{\beta }$ for EV-A71 and CV-A16. Calculated with α = 0.95 and the province-specific maximum likelihood estimates of cross-protection, using the two-serotype model.(TIFF)

S1 TableTwo-serotype TSIR model estimates for EV-A71 and CV-A16.Mean proportion of individuals that are susceptible to EV-A71 and CV-A16 ($\bar{S}$), reporting rate of EV-A71 and CV-A16 (ρ), mean weekly estimated transmission rate of EV-A71 and CV-A16 ($\bar{\beta }$), and coefficient of variation (CV) in estimated transmission rate of EV-A71 and CV-A16 by province. Calculated with α = 0.95 and province-specific maximum likelihood estimates of cross-protection. The 95% CIs for $\bar{S}$ are derived from the profile likelihood using the χ^2^ distribution with 1 degree of freedom; the 95% CIs for ρ are derived from the standard errors for the coefficient 1/ρ in the OLS regression of cumulative births and cumulative cases; the maximum standard errors (se) of the log(β_*s*_) terms across weekly ${\hat{\beta }}_{s}$ values are from the log-linear regression, representing variability around estimated β_*s*_.(DOCX)

S2 TableSpatial and temporal variation in *R*_0_ of EV-A71 across provinces.
${\hat{\beta }}_{s}$ and 95% CIs on each weekly β_*s*_ value (row) for EV-A71 for each province (column) from the two-serotype model with α = 0.95 and province-specific maximum likelihood estimates of cross-protection.(DOCX)

S3 TableSpatial and temporal variation in *R*_0_ of CV-A16 across provinces.
${\hat{\beta }}_{s}$ and 95% CIs on each weekly β_*s*_ value (row) for CV-A16 for each province (column) from the two-serotype model with α = 0.95 and province-specific maximum likelihood estimates of cross-protection.(DOCX)

S4 Table Omission of co-infection from the two-serotype model. We omit co-infection from this analysis, as co-infection of a single individual with both serotypes (EV-A71 and CV-A16) is rarer than expected by chance, given a reasonable sample size of EV-A71 and CV-A16 infections in a given year. We determined this using data from two published studies and χ^2^ tests.
(DOCX)

S5 TableOne-serotype TSIR model estimates for EV-A71 and CV-A16.Mean proportion of individuals that are susceptible to EV-A71 and CV-A16 ($\bar{S}$), reporting rate of EV-A71 and CV-A16 (ρ), mean weekly estimated transmission rate of EV-A71 and CV-A16 ($\bar{\beta }$), and coefficient of variation (CV) in estimated transmission rate of EV-A71 and CV-A16 by province. Calculated with α = 0.95. The 95% CIs for $\bar{S}$ are derived from the profile likelihood using the χ^2^ distribution with 1 degree of freedom; the 95% CIs for ρ are derived from the standard errors for the coefficient 1/ρ in the OLS regression of cumulative births and cumulative cases; the maximum standard errors (se) of the log(β_*s*_) terms across weekly ${\hat{\beta }}_{s}$ values are from the log-linear regression, representing variability around estimated β_*s*_.(DOCX)

S6 TableThree-serotype TSIR model estimates for EV-A71 and CV-A16.Mean proportion of individuals that are susceptible to EV-A71 and CV-A16 ($\bar{S}$), reporting rate of EV-A71 and CV-A16 (ρ), mean weekly estimated transmission rate of EV-A71 and CV-A16 ($\bar{\beta }$), and coefficient of variation (CV) in estimated transmission rate of EV-A71 and CV-A16 by province. Calculated with α = 0.95 and province-specific maximum likelihood estimates of cross-protection from the two-serotype model. The 95% CIs for $\bar{S}$ are derived from the profile likelihood using the *χ* ^2^ distribution with 1 degree of freedom; the 95% CIs for ρ are derived from the standard errors for the coefficient 1/ρ in the OLS regression of cumulative births and cumulative cases; the maximum standard errors (se) of the log(β_*s*_) terms across weekly ${\hat{\beta }}_{s}$ values are from the log-linear regression, representing variability around estimated β_*s*_.(DOCX)

S7 Table Within-province estimates of mean absolute yearly prediction error between observed and simulated weekly incidence. Shown for both EV-A71 and CV-A16 from 2010 to 2013, for the one-, two-, and three-serotype models with α = 0.95 and province-specific maximum likelihood estimates of cross-protection from the two-serotype model. Values with asterisks indicate lower PE for the serotype in the two-serotype model compared to the one-serotype model, and values with carets indicate lower PE for the serotype in the three-serotype model compared to the two-serotype model.
(DOCX)

S8 Table Within-province estimates of the coefficient of determination (*R* ^2^) between observed and simulated weekly incidence. Shown for both EV-A71 and CV-A16 from 2010 to 2013, for the one-, two-, and three-serotype models with α = 0.95 and province-specific maximum likelihood estimates of cross-protection from the two-serotype model.
(DOCX)

## Abbreviations

China CDC: Chinese Center for Disease Control and Prevention
CI: confidence interval
CV-A16: Coxsackievirus A16
EV-A71: Enterovirus A71
GLM: generalized linear model
HFMD: hand, foot, and mouth disease
IQR: interquartile range
OLS: ordinary least squares
PE: prediction error
SIR: susceptible–infected–recovered
SIRS: susceptible–infected–recovered–susceptible
TSIR: time series susceptible–infected–recovered
