# Supplementary material for: Hand, Foot, and Mouth Disease in China: Modeling Epidemic Dynamics of Enterovirus Serotypes and Implications for Vaccination
Source: PLoS Med. 2016 Feb 16;13(2):e1001958. doi: 10.1371/journal.pmed.1001958 (PMC4755668; doi:10.1371/journal.pmed.1001958)
Supplement: S1 Chinese Abstract — (DOCX) [file pmed.1001958.s001.docx]

**中国手足口病：相关肠道病毒血清型的传播动力学模型研究及其对疫苗免疫策略的启示**

**Hand, foot, and mouth disease in China: modelling epidemic dynamics of enterovirus serotypes and implications for vaccination**

Saki Takahashi^1^, 廖巧红^2^, Thomas P. Van Boeckel^1^, 邢薇佳^2^, 孙军玲^2^, Victor Y. Hsiao^1^, C. Jessica E. Metcalf^1,3^, 常昭瑞^2^, 刘凤凤^2^, 张静^2^, Joseph T. Wu^4^, Benjamin J. Cowling^4^, Gabriel M. Leung^4^, Jeremy J. Farrar^5^, H. Rogier van Doorn^5,6^, Bryan T. Grenfell^1,7,*^, 余宏杰^2,*^.

**作者单位**

1. Department of Ecology and Evolutionary Biology, Princeton University, Princeton, New Jersey, USA.

2.中国疾病预防控制中心传染病预防控制处、传染病监测预警重点实验室，北京，中国。

3. Woodrow Wilson School of Public and International Affairs, Princeton University, Princeton, New Jersey, USA.

4. WHO Collaborating Centre for Infectious Disease Epidemiology and Control, School of Public Health, Li Ka Shing Faculty of Medicine, The University of Hong Kong, Hong Kong Special Administrative Region, China.

5. Oxford University Clinical Research Unit, Hospital for Tropical Diseases, Ho Chi Minh City, Viet Nam.

6. Centre for Tropical Medicine, Nuffield Department of Medicine, University of Oxford, Oxford, UK.

7. Fogarty International Center, National Institutes of Health, Bethesda, Maryland, USA.

*通信:yuhj@chinacdc.cn或者grenfell@princeton.edu。

摘要

背景

手足口病是由小核糖核酸病毒科肠道病毒属肠道病毒A组和B组多种血清型引起的儿童常见疾病。过去15年中，手足口病在东亚和东南亚地区造成严重的疾病负担。2008-2013年，中国共报告了约900万例手足口病，其主要致病原是Enterovirus A71 (EV-A71) and Coxsackievirus A16 (CV-A16)。三个近期开展的III期临床试验证实了中国生产的单价EV-A71灭活疫苗对EV-A71相关的手足口病有高度的保护效力，但对CV-A16引起的手足口病没有保护作用。为更好地引导疫苗政策，本研究利用数学模型评估了疫苗使用对EV-A71相关手足口病的预期效果，以及由CV-A16血清型替换带来的潜在风险。同时，我们还扩展并拟合了非EV-A71和非CV-A16的其他肠道病毒血清型共同循环的模型，并分析其对疫苗免疫策略的影响。

方法和结果

本研究使用2009-2013年中国31个省（自治区、直辖市）手足口病周报告发病数，对多种血清型的时间序列易感-感染-康复（TSIR）流行病学模型进行了拟合。两种血清型交叉保护的TSIR模型拟合效果极佳，能够捕获省级水平传播的季节性和空间异质性，且实际观察发病序列与模型拟合的发病序列的相关性较强。经各省人口数加权处理，估算全国EV-A71和CV-A16的基本复制指数（R_0_）分别为26.63 (四分位数间距: 23.14- 30.40)和27.13 (四分位数间距: 23.15- 31.34) 。虽然各省间估计的基本复制指数有一定差异，但EV-A71疫苗免疫的整体预期效果针对此差异仍是稳定的。EV-A71疫苗覆盖率越高，预测的EV-A71相关手足口病发病数下降越显著；EV-A71疫苗使用后，各省CV-A16相关手足口病发病数与疫苗使用前的水平相近或仅有轻微上升。EV-A71或 CV-A16感染后的交叉免疫保护在68%（95%可信区间：37%-96%）的人群中可持续9.95周（95%可信区间：3.39-23.40）。本研究的预测结果受到发病时间序列较短、标本采集量较少、可能存在其他未鉴定血清型的肠道病毒共同循环等因素影响，但敏感性分析结果表明预测结果是稳定的，疫苗可显著降低EV-A71的发病水平，且CV-A16的发病水平不会出现明显的、竞争性的上升。

结论

本研究所用模型有效地拟合了实际观察的发病序列，提示群体免疫驱动了由多种肠道病毒血清型引起的手足口病流行。研究结果表明EV-A71 和 CV-A16 两种血清型间可产生短暂的交叉免疫保护效果。为消除EV-A71持续循环，EV-A71疫苗接种需达到较高的接种率。与EV-A71疫苗接种所带来的EV-A71 相关手足口病疾病负担的下降程度相比，该疫苗接种后的CV-A16 血清型替换是短暂的，且由此引起的CV-A16发病数上升是轻微的。因此，在婴幼儿中实施大规模EV-A71疫苗免疫接种，将会显著降低手足口病的总体疾病负担。
