# Supplementary material for: Hand, Foot, and Mouth Disease in China: Modeling Epidemic Dynamics of Enterovirus Serotypes and Implications for Vaccination
Source: PLoS Med. 2016 Feb 16;13(2):e1001958. doi: 10.1371/journal.pmed.1001958 (PMC4755668; doi:10.1371/journal.pmed.1001958)
Supplement: S1 Dataset — Province identification number (ID) (for linking to S2 Dataset), yearly population size (in 10,000s), and yearly crude birth rate (per 1,000) by province and national total between 2009 and 2013. (DOCX) [file pmed.1001958.s002.docx]

**S1 Dataset.** **Demographic data of China.** Province identification number (ID) (for linking to S2 Dataset), yearly population size (in 10,000s), and yearly crude birth rate (CBR) (per 1,000) by province and national total between 2009 and 2013.

| Province | ID | 2009  Population | 2010  Population | 2011  Population | 2012  Population | 2013  Population | 2009 CBR | 2010 CBR | 2011 CBR | 2012 CBR | 2013 CBR |
| --- | --- | --- | --- | --- | --- | --- | --- | --- | --- | --- | --- |
| Beijing | 11 | 1755 | 1961.9 | 2018.6 | 2069.3 | 2114.8 | 8.06 | 7.48 | 8.29 | 9.05 | 8.93 |
| Tianjin | 12 | 1228.16 | 1299.29 | 1355 | 1413.15 | 1472.21 | 8.30 | 8.18 | 8.58 | 8.75 | 8.28 |
| Hebei | 13 | 7034.4 | 7193.6 | 7240.51 | 7287.51 | 7332.61 | 12.93 | 13.22 | 13.02 | 12.88 | 13.04 |
| Shanxi | 14 | 3427.36 | 3574.115 | 3593 | 3610.83 | 3629.8 | 10.87 | 10.68 | 10.47 | 10.7 | 10.81 |
| Inner Mongolia | 15 | 2422.07 | 2472.179 | 2481.71 | 2489.85 | 2497.61 | 9.57 | 9.30 | 8.94 | 9.17 | 8.98 |
| Liaoning | 21 | 4319 | 4374.9 | 4383 | 4389 | 4390 | 6.06 | 6.68 | 5.71 | 6.15 | 6.09 |
| Jilin | 22 | 2739.55 | 2746.602 | 2749.41 | 2750.4 | 2751.28 | 6.69 | 7.91 | 6.53 | 5.73 | 5.36 |
| Heilongjiang | 23 | 3826 | 3833.402 | 3834 | 3834 | 3835.02 | 7.48 | 7.35 | 6.99 | 7.30 | 6.86 |
| Shanghai | 31 | 1921 | 2302.661 | 2347.46 | 2380.43 | 2415.15 | 8.64 | 7.05 | 6.97 | 9.56 | 8.18 |
| Jiangsu | 32 | 7725 | 7869.34 | 7898.8 | 7919.98 | 7939.49 | 9.55 | 9.73 | 9.59 | 9.44 | 9.44 |
| Zhejiang | 33 | 5180 | 5446.51 | 5463 | 5477 | 5498 | 10.22 | 10.27 | 9.47 | 10.12 | 10.01 |
| Anhui | 34 | 6131 | 5956.71 | 5968 | 5988 | 6029.8 | 13.07 | 12.70 | 12.23 | 13.00 | 12.88 |
| Fujian | 35 | 3627 | 3693 | 3720 | 3748 | 3774 | 12.20 | 11.27 | 11.41 | 12.74 | 12.20 |
| Jiangxi | 36 | 4432.158 | 4462.249 | 4488.437 | 4503.932 | 4522.15 | 13.87 | 13.72 | 13.48 | 13.46 | 13.19 |
| Shandong | 37 | 9470.3 | 9587.864 | 9637 | 9684.97 | 9733.39 | 11.70 | 11.65 | 11.50 | 11.90 | 11.41 |
| Henan | 41 | 9487 | 9405.47 | 9388 | 9406 | 9413.35 | 11.45 | 11.52 | 11.56 | 11.87 | 12.27 |
| Hubei | 42 | 5720 | 5727.914 | 5757.5 | 5779 | 5799 | 9.48 | 10.36 | 10.39 | 11.00 | 11.08 |
| Hunan | 43 | 6406 | 6570.1 | 6595.6 | 6638.93 | 6690.6 | 13.05 | 13.10 | 13.35 | 13.58 | 13.50 |
| Guangdong | 44 | 9638 | 10440.96 | 10504.85 | 10594 | 10644 | 11.78 | 11.18 | 10.45 | 11.60 | 10.71 |
| Guangxi | 45 | 4856 | 4610 | 4645 | 4682 | 4719 | 14.17 | 14.13 | 13.71 | 14.20 | 14.28 |
| Hainan | 46 | 864.07 | 868.5512 | 877.34 | 886.55 | 895.28 | 14.66 | 14.71 | 14.72 | 14.66 | 14.59 |
| Chongqing | 50 | 2859 | 2884.62 | 2919 | 2945 | 2970 | 9.90 | 9.17 | 9.88 | 10.86 | 10.37 |
| Sichuan | 51 | 8185 | 8044.92 | 8050 | 8076.2 | 8107 | 9.15 | 8.93 | 9.79 | 9.89 | 9.90 |
| Guizhou | 52 | 3798 | 3478.941 | 3468.72 | 3484.07 | 3502.22 | 13.65 | 13.96 | 13.31 | 13.27 | 13.05 |
| Yunnan | 53 | 4571 | 4601.6 | 4630.8 | 4659 | 4686.6 | 12.53 | 13.10 | 12.71 | 12.63 | 12.60 |
| Tibet | 54 | 290.03 | 300.7218 | 303.3 | 307.62 | 312.04 | 15.31 | 15.80 | 15.39 | 15.48 | 15.77 |
| Shaanxi | 61 | 3772 | 3735.227 | 3742.6 | 3753.09 | 3764 | 10.24 | 9.73 | 9.75 | 10.12 | 10.01 |
| Gansu | 62 | 2635.46 | 2559.98 | 2564.19 | 2577.55 | 2582.18 | 13.32 | 12.05 | 12.08 | 12.11 | 12.16 |
| Qinghai | 63 | 557.3 | 563 | 568.17 | 573.17 | 577.79 | 14.51 | 14.94 | 14.43 | 14.30 | 14.16 |
| Ningxia | 64 | 625.2 | 632.96 | 639.45 | 647.19 | 654.19 | 14.38 | 14.14 | 13.65 | 13.26 | 13.12 |
| Xinjiang | 65 | 2158.63 | 2185.11 | 2208.71 | 2232.78 | 2264.3 | 15.99 | 15.99 | 14.99 | 15.32 | 15.84 |
| National |  | 133474 | 134091 | 134735 | 135404 | 136072 | 12.13 | 11.90 | 11.93 | 12.10 | 12.08 |
