# Supplementary figures and images for: Hand, Foot, and Mouth Disease in China: Modeling Epidemic Dynamics of Enterovirus Serotypes and Implications for Vaccination
Source: PLoS Med. 2016 Feb 16;13(2):e1001958. doi: 10.1371/journal.pmed.1001958 (PMC4755668; doi:10.1371/journal.pmed.1001958)

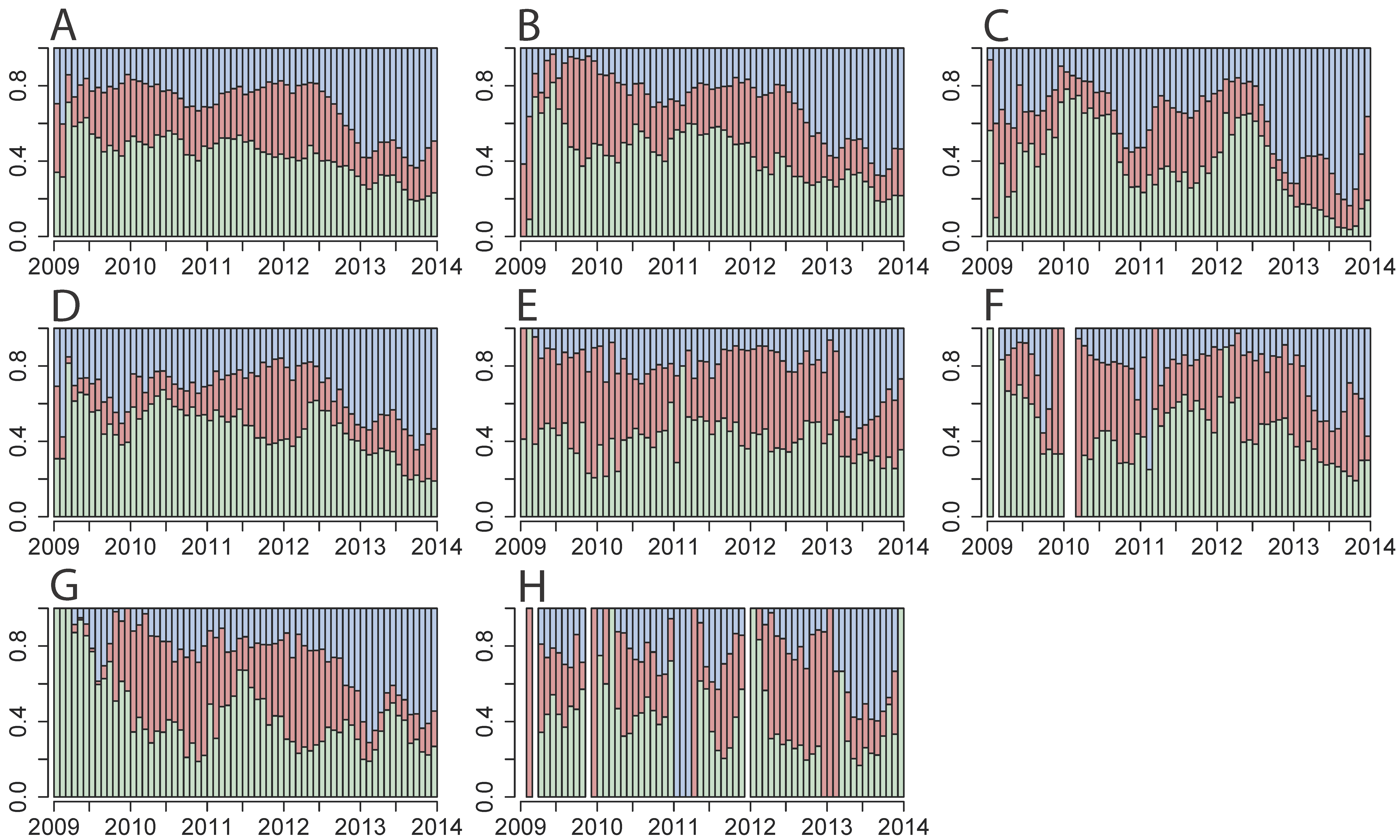

Supplement: S1 Fig — Classified as proportion of EV-A71 (green), CV-A16 (red), or other non-EV-A71 and non-CV-A16 serotypes of enterovirus (blue) (y-axis) between 1 January 2009 and 31 December 2013, every 4 wk (x-axis), on a national scale (A) and by region: east region (B), south region (C), central region (D), north region (E), northwest region (F), southwest region (G), and northeast region (H). (TIFF) [file pmed.1001958.s004.tiff]

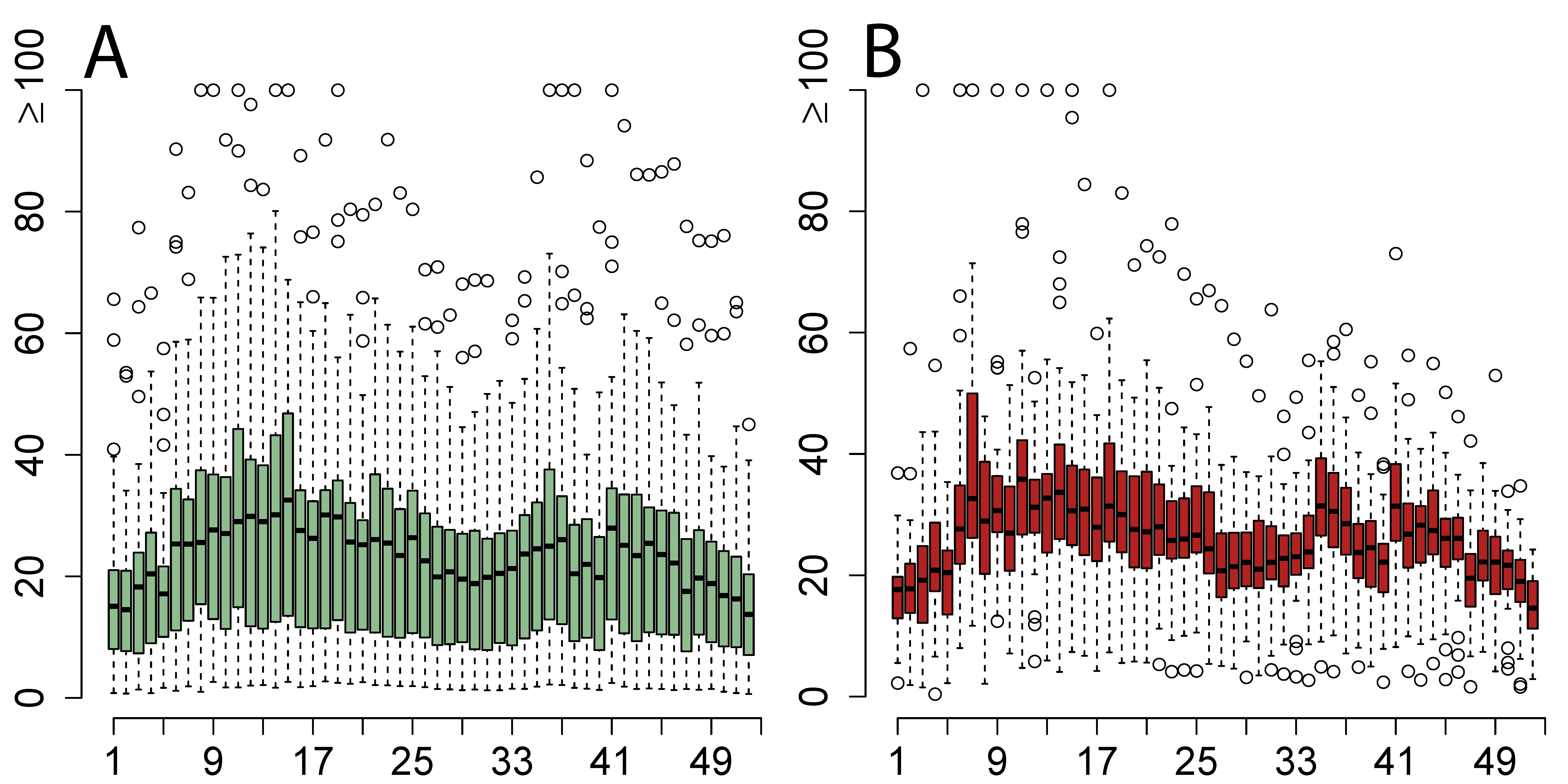

Supplement: S2 Fig — Range in estimated β^s values (y-axis) for each of the 52 wk of the year (x-axis) across all 31 provinces for (A) EV-A71 and (B) CV-A16 in the two-serotype model, using province-specific maximum likelihood estimates of cross-protection. Each data point represents a province. (TIFF) [file pmed.1001958.s005.tiff]

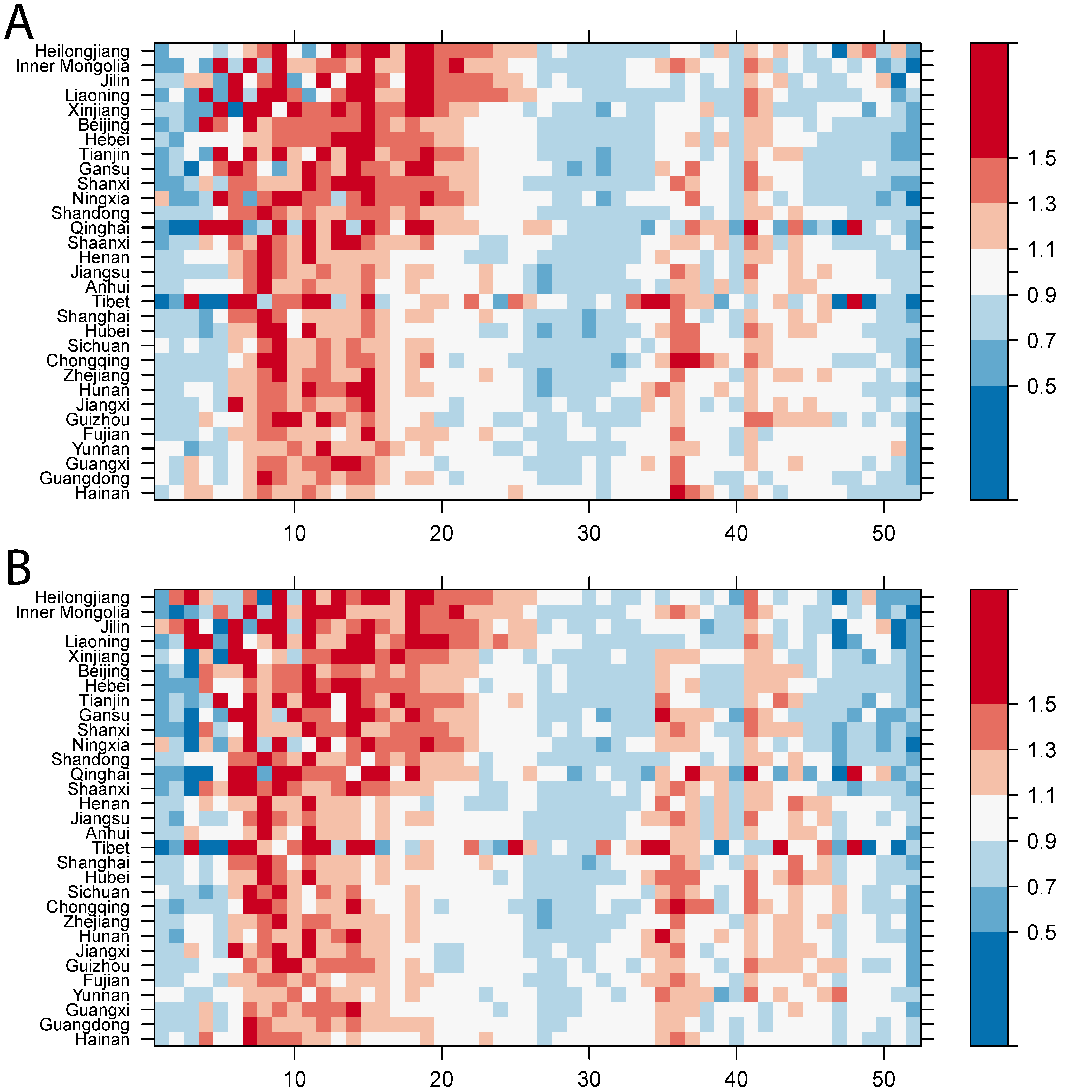

Supplement: S3 Fig — Estimated R E values (y-axis) for each of the 52 wk of the year (x-axis) (each grid cell represents the median value of the 4 y from 2010 to 2013) by province for (A) EV-A71 and (B) CV-A16. Calculated with α = 0.95 and province-specific maximum likelihood estimates of cross-protection, with provinces ordered along the latitudinal gradient (north to south). (TIFF) [file pmed.1001958.s006.tiff]

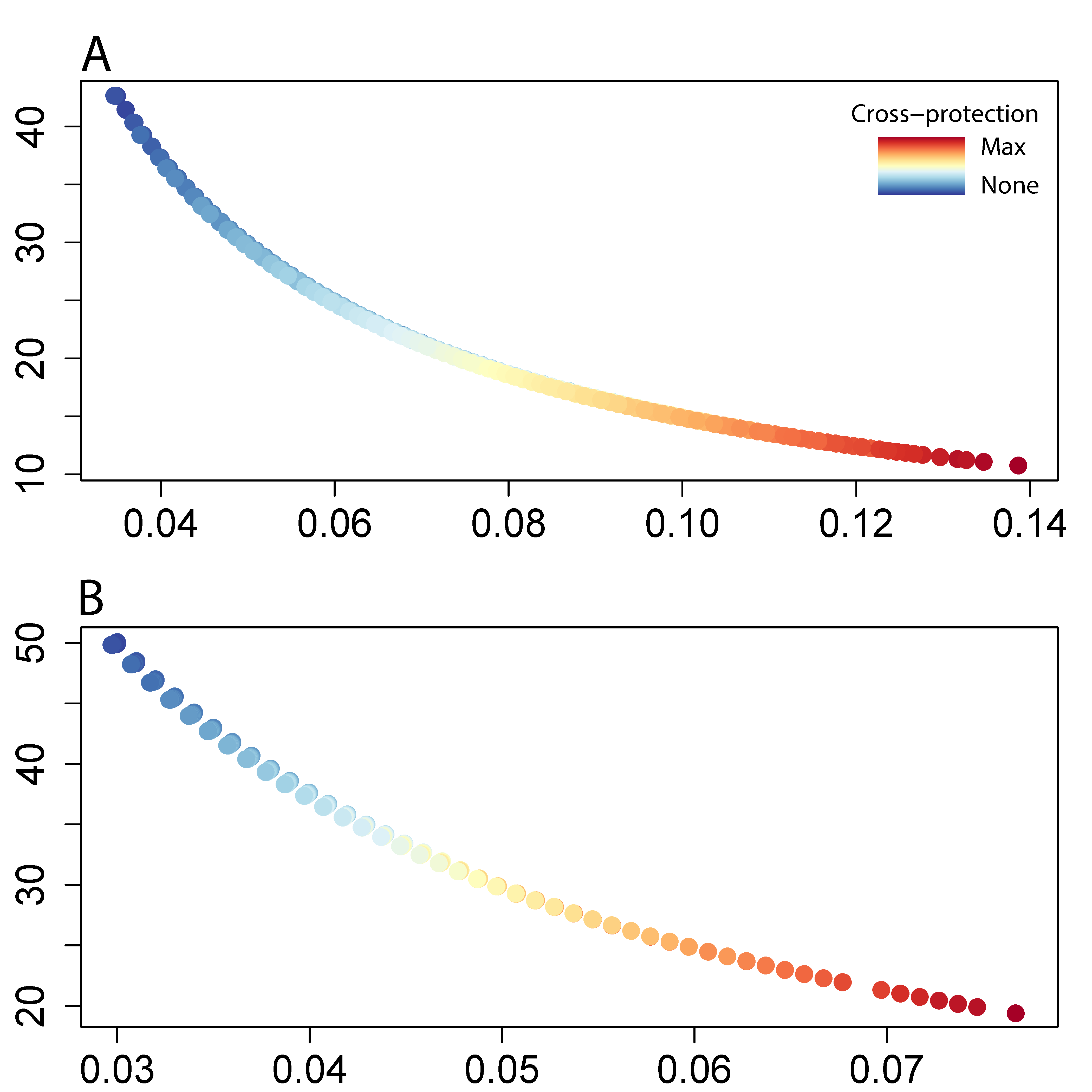

Supplement: S4 Fig — Estimated S¯ (x-axis) against β¯ (y-axis) for (A) EV-A71 and (B) CV-A16 in the two-serotype model for α = 0.95, under varying cross-protection levels (blue–red spectrum, where “None” represents k = 0 wk and δ = 0, and “Max” represents k = 52 wk and δ = 1). (TIFF) [file pmed.1001958.s007.tiff]

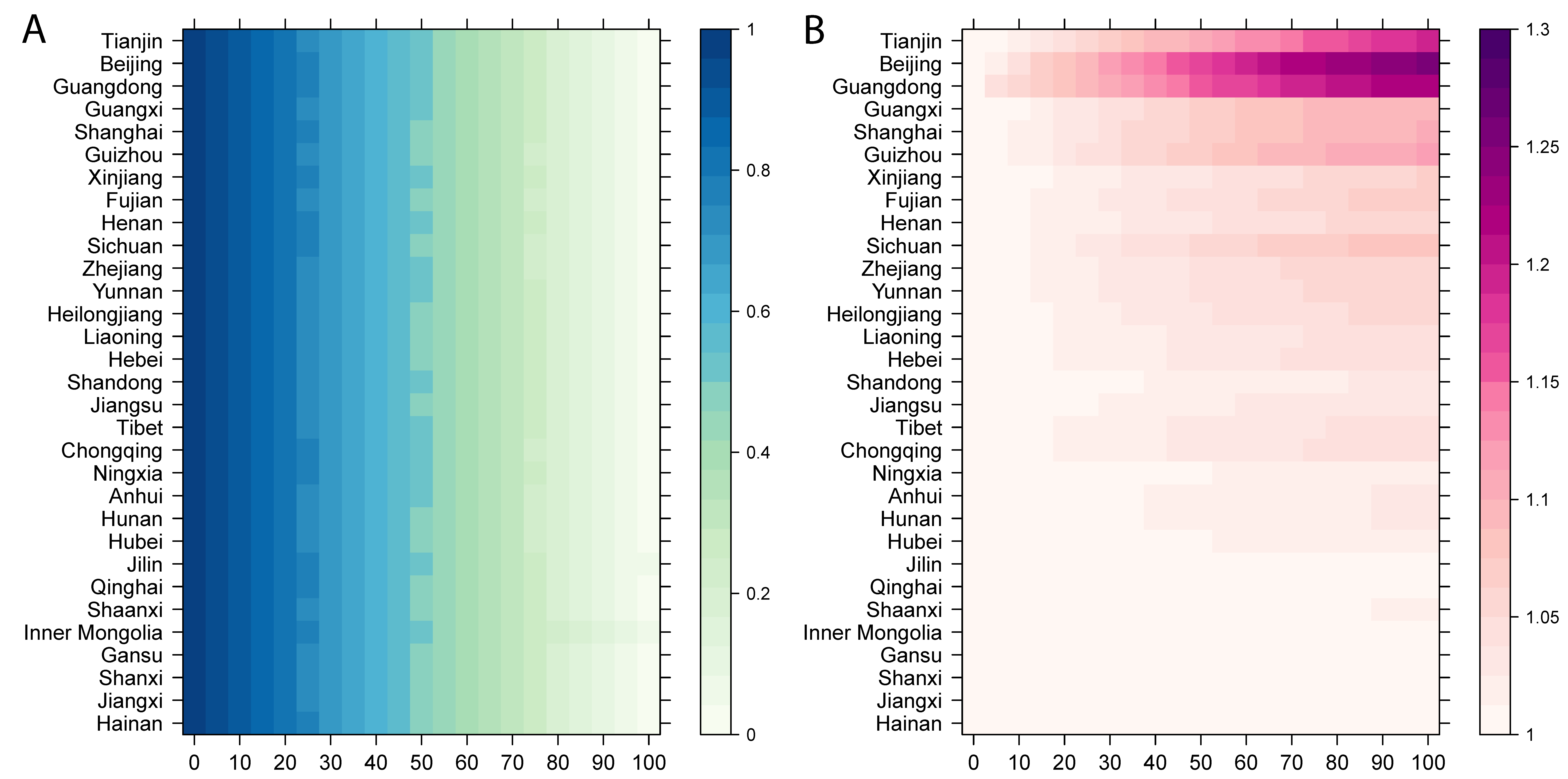

Supplement: S5 Fig — (A) Relative change in yearly incidence of EV-A71 (deterministic simulation) by province (y-axis) 60 y following vaccine initiation compared to pre-vaccination equilibria, ignoring seasonality in βs, as a function of narrow EV-A71 vaccine coverage (x-axis). Calculated with α = 0.95 and province-specific maximum likelihood estimates of cross-protection, with provinces ordered by degree of cross-protection (high to low). (B) Maximum transient increase in yearly incidence of CV-A16 (deterministic simulation) by province (y-axis) following vaccine initiation compared to pre-vaccination equilibria, ignoring seasonality in βs, as a function of narrow EV-A71 vaccine coverage (x-axis). Calculated with α = 0.95 and province-specific maximum likelihood estimates of cross-protection, with provinces ordered by degree of cross-protection (high to low). Note that there is no change in the yearly incidence of CV-A16 after vaccination in provinces where the maximum likelihood estimates of cross-protection are zero. (TIFF) [file pmed.1001958.s008.tiff]

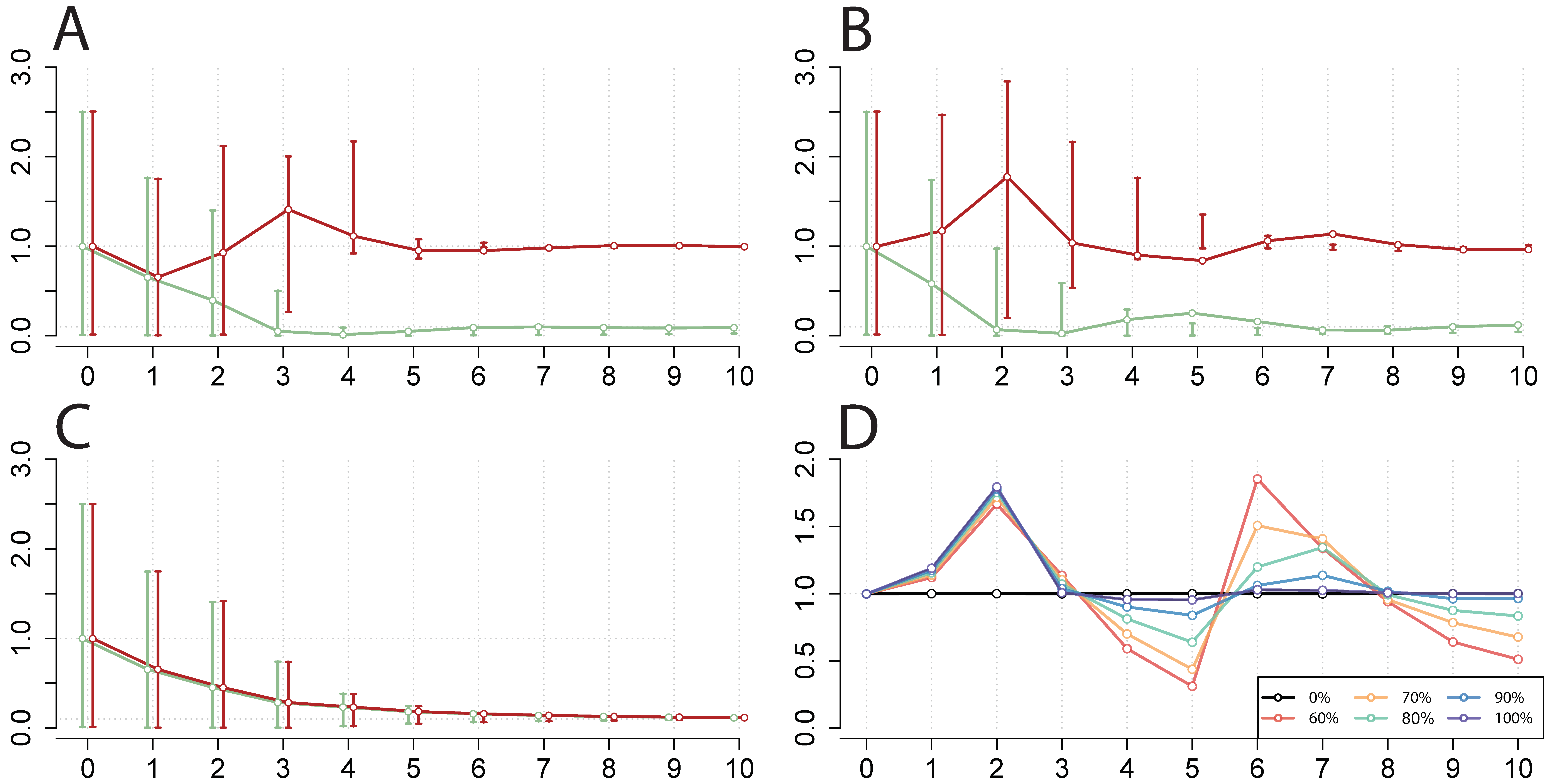

Supplement: S6 Fig — (A–C) Relative change in incidence of EV-A71 (green) and CV-A16 (red) (y-axis) by year (x-axis) for 10 y following vaccine initiation compared to pre-vaccination equilibria (at year 0) in the two-serotype model, ignoring seasonality in βs. The circles indicate the output from the deterministic simulation, with error bars showing the 5th and 95th percentiles of 500 stochastic simulations. Vaccine scenarios explored: (A) broad monovalent EV-A71 vaccine (administered at birth) achieving 90% coverage, (B) narrow monovalent EV-A71 vaccine achieving 90% coverage, and (C) narrow bivalent EV-A71, CV-A16 vaccine achieving 90% coverage. (D) Duration and magnitude of change in CV-A16 yearly incidence compared to pre-vaccination equilibria, as a function of narrow monovalent EV-A71 vaccine coverage (0%, 60%, 70%, 80%, 90%, and 100%). Calculated with α = 0.95, k infection = 52 wk, and δinfection = 1. (TIF) [file pmed.1001958.s009.tif]

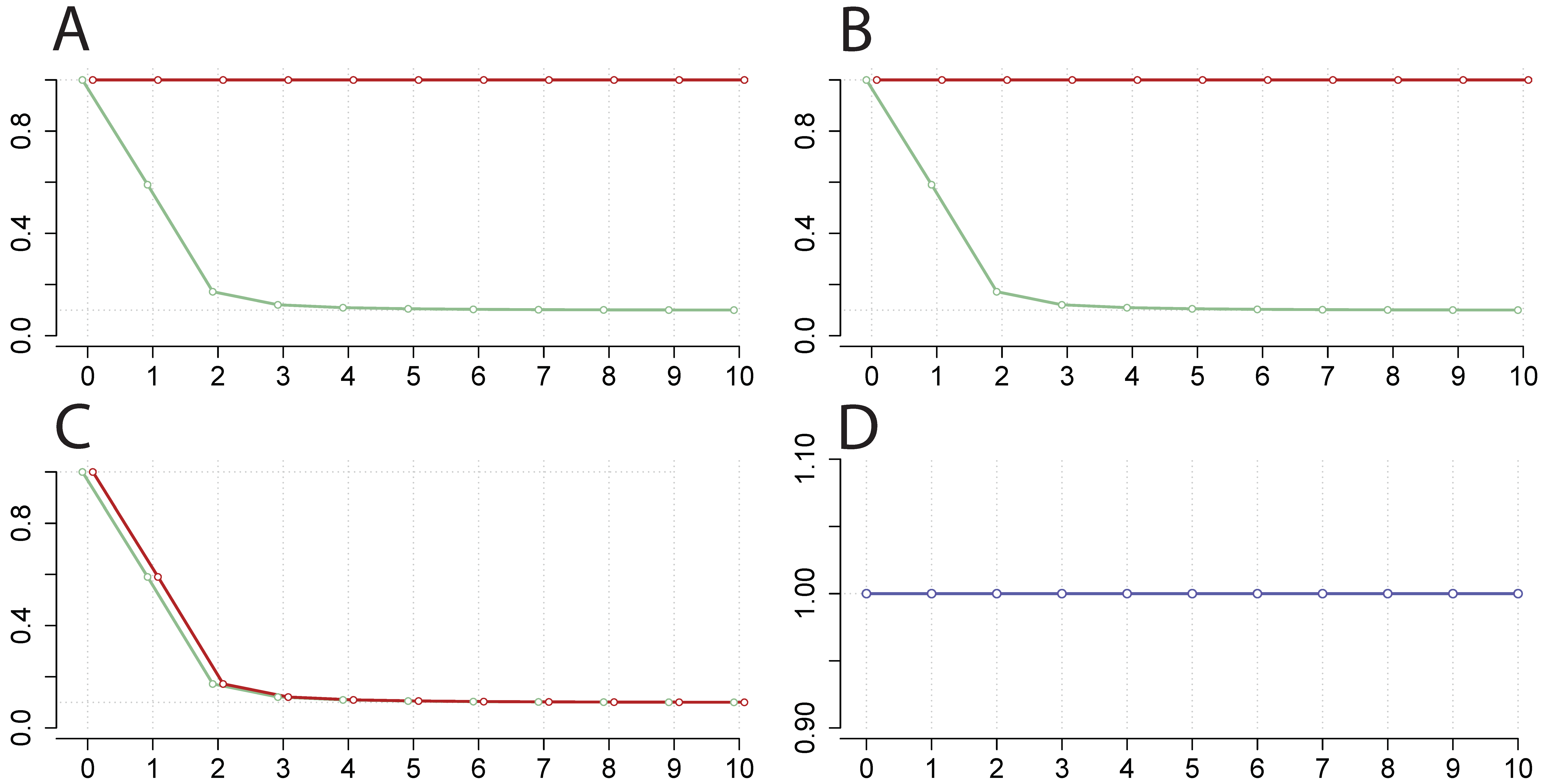

Supplement: S7 Fig — (A–C) Relative change in incidence of EV-A71 (green) and CV-A16 (red) (y-axis) by year (x-axis) for 10 y following vaccine initiation compared to pre-vaccination equilibria (at year 0) in the two-serotype model, ignoring seasonality in βs. The circles indicate the output from the deterministic simulation, with error bars showing the 5th and 95th percentiles of 500 stochastic simulations. Vaccine scenarios explored: (A) broad monovalent EV-A71 vaccine (administered at birth) achieving 90% coverage, (B) narrow monovalent EV-A71 vaccine achieving 90% coverage, and (C) narrow bivalent EV-A71, CV-A16 vaccine achieving 90% coverage. (D) Duration and magnitude of change in CV-A16 yearly incidence compared to pre-vaccination equilibria, as a function of narrow monovalent EV-A71 vaccine coverage (same value for all vaccine coverage levels). Calculated with α = 0.95, k infection = 0 wk, and δinfection = 0. (TIF) [file pmed.1001958.s010.tif]

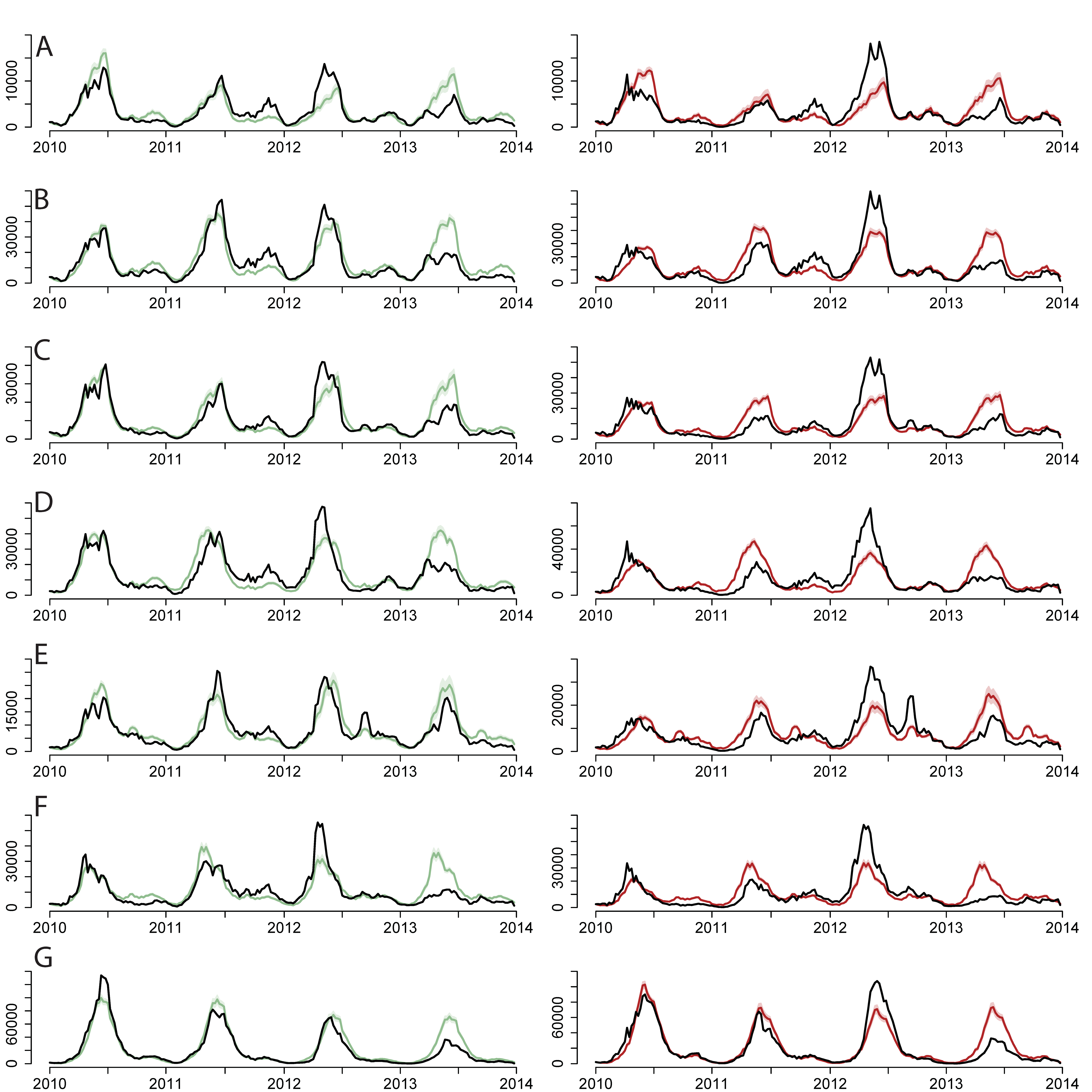

Supplement: S8 Fig — Observed number of cases adjusted for reporting rate (y-axis) by week (years 2010–2013, x-axis) (black line) against predictions from 1,000 stochastic simulations of the entire time series for EV-A71 (green) and CV-A16 (red), showing median value (solid colored line) and 5th and 95th percentiles of the simulations (shaded area). Showing individual provinces comprising the east region: (A) Shanghai, (B) Jiangsu, (C) Zhejiang, (D) Anhui, (E) Fujian, (F) Jiangxi, (G) Shandong. Calculated with α = 0.95 and province-specific maximum likelihood estimates of cross-protection. (TIFF) [file pmed.1001958.s011.tiff]

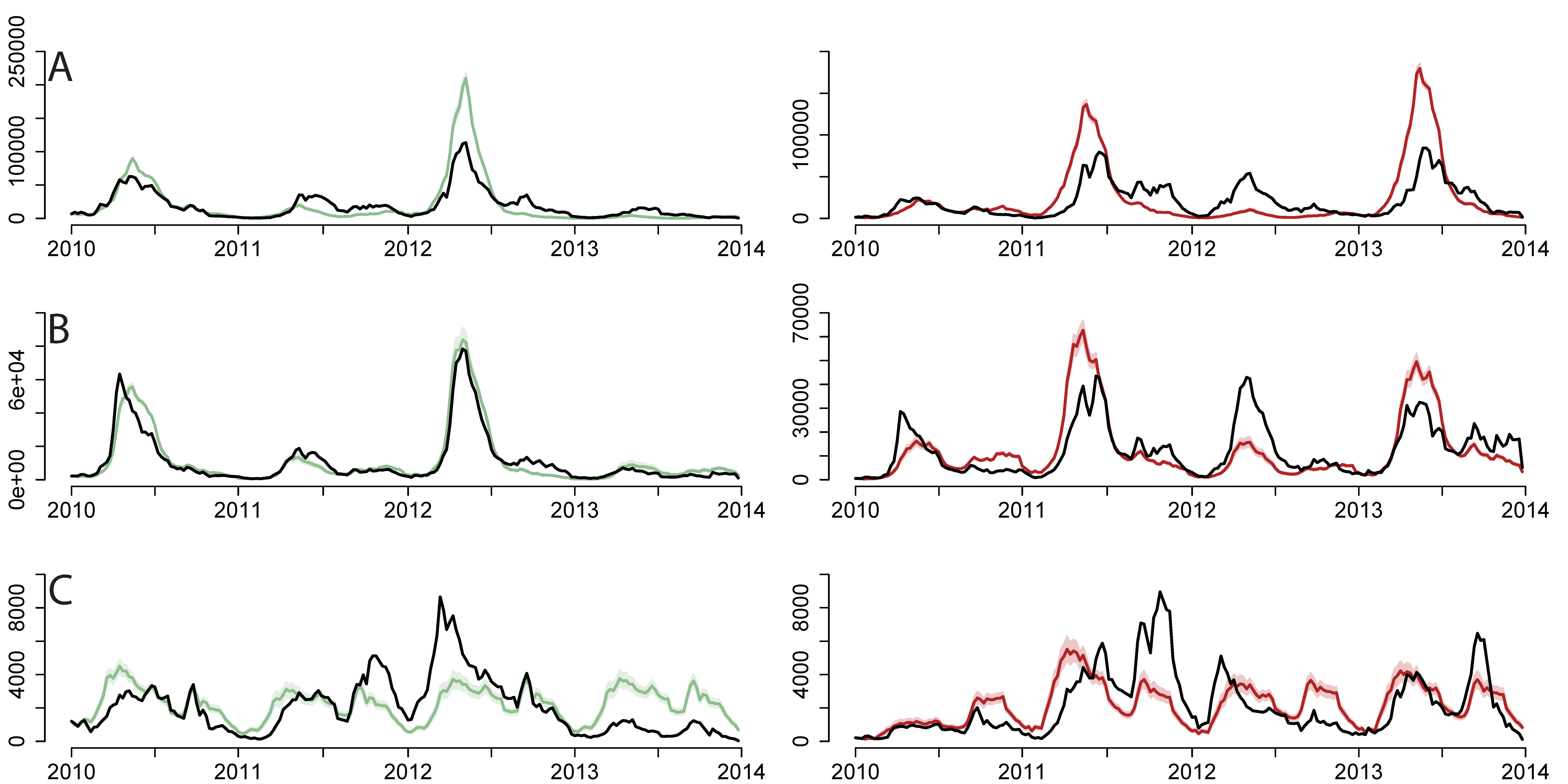

Supplement: S9 Fig — Observed number of cases adjusted for reporting rate (y-axis) by week (years 2010–2013, x-axis) (black line) against predictions from 1,000 stochastic simulations of the entire time series for EV-A71 (green) and CV-A16 (red), showing median value (solid colored line) and 5th and 95th percentiles of the simulations (shaded area). Showing individual provinces comprising the south region: (A) Guangdong, (B) Guangxi, (C) Hainan. Calculated with α = 0.95 and province-specific maximum likelihood estimates of cross-protection. (TIFF) [file pmed.1001958.s012.tiff]

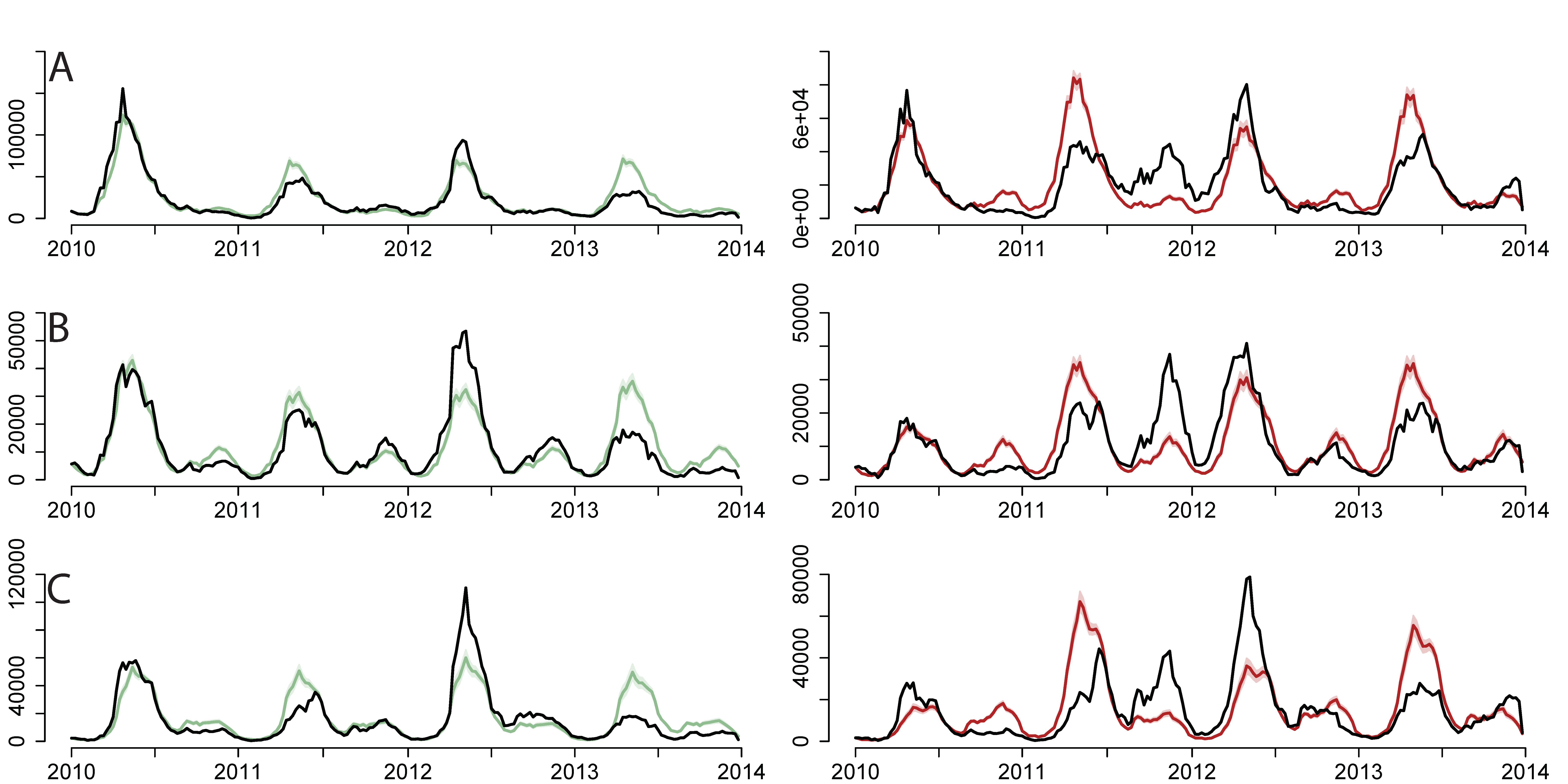

Supplement: S10 Fig — Observed number of cases adjusted for reporting rate (y-axis) by week (years 2010–2013, x-axis) (black line) against predictions from 1,000 stochastic simulations of the entire time series for EV-A71 (green) and CV-A16 (red), showing median value (solid colored line) and 5th and 95th percentiles of the simulations (shaded area). Showing individual provinces comprising the central region: (A) Henan, (B) Hubei, (C) Hunan. Calculated with α = 0.95 and province-specific maximum likelihood estimates of cross-protection. (TIFF) [file pmed.1001958.s013.tiff]

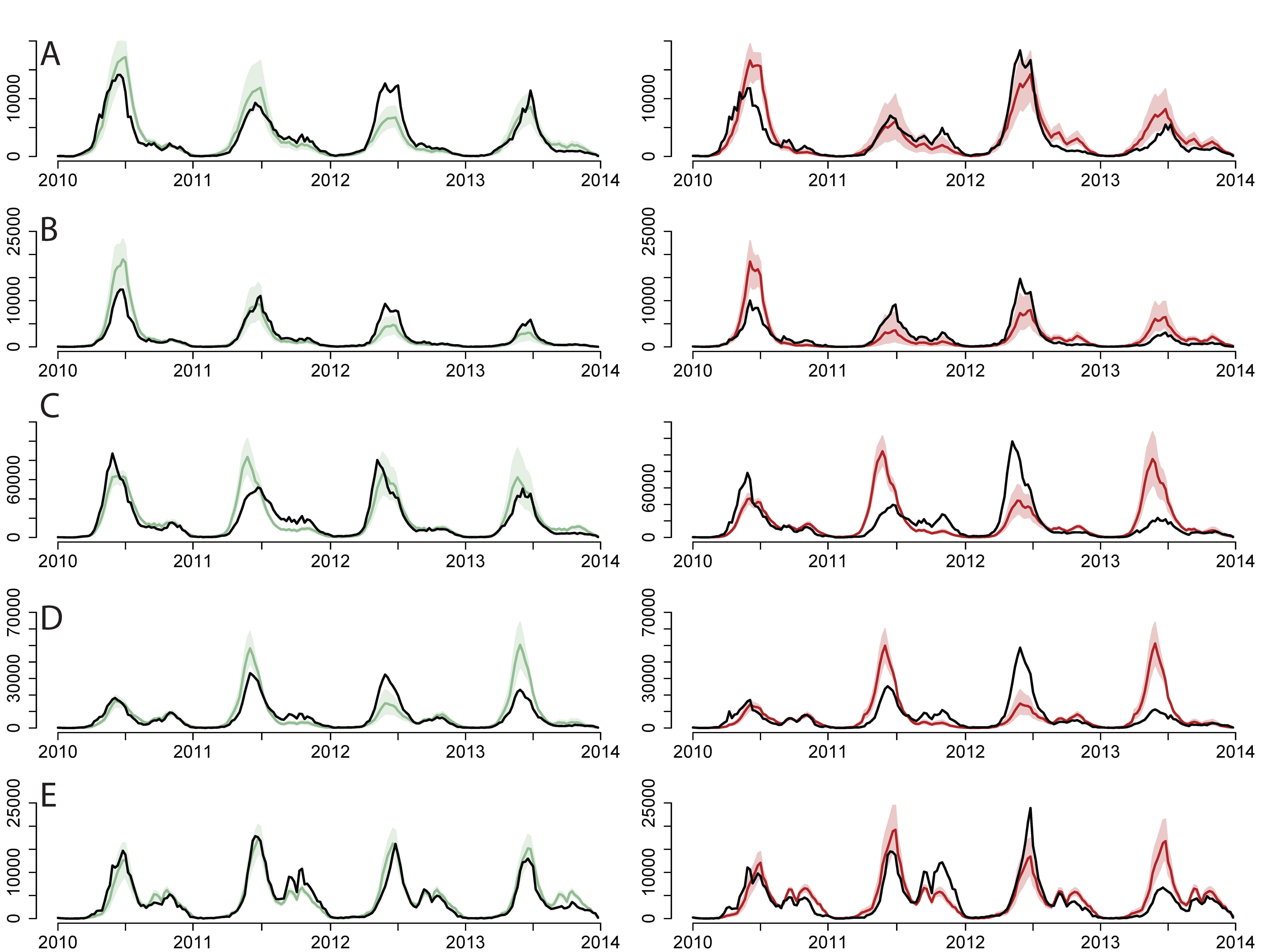

Supplement: S11 Fig — Observed number of cases adjusted for reporting rate (y-axis) by week (years 2010–2013, x-axis) (black line) against predictions from 1,000 stochastic simulations of the entire time series for EV-A71 (green) and CV-A16 (red), showing median value (solid colored line) and 5th and 95th percentiles of the simulations (shaded area). Showing individual provinces comprising the north region: (A) Beijing, (B) Tianjin, (C) Hebei, (D) Shanxi, (E) Inner Mongolia. Calculated with α = 0.95 and province-specific maximum likelihood estimates of cross-protection. (TIFF) [file pmed.1001958.s014.tiff]

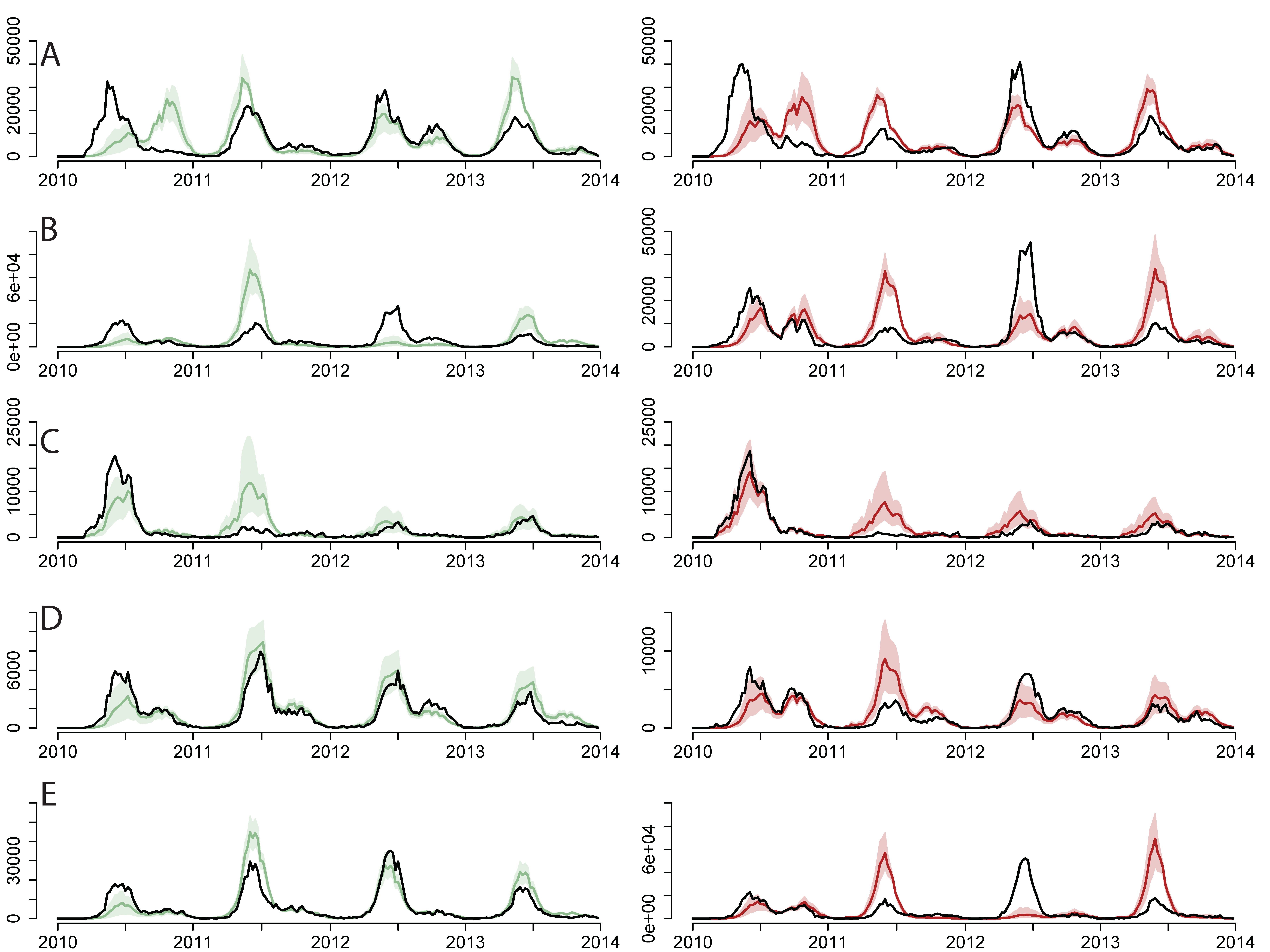

Supplement: S12 Fig — Observed number of cases adjusted for reporting rate (y-axis) by week (years 2010–2013, x-axis) (black line) against predictions from 1,000 stochastic simulations of the entire time series for EV-A71 (green) and CV-A16 (red), showing median value (solid colored line) and 5th and 95th percentiles of the simulations (shaded area). Showing individual provinces comprising the northwest region: (A) Shaanxi, (B) Gansu, (C) Qinghai, (D) Ningxia, (E) Xinjiang. Calculated with α = 0.95 and province-specific maximum likelihood estimates of cross-protection. (TIFF) [file pmed.1001958.s015.tiff]

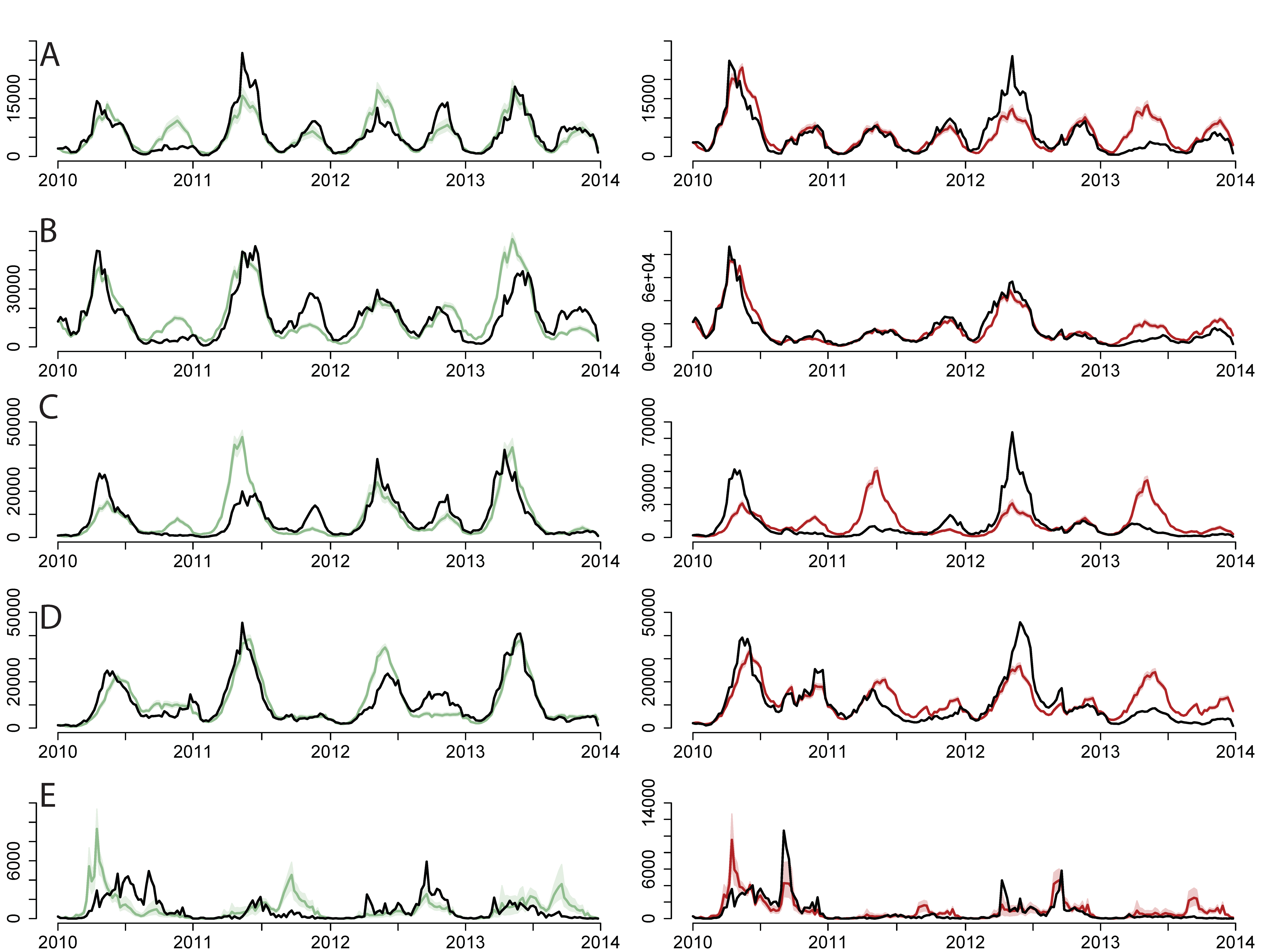

Supplement: S13 Fig — Observed number of cases adjusted for reporting rate (y-axis) by week (years 2010–2013, x-axis) (black line) against predictions from 1,000 stochastic simulations of the entire time series for EV-A71 (green) and CV-A16 (red), showing median value (solid colored line) and 5th and 95th percentiles of the simulations (shaded area). Showing individual provinces comprising the southwest region: (A) Chongqing, (B) Sichuan, (C) Guizhou, (D) Yunnan, (E) Tibet. Calculated with α = 0.95 and province-specific maximum likelihood estimates of cross-protection. (TIFF) [file pmed.1001958.s016.tiff]

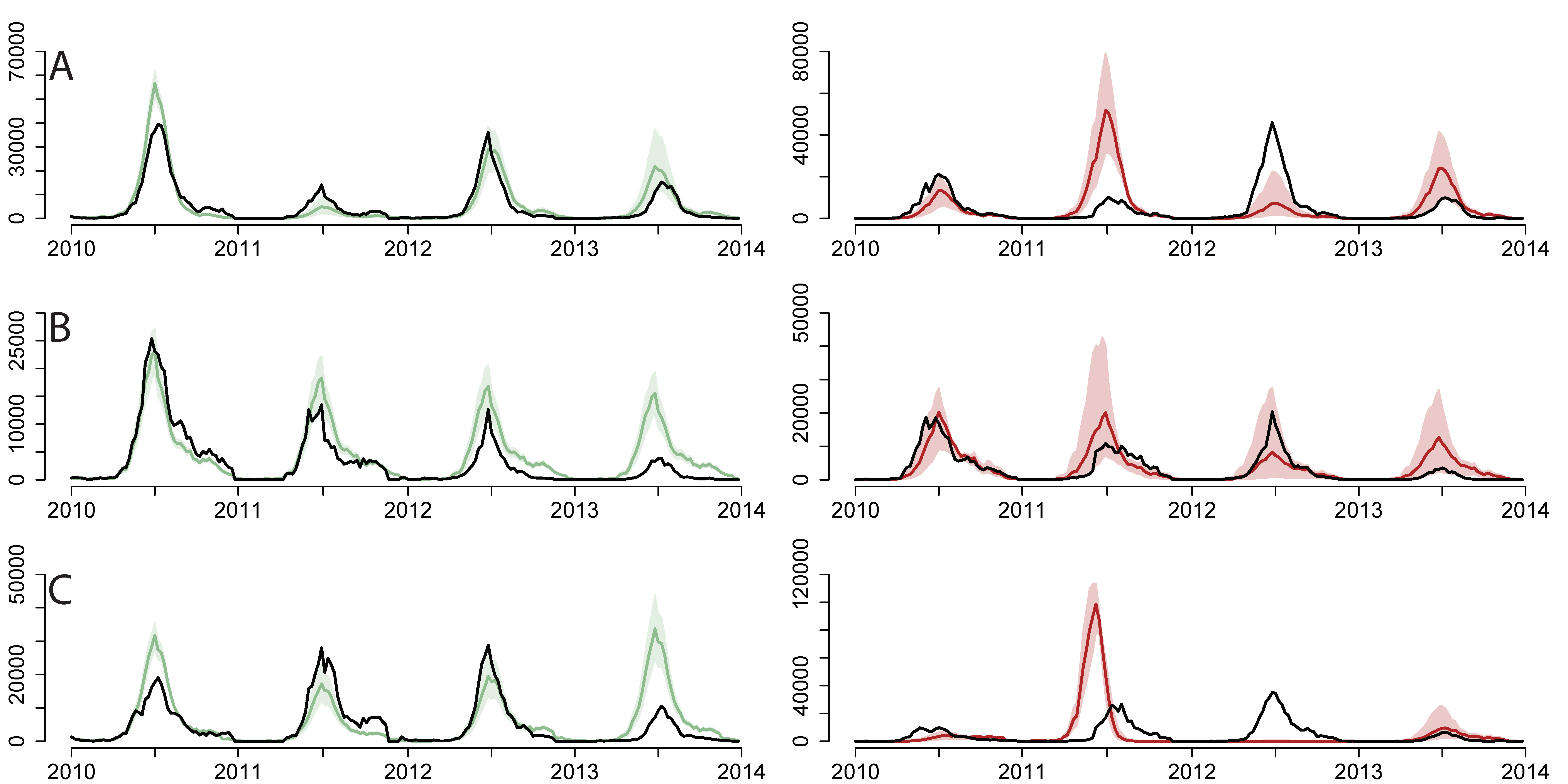

Supplement: S14 Fig — Observed number of cases adjusted for reporting rate (y-axis) by week (years 2010–2013, x-axis) (black line) against predictions from 1,000 stochastic simulations of the entire time series for EV-A71 (green) and CV-A16 (red), showing median value (solid colored line) and 5th and 95th percentiles of the simulations (shaded area). Showing individual provinces comprising the northeast region: (A) Liaoning, (B) Jilin, (C) Heilongjiang. Calculated with α = 0.95 and province-specific maximum likelihood estimates of cross-protection. (TIFF) [file pmed.1001958.s017.tiff]

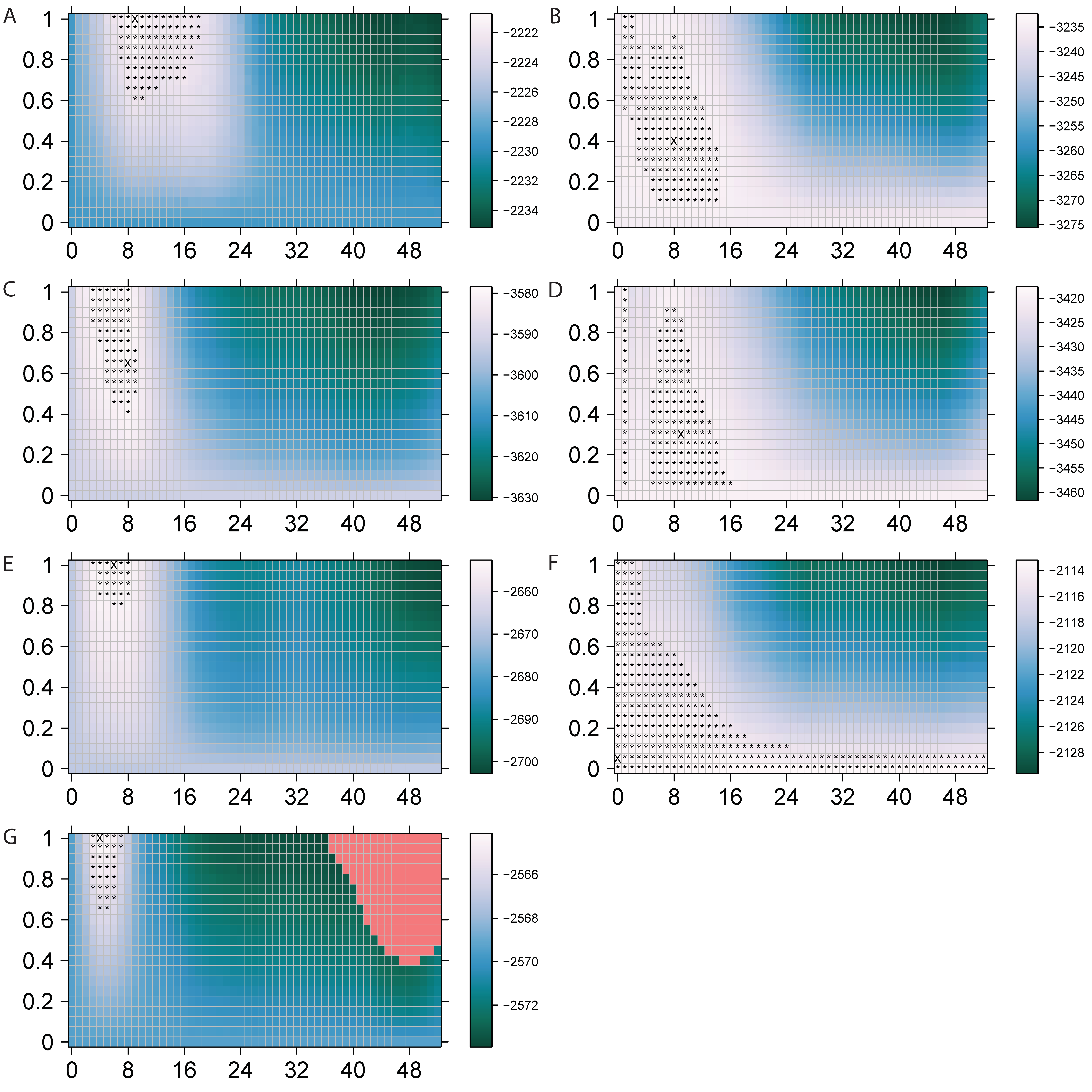

Supplement: S15 Fig — Estimated log-likelihood values over a range of the cross-protection parameters k, from 0 to 52 (in weeks; x-axis), and δ, from 0 to 1 (as proportion; y-axis), in the two-serotype model. Calculated with α = 0.95, showing the maximum likelihood estimate (grid cell with “X”) and 95% confidence region (grid cells with asterisks) in individual provinces comprising the east region: (A) Shanghai, (B) Jiangsu, (C) Zhejiang, (D) Anhui, (E) Fujian, (F) Jiangxi, (G) Shandong. Grid cells highlighted pink are where S¯ for either EV-A71 or CV-A16 is estimated to be 100%. (TIFF) [file pmed.1001958.s018.tiff]

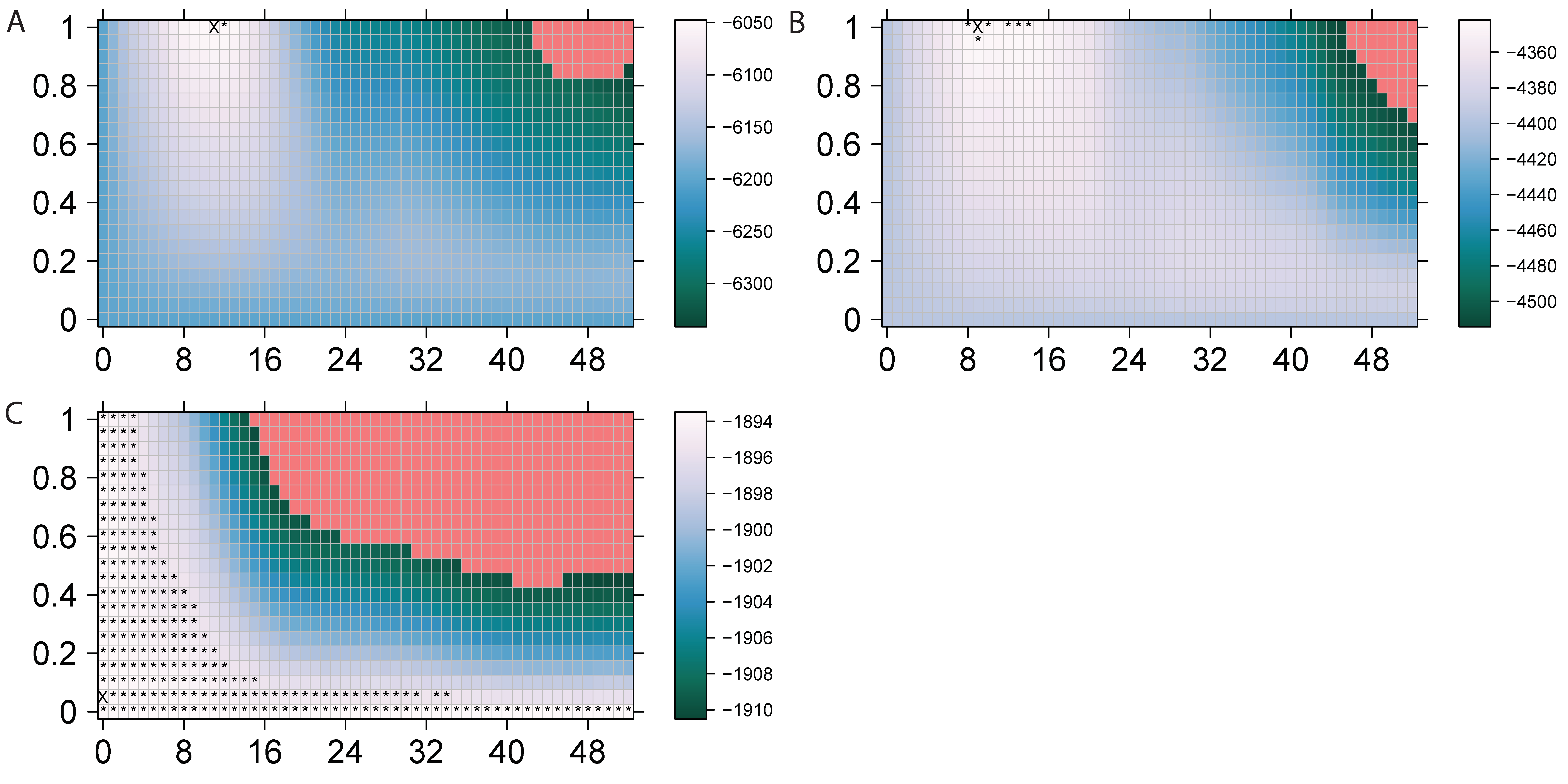

Supplement: S16 Fig — Estimated log-likelihood values over a range of the cross-protection parameters k, from 0 to 52 (in weeks; x-axis), and δ, from 0 to 1 (as proportion; y-axis), in the two-serotype model. Calculated with α = 0.95, showing the maximum likelihood estimate (grid cell with “X”) and 95% confidence region (grid cells with asterisks) in individual provinces comprising the south region: (A) Guangdong, (B) Guangxi, (C) Hainan. Grid cells highlighted pink are where S¯ for either EV-A71 or CV-A16 is estimated to be 100%. (TIFF) [file pmed.1001958.s019.tiff]

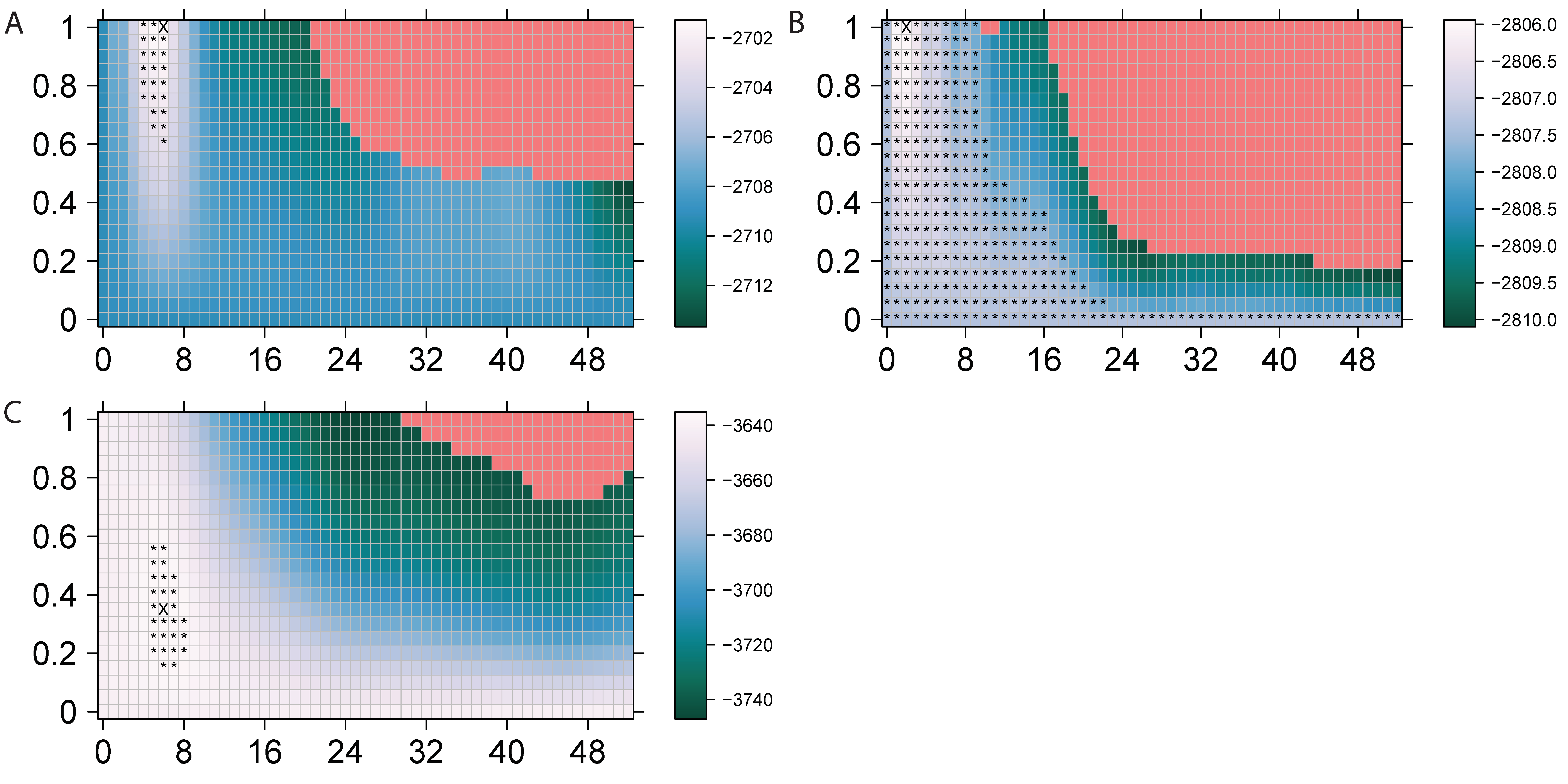

Supplement: S17 Fig — Estimated log-likelihood values over a range of the cross-protection parameters k, from 0 to 52 (in weeks; x-axis), and δ, from 0 to 1 (as proportion; y-axis), in the two-serotype model. Calculated with α = 0.95, showing the maximum likelihood estimate (grid cell with “X”) and 95% confidence region (grid cells with asterisks) in individual provinces comprising the central region: (A) Henan, (B) Hubei, (C) Hunan. Grid cells highlighted pink are where S¯ for either EV-A71 or CV-A16 is estimated to be 100%. (TIFF) [file pmed.1001958.s020.tiff]

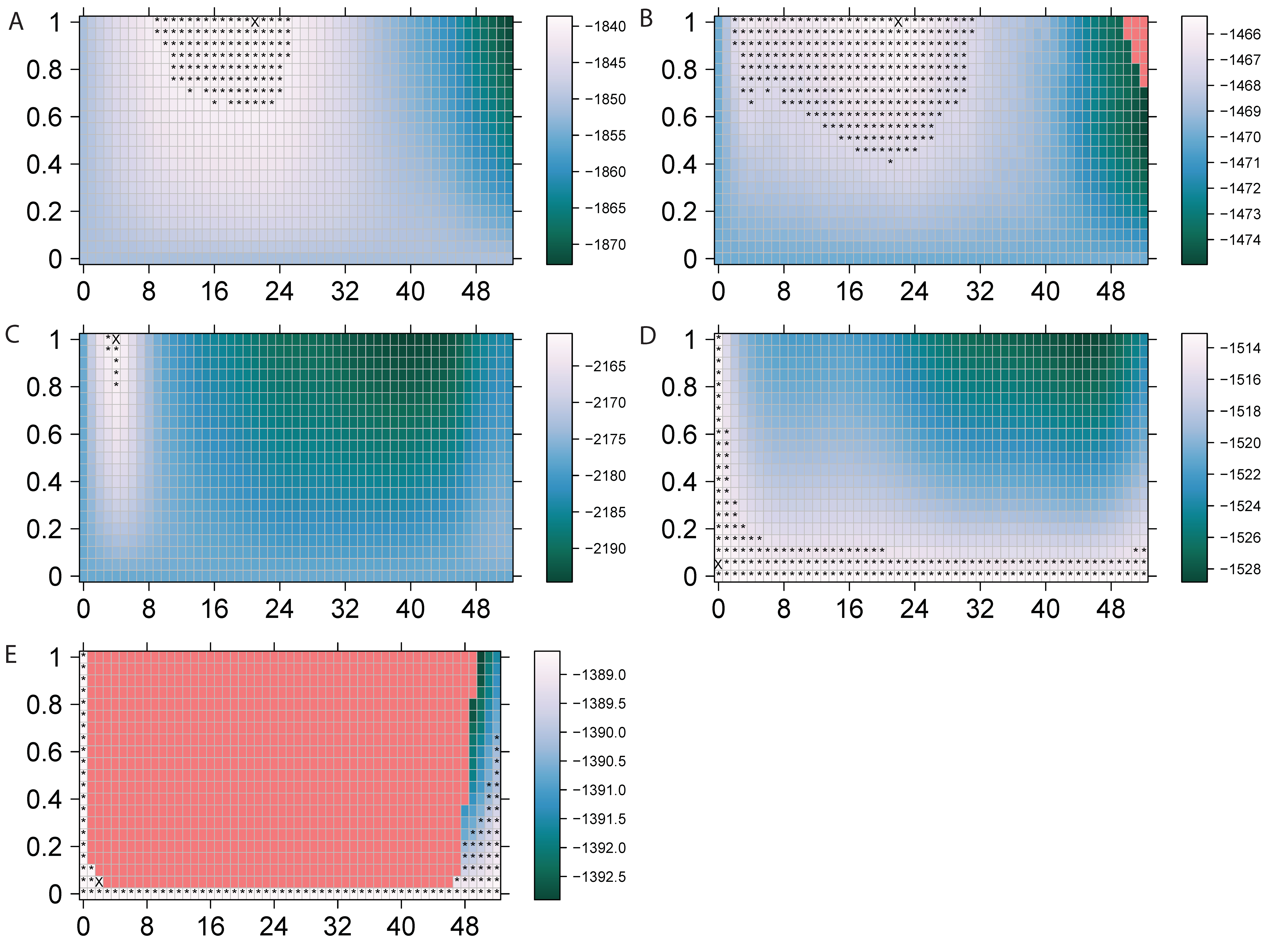

Supplement: S18 Fig — Estimated log-likelihood values over a range of the cross-protection parameters k, from 0 to 52 (in weeks; x-axis), and δ, from 0 to 1 (as proportion; y-axis), in the two-serotype model. Calculated with α = 0.95, showing the maximum likelihood estimate (grid cell with “X”) and 95% confidence region (grid cells with asterisks) in individual provinces comprising the north region: (A) Beijing, (B) Tianjin, (C) Hebei, (D) Shanxi, (E) Inner Mongolia. Grid cells highlighted pink are where S¯ for either EV-A71 or CV-A16 is estimated to be 100%. (TIFF) [file pmed.1001958.s021.tiff]

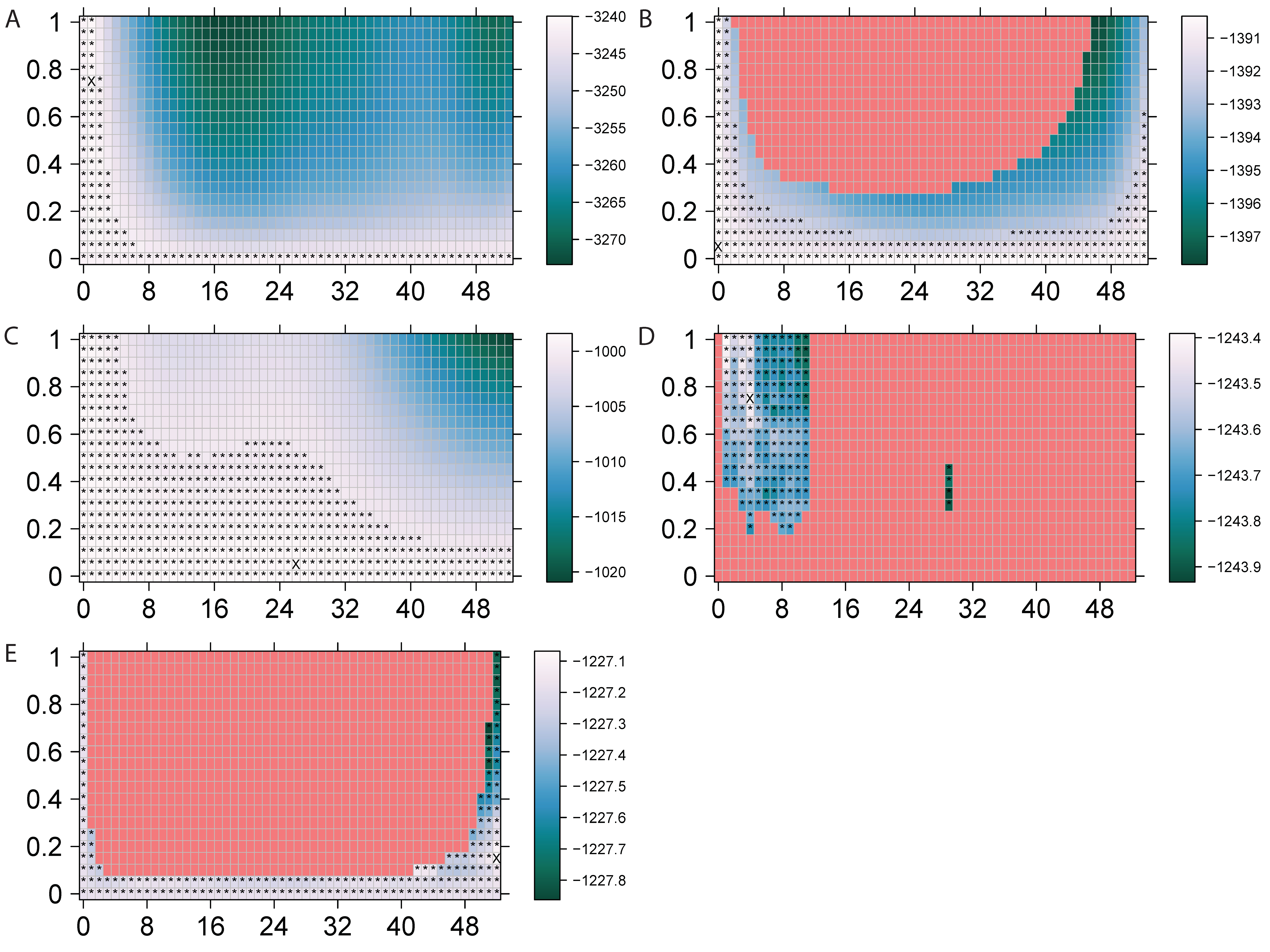

Supplement: S19 Fig — Estimated log-likelihood values over a range of the cross-protection parameters k, from 0 to 52 (in weeks; x-axis), and δ, from 0 to 1 (as proportion; y-axis), in the two-serotype model. Calculated with α = 0.95, showing the maximum likelihood estimate (grid cell with “X”) and 95% confidence region (grid cells with asterisks) in individual provinces comprising the northwest region: (A) Shaanxi, (B) Gansu, (C) Qinghai, (D) Ningxia, (E) Xinjiang. Grid cells highlighted pink are where S¯ for either EV-A71 or CV-A16 is estimated to be 100%. (TIFF) [file pmed.1001958.s022.tiff]

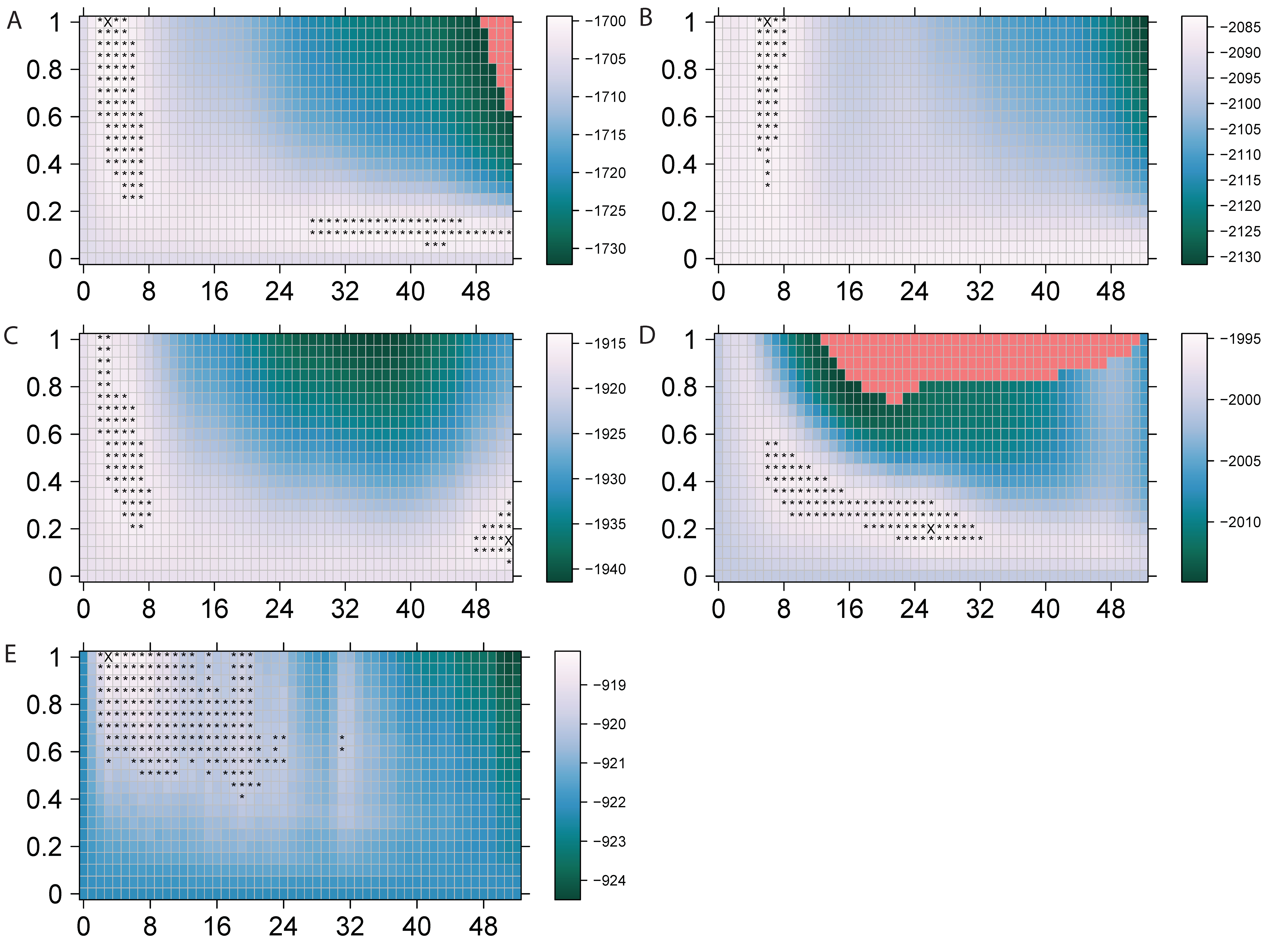

Supplement: S20 Fig — Estimated log-likelihood values over a range of the cross-protection parameters k, from 0 to 52 (in weeks; x-axis), and δ, from 0 to 1 (as proportion; y-axis), in the two-serotype model. Calculated with α = 0.95, showing the maximum likelihood estimate (grid cell with “X”) and 95% confidence region (grid cells with asterisks) in individual provinces comprising the southwest region: (A) Chongqing, (B) Sichuan, (C) Guizhou, (D) Yunnan, (E) Tibet. Grid cells highlighted pink are where S¯ for either EV-A71 or CV-A16 is estimated to be 100%. (TIFF) [file pmed.1001958.s023.tiff]

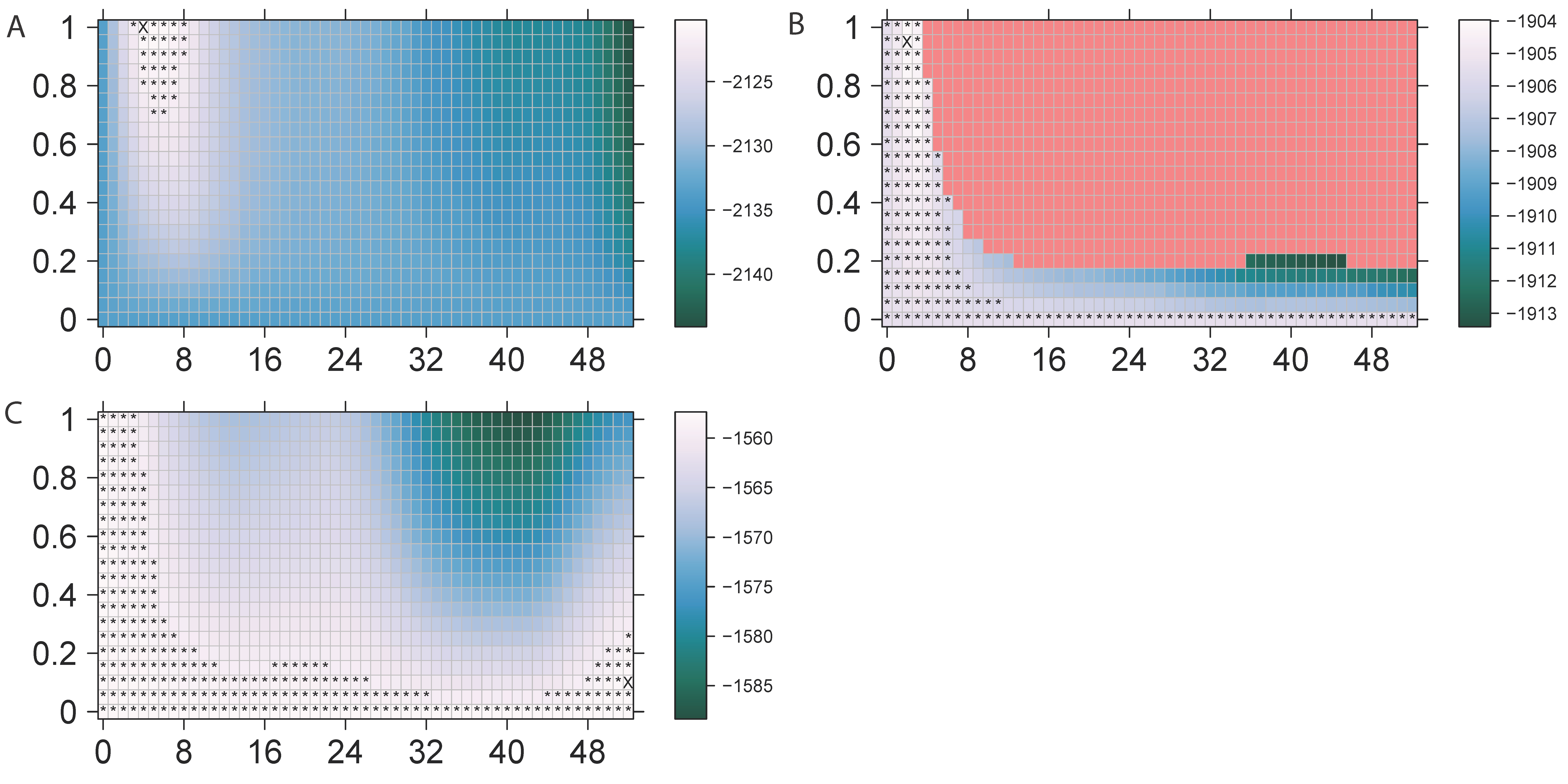

Supplement: S21 Fig — Estimated log-likelihood values over a range of the cross-protection parameters k, from 0 to 52 (in weeks; x-axis), and δ, from 0 to 1 (as proportion; y-axis), in the two-serotype model. Calculated with α = 0.95, showing the maximum likelihood estimate (grid cell with “X”) and 95% confidence region (grid cells with asterisks) in individual provinces comprising the northeast region: (A) Liaoning, (B) Jilin, (C) Heilongjiang. Grid cells highlighted pink are where S¯ for either EV-A71 or CV-A16 is estimated to be 100%. (TIFF) [file pmed.1001958.s024.tiff]

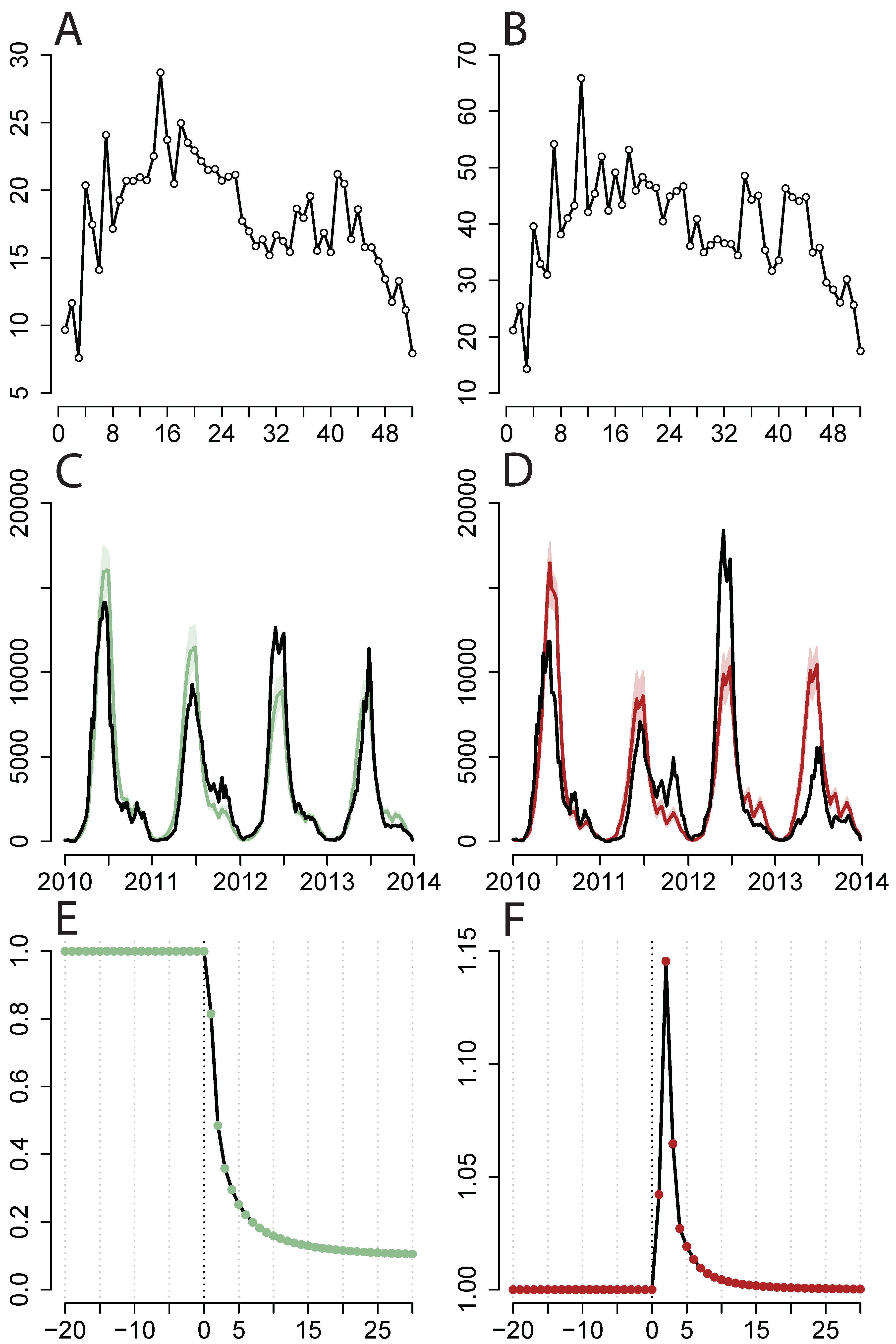

Supplement: S22 Fig — (A and B) Estimated β^s (y-axis) for (A) EV-A71 and (B) CV-A16 by week (x-axis). (C and D) Observed number of cases adjusted for reporting rate (y-axis) from 2010 to 2013 (black line) by week (x-axis) against predictions from 1,000 stochastic simulations of the entire time series for (C) EV-A71 and (D) CV-A16, showing median value (solid colored line) and 5th and 95th percentiles of the simulations (shaded area). (E and F) Output from deterministic simulation of incidence (y-axis) of (E) EV-A71 and (F) CV-A16 by year (x-axis) for 20 y before to 30 y following vaccine initiation (dotted black line, at year 0), normalized by serotype-specific yearly incidence in year −20 and ignoring seasonality in βs. Vaccination assumed to be narrow monovalent EV-A71 vaccine (administered at birth) achieving 90% coverage. S¯ for EV-A71 = 0.112 and S¯ for CV-A16 = 0.052. Calculated with province-specific maximum likelihood estimates of cross-protection (k = 21 wk and δ = 1). (TIFF) [file pmed.1001958.s025.tiff]

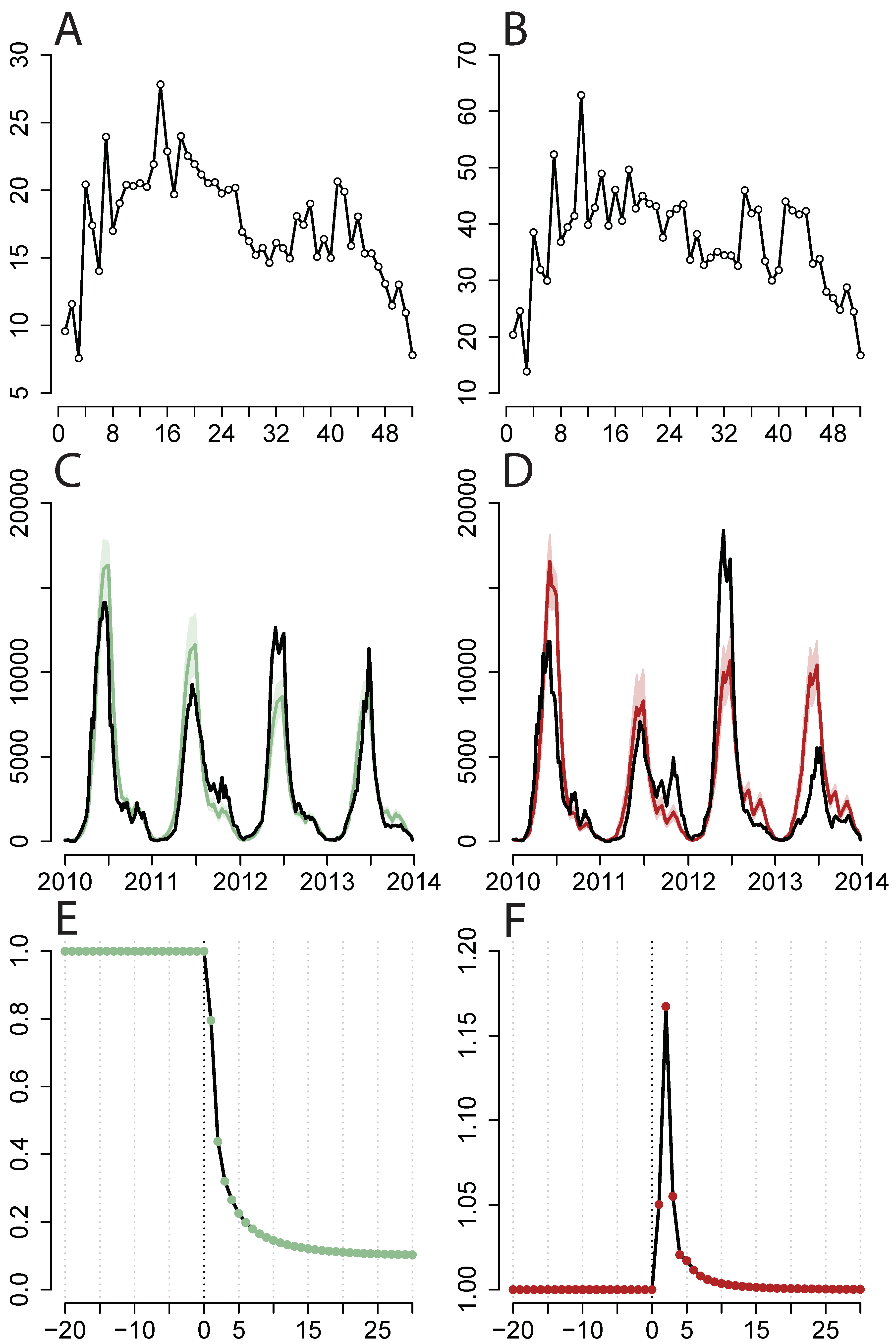

Supplement: S23 Fig — (A and B) Estimated β^s (y-axis) for (A) EV-A71 and (B) CV-A16 by week (x-axis). (C and D) Observed number of cases adjusted for reporting rate (y-axis) from 2010 to 2013 (black line) by week (x-axis) against predictions from 1,000 stochastic simulations of the entire time series for (C) EV-A71 and (D) CV-A16, showing median value (solid colored line) and 5th and 95th percentiles of the simulations (shaded area). (E and F) Output from deterministic simulation of incidence (y-axis) of (E) EV-A71 and (F) CV-A16 by year (x-axis) for 20 y before to 30 y following vaccine initiation (dotted black line, at year 0), normalized by serotype-specific yearly incidence in year −20 and ignoring seasonality in βs. Vaccination assumed to be narrow monovalent EV-A71 vaccine (administered at birth) achieving 90% coverage. S¯ for EV-A71 = 0.107 and S¯ for CV-A16 = 0.051. Calculated with province-specific maximum likelihood estimates of cross-protection (k = 21 wk and δ = 1). (TIFF) [file pmed.1001958.s026.tiff]

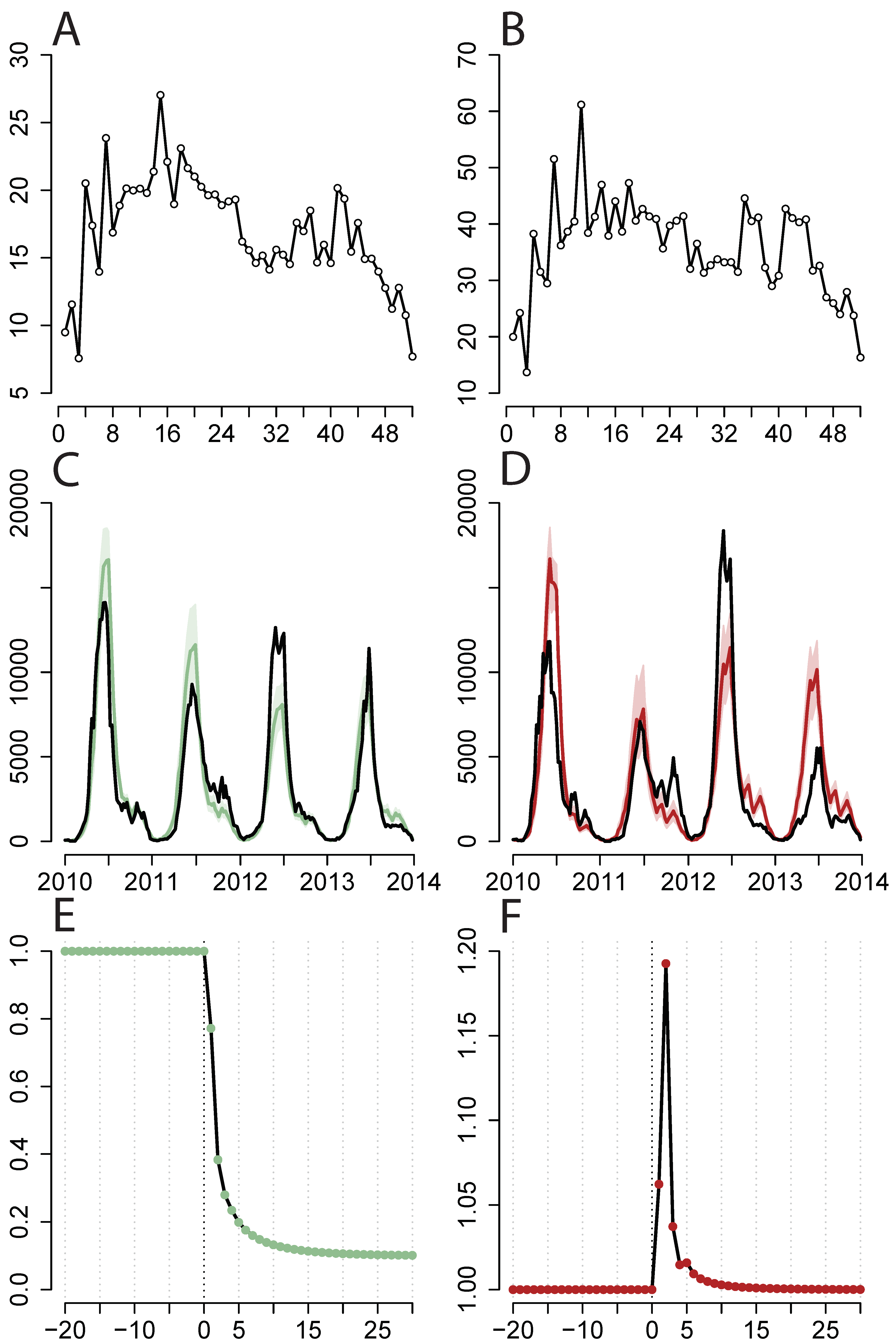

Supplement: S24 Fig — (A and B) Estimated β^s (y-axis) for (A) EV-A71 and (B) CV-A16 by week (x-axis). (C and D) Observed number of cases adjusted for reporting rate (y-axis) from 2010 to 2013 (black line) by week (x-axis) against predictions from 1,000 stochastic simulations of the entire time series for (C) EV-A71 and (D) CV-A16, showing median value (solid colored line) and 5th and 95th percentiles of the simulations (shaded area). (E and F) Output from deterministic simulation of incidence (y-axis) of (E) EV-A71 and (F) CV-A16 by year (x-axis) for 20 y before to 30 y following vaccine initiation (dotted black line, at year 0), normalized by serotype-specific yearly incidence in year −20 and ignoring seasonality in βs. Vaccination assumed to be narrow monovalent EV-A71 vaccine (administered at birth) achieving 90% coverage. S¯ for EV-A71 = 0.102 and S¯ for CV-A16 = 0.049. Calculated with province-specific maximum likelihood estimates of cross-protection (k = 21 wk and δ = 1). (TIFF) [file pmed.1001958.s027.tiff]

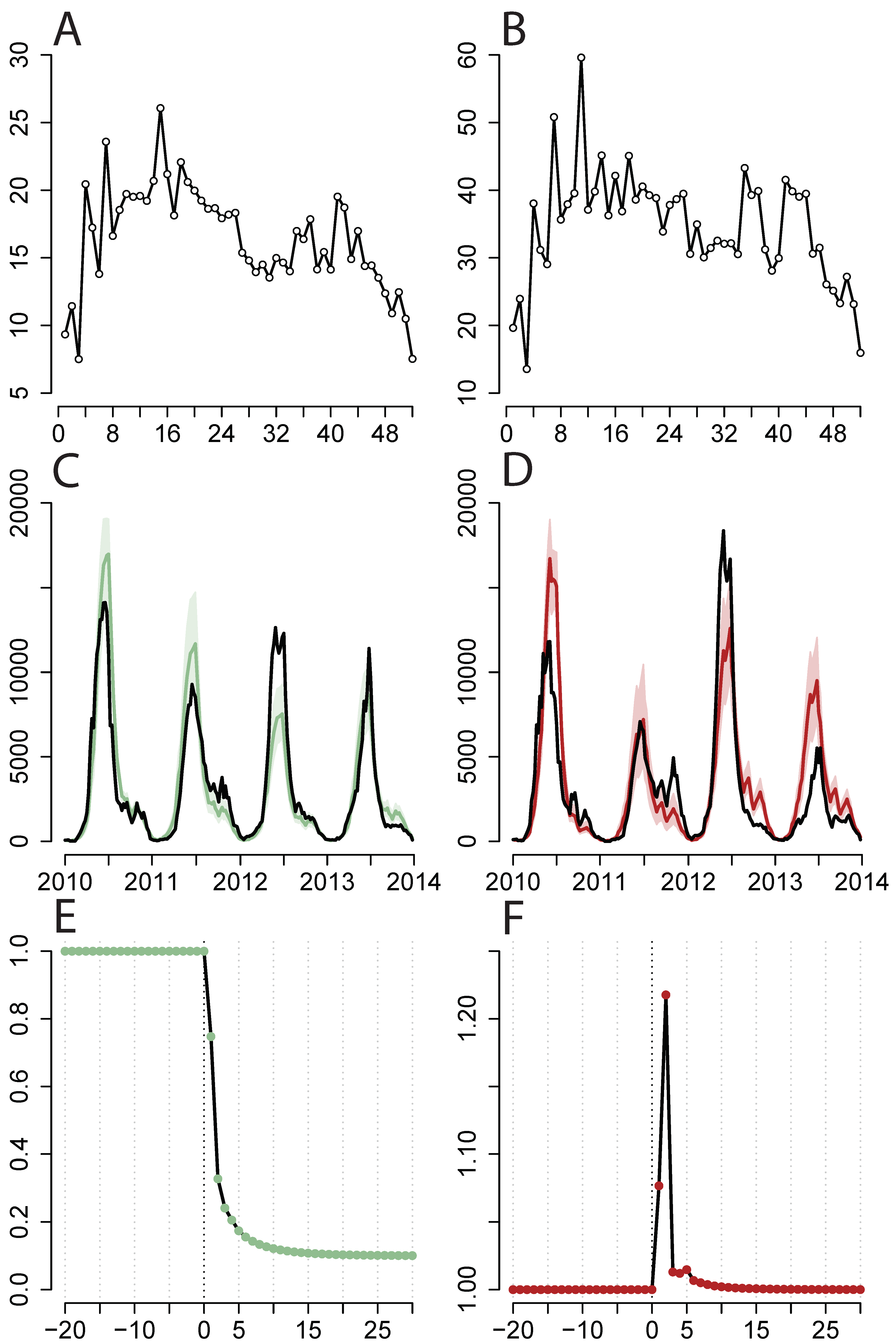

Supplement: S25 Fig — (A and B) Estimated β^s (y-axis) for (A) EV-A71 and (B) CV-A16 by week (x-axis). (C and D) Observed number of cases adjusted for reporting rate (y-axis) from 2010 to 2013 (black line) by week (x-axis) against predictions from 1,000 stochastic simulations of the entire time series for (C) EV-A71 and (D) CV-A16, showing median value (solid colored line) and 5th and 95th percentiles of the simulations (shaded area). (E and F) Output from deterministic simulation of incidence (y-axis) of (E) EV-A71 and (F) CV-A16 by year (x-axis) for 20 y before to 30 y following vaccine initiation (dotted black line, at year 0), normalized by serotype-specific yearly incidence in year −20 and ignoring seasonality in βs. Vaccination assumed to be narrow monovalent EV-A71 vaccine (administered at birth) achieving 90% coverage. S¯ for EV-A71 = 0.098 and S¯ for CV-A16 = 0.047. Calculated with province-specific maximum likelihood estimates of cross-protection (k = 21 wk and δ = 1). (TIFF) [file pmed.1001958.s028.tiff]

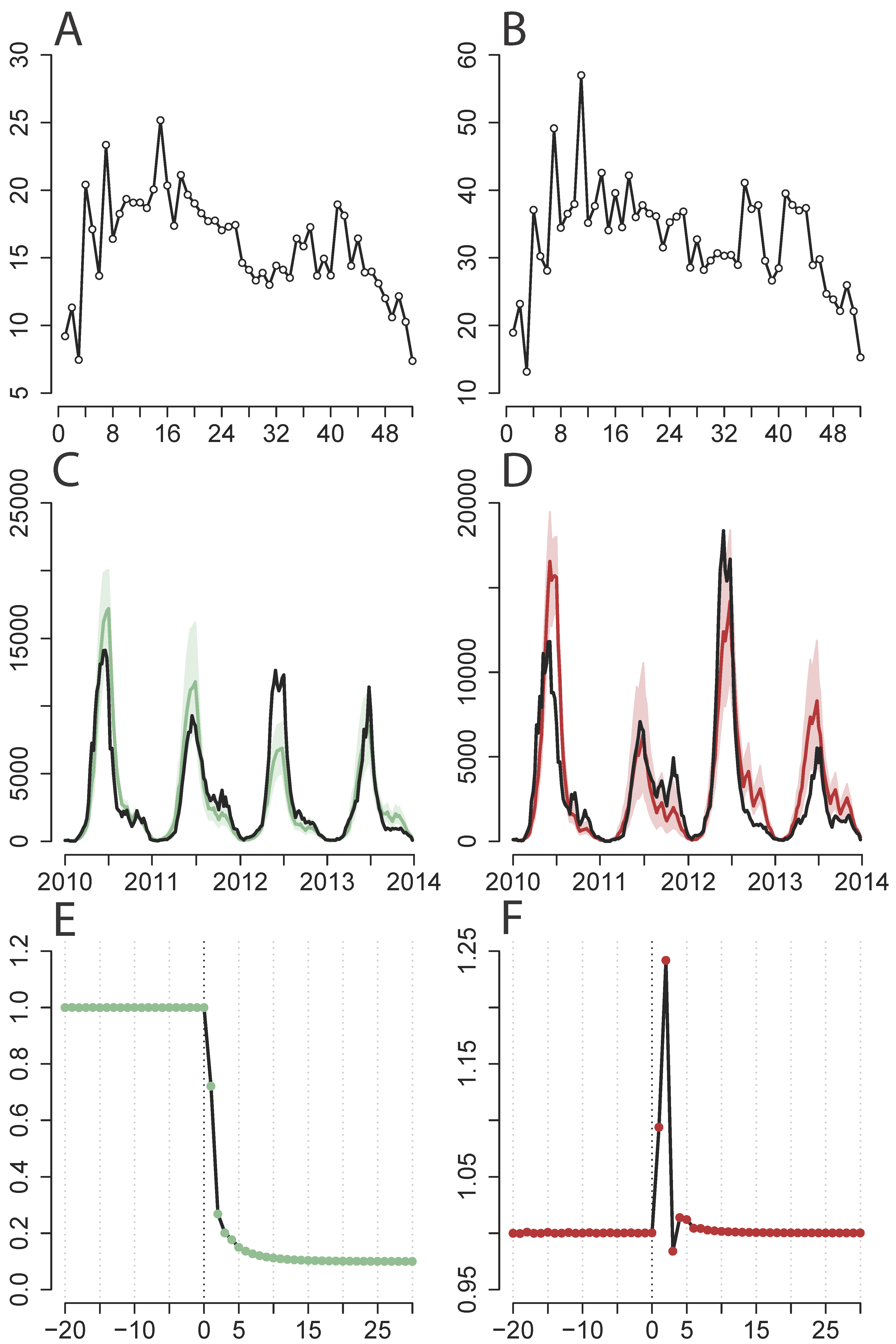

Supplement: S26 Fig — (A and B) Estimated β^s (y-axis) for (A) EV-A71 and (B) CV-A16 by week (x-axis). (C and D) Observed number of cases adjusted for reporting rate (y-axis) from 2010 to 2013 (black line) by week (x-axis) against predictions from 1,000 stochastic simulations of the entire time series for (C) EV-A71 and (D) CV-A16, showing median value (solid colored line) and 5th and 95th percentiles of the simulations (shaded area). (E and F) Output from deterministic simulation of incidence (y-axis) of (E) EV-A71 and (F) CV-A16 by year (x-axis) for 20 y before to 30 y following vaccine initiation (dotted black line, at year 0), normalized by serotype-specific yearly incidence in year −20 and ignoring seasonality in βs. Vaccination assumed to be narrow monovalent EV-A71 vaccine (administered at birth) achieving 90% coverage. S¯ for EV-A71 = 0.094 and S¯ for CV-A16 = 0.046. Calculated with province-specific maximum likelihood estimates of cross-protection (k = 21 wk and δ = 1). (TIFF) [file pmed.1001958.s029.tiff]

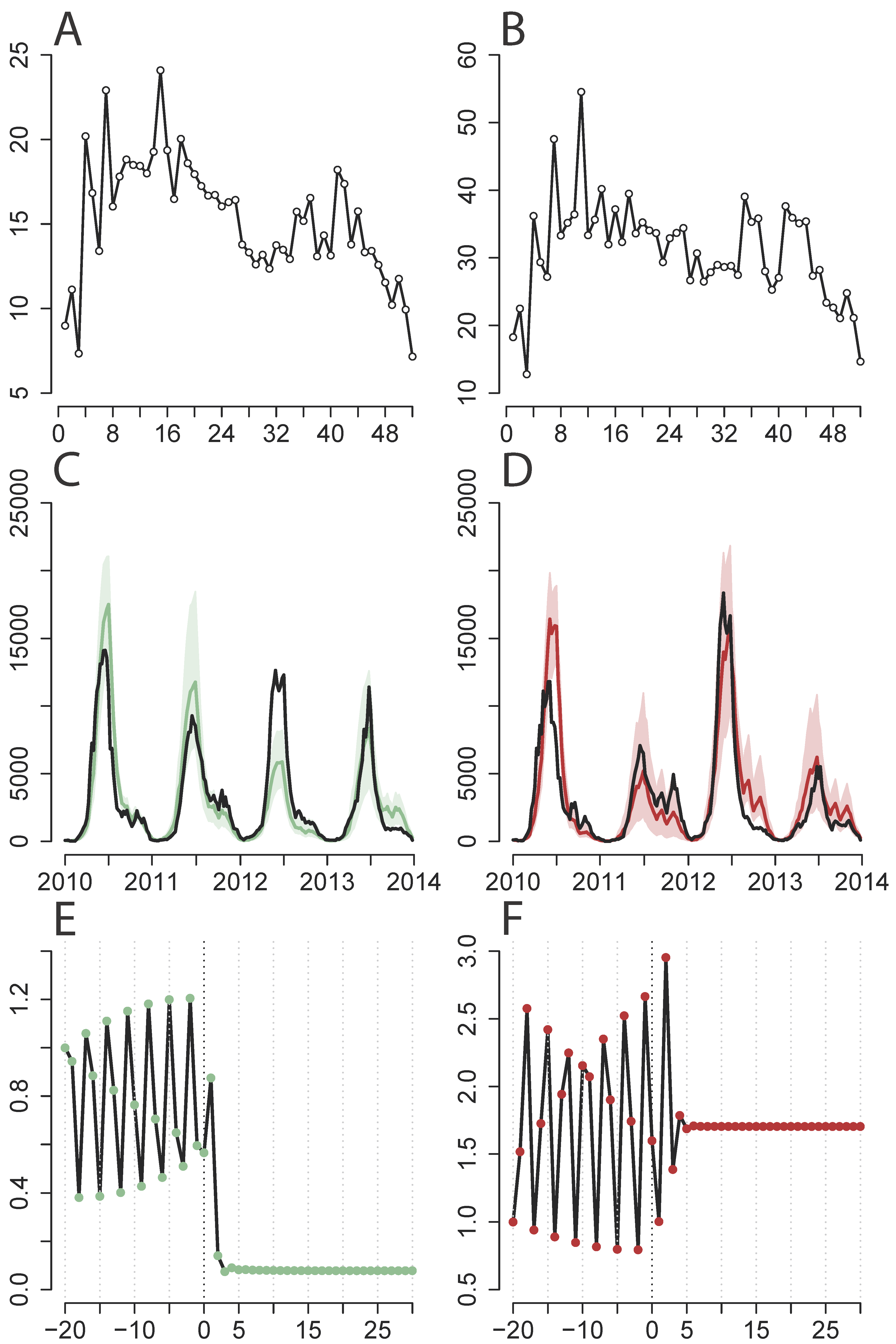

Supplement: S27 Fig — (A and B) Estimated β^s (y-axis) for (A) EV-A71 and (B) CV-A16 by week (x-axis). (C and D) Observed number of cases adjusted for reporting rate (y-axis) from 2010 to 2013 (black line) by week (x-axis) against predictions from 1,000 stochastic simulations of the entire time series for (C) EV-A71 and (D) CV-A16, showing median value (solid colored line) and 5th and 95th percentiles of the simulations (shaded area). (E and F) Output from deterministic simulation of incidence (y-axis) of (E) EV-A71 and (F) CV-A16 by year (x-axis) for 20 y before to 30 y following vaccine initiation (dotted black line, at year 0), normalized by serotype-specific yearly incidence in year −20 and ignoring seasonality in βs. Vaccination assumed to be narrow monovalent EV-A71 vaccine (administered at birth) achieving 90% coverage. S¯ for EV-A71 = 0.091 and S¯ for CV-A16 = 0.045. Calculated with province-specific maximum likelihood estimates of cross-protection (k = 21 wk and δ = 1). (TIFF) [file pmed.1001958.s030.tiff]

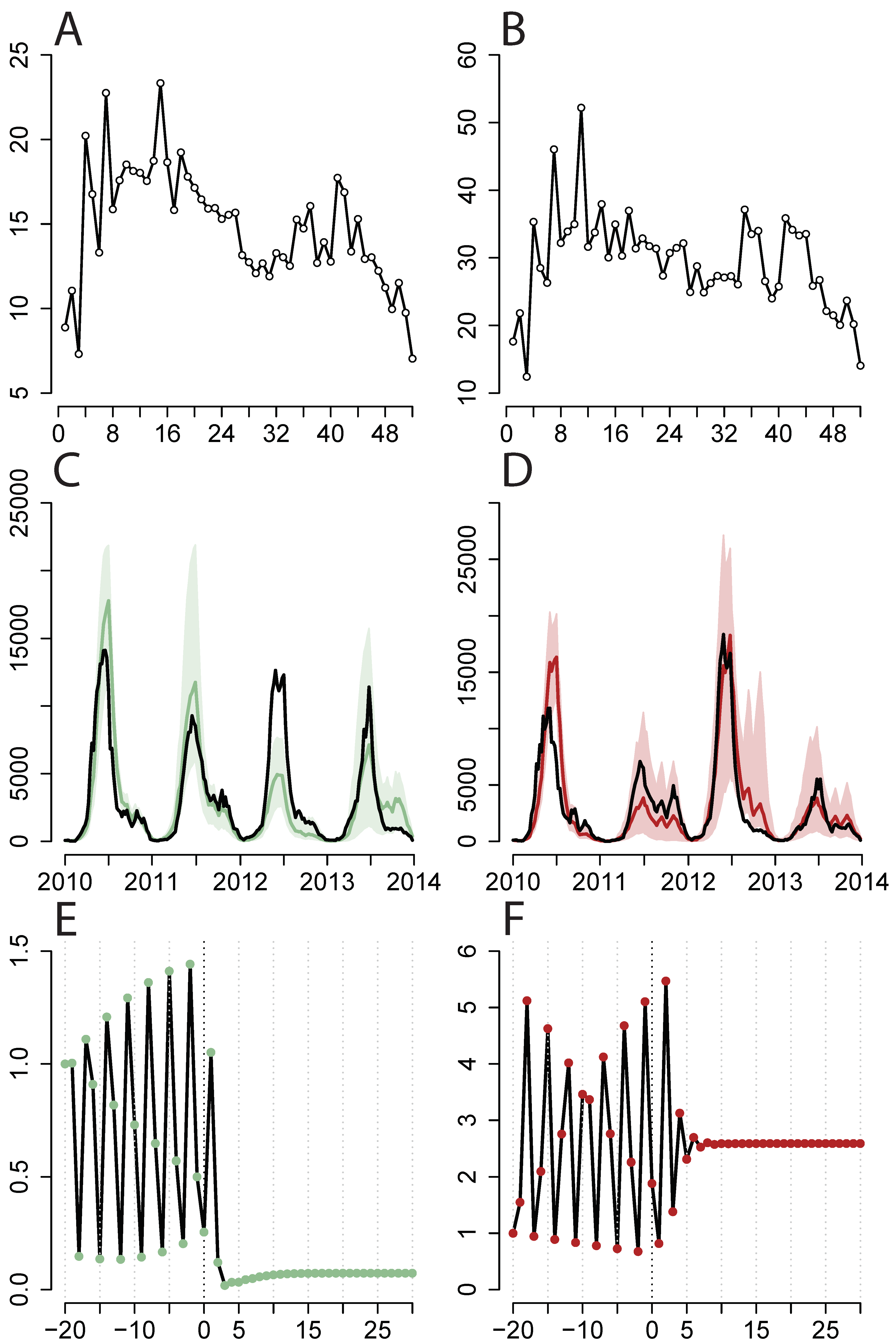

Supplement: S28 Fig — (A and B) Estimated β^s (y-axis) for (A) EV-A71 and (B) CV-A16 by week (x-axis). (C and D) Observed number of cases adjusted for reporting rate (y-axis) from 2010 to 2013 (black line) by week (x-axis) against predictions from 1,000 stochastic simulations of the entire time series for (C) EV-A71 and (D) CV-A16, showing median value (solid colored line) and 5th and 95th percentiles of the simulations (shaded area). (E and F) Output from deterministic simulation of incidence (y-axis) of (E) EV-A71 and (F) CV-A16 by year (x-axis) for 20 y before to 30 y following vaccine initiation (dotted black line, at year 0), normalized by serotype-specific yearly incidence in year −20 and ignoring seasonality in βs. Vaccination assumed to be narrow monovalent EV-A71 vaccine (administered at birth) achieving 90% coverage. S¯ for EV-A71 = 0.087 and S¯ for CV-A16 = 0.044. Calculated with province-specific maximum likelihood estimates of cross-protection (k = 21 wk and δ = 1). (TIFF) [file pmed.1001958.s031.tiff]

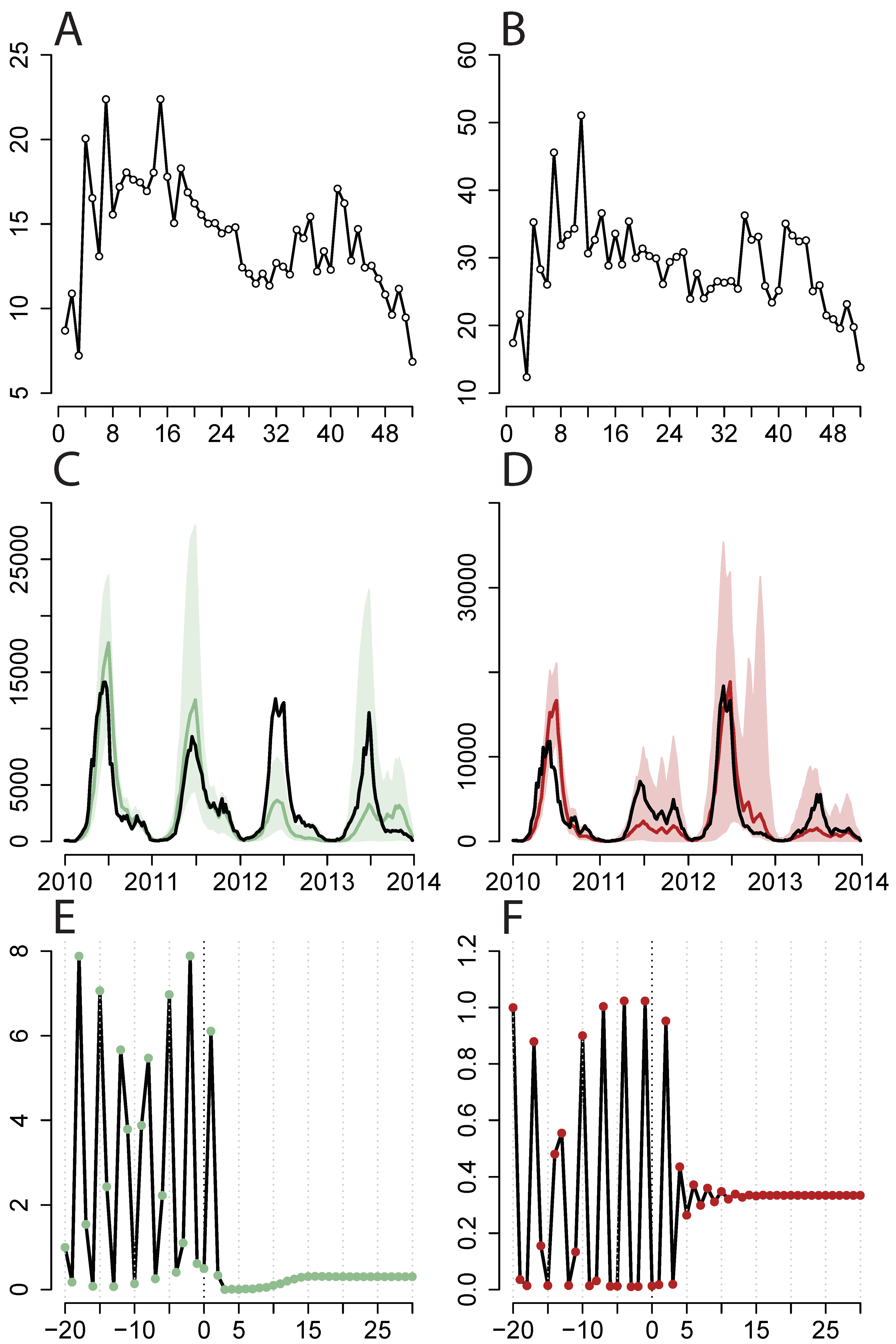

Supplement: S29 Fig — (A and B) Estimated β^s (y-axis) for (A) EV-A71 and (B) CV-A16 by week (x-axis). (C and D) Observed number of cases adjusted for reporting rate (y-axis) from 2010 to 2013 (black line) by week (x-axis) against predictions from 1,000 stochastic simulations of the entire time series for (C) EV-A71 and (D) CV-A16, showing median value (solid colored line) and 5th and 95th percentiles of the simulations (shaded area). (E and F) Output from deterministic simulation of incidence (y-axis) of (E) EV-A71 and (F) CV-A16 by year (x-axis) for 20 y before to 30 y following vaccine initiation (dotted black line, at year 0), normalized by serotype-specific yearly incidence in year −20 and ignoring seasonality in βs. Vaccination assumed to be narrow monovalent EV-A71 vaccine (administered at birth) achieving 90% coverage. S¯ for EV-A71 = 0.084 and S¯ for CV-A16 = 0.042. Calculated with province-specific maximum likelihood estimates of cross-protection (k = 21 wk and δ = 1). (TIFF) [file pmed.1001958.s032.tiff]

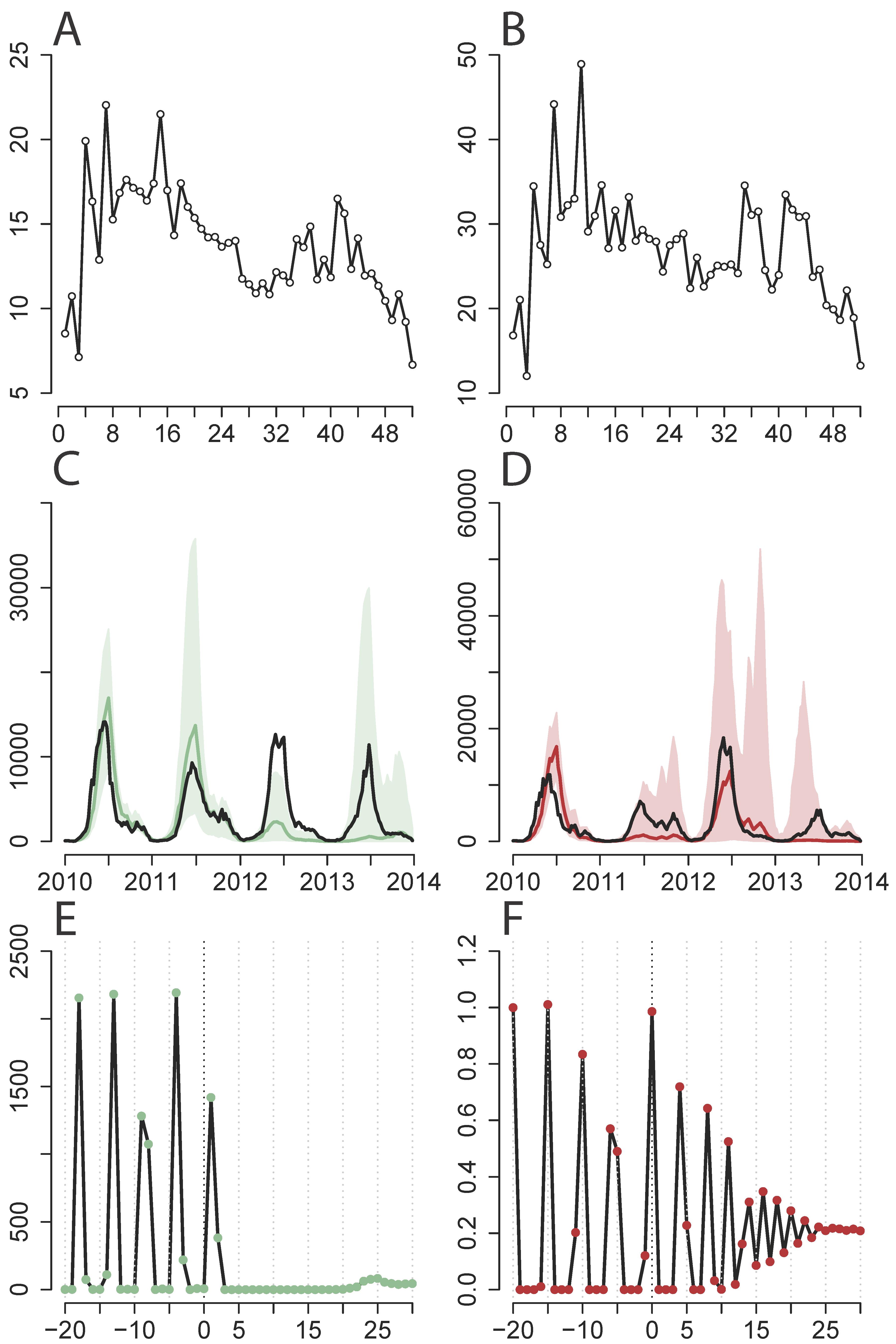

Supplement: S30 Fig — (A and B) Estimated β^s (y-axis) for (A) EV-A71 and (B) CV-A16 by week (x-axis). (C and D) Observed number of cases adjusted for reporting rate (y-axis) from 2010 to 2013 (black line) by week (x-axis) against predictions from 1,000 stochastic simulations of the entire time series for (C) EV-A71 and (D) CV-A16, showing median value (solid colored line) and 5th and 95th percentiles of the simulations (shaded area). (E and F) Output from deterministic simulation of incidence (y-axis) of (E) EV-A71 and (F) CV-A16 by year (x-axis) for 20 y before to 30 y following vaccine initiation (dotted black line, at year 0), normalized by serotype-specific yearly incidence in year −20 and ignoring seasonality in βs. Vaccination assumed to be narrow monovalent EV-A71 vaccine (administered at birth) achieving 90% coverage. S¯ for EV-A71 = 0.081 and S¯ for CV-A16 = 0.041. Calculated with province-specific maximum likelihood estimates of cross-protection (k = 21 wk and δ = 1). (TIFF) [file pmed.1001958.s033.tiff]

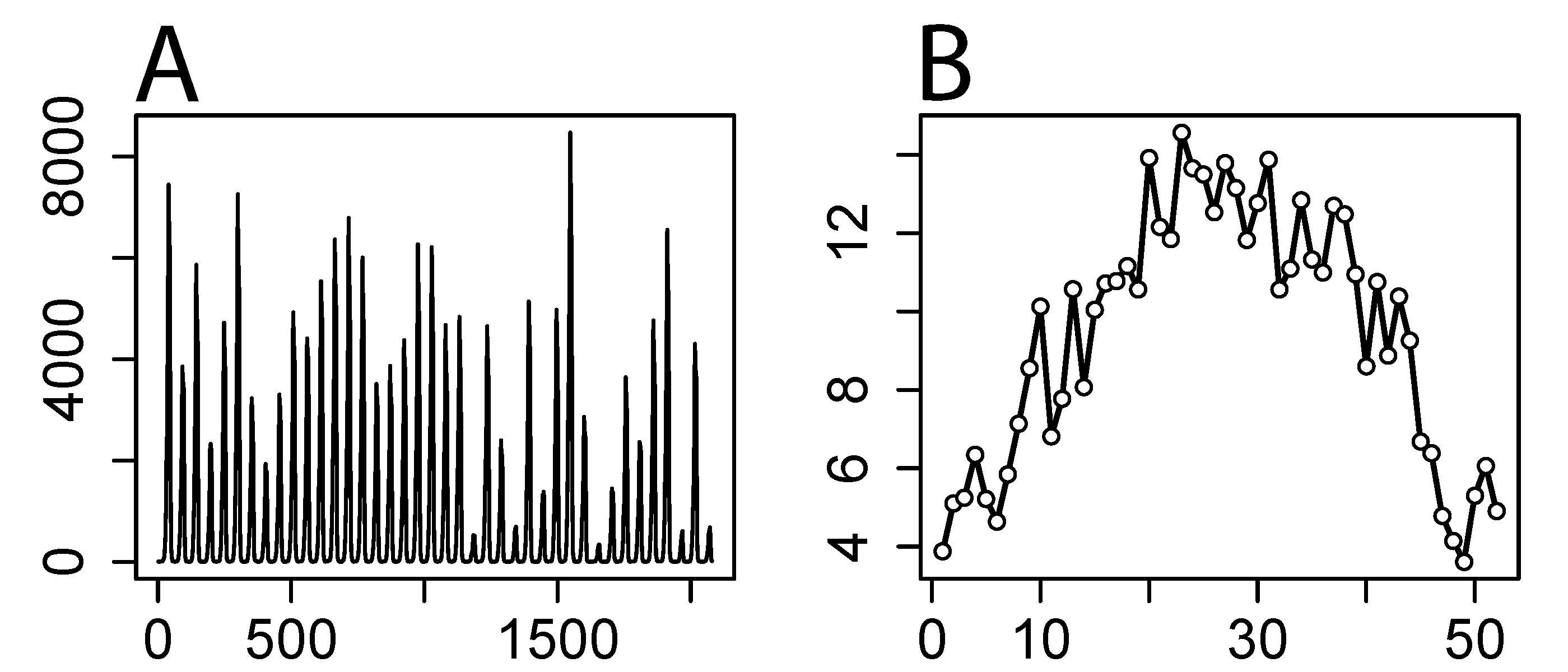

Supplement: S31 Fig — Representative stochastically simulated, single-serotype 40-y time series data of incidence (y-axis) by week (x-axis) (S¯=0.128) (A) with weekly varying (x-axis), stationary βs (β¯=9.407) (y-axis) (B). (TIFF) [file pmed.1001958.s034.tiff]

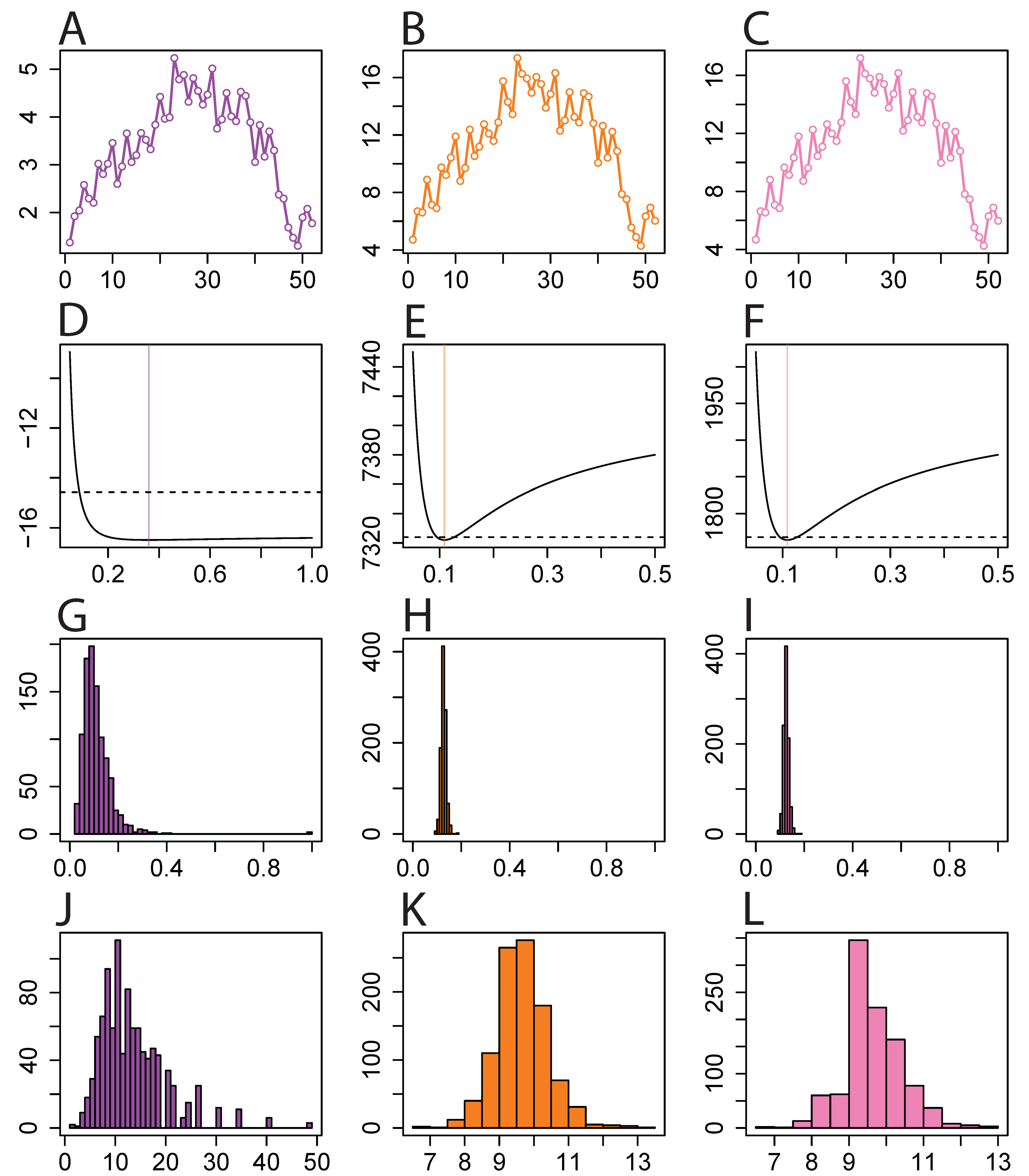

Supplement: S32 Fig — Calculated with α = 0.95, k = 0 wk, and δ = 0, using data from S31 Fig. (A–C) Estimated βs (y-axis) by week (x-axis) for this single time series with three regression models: (A) OLS regression with log-transformed data (β¯ estimated to be 3.352), (B) Poisson GLM with log link (β¯ estimated to be 11.224), and (C) quasi-Poisson GLM with log link (β¯ estimated to be 11.122). (D–F) Estimated S¯ for this single time series (x-axis) and 95% CIs (derived from the profile likelihood [y-axis] using the χ2 distribution with 1 degree of freedom) with three regression models: (D) OLS regression with log-transformed data (0.360, 95% CI: 0.088, 1.000), with negative log-likelihood on the y-axis; (E) Poisson GLM with log link (0.109, 95% CI: 0.095, 0.127), with negative log-likelihood on the y-axis; and (F) quasi-Poisson GLM with log link (0.109, 95% CI: 0.099, 0.121), with deviance on the y-axis. (G–I) Distribution of recovered S¯ values (x-axis) from 1,000 stochastically simulated time series (S¯=0.128 and seasonally varying, stationary βs, with β¯=9.407 as in S31 Fig) with three regression models: (G) OLS regression with log-transformed data, (H) Poisson GLM with log link, and (I) quasi-Poisson GLM with log link. (J–L) Distribution of recovered β¯ values (x-axis) from 1,000 stochastically simulated time series (S¯=0.128 and seasonally varying, stationary βs, with β¯=9.407 as in S31 Fig) with three regression models: (J) OLS regression with log-transformed data, (K) Poisson GLM with log link, and (L) quasi-Poisson GLM with log link. (TIFF) [file pmed.1001958.s035.tiff]

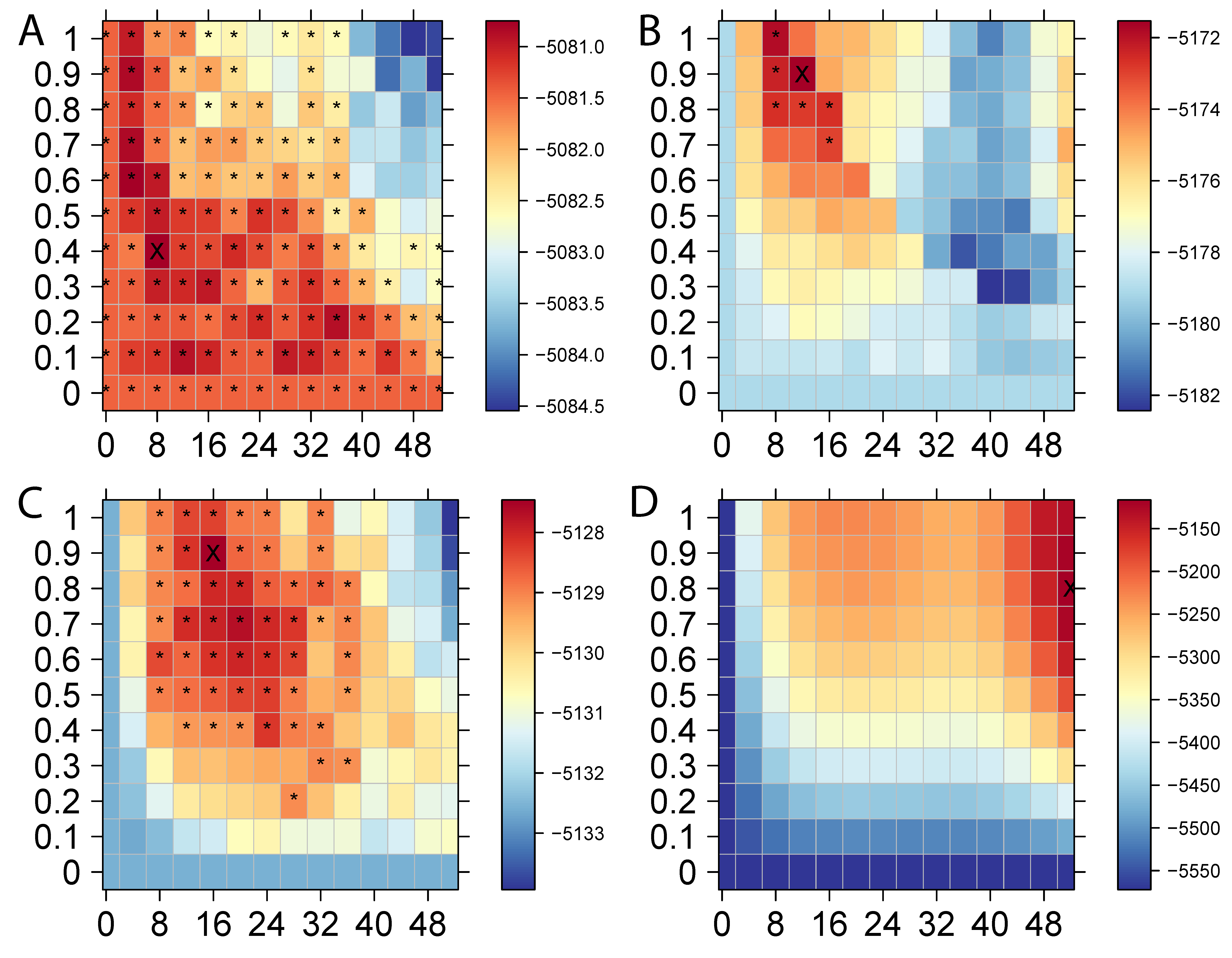

Supplement: S33 Fig — Estimated log-likelihood values over a range of the cross-protection parameters k, from 0 to 52 (in weeks; x-axis), and δ, from 0 to 1 (as proportion; y-axis), in the two-serotype model for α = 0.95, showing the maximum likelihood estimate (grid cell with “X”) and 95% confidence region (grid cells with asterisk) for simulated two-serotype data with true cross-protection parameter values of (A) k = 0 wk and δ = 0 (no cross-protection), (B) k = 12 wk and δ = 1, (C) k = 24 wk and δ = 1, and (D) k = 48 wk and δ = 1. (TIFF) [file pmed.1001958.s036.tiff]

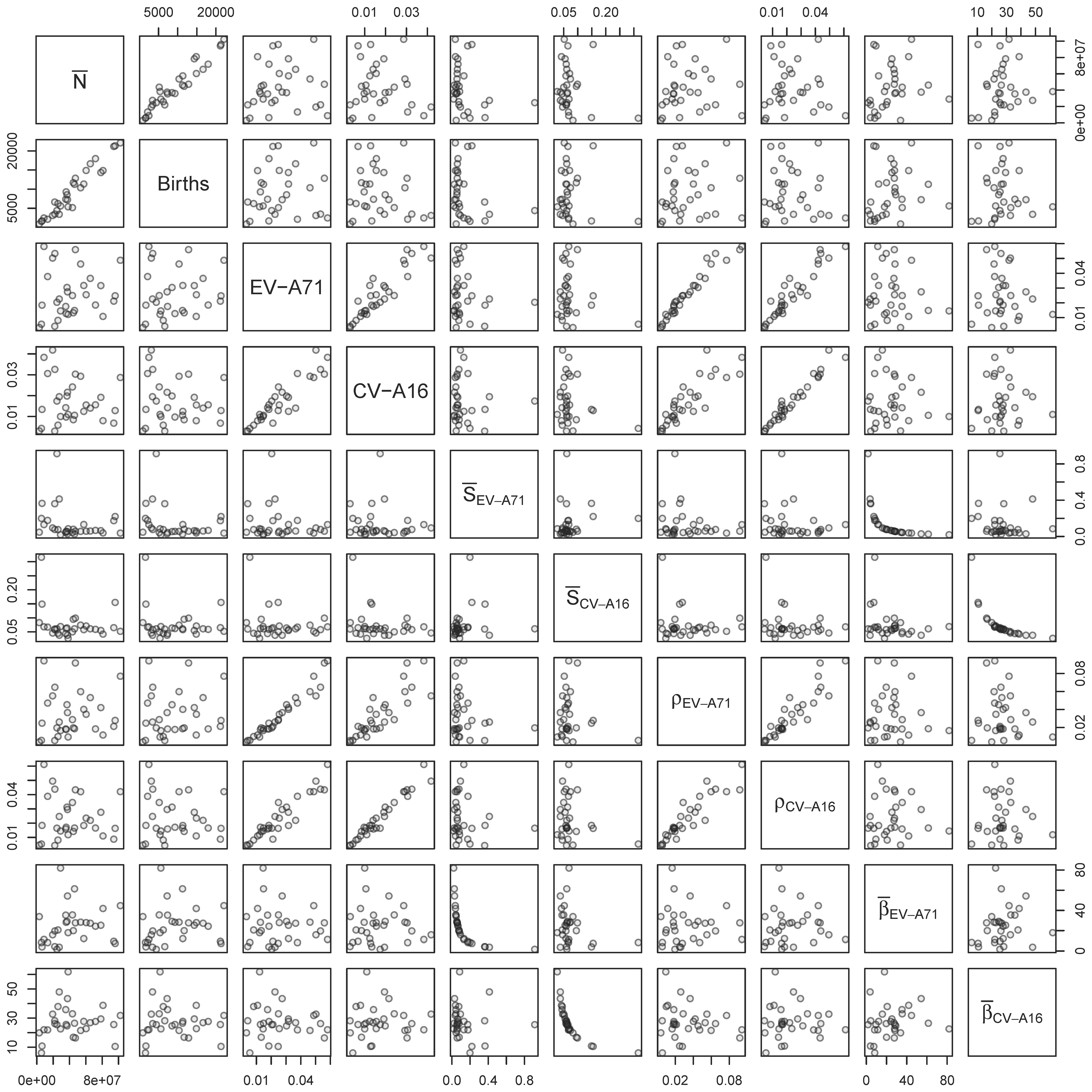

Supplement: S34 Fig — Each data point represents a province; from top left: mean population size from 2009 to 2013, mean births per week from 2009 to 2013, ratio of total reported EV-A71 cases to total births from 2009 to 2013, ratio of total reported CV-A16 cases to total births from 2009 to 2013, estimated S¯ for EV-A71 and CV-A16, estimated reporting rate ρ for EV-A71 and CV-A16, and estimated β¯ for EV-A71 and CV-A16. Calculated with α = 0.95 and the province-specific maximum likelihood estimates of cross-protection, using the two-serotype model. (TIFF) [file pmed.1001958.s037.tiff]
