# Supplementary material for: Hand, Foot, and Mouth Disease in China: Modeling Epidemic Dynamics of Enterovirus Serotypes and Implications for Vaccination
Source: PLoS Med. 2016 Feb 16;13(2):e1001958. doi: 10.1371/journal.pmed.1001958 (PMC4755668; doi:10.1371/journal.pmed.1001958)
Supplement: S1 Table — Mean proportion of individuals that are susceptible to EV-A71 and CV-A16 (S¯), reporting rate of EV-A71 and CV-A16 (ρ), mean weekly estimated transmission rate of EV-A71 and CV-A16 (β¯), and coefficient of variation (CV) in estimated transmission rate of EV-A71 and CV-A16 by province. Calculated with α = 0.95 and province-specific maximum likelihood estimates of cross-protection. The 95% CIs for S¯ are derived from the profile likelihood using the χ2 distribution with 1 degree of freedom; the 95% CIs for ρ are derived from the standard errors for the coefficient 1/ρ in the OLS regression of cumulative births and cumulative cases; the maximum standard errors (se) of the log(βs) terms across weekly β^s values are from the log-linear regression, representing variability around estimated βs. (DOCX) [file pmed.1001958.s038.docx]

**S1 Table. Two-serotype TSIR model estimates for EV-A71 and CV-A16.** Mean proportion of individuals that are susceptible to EV-A71 and CV-A16 ($\bar{S}$), reporting rate of EV-A71 and CV-A16 ($\rho$), mean weekly estimated transmission rate of EV-A71 and CV-A16 ($\bar{\beta}$), and coefficient of variation (CV) in estimated transmission rate of EV-A71 and CV-A16 by province. Calculated with $\alpha$ = 0.95 and province-specific maximum likelihood estimates of cross-protection. The 95% CIs for $\bar{S}$ are derived from the profile likelihood using the $\chi^{2}$ distribution with 1 degree of freedom; the 95% CIs for $\rho$ are derived from the standard errors for the coefficient $1/\rho$ in the OLS regression of cumulative births and cumulative cases; the maximum standard errors (se) of the $\log\left( \beta_{s} \right)$ terms across weekly $\hat{\beta}_{s}$ values are from the log-linear regression, representing variability around estimated $\beta_{s}$.

| Province | $\bar{\boldsymbol{S}}$ of  EV-A71  (CI) | $\bar{\boldsymbol{S}}$ of  CV-A16  (CI) | $\boldsymbol{\rho}$ of  EV-A71  (CI) | $\boldsymbol{\rho}$ of  CV-A16  (CI) | $\bar{\boldsymbol{\beta}}$ of  EV-A71  (se) | $\bar{\boldsymbol{\beta}}$ of  CV-A16 (se) | CV of $\boldsymbol{\beta}_{\mathbf{s}}$, EV-A71 | CV of $\boldsymbol{\beta}_{\mathbf{s}}$, CV-A16 |
| --- | --- | --- | --- | --- | --- | --- | --- | --- |
| Beijing | 0.094 (0.090, 0.099) | 0.046 (0.045, 0.047) | 0.055 (0.052, 0.058) | 0.049 (0.047, 0.052) | 15.89 (0.06) | 32.75 (0.06) | 0.23 | 0.24 |
| Tianjin | 0.177 (0.155, 0.207) | 0.067 (0.063, 0.072) | 0.047 (0.044, 0.050) | 0.039 (0.037, 0.041) | 8.22 (0.09) | 21.90 (0.09) | 0.29 | 0.27 |
| Hebei | 0.067 (0.066, 0.068) | 0.059 (0.059, 0.059) | 0.018 (0.018, 0.019) | 0.017 (0.016, 0.017) | 24.35 (0.02) | 27.69 (0.02) | 0.25 | 0.26 |
| Shanxi | 0.044 (0.042, 0.046) | 0.044 (0.044, 0.045) | 0.018 (0.018, 0.019) | 0.016 (0.016, 0.016) | 35.32 (0.05) | 35.84 (0.04) | 0.27 | 0.32 |
| Inner Mongolia | 0.913 (0.333, 1.000) | 0.061 (0.057, 0.065) | 0.020 (0.019, 0.020) | 0.016 (0.016, 0.017) | 1.69 (0.07) | 25.51 (0.07) | 0.31 | 0.33 |
| Liaoning | 0.043 (0.042, 0.045) | 0.050 (0.048, 0.051) | 0.029 (0.028, 0.030) | 0.034 (0.033, 0.036) | 35.55 (0.06) | 32.97 (0.23) | 0.34 | 0.69 |
| Jilin | 0.413 (0.281, 0.781) | 0.038 (0.036, 0.041) | 0.026 (0.025, 0.027) | 0.025 (0.024, 0.025) | 3.74 (0.07) | 48.00 (0.24) | 0.40 | 1.08 |
| Heilongjiang | 0.085 (0.078, 0.094) | 0.027 (0.027, 0.027) | 0.009 (0.009, 0.010) | 0.012 (0.011, 0.012) | 17.99 (0.06) | 61.81 (0.15) | 0.33 | 0.56 |
| Shanghai | 0.077 (0.073, 0.082) | 0.058 (0.056, 0.061) | 0.065 (0.063, 0.067) | 0.044 (0.043, 0.045) | 19.69 (0.03) | 26.23 (0.02) | 0.21 | 0.20 |
| Jiangsu | 0.064 (0.062, 0.066) | 0.055 (0.054, 0.056) | 0.042 (0.041, 0.043) | 0.028 (0.027, 0.028) | 25.32 (0.01) | 29.44 (0.01) | 0.20 | 0.19 |
| Zhejiang | 0.056 (0.055, 0.058) | 0.074 (0.072, 0.076) | 0.060 (0.058, 0.061) | 0.042 (0.041, 0.043) | 28.38 (0.01) | 21.52 (0.01) | 0.19 | 0.19 |
| Anhui | 0.058 (0.057, 0.059) | 0.071 (0.070, 0.073) | 0.035 (0.034, 0.035) | 0.024 (0.023, 0.024) | 27.93 (0.01) | 22.77 (0.01) | 0.17 | 0.18 |
| Fujian | 0.054 (0.053, 0.055) | 0.064 (0.062, 0.066) | 0.045 (0.044, 0.046) | 0.031 (0.03, 0.032) | 29.18 (0.01) | 24.67 (0.01) | 0.17 | 0.17 |
| Jiangxi | 0.080 (0.078, 0.083) | 0.095 (0.092, 0.097) | 0.020 (0.019, 0.020) | 0.014 (0.014, 0.015) | 19.98 (0.01) | 16.85 (0.01) | 0.19 | 0.19 |
| Shandong | 0.220 (0.209, 0.233) | 0.155 (0.146, 0.165) | 0.028 (0.027, 0.029) | 0.016 (0.016, 0.017) | 7.41 (0.02) | 10.53 (0.02) | 0.22 | 0.22 |
| Henan | 0.174 (0.165, 0.185) | 0.065 (0.065, 0.066) | 0.021 (0.021, 0.022) | 0.009 (0.009, 0.009) | 9.46 (0.01) | 25.60 (0.01) | 0.22 | 0.18 |
| Hubei | 0.132 (0.118, 0.148) | 0.058 (0.057, 0.059) | 0.040 (0.039, 0.041) | 0.018 (0.018, 0.019) | 12.16 (0.01) | 27.76 (0.01) | 0.23 | 0.21 |
| Hunan | 0.060 (0.059, 0.061) | 0.061 (0.061, 0.062) | 0.053 (0.052, 0.055) | 0.022 (0.021, 0.023) | 27.30 (0.02) | 26.88 (0.01) | 0.23 | 0.22 |
| Guangdong | 0.037 (0.037, 0.037) | 0.052 (0.052, 0.052) | 0.077 (0.075, 0.079) | 0.042 (0.041, 0.044) | 44.72 (0.01) | 31.81 (0.01) | 0.17 | 0.17 |
| Guangxi | 0.058 (0.057, 0.059) | 0.099 (0.098, 0.101) | 0.092 (0.088, 0.095) | 0.043 (0.042, 0.045) | 27.71 (0.01) | 16.38 (0.01) | 0.19 | 0.18 |
| Hainan | 0.131 (0.121, 0.145) | 0.068 (0.065, 0.072) | 0.094 (0.092, 0.097) | 0.061 (0.06, 0.063) | 11.32 (0.02) | 22.02 (0.02) | 0.15 | 0.19 |
| Chongqing | 0.019 (0.019, 0.019) | 0.069 (0.066, 0.073) | 0.017 (0.016, 0.017) | 0.014 (0.014, 0.015) | 81.95 (0.02) | 22.59 (0.02) | 0.21 | 0.22 |
| Sichuan | 0.039 (0.038, 0.039) | 0.042 (0.042, 0.043) | 0.012 (0.011, 0.012) | 0.011 (0.011, 0.011) | 41.90 (0.01) | 38.76 (0.01) | 0.18 | 0.18 |
| Guizhou | 0.056 (0.055, 0.057) | 0.062 (0.061, 0.063) | 0.018 (0.017, 0.018) | 0.017 (0.017, 0.018) | 28.23 (0.01) | 25.64 (0.01) | 0.20 | 0.20 |
| Yunnan | 0.026 (0.025, 0.026) | 0.061 (0.060, 0.063) | 0.019 (0.018, 0.019) | 0.017 (0.016, 0.017) | 61.39 (0.01) | 26.49 (0.01) | 0.14 | 0.16 |
| Tibet | 0.047 (0.046, 0.048) | 0.083 (0.078, 0.089) | 0.004 (0.004, 0.004) | 0.005 (0.004, 0.005) | 33.88 (0.18) | 19.90 (0.24) | 0.54 | 0.63 |
| Shaanxi | 0.029 (0.028, 0.030) | 0.038 (0.038, 0.038) | 0.037 (0.036, 0.037) | 0.030 (0.029, 0.030) | 54.30 (0.03) | 43.41 (0.05) | 0.26 | 0.50 |
| Gansu | 0.076 (0.071, 0.082) | 0.045 (0.044, 0.046) | 0.010 (0.010, 0.010) | 0.008 (0.008, 0.008) | 20.66 (0.05) | 37.96 (0.14) | 0.34 | 0.66 |
| Qinghai | 0.199 (0.185, 0.216) | 0.316 (0.285, 0.354) | 0.006 (0.005, 0.006) | 0.005 (0.005, 0.006) | 8.32 (0.19) | 6.09 (0.50) | 0.70 | 1.34 |
| Ningxia | 0.365 (0.207, 1.000) | 0.149 (0.124, 0.185) | 0.025 (0.024, 0.025) | 0.018 (0.018, 0.019) | 4.04 (0.11) | 10.83 (0.16) | 0.32 | 0.81 |
| Xinjiang | 0.361 (0.285, 0.495) | 0.060 (0.059, 0.062) | 0.006 (0.005, 0.006) | 0.004 (0.004, 0.004) | 4.36 (0.06) | 28.19 (0.14) | 0.33 | 0.69 |
