# Supplementary material for: Hand, Foot, and Mouth Disease in China: Modeling Epidemic Dynamics of Enterovirus Serotypes and Implications for Vaccination
Source: PLoS Med. 2016 Feb 16;13(2):e1001958. doi: 10.1371/journal.pmed.1001958 (PMC4755668; doi:10.1371/journal.pmed.1001958)
Supplement: S2 Table — β^s and 95% CIs on each weekly βs value (row) for EV-A71 for each province (column) from the two-serotype model with α = 0.95 and province-specific maximum likelihood estimates of cross-protection. (DOCX) [file pmed.1001958.s039.docx]

**S2 Table. Spatial and temporal variation in** $\boldsymbol{R}_{\boldsymbol{0}}$ **of EV-A71 across provinces.** $\hat{\beta}_{s}$ and 95% CIs on each weekly $\beta_{s}$ value (row) for EV-A71 for each province (column) from the two-serotype model with $\alpha$ = 0.95 and province-specific maximum likelihood estimates of cross-protection.

|  | Beijing | Tianjin | Hebei | Shanxi | Inner Mongolia |
| --- | --- | --- | --- | --- | --- |
| 1 | 9.21 (8.45, 10.01) | 4.64 (4.13, 5.19) | 15.06 (14.58, 15.55) | 20.29 (19.24, 21.39) | 0.8 (0.75, 0.85) |
| 2 | 11.33 (10.31, 12.42) | 5.25 (4.57, 5.98) | 11.99 (11.48, 12.51) | 17.81 (16.58, 19.09) | 0.72 (0.65, 0.79) |
| 3 | 7.46 (6.57, 8.44) | 6.37 (5.52, 7.31) | 20.47 (19.6, 21.36) | 23.06 (21.26, 24.95) | 1.37 (1.24, 1.51) |
| 4 | 20.41 (18.43, 22.54) | 3.98 (3.29, 4.77) | 20.75 (19.87, 21.64) | 33.75 (31.3, 36.33) | 0.76 (0.67, 0.87) |
| 5 | 17.11 (15.63, 18.69) | 12.39 (10.74, 14.2) | 20.69 (19.82, 21.58) | 21.16 (19.34, 23.09) | 2.15 (1.94, 2.38) |
| 6 | 13.67 (12.49, 14.93) | 6.54 (5.63, 7.54) | 20.21 (19.35, 21.09) | 38.45 (35.55, 41.5) | 1.17 (1.04, 1.31) |
| 7 | 23.36 (21.8, 24.98) | 12.2 (10.89, 13.6) | 25.57 (24.6, 26.56) | 38.54 (36, 41.19) | 2.55 (2.35, 2.77) |
| 8 | 16.4 (15.41, 17.44) | 7.71 (6.91, 8.56) | 23.74 (22.91, 24.6) | 35.85 (33.71, 38.09) | 1 (0.9, 1.1) |
| 9 | 18.26 (17.3, 19.25) | 10.63 (9.73, 11.58) | 27.07 (26.24, 27.91) | 35.69 (33.75, 37.71) | 2.62 (2.44, 2.82) |
| 10 | 19.35 (18.5, 20.24) | 7.92 (7.28, 8.6) | 28.31 (27.57, 29.06) | 36.29 (34.5, 38.14) | 1.72 (1.61, 1.83) |
| 11 | 19.08 (18.36, 19.83) | 7.54 (6.95, 8.17) | 27.42 (26.8, 28.05) | 54.72 (52.72, 56.77) | 1.72 (1.61, 1.82) |
| 12 | 19.09 (18.46, 19.73) | 10.83 (10.14, 11.56) | 30.09 (29.52, 30.66) | 41.68 (40.38, 43.02) | 2.03 (1.93, 2.14) |
| 13 | 18.68 (18.15, 19.23) | 8.19 (7.69, 8.71) | 36.44 (35.91, 36.97) | 47.86 (46.65, 49.09) | 2.13 (2.04, 2.22) |
| 14 | 20.06 (19.57, 20.56) | 12.48 (11.9, 13.08) | 36.77 (36.36, 37.18) | 58.08 (56.99, 59.17) | 1.67 (1.61, 1.74) |
| 15 | 25.18 (24.71, 25.65) | 14.22 (13.74, 14.71) | 41.79 (41.45, 42.13) | 49.12 (48.37, 49.88) | 2.64 (2.56, 2.72) |
| 16 | 20.35 (20.02, 20.68) | 9.91 (9.62, 10.21) | 32.89 (32.67, 33.12) | 45.3 (44.71, 45.9) | 1.77 (1.72, 1.82) |
| 17 | 17.37 (17.11, 17.64) | 11.29 (11.01, 11.57) | 33.72 (33.52, 33.91) | 44.84 (44.34, 45.35) | 1.94 (1.89, 1.99) |
| 18 | 21.13 (20.85, 21.4) | 11.82 (11.59, 12.06) | 32.92 (32.76, 33.08) | 45.13 (44.69, 45.58) | 2.72 (2.67, 2.78) |
| 19 | 19.67 (19.44, 19.9) | 12.37 (12.17, 12.57) | 32.64 (32.5, 32.78) | 51.3 (50.89, 51.71) | 2.48 (2.44, 2.52) |
| 20 | 19.02 (18.82, 19.23) | 10.98 (10.82, 11.13) | 28.64 (28.53, 28.76) | 47.33 (47, 47.66) | 2.32 (2.29, 2.35) |
| 21 | 18.3 (18.12, 18.49) | 11.5 (11.36, 11.64) | 28.54 (28.42, 28.65) | 43.3 (43.03, 43.58) | 2.6 (2.57, 2.63) |
| 22 | 17.71 (17.54, 17.89) | 10.28 (10.17, 10.39) | 25.07 (24.97, 25.17) | 41.05 (40.8, 41.3) | 2.11 (2.09, 2.13) |
| 23 | 17.76 (17.59, 17.93) | 9.07 (8.97, 9.17) | 25.49 (25.38, 25.59) | 35.46 (35.25, 35.68) | 2.08 (2.06, 2.1) |
| 24 | 17.04 (16.88, 17.21) | 8.86 (8.77, 8.96) | 24.2 (24.09, 24.31) | 34.56 (34.33, 34.78) | 1.93 (1.91, 1.94) |
| 25 | 17.3 (17.14, 17.47) | 9.5 (9.4, 9.6) | 26.94 (26.82, 27.05) | 35.44 (35.2, 35.67) | 1.93 (1.91, 1.94) |
| 26 | 17.44 (17.27, 17.6) | 8.63 (8.54, 8.72) | 25.44 (25.33, 25.56) | 33.35 (33.11, 33.59) | 1.76 (1.74, 1.77) |
| 27 | 14.63 (14.48, 14.78) | 6.7 (6.62, 6.78) | 22.02 (21.91, 22.13) | 30.01 (29.76, 30.25) | 1.47 (1.46, 1.48) |
| 28 | 14.12 (13.96, 14.28) | 7.15 (7.06, 7.25) | 21.89 (21.77, 22.01) | 30.87 (30.59, 31.15) | 1.4 (1.38, 1.41) |
| 29 | 13.33 (13.15, 13.5) | 6.91 (6.81, 7.02) | 21.47 (21.34, 21.6) | 28.88 (28.59, 29.18) | 1.28 (1.26, 1.29) |
| 30 | 13.9 (13.7, 14.1) | 6.76 (6.65, 6.88) | 21.78 (21.64, 21.93) | 32.28 (31.92, 32.63) | 1.38 (1.37, 1.4) |
| 31 | 13 (12.79, 13.22) | 6.03 (5.91, 6.15) | 19.95 (19.8, 20.1) | 30.22 (29.85, 30.6) | 1.27 (1.25, 1.29) |
| 32 | 14.43 (14.17, 14.69) | 6.41 (6.26, 6.57) | 21.02 (20.85, 21.19) | 27.69 (27.3, 28.09) | 1.27 (1.25, 1.29) |
| 33 | 14.13 (13.85, 14.4) | 7.23 (7.05, 7.42) | 21.29 (21.1, 21.48) | 27.69 (27.24, 28.14) | 1.52 (1.5, 1.55) |
| 34 | 13.53 (13.24, 13.82) | 6.36 (6.18, 6.55) | 20.98 (20.78, 21.18) | 30.09 (29.56, 30.62) | 1.49 (1.46, 1.52) |
| 35 | 16.43 (16.08, 16.78) | 8.8 (8.55, 9.05) | 24.96 (24.72, 25.19) | 33.78 (33.18, 34.4) | 1.84 (1.81, 1.88) |
| 36 | 15.85 (15.51, 16.19) | 7.57 (7.35, 7.8) | 22.17 (21.95, 22.39) | 45.54 (44.82, 46.26) | 2.19 (2.16, 2.22) |
| 37 | 17.28 (16.92, 17.64) | 8.38 (8.14, 8.63) | 27.36 (27.1, 27.61) | 43.15 (42.54, 43.78) | 2.12 (2.09, 2.15) |
| 38 | 13.68 (13.37, 13.99) | 7.13 (6.91, 7.36) | 20.52 (20.31, 20.73) | 35.36 (34.85, 35.88) | 1.66 (1.63, 1.68) |
| 39 | 14.95 (14.59, 15.31) | 7.56 (7.32, 7.81) | 26.83 (26.57, 27.1) | 36.96 (36.44, 37.49) | 1.52 (1.5, 1.54) |
| 40 | 13.7 (13.35, 14.06) | 6.98 (6.74, 7.23) | 20.28 (20.06, 20.5) | 30.1 (29.64, 30.57) | 1.32 (1.3, 1.34) |
| 41 | 18.95 (18.51, 19.4) | 9.79 (9.48, 10.1) | 28.55 (28.27, 28.84) | 46.82 (46.19, 47.46) | 2.45 (2.42, 2.48) |
| 42 | 18.13 (17.73, 18.53) | 7.89 (7.64, 8.14) | 27.72 (27.47, 27.98) | 41.23 (40.71, 41.75) | 1.85 (1.83, 1.88) |
| 43 | 14.41 (14.08, 14.75) | 8.99 (8.72, 9.27) | 23.42 (23.2, 23.64) | 34.14 (33.7, 34.59) | 1.47 (1.45, 1.49) |
| 44 | 16.44 (16.06, 16.81) | 9.09 (8.84, 9.35) | 25.73 (25.49, 25.96) | 38.38 (37.9, 38.87) | 1.56 (1.54, 1.58) |
| 45 | 13.92 (13.59, 14.26) | 6.98 (6.77, 7.2) | 23.6 (23.38, 23.81) | 31.65 (31.23, 32.08) | 1.47 (1.45, 1.49) |
| 46 | 13.99 (13.63, 14.35) | 6.91 (6.68, 7.14) | 20.52 (20.31, 20.73) | 28.57 (28.14, 29) | 1.3 (1.28, 1.32) |
| 47 | 13.11 (12.75, 13.48) | 6.62 (6.38, 6.86) | 20.76 (20.53, 20.98) | 26.43 (25.98, 26.89) | 1.46 (1.44, 1.49) |
| 48 | 12.01 (11.64, 12.39) | 6.17 (5.92, 6.42) | 17.56 (17.34, 17.78) | 27.7 (27.16, 28.24) | 1.32 (1.3, 1.35) |
| 49 | 10.61 (10.22, 11.01) | 5.5 (5.24, 5.77) | 18.09 (17.83, 18.35) | 24.07 (23.52, 24.63) | 1.47 (1.44, 1.51) |
| 50 | 12.17 (11.68, 12.67) | 5.97 (5.65, 6.29) | 16.57 (16.3, 16.85) | 23.49 (22.85, 24.14) | 1.02 (0.99, 1.05) |
| 51 | 10.27 (9.78, 10.77) | 4.59 (4.28, 4.91) | 14.53 (14.23, 14.84) | 20.7 (20, 21.41) | 1.18 (1.14, 1.21) |
| 52 | 7.38 (6.89, 7.89) | 3.8 (3.45, 4.17) | 13.75 (13.39, 14.11) | 21.44 (20.56, 22.34) | 0.65 (0.62, 0.68) |

|  | Liaoning | Jilin | Heilongjiang |
| --- | --- | --- | --- |
| 1 | 15.88 (14.67, 17.15) | 2.48 (2.27, 2.7) | 8.85 (8.32, 9.39) |
| 2 | 26.51 (24.37, 28.78) | 2.33 (2.1, 2.57) | 13.04 (12.17, 13.95) |
| 3 | 18.25 (16.37, 20.27) | 3.15 (2.84, 3.48) | 13.41 (12.43, 14.43) |
| 4 | 42.46 (38.83, 46.32) | 3.16 (2.85, 3.49) | 13.54 (12.47, 14.66) |
| 5 | 15.44 (13.63, 17.4) | 1.66 (1.44, 1.91) | 11.38 (10.33, 12.51) |
| 6 | 74.22 (68.74, 79.97) | 6.24 (5.65, 6.87) | 12.14 (10.88, 13.5) |
| 7 | 20.53 (18.7, 22.47) | 2.57 (2.3, 2.86) | 17.01 (15.33, 18.82) |
| 8 | 42.72 (39.54, 46.06) | 3.93 (3.56, 4.32) | 19.48 (17.75, 21.31) |
| 9 | 45.71 (42.95, 48.58) | 5.13 (4.76, 5.53) | 25.9 (24.12, 27.75) |
| 10 | 38.08 (36.03, 40.2) | 1.86 (1.68, 2.05) | 15.87 (14.79, 17) |
| 11 | 20.39 (19.06, 21.78) | 3.11 (2.81, 3.43) | 10.3 (9.44, 11.21) |
| 12 | 29.87 (27.93, 31.89) | 4.48 (4.12, 4.86) | 15.06 (13.8, 16.41) |
| 13 | 39.75 (37.52, 42.06) | 2.95 (2.71, 3.21) | 32.66 (30.76, 34.63) |
| 14 | 48.9 (46.75, 51.11) | 5.05 (4.72, 5.4) | 22.35 (21.26, 23.49) |
| 15 | 53.79 (52, 55.62) | 10.86 (10.46, 11.26) | 32.17 (31.05, 33.33) |
| 16 | 35.83 (34.71, 36.96) | 4.45 (4.31, 4.59) | 24.42 (23.72, 25.13) |
| 17 | 40.86 (39.75, 42) | 3.67 (3.56, 3.78) | 19.4 (18.88, 19.93) |
| 18 | 52.38 (51.26, 53.51) | 6.03 (5.89, 6.17) | 27.63 (27.06, 28.22) |
| 19 | 56.01 (55.09, 56.93) | 6.28 (6.16, 6.39) | 29.78 (29.31, 30.26) |
| 20 | 45.64 (45, 46.28) | 4.89 (4.82, 4.97) | 23.03 (22.71, 23.36) |
| 21 | 49.8 (49.22, 50.39) | 5.05 (4.98, 5.11) | 23.47 (23.18, 23.76) |
| 22 | 47.96 (47.48, 48.44) | 5.17 (5.11, 5.23) | 25.35 (25.09, 25.61) |
| 23 | 49.58 (49.16, 50.01) | 4.88 (4.84, 4.93) | 25.14 (24.92, 25.37) |
| 24 | 50.19 (49.82, 50.56) | 4.25 (4.21, 4.29) | 23.43 (23.25, 23.62) |
| 25 | 46.05 (45.74, 46.36) | 4.58 (4.54, 4.62) | 21.98 (21.82, 22.14) |
| 26 | 45.11 (44.83, 45.39) | 4.13 (4.09, 4.16) | 22.08 (21.93, 22.23) |
| 27 | 36.34 (36.1, 36.58) | 3.13 (3.1, 3.16) | 17.28 (17.15, 17.4) |
| 28 | 39.01 (38.75, 39.27) | 3.58 (3.54, 3.62) | 19.31 (19.17, 19.45) |
| 29 | 36.34 (36.08, 36.59) | 3.4 (3.37, 3.44) | 17.39 (17.26, 17.53) |
| 30 | 33.16 (32.89, 33.42) | 3.11 (3.07, 3.15) | 15.39 (15.26, 15.52) |
| 31 | 32.55 (32.27, 32.84) | 2.94 (2.9, 2.98) | 15.42 (15.27, 15.56) |
| 32 | 31.49 (31.19, 31.81) | 3.13 (3.09, 3.18) | 14.86 (14.7, 15.03) |
| 33 | 29.13 (28.8, 29.47) | 3.43 (3.38, 3.49) | 15.71 (15.52, 15.9) |
| 34 | 26.59 (26.22, 26.95) | 3.81 (3.75, 3.87) | 16.09 (15.88, 16.29) |
| 35 | 30.52 (30.06, 30.99) | 3.37 (3.32, 3.43) | 13.91 (13.71, 14.12) |
| 36 | 31.36 (30.84, 31.88) | 3.19 (3.14, 3.25) | 16.81 (16.55, 17.07) |
| 37 | 34.37 (33.78, 34.96) | 3.87 (3.8, 3.93) | 17.28 (17.01, 17.55) |
| 38 | 26.51 (25.98, 27.05) | 2.69 (2.63, 2.74) | 14.57 (14.31, 14.83) |
| 39 | 30.32 (29.65, 30.99) | 3.52 (3.45, 3.6) | 20.25 (19.92, 20.59) |
| 40 | 27.7 (27.02, 28.4) | 3.09 (3.03, 3.16) | 13.14 (12.89, 13.4) |
| 41 | 49.71 (48.68, 50.76) | 4.33 (4.24, 4.42) | 21.89 (21.51, 22.28) |
| 42 | 38.38 (37.61, 39.15) | 3.41 (3.34, 3.49) | 16.52 (16.22, 16.83) |
| 43 | 36.26 (35.54, 36.99) | 4.04 (3.96, 4.12) | 20.02 (19.67, 20.37) |
| 44 | 30.87 (30.21, 31.54) | 3.02 (2.95, 3.09) | 16.77 (16.47, 17.08) |
| 45 | 30.29 (29.59, 31.01) | 3.29 (3.21, 3.37) | 15.1 (14.8, 15.41) |
| 46 | 30.09 (29.33, 30.86) | 2.71 (2.63, 2.78) | 15.55 (15.22, 15.89) |
| 47 | 24.66 (23.92, 25.42) | 2.69 (2.6, 2.77) | 5.87 (5.65, 6.09) |
| 48 | 20.7 (19.88, 21.55) | 2.79 (2.69, 2.89) | 16.03 (15.42, 16.66) |
| 49 | 26.03 (24.82, 27.27) | 2.79 (2.68, 2.9) | 18.56 (17.88, 19.26) |
| 50 | 27.78 (26.34, 29.27) | 3.31 (3.17, 3.45) | 9.94 (9.46, 10.43) |
| 51 | 14.82 (13.66, 16.04) | 0.81 (0.74, 0.88) | 14.72 (13.96, 15.5) |
| 52 | 21.5 (19.51, 23.63) | 2.6 (2.35, 2.87) | 8.12 (7.52, 8.75) |

|  | Shanghai | Jiangsu | Zhejiang | Anhui | Fujian | Jiangxi | Shandong |
| --- | --- | --- | --- | --- | --- | --- | --- |
| 1 | 13.86 (13.45, 14.28) | 18.56 (18.3, 18.82) | 19.91 (19.56, 20.27) | 21.02 (20.71, 21.33) | 21.51 (21.06, 21.96) | 15.1 (14.83, 15.38) | 5.21 (5.09, 5.33) |
| 2 | 13.11 (12.65, 13.57) | 16.96 (16.68, 17.25) | 20.78 (20.36, 21.2) | 21.07 (20.73, 21.42) | 22.33 (21.82, 22.85) | 15.77 (15.46, 16.08) | 5.1 (4.98, 5.23) |
| 3 | 15.43 (14.84, 16.03) | 18.64 (18.29, 19) | 22.64 (22.15, 23.13) | 24.73 (24.32, 25.14) | 22.48 (21.92, 23.05) | 15.96 (15.62, 16.3) | 4.68 (4.54, 4.82) |
| 4 | 11.97 (11.42, 12.54) | 17.04 (16.67, 17.42) | 21.28 (20.78, 21.79) | 23.21 (22.81, 23.62) | 23.98 (23.35, 24.62) | 17.19 (16.81, 17.57) | 5.69 (5.51, 5.87) |
| 5 | 15.86 (15.09, 16.65) | 19.93 (19.46, 20.41) | 19 (18.48, 19.53) | 26.98 (26.52, 27.44) | 21.53 (20.91, 22.16) | 14.7 (14.34, 15.06) | 5.96 (5.76, 6.16) |
| 6 | 23.93 (22.93, 24.95) | 25.16 (24.6, 25.74) | 30.36 (29.6, 31.13) | 27.12 (26.67, 27.57) | 30.22 (29.42, 31.04) | 27.72 (27.17, 28.27) | 8.96 (8.71, 9.21) |
| 7 | 25.29 (24.42, 26.19) | 30.6 (30.02, 31.2) | 29.61 (28.93, 30.3) | 33.07 (32.59, 33.56) | 32.23 (31.46, 33.01) | 20.62 (20.23, 21) | 9.12 (8.91, 9.33) |
| 8 | 31.27 (30.45, 32.1) | 36.94 (36.38, 37.51) | 36.36 (35.67, 37.07) | 40.89 (40.42, 41.37) | 37.93 (37.18, 38.69) | 25.57 (25.16, 25.97) | 11.71 (11.5, 11.91) |
| 9 | 25.59 (25.03, 26.17) | 34.4 (33.97, 34.83) | 40.96 (40.34, 41.59) | 33.66 (33.32, 34.01) | 34.61 (34.01, 35.21) | 27.61 (27.25, 27.97) | 8.85 (8.72, 8.99) |
| 10 | 19.81 (19.38, 20.24) | 27.05 (26.73, 27.37) | 29.94 (29.52, 30.37) | 29.29 (29.01, 29.58) | 29.96 (29.47, 30.46) | 22.05 (21.78, 22.31) | 8.73 (8.61, 8.85) |
| 11 | 21.96 (21.53, 22.4) | 29 (28.7, 29.32) | 34.51 (34.08, 34.94) | 35.45 (35.15, 35.75) | 33.42 (32.93, 33.91) | 26.37 (26.1, 26.64) | 9.69 (9.58, 9.8) |
| 12 | 23.83 (23.41, 24.25) | 32.64 (32.34, 32.94) | 36.76 (36.38, 37.16) | 32.63 (32.38, 32.89) | 34.74 (34.29, 35.19) | 24.81 (24.59, 25.04) | 8.31 (8.22, 8.4) |
| 13 | 21.53 (21.18, 21.89) | 29.02 (28.77, 29.26) | 32.4 (32.08, 32.71) | 34.51 (34.27, 34.74) | 29.78 (29.41, 30.16) | 22.52 (22.33, 22.71) | 8.88 (8.79, 8.96) |
| 14 | 22.51 (22.17, 22.86) | 27.97 (27.75, 28.19) | 32.31 (32.02, 32.6) | 30.84 (30.64, 31.04) | 32.99 (32.61, 33.37) | 26.64 (26.45, 26.84) | 9.49 (9.41, 9.57) |
| 15 | 25.87 (25.53, 26.22) | 32.55 (32.33, 32.78) | 41.76 (41.46, 42.07) | 36.92 (36.71, 37.13) | 44.39 (43.99, 44.8) | 30.8 (30.62, 30.98) | 9.92 (9.85, 9.99) |
| 16 | 22.03 (21.76, 22.31) | 29.26 (29.07, 29.45) | 32.88 (32.66, 33.11) | 30.67 (30.5, 30.84) | 32.76 (32.48, 33.04) | 22.99 (22.86, 23.12) | 10.12 (10.06, 10.18) |
| 17 | 18.85 (18.61, 19.1) | 26.24 (26.08, 26.41) | 29.38 (29.18, 29.58) | 28.81 (28.66, 28.97) | 30.2 (29.94, 30.45) | 19.19 (19.07, 19.3) | 9.16 (9.11, 9.21) |
| 18 | 23.71 (23.43, 23.99) | 32.76 (32.57, 32.94) | 33.71 (33.49, 33.92) | 31.74 (31.57, 31.91) | 34.63 (34.36, 34.91) | 22.49 (22.36, 22.62) | 10.07 (10.02, 10.11) |
| 19 | 23.35 (23.1, 23.61) | 28.55 (28.4, 28.71) | 31.53 (31.34, 31.73) | 29.92 (29.77, 30.08) | 37.06 (36.79, 37.32) | 19.07 (18.95, 19.18) | 9.18 (9.14, 9.22) |
| 20 | 21.34 (21.1, 21.57) | 26.55 (26.41, 26.69) | 30.8 (30.61, 30.99) | 29.14 (28.98, 29.29) | 32.74 (32.52, 32.97) | 19.03 (18.91, 19.15) | 9.31 (9.27, 9.35) |
| 21 | 20.11 (19.88, 20.33) | 26.08 (25.94, 26.23) | 27.6 (27.42, 27.78) | 28.15 (28, 28.31) | 29.36 (29.15, 29.57) | 17.43 (17.31, 17.55) | 9.09 (9.05, 9.12) |
| 22 | 21.18 (20.95, 21.42) | 26.19 (26.05, 26.34) | 31.74 (31.54, 31.94) | 26.5 (26.35, 26.65) | 32.52 (32.3, 32.74) | 20.44 (20.3, 20.58) | 7.98 (7.95, 8.01) |
| 23 | 24.62 (24.37, 24.87) | 30.48 (30.33, 30.64) | 35.02 (34.82, 35.23) | 32.49 (32.31, 32.67) | 33.69 (33.47, 33.91) | 21.77 (21.62, 21.92) | 8.54 (8.51, 8.57) |
| 24 | 22.42 (22.2, 22.64) | 26.38 (26.24, 26.52) | 31.79 (31.6, 31.97) | 29.29 (29.12, 29.46) | 30.34 (30.13, 30.55) | 19.5 (19.37, 19.64) | 7.54 (7.51, 7.57) |
| 25 | 21.59 (21.38, 21.8) | 26.49 (26.35, 26.63) | 32.75 (32.56, 32.93) | 27.14 (26.98, 27.3) | 28.75 (28.54, 28.96) | 20.04 (19.9, 20.19) | 7.98 (7.95, 8.01) |
| 26 | 17.66 (17.47, 17.85) | 22.56 (22.43, 22.69) | 25.34 (25.18, 25.51) | 25.29 (25.12, 25.45) | 25.73 (25.52, 25.93) | 16.02 (15.88, 16.15) | 7.88 (7.85, 7.91) |
| 27 | 14.97 (14.78, 15.17) | 17.63 (17.5, 17.76) | 20.36 (20.2, 20.52) | 20.3 (20.14, 20.46) | 23.01 (22.79, 23.22) | 15.35 (15.2, 15.49) | 6.03 (6, 6.05) |
| 28 | 16.35 (16.12, 16.59) | 20.75 (20.59, 20.92) | 23.08 (22.87, 23.28) | 25.14 (24.93, 25.35) | 24.91 (24.65, 25.16) | 19.1 (18.91, 19.29) | 7.04 (7.01, 7.07) |
| 29 | 14.96 (14.71, 15.21) | 19.56 (19.38, 19.74) | 23.35 (23.11, 23.58) | 23.92 (23.7, 24.14) | 27.7 (27.4, 28) | 17.07 (16.89, 17.26) | 6.89 (6.86, 6.93) |
| 30 | 13.25 (12.98, 13.53) | 18.82 (18.61, 19.03) | 23.29 (23.02, 23.56) | 22.8 (22.57, 23.04) | 27.7 (27.39, 28.01) | 16.52 (16.33, 16.72) | 6.39 (6.36, 6.43) |
| 31 | 16.41 (16.05, 16.79) | 20.61 (20.36, 20.86) | 23.91 (23.61, 24.21) | 24.24 (23.97, 24.51) | 24.05 (23.75, 24.35) | 19.41 (19.18, 19.64) | 6.24 (6.2, 6.28) |
| 32 | 16.21 (15.81, 16.61) | 20.48 (20.2, 20.75) | 22.95 (22.62, 23.27) | 22.59 (22.31, 22.87) | 27.03 (26.68, 27.39) | 16.99 (16.77, 17.21) | 5.86 (5.82, 5.91) |
| 33 | 18.26 (17.8, 18.72) | 22.76 (22.44, 23.07) | 26.11 (25.73, 26.49) | 24.83 (24.51, 25.16) | 27.32 (26.96, 27.69) | 17.25 (17.01, 17.49) | 5.63 (5.59, 5.68) |
| 34 | 20.16 (19.67, 20.65) | 26.44 (26.08, 26.79) | 28.56 (28.15, 28.98) | 27.92 (27.56, 28.28) | 31.22 (30.82, 31.63) | 23.66 (23.36, 23.95) | 6.06 (6, 6.11) |
| 35 | 18.09 (17.64, 18.55) | 24.53 (24.21, 24.86) | 26.3 (25.91, 26.69) | 24.06 (23.73, 24.39) | 30.44 (30.05, 30.82) | 19.88 (19.63, 20.13) | 5.68 (5.63, 5.74) |
| 36 | 24.69 (24.15, 25.23) | 32.96 (32.59, 33.34) | 36.78 (36.3, 37.25) | 33.2 (32.79, 33.61) | 38.39 (37.97, 38.82) | 24.96 (24.68, 25.24) | 6.89 (6.82, 6.97) |
| 37 | 20.69 (20.26, 21.13) | 27.29 (26.99, 27.59) | 28.09 (27.73, 28.46) | 31.97 (31.61, 32.33) | 31.25 (30.91, 31.59) | 19.92 (19.7, 20.14) | 6.97 (6.9, 7.05) |
| 38 | 15.45 (15.09, 15.81) | 20.42 (20.18, 20.67) | 26.21 (25.87, 26.57) | 20.57 (20.3, 20.83) | 28.77 (28.45, 29.09) | 15.93 (15.73, 16.13) | 5.48 (5.41, 5.55) |
| 39 | 17.93 (17.51, 18.37) | 25.49 (25.19, 25.79) | 26.73 (26.37, 27.09) | 29.8 (29.43, 30.17) | 26.88 (26.56, 27.19) | 19.47 (19.23, 19.71) | 6.96 (6.87, 7.04) |
| 40 | 15.82 (15.41, 16.24) | 19.8 (19.54, 20.06) | 20.88 (20.55, 21.2) | 20.7 (20.41, 20.99) | 20.5 (20.21, 20.79) | 16.98 (16.75, 17.2) | 5.68 (5.6, 5.76) |
| 41 | 23.9 (23.35, 24.45) | 32.59 (32.22, 32.96) | 33.58 (33.11, 34.05) | 33.63 (33.21, 34.05) | 31.92 (31.49, 32.35) | 22.19 (21.92, 22.47) | 8.35 (8.24, 8.46) |
| 42 | 20.13 (19.68, 20.58) | 26.17 (25.89, 26.46) | 25.44 (25.07, 25.81) | 28.42 (28.07, 28.76) | 25.21 (24.85, 25.57) | 18.02 (17.79, 18.26) | 8.01 (7.91, 8.1) |
| 43 | 20.44 (20, 20.88) | 26.66 (26.38, 26.94) | 30.17 (29.75, 30.59) | 26.97 (26.65, 27.3) | 29.32 (28.9, 29.74) | 22.42 (22.15, 22.7) | 6.87 (6.79, 6.96) |
| 44 | 23.66 (23.21, 24.13) | 28.36 (28.08, 28.64) | 31.84 (31.43, 32.25) | 31.92 (31.57, 32.28) | 28.85 (28.44, 29.26) | 19.71 (19.47, 19.95) | 7.98 (7.88, 8.07) |
| 45 | 19.71 (19.34, 20.09) | 25.71 (25.46, 25.96) | 28.57 (28.21, 28.94) | 31.36 (31.03, 31.68) | 31.07 (30.65, 31.5) | 21.29 (21.04, 21.54) | 7.05 (6.97, 7.13) |
| 46 | 22.19 (21.79, 22.59) | 27.77 (27.51, 28.03) | 30.84 (30.47, 31.21) | 29.57 (29.27, 29.86) | 29.72 (29.32, 30.12) | 19.92 (19.69, 20.15) | 6.86 (6.78, 6.94) |
| 47 | 17.14 (16.81, 17.47) | 22.88 (22.66, 23.1) | 26.13 (25.81, 26.46) | 27.05 (26.78, 27.32) | 26.18 (25.81, 26.55) | 17.53 (17.31, 17.74) | 6.41 (6.33, 6.49) |
| 48 | 19.7 (19.33, 20.08) | 25.09 (24.85, 25.34) | 27.05 (26.71, 27.39) | 27.49 (27.21, 27.76) | 28.14 (27.74, 28.54) | 18.22 (17.99, 18.45) | 6.14 (6.05, 6.22) |
| 49 | 19.16 (18.79, 19.53) | 24.66 (24.42, 24.91) | 26.19 (25.86, 26.53) | 25.64 (25.37, 25.9) | 25.82 (25.43, 26.21) | 17.8 (17.56, 18.03) | 6.18 (6.09, 6.27) |
| 50 | 16.38 (16.04, 16.73) | 21.51 (21.29, 21.74) | 23.9 (23.57, 24.24) | 24.26 (23.99, 24.52) | 25.88 (25.48, 26.29) | 16.83 (16.59, 17.07) | 5.54 (5.45, 5.63) |
| 51 | 16.29 (15.92, 16.66) | 20.83 (20.59, 21.07) | 24.41 (24.05, 24.76) | 22.94 (22.66, 23.21) | 27.75 (27.31, 28.19) | 17.1 (16.85, 17.36) | 5.97 (5.87, 6.08) |
| 52 | 13.11 (12.75, 13.47) | 18.83 (18.58, 19.08) | 20 (19.66, 20.34) | 20.7 (20.42, 20.98) | 18.48 (18.12, 18.84) | 14.24 (14, 14.49) | 4.88 (4.78, 4.98) |

|  | Henan | Hubei | Hunan | Guangdong | Guangxi | Hainan |
| --- | --- | --- | --- | --- | --- | --- |
| 1 | 7.27 (7.17, 7.37) | 9.15 (9.01, 9.29) | 20 (19.61, 20.38) | 40.93 (40.32, 41.54) | 22.2 (21.69, 22.71) | 8.95 (8.63, 9.28) |
| 2 | 6.6 (6.5, 6.71) | 8.78 (8.62, 8.93) | 17.75 (17.35, 18.16) | 31.33 (30.8, 31.86) | 20.11 (19.61, 20.62) | 10.57 (10.18, 10.96) |
| 3 | 7.79 (7.67, 7.92) | 8.84 (8.67, 9.02) | 21.84 (21.32, 22.37) | 49.6 (48.86, 50.35) | 31.26 (30.57, 31.95) | 13.29 (12.85, 13.73) |
| 4 | 9.08 (8.94, 9.23) | 7.69 (7.51, 7.87) | 21.13 (20.59, 21.68) | 36.61 (36.05, 37.19) | 22.85 (22.34, 23.37) | 12.64 (12.26, 13.03) |
| 5 | 8.14 (8.01, 8.27) | 9.82 (9.57, 10.06) | 17.28 (16.77, 17.81) | 33.71 (33.15, 34.28) | 19.45 (18.97, 19.94) | 10.45 (10.13, 10.78) |
| 6 | 10.05 (9.9, 10.2) | 12.74 (12.45, 13.03) | 28.12 (27.35, 28.9) | 41.24 (40.58, 41.91) | 25.33 (24.72, 25.94) | 10.08 (9.75, 10.41) |
| 7 | 11.31 (11.17, 11.46) | 13.68 (13.4, 13.97) | 26.62 (25.94, 27.31) | 44.45 (43.79, 45.13) | 26.07 (25.48, 26.67) | 13.19 (12.8, 13.58) |
| 8 | 17.8 (17.64, 17.96) | 18.81 (18.52, 19.11) | 34.09 (33.37, 34.82) | 61.4 (60.67, 62.13) | 31.06 (30.45, 31.68) | 14.43 (14.06, 14.81) |
| 9 | 12.22 (12.12, 12.31) | 17.17 (16.95, 17.39) | 33.49 (32.89, 34.09) | 54.41 (53.86, 54.96) | 35.47 (34.9, 36.04) | 13.71 (13.39, 14.03) |
| 10 | 10.05 (9.98, 10.13) | 12.64 (12.49, 12.8) | 28.3 (27.84, 28.77) | 47.02 (46.59, 47.46) | 29.59 (29.16, 30.03) | 13.86 (13.57, 14.16) |
| 11 | 14.42 (14.33, 14.5) | 19.31 (19.13, 19.5) | 41.3 (40.78, 41.82) | 49.55 (49.14, 49.97) | 34.42 (33.99, 34.84) | 15.41 (15.13, 15.69) |
| 12 | 11.44 (11.38, 11.5) | 14.46 (14.33, 14.58) | 33.09 (32.73, 33.45) | 52.3 (51.91, 52.68) | 34.83 (34.47, 35.19) | 12.1 (11.89, 12.32) |
| 13 | 11.74 (11.69, 11.8) | 15.5 (15.38, 15.62) | 36.81 (36.48, 37.14) | 45.69 (45.37, 46.01) | 39.89 (39.57, 40.22) | 11.08 (10.87, 11.28) |
| 14 | 12.22 (12.17, 12.27) | 15.22 (15.12, 15.33) | 41.92 (41.63, 42.21) | 59.59 (59.25, 59.94) | 44.53 (44.25, 44.81) | 12.56 (12.34, 12.78) |
| 15 | 12.22 (12.17, 12.27) | 18.08 (17.97, 18.18) | 44.47 (44.23, 44.71) | 59.71 (59.41, 60) | 37.75 (37.55, 37.95) | 12.78 (12.56, 12.99) |
| 16 | 12.26 (12.22, 12.3) | 13.75 (13.68, 13.83) | 31.61 (31.46, 31.77) | 48.2 (47.97, 48.43) | 30.82 (30.66, 30.98) | 11.02 (10.83, 11.21) |
| 17 | 9.26 (9.22, 9.29) | 11.82 (11.75, 11.89) | 29.67 (29.52, 29.81) | 47.46 (47.24, 47.68) | 28.23 (28.08, 28.37) | 11.62 (11.42, 11.83) |
| 18 | 10.33 (10.29, 10.37) | 13.77 (13.69, 13.84) | 31.85 (31.71, 32) | 54 (53.76, 54.23) | 31.16 (31.01, 31.32) | 10.78 (10.59, 10.97) |
| 19 | 9.95 (9.91, 9.98) | 13.33 (13.26, 13.4) | 31.76 (31.63, 31.9) | 51.22 (51.01, 51.43) | 29.34 (29.18, 29.49) | 11.46 (11.25, 11.67) |
| 20 | 9.25 (9.22, 9.29) | 11.6 (11.54, 11.67) | 25.66 (25.54, 25.77) | 43.41 (43.22, 43.6) | 26.8 (26.65, 26.95) | 10.51 (10.31, 10.71) |
| 21 | 9.28 (9.24, 9.32) | 11.94 (11.87, 12.01) | 26.95 (26.82, 27.08) | 41.25 (41.05, 41.44) | 28.2 (28.04, 28.37) | 10.93 (10.72, 11.14) |
| 22 | 8.59 (8.55, 8.63) | 12.17 (12.09, 12.24) | 28.82 (28.68, 28.96) | 46.99 (46.76, 47.22) | 29.78 (29.6, 29.96) | 11.21 (10.99, 11.43) |
| 23 | 7.99 (7.95, 8.03) | 11.11 (11.04, 11.19) | 27.49 (27.35, 27.62) | 46.13 (45.9, 46.37) | 29.56 (29.38, 29.76) | 11.09 (10.87, 11.31) |
| 24 | 8.19 (8.14, 8.24) | 10.91 (10.83, 10.98) | 27.63 (27.49, 27.77) | 49.18 (48.93, 49.43) | 28.64 (28.45, 28.84) | 12.08 (11.85, 12.32) |
| 25 | 9.32 (9.27, 9.38) | 11.87 (11.78, 11.95) | 27.74 (27.6, 27.89) | 49.03 (48.78, 49.29) | 29.87 (29.65, 30.08) | 12.44 (12.22, 12.68) |
| 26 | 9.43 (9.38, 9.49) | 10.45 (10.37, 10.54) | 23.95 (23.8, 24.09) | 46.37 (46.13, 46.62) | 27.35 (27.14, 27.56) | 10.78 (10.57, 10.99) |
| 27 | 7.72 (7.66, 7.77) | 8.47 (8.39, 8.55) | 19.91 (19.76, 20.05) | 43.69 (43.44, 43.95) | 24.01 (23.79, 24.22) | 10.22 (10.01, 10.42) |
| 28 | 8.11 (8.05, 8.17) | 9.57 (9.47, 9.67) | 22.73 (22.55, 22.92) | 41.78 (41.52, 42.05) | 24.18 (23.94, 24.42) | 10.8 (10.58, 11.02) |
| 29 | 8.49 (8.42, 8.55) | 9.81 (9.69, 9.93) | 24.04 (23.83, 24.25) | 43.61 (43.31, 43.91) | 26.22 (25.94, 26.5) | 10.94 (10.71, 11.17) |
| 30 | 7.96 (7.89, 8.02) | 8.04 (7.93, 8.16) | 22.48 (22.26, 22.7) | 44.8 (44.48, 45.12) | 27.31 (27, 27.61) | 11.37 (11.13, 11.61) |
| 31 | 7.46 (7.39, 7.53) | 8.33 (8.18, 8.47) | 19.84 (19.61, 20.08) | 36.31 (36.01, 36.61) | 22.42 (22.13, 22.7) | 8.29 (8.09, 8.5) |
| 32 | 8.21 (8.13, 8.3) | 9.84 (9.66, 10.03) | 25.13 (24.82, 25.44) | 46.47 (46.08, 46.86) | 27.11 (26.76, 27.47) | 10.91 (10.64, 11.19) |
| 33 | 7.63 (7.54, 7.71) | 9.67 (9.47, 9.87) | 24.76 (24.45, 25.08) | 48.53 (48.13, 48.94) | 26.58 (26.22, 26.95) | 10.69 (10.41, 10.97) |
| 34 | 8.26 (8.16, 8.35) | 11.91 (11.67, 12.14) | 30.06 (29.7, 30.42) | 44.88 (44.5, 45.26) | 29.88 (29.48, 30.28) | 11.31 (11.02, 11.6) |
| 35 | 10.88 (10.76, 10.99) | 14.21 (13.96, 14.46) | 37.1 (36.72, 37.48) | 46.96 (46.56, 47.35) | 27.63 (27.26, 28) | 11.29 (11, 11.58) |
| 36 | 9.95 (9.85, 10.05) | 15.99 (15.75, 16.23) | 33.21 (32.9, 33.52) | 60.12 (59.68, 60.57) | 35.4 (34.99, 35.82) | 16.25 (15.91, 16.6) |
| 37 | 9.88 (9.78, 9.98) | 15.15 (14.95, 15.35) | 26.03 (25.78, 26.28) | 49.57 (49.22, 49.93) | 27.95 (27.63, 28.28) | 14.41 (14.14, 14.68) |
| 38 | 7.16 (7.08, 7.24) | 10.78 (10.64, 10.93) | 25.58 (25.33, 25.84) | 44.94 (44.61, 45.27) | 26.43 (26.12, 26.75) | 12.48 (12.26, 12.71) |
| 39 | 9.48 (9.38, 9.59) | 11.66 (11.5, 11.82) | 29.03 (28.76, 29.31) | 40.03 (39.71, 40.34) | 23.97 (23.67, 24.29) | 10.31 (10.12, 10.51) |
| 40 | 6.9 (6.82, 6.99) | 9.7 (9.55, 9.84) | 23.57 (23.33, 23.81) | 36.54 (36.22, 36.87) | 24.81 (24.48, 25.15) | 8.79 (8.6, 8.98) |
| 41 | 10.7 (10.57, 10.82) | 16.14 (15.94, 16.35) | 30.58 (30.29, 30.88) | 47.31 (46.91, 47.73) | 30.59 (30.2, 30.98) | 12.84 (12.59, 13.1) |
| 42 | 10.13 (10.02, 10.24) | 12.91 (12.76, 13.07) | 27.79 (27.52, 28.05) | 37.03 (36.68, 37.39) | 25.11 (24.78, 25.45) | 11.13 (10.9, 11.35) |
| 43 | 8.91 (8.81, 9.01) | 12.78 (12.63, 12.93) | 27.64 (27.38, 27.91) | 40.17 (39.77, 40.58) | 23.43 (23.1, 23.77) | 9.66 (9.45, 9.87) |
| 44 | 10.41 (10.3, 10.51) | 14.07 (13.92, 14.22) | 27.98 (27.72, 28.24) | 42.44 (42, 42.88) | 29.85 (29.45, 30.26) | 11.21 (10.97, 11.46) |
| 45 | 9.9 (9.81, 10) | 13.57 (13.43, 13.71) | 27.8 (27.54, 28.06) | 46.16 (45.69, 46.62) | 26.77 (26.41, 27.13) | 11.02 (10.78, 11.26) |
| 46 | 9.56 (9.47, 9.65) | 12.92 (12.8, 13.05) | 27.63 (27.38, 27.88) | 43.87 (43.43, 44.31) | 27.04 (26.68, 27.41) | 11.22 (10.97, 11.47) |
| 47 | 8.71 (8.63, 8.8) | 11.45 (11.34, 11.57) | 24.55 (24.32, 24.79) | 38.36 (37.94, 38.77) | 24.34 (24, 24.69) | 9.95 (9.71, 10.18) |
| 48 | 8.54 (8.45, 8.63) | 11.82 (11.7, 11.94) | 23.79 (23.55, 24.04) | 37.89 (37.45, 38.33) | 23.35 (23.01, 23.7) | 9 (8.77, 9.24) |
| 49 | 8.78 (8.69, 8.87) | 10.63 (10.52, 10.75) | 23 (22.75, 23.25) | 38.14 (37.67, 38.6) | 22.53 (22.17, 22.89) | 9.56 (9.29, 9.83) |
| 50 | 8.03 (7.95, 8.12) | 9.91 (9.79, 10.03) | 21.76 (21.5, 22.02) | 35.8 (35.33, 36.28) | 23.48 (23.09, 23.87) | 8.94 (8.66, 9.22) |
| 51 | 7.2 (7.11, 7.29) | 9.97 (9.84, 10.1) | 19.26 (19, 19.53) | 35.79 (35.28, 36.3) | 21.85 (21.46, 22.24) | 9.51 (9.19, 9.83) |
| 52 | 6.7 (6.61, 6.8) | 8.36 (8.23, 8.49) | 14.96 (14.69, 15.23) | 23.61 (23.17, 24.06) | 14.26 (13.93, 14.6) | 7.58 (7.28, 7.88) |

|  | Chongqing | Sichuan | Guizhou | Yunnan | Tibet |
| --- | --- | --- | --- | --- | --- |
| 1 | 65.61 (64.16, 67.08) | 39.7 (39.26, 40.15) | 21.01 (20.51, 21.53) | 58.9 (58.12, 59.69) | 10.08 (8.52, 11.81) |
| 2 | 53.54 (52.13, 54.98) | 29.84 (29.45, 30.23) | 20.74 (20.19, 21.31) | 52.99 (52.25, 53.74) | 13.74 (10.96, 16.96) |
| 3 | 64.36 (62.55, 66.21) | 38.47 (37.97, 38.98) | 22.34 (21.7, 23) | 36.35 (35.72, 37) | 77.39 (68.48, 87.05) |
| 4 | 66.62 (64.65, 68.64) | 28.64 (28.2, 29.09) | 30.36 (29.56, 31.16) | 49.98 (49.06, 50.92) | 8.92 (7.21, 10.88) |
| 5 | 57.51 (55.59, 59.48) | 31.28 (30.74, 31.83) | 26.39 (25.71, 27.08) | 46.64 (45.7, 47.59) | 10.26 (7.11, 14.24) |
| 6 | 75.02 (72.58, 77.52) | 47.34 (46.61, 48.08) | 26.86 (26.18, 27.55) | 53.03 (51.95, 54.12) | 90.3 (74.85, 107.74) |
| 7 | 83.17 (80.66, 85.72) | 38.9 (38.31, 39.5) | 29.59 (28.9, 30.3) | 58.95 (57.81, 60.11) | 68.88 (61.34, 77.02) |
| 8 | 106.49 (103.88, 109.14) | 50 (49.33, 50.67) | 35.31 (34.61, 36.03) | 65.86 (64.71, 67.03) | 20.79 (18.21, 23.6) |
| 9 | 107.22 (105.07, 109.4) | 59.75 (59.11, 60.39) | 37.83 (37.2, 38.46) | 65.82 (64.78, 66.88) | 31.56 (28.09, 35.31) |
| 10 | 91.82 (90.17, 93.48) | 44.69 (44.24, 45.14) | 39.75 (39.21, 40.3) | 72.56 (71.56, 73.57) | 36.36 (32.99, 39.96) |
| 11 | 90.01 (88.55, 91.49) | 47.21 (46.78, 47.65) | 32.56 (32.15, 32.97) | 66.74 (65.91, 67.58) | 72.91 (68.84, 77.15) |
| 12 | 97.65 (96.26, 99.04) | 52.99 (52.57, 53.42) | 42.66 (42.23, 43.09) | 84.34 (83.5, 85.2) | 76.4 (73.85, 79.01) |
| 13 | 83.66 (82.54, 84.8) | 47.43 (47.08, 47.78) | 35.93 (35.61, 36.25) | 67.31 (66.69, 67.93) | 19.82 (19.02, 20.64) |
| 14 | 109.64 (108.42, 110.87) | 54.9 (54.55, 55.26) | 34.66 (34.37, 34.94) | 67.87 (67.3, 68.44) | 32.26 (31.05, 33.49) |
| 15 | 102.01 (101.02, 103.02) | 51.42 (51.12, 51.72) | 38.12 (37.85, 38.39) | 68.77 (68.24, 69.31) | 62.96 (61.41, 64.54) |
| 16 | 89.23 (88.4, 90.06) | 47.26 (47, 47.53) | 35.43 (35.21, 35.66) | 75.86 (75.35, 76.38) | 20.57 (19.97, 21.18) |
| 17 | 76.63 (75.9, 77.38) | 38.48 (38.25, 38.71) | 28.25 (28.06, 28.43) | 66.02 (65.6, 66.45) | 31 (30.13, 31.89) |
| 18 | 91.82 (90.98, 92.66) | 49.25 (48.97, 49.52) | 31.57 (31.36, 31.77) | 64.94 (64.53, 65.35) | 28.39 (27.58, 29.21) |
| 19 | 114.45 (113.55, 115.34) | 49.37 (49.11, 49.63) | 32.63 (32.43, 32.83) | 75.11 (74.68, 75.54) | 31.09 (30.24, 31.96) |
| 20 | 80.42 (79.77, 81.07) | 42.66 (42.43, 42.89) | 26.22 (26.05, 26.39) | 63.03 (62.67, 63.39) | 31.4 (30.57, 32.24) |
| 21 | 79.5 (78.83, 80.17) | 40.81 (40.58, 41.04) | 24.04 (23.87, 24.22) | 65.88 (65.51, 66.25) | 29.11 (28.33, 29.89) |
| 22 | 81.21 (80.5, 81.93) | 44.72 (44.47, 44.96) | 27.47 (27.26, 27.68) | 65.72 (65.34, 66.09) | 42.44 (41.49, 43.39) |
| 23 | 91.87 (91.08, 92.66) | 43.78 (43.54, 44.02) | 30.08 (29.85, 30.31) | 61.38 (61.02, 61.74) | 33.82 (33.11, 34.54) |
| 24 | 83.09 (82.35, 83.83) | 43.88 (43.64, 44.12) | 26.02 (25.82, 26.23) | 56.96 (56.59, 57.32) | 18.66 (18.15, 19.18) |
| 25 | 80.4 (79.63, 81.17) | 44.25 (44, 44.49) | 26.36 (26.14, 26.59) | 61.07 (60.65, 61.48) | 43.43 (42.44, 44.45) |
| 26 | 70.47 (69.71, 71.24) | 38 (37.77, 38.22) | 26.86 (26.62, 27.09) | 61.57 (61.13, 62) | 38.08 (37.29, 38.89) |
| 27 | 70.91 (70.05, 71.78) | 37.28 (37.04, 37.52) | 26.42 (26.17, 26.67) | 61.03 (60.57, 61.48) | 29.92 (29.28, 30.58) |
| 28 | 63.01 (62.11, 63.92) | 32.55 (32.31, 32.8) | 21.43 (21.2, 21.67) | 51.16 (50.72, 51.6) | 30.13 (29.46, 30.81) |
| 29 | 68.1 (67.01, 69.2) | 35.32 (35.03, 35.62) | 26.29 (25.99, 26.59) | 56.01 (55.49, 56.54) | 24.24 (23.62, 24.87) |
| 30 | 68.77 (67.56, 69.99) | 35.36 (35.03, 35.68) | 22.45 (22.16, 22.74) | 57.04 (56.47, 57.62) | 22.71 (22.01, 23.43) |
| 31 | 68.65 (67.33, 69.98) | 30.72 (30.4, 31.05) | 20.33 (20.02, 20.64) | 50 (49.43, 50.58) | 28.14 (27.22, 29.09) |
| 32 | 52.14 (50.89, 53.4) | 31.18 (30.8, 31.56) | 22.64 (22.26, 23.02) | 48.4 (47.75, 49.05) | 23.38 (22.5, 24.29) |
| 33 | 62.14 (60.47, 63.84) | 40.27 (39.78, 40.77) | 23.12 (22.7, 23.54) | 59.1 (58.29, 59.92) | 38.97 (37.63, 40.35) |
| 34 | 69.27 (67.32, 71.26) | 34.11 (33.66, 34.57) | 26.17 (25.69, 26.65) | 65.34 (64.47, 66.23) | 51.08 (49.72, 52.46) |
| 35 | 85.68 (83.42, 87.99) | 41.81 (41.27, 42.35) | 24.86 (24.39, 25.34) | 60.71 (59.89, 61.55) | 44.62 (43.6, 45.66) |
| 36 | 113.53 (111.08, 116.01) | 54.66 (54.07, 55.27) | 30.36 (29.82, 30.91) | 73.08 (72.16, 74) | 38.67 (37.81, 39.54) |
| 37 | 114.8 (112.77, 116.84) | 54.3 (53.79, 54.82) | 25.05 (24.59, 25.52) | 70.18 (69.36, 71.02) | 38.29 (37.47, 39.13) |
| 38 | 104.41 (102.81, 106.02) | 44.21 (43.81, 44.61) | 28.21 (27.7, 28.72) | 66.27 (65.51, 67.03) | 37.83 (37.03, 38.64) |
| 39 | 88.42 (87.13, 89.72) | 39.71 (39.35, 40.08) | 27.96 (27.47, 28.46) | 62.47 (61.75, 63.19) | 21.97 (21.39, 22.57) |
| 40 | 77.48 (76.33, 78.64) | 38.37 (38, 38.74) | 25.23 (24.77, 25.69) | 50.26 (49.62, 50.9) | 30.75 (29.88, 31.63) |
| 41 | 107.45 (106.08, 108.83) | 52.8 (52.35, 53.24) | 35.36 (34.8, 35.92) | 75 (74.14, 75.88) | 27.92 (27.06, 28.81) |
| 42 | 94.15 (93.04, 95.27) | 46.31 (45.94, 46.68) | 35.82 (35.33, 36.31) | 62.49 (61.77, 63.21) | 31.12 (30.14, 32.13) |
| 43 | 86.14 (85.15, 87.14) | 43.64 (43.3, 43.98) | 33.4 (32.98, 33.82) | 60.37 (59.67, 61.08) | 33.51 (32.46, 34.58) |
| 44 | 86.06 (85.08, 87.04) | 43.67 (43.33, 44) | 30.62 (30.26, 30.98) | 59.22 (58.51, 59.92) | 25.45 (24.54, 26.38) |
| 45 | 86.56 (85.59, 87.54) | 44.85 (44.51, 45.18) | 29.38 (29.05, 29.72) | 64.97 (64.22, 65.72) | 30.61 (29.48, 31.77) |
| 46 | 87.86 (86.89, 88.84) | 40.17 (39.86, 40.48) | 32.43 (32.08, 32.78) | 62.15 (61.45, 62.87) | 36.54 (35.26, 37.85) |
| 47 | 77.59 (76.69, 78.5) | 40.67 (40.35, 40.99) | 23.79 (23.52, 24.07) | 58.2 (57.52, 58.88) | 13.2 (12.47, 13.95) |
| 48 | 75.26 (74.32, 76.21) | 39.95 (39.63, 40.28) | 25.2 (24.89, 25.5) | 61.36 (60.66, 62.07) | 51.86 (49.59, 54.2) |
| 49 | 75.16 (74.16, 76.17) | 37.79 (37.46, 38.12) | 23.52 (23.22, 23.83) | 59.64 (58.95, 60.33) | 12.48 (11.59, 13.42) |
| 50 | 76.07 (75, 77.15) | 34.78 (34.44, 35.11) | 24.09 (23.76, 24.43) | 59.94 (59.26, 60.63) | 22.89 (21.01, 24.89) |
| 51 | 63.57 (62.54, 64.61) | 35.51 (35.14, 35.88) | 21.08 (20.75, 21.42) | 65.07 (64.37, 65.78) | 23.55 (21.36, 25.89) |
| 52 | 45.01 (44.03, 45.99) | 23.93 (23.61, 24.26) | 13.9 (13.59, 14.21) | 39.08 (38.56, 39.6) | 11.07 (9.42, 12.9) |

|  | Shaanxi | Gansu | Qinghai | Ningxia | Xinjiang |
| --- | --- | --- | --- | --- | --- |
| 1 | 29.31 (27.99, 30.68) | 10.26 (9.7, 10.84) | 4.06 (3.55, 4.61) | 4.31 (3.84, 4.82) | 2.72 (2.58, 2.87) |
| 2 | 34.11 (32.3, 35.98) | 14.52 (13.64, 15.43) | 2.28 (1.82, 2.82) | 2.28 (1.97, 2.61) | 2.73 (2.56, 2.91) |
| 3 | 33.2 (31.14, 35.36) | 7.21 (6.53, 7.93) | 2.11 (1.41, 3.01) | 1.88 (1.55, 2.25) | 2.2 (2.02, 2.39) |
| 4 | 53.7 (50.62, 56.9) | 16.12 (14.58, 17.77) | 25.76 (21.46, 30.61) | 2.65 (2.14, 3.25) | 2.17 (1.95, 2.41) |
| 5 | 41.63 (39.13, 44.23) | 21.73 (19.89, 23.69) | 8.41 (7.18, 9.76) | 6.61 (5.68, 7.63) | 7.68 (7.14, 8.26) |
| 6 | 58.58 (55.49, 61.79) | 26.28 (24.47, 28.19) | 16.88 (15.32, 18.54) | 4.69 (4.12, 5.3) | 1.43 (1.27, 1.61) |
| 7 | 50.5 (47.97, 53.11) | 23.61 (22.2, 25.07) | 2.91 (2.51, 3.35) | 1.9 (1.59, 2.25) | 7.33 (6.74, 7.94) |
| 8 | 65.49 (62.75, 68.3) | 17.47 (16.44, 18.56) | 4.57 (3.84, 5.4) | 4.54 (3.91, 5.24) | 5.84 (5.47, 6.22) |
| 9 | 63.09 (60.84, 65.39) | 10.56 (9.76, 11.4) | 20.72 (18.82, 22.75) | 6.22 (5.57, 6.92) | 3.7 (3.47, 3.95) |
| 10 | 56.66 (54.82, 58.54) | 19.8 (18.4, 21.28) | 5.02 (4.5, 5.58) | 5.27 (4.82, 5.75) | 4.83 (4.56, 5.11) |
| 11 | 107.78 (105.46, 110.13) | 50.88 (48.77, 53.05) | 36.29 (34.66, 37.97) | 4.39 (4.05, 4.75) | 5.32 (5.07, 5.58) |
| 12 | 59.33 (58.19, 60.49) | 23.67 (22.82, 24.55) | 8.48 (8.14, 8.83) | 5.22 (4.89, 5.57) | 5.53 (5.31, 5.75) |
| 13 | 74.09 (72.94, 75.26) | 20.16 (19.48, 20.86) | 9.86 (9.54, 10.2) | 3.08 (2.86, 3.3) | 6.33 (6.14, 6.53) |
| 14 | 80.1 (79.12, 81.09) | 30.1 (29.31, 30.91) | 5.91 (5.7, 6.13) | 4.61 (4.33, 4.9) | 5.55 (5.41, 5.7) |
| 15 | 68.53 (67.81, 69.26) | 26.52 (25.94, 27.11) | 11.3 (10.99, 11.62) | 6.29 (6, 6.59) | 6.6 (6.46, 6.73) |
| 16 | 65.09 (64.48, 65.7) | 27.54 (27.05, 28.05) | 9.45 (9.23, 9.68) | 4.98 (4.78, 5.18) | 5.64 (5.54, 5.73) |
| 17 | 60.36 (59.84, 60.89) | 23.3 (22.92, 23.69) | 6.54 (6.38, 6.71) | 4.87 (4.7, 5.04) | 6.09 (6.01, 6.18) |
| 18 | 64.56 (64.05, 65.07) | 30.1 (29.71, 30.5) | 11.08 (10.86, 11.3) | 5.42 (5.26, 5.58) | 6.4 (6.33, 6.47) |
| 19 | 78.64 (78.13, 79.15) | 34.51 (34.17, 34.86) | 13.22 (13.03, 13.42) | 7.69 (7.54, 7.86) | 7.1 (7.03, 7.16) |
| 20 | 55.51 (55.15, 55.87) | 25.23 (25, 25.45) | 8.76 (8.64, 8.88) | 5.36 (5.26, 5.45) | 6.17 (6.12, 6.21) |
| 21 | 58.75 (58.37, 59.12) | 25.2 (25, 25.4) | 8.58 (8.47, 8.69) | 5.46 (5.37, 5.54) | 5.09 (5.05, 5.12) |
| 22 | 54.94 (54.58, 55.3) | 26.06 (25.87, 26.24) | 8.25 (8.15, 8.35) | 4.58 (4.52, 4.65) | 5.56 (5.52, 5.59) |
| 23 | 48.66 (48.31, 49.01) | 20.85 (20.7, 21) | 7.77 (7.67, 7.87) | 4.31 (4.25, 4.37) | 4.32 (4.29, 4.35) |
| 24 | 50.27 (49.89, 50.66) | 23.38 (23.22, 23.54) | 7.26 (7.16, 7.35) | 4.29 (4.23, 4.35) | 4.92 (4.89, 4.95) |
| 25 | 57.56 (57.12, 58.01) | 22.26 (22.11, 22.41) | 6.81 (6.71, 6.91) | 4.42 (4.36, 4.48) | 4.37 (4.34, 4.4) |
| 26 | 52.93 (52.5, 53.36) | 21 (20.85, 21.14) | 8.35 (8.24, 8.47) | 4.4 (4.34, 4.46) | 3.39 (3.37, 3.42) |
| 27 | 57.02 (56.56, 57.49) | 21.27 (21.12, 21.42) | 8.89 (8.77, 9) | 4.42 (4.36, 4.47) | 4.57 (4.54, 4.61) |
| 28 | 46.4 (45.97, 46.83) | 15.37 (15.24, 15.5) | 7.26 (7.16, 7.36) | 3.09 (3.04, 3.14) | 3.69 (3.65, 3.72) |
| 29 | 44.55 (44.08, 45.02) | 14.92 (14.77, 15.07) | 5.29 (5.2, 5.38) | 3.48 (3.42, 3.54) | 3.23 (3.2, 3.27) |
| 30 | 47.05 (46.5, 47.6) | 18.43 (18.23, 18.64) | 5.62 (5.51, 5.74) | 3.86 (3.8, 3.93) | 3.28 (3.24, 3.31) |
| 31 | 39.37 (38.82, 39.93) | 13.4 (13.22, 13.59) | 6.09 (5.96, 6.23) | 2.52 (2.47, 2.58) | 2.99 (2.95, 3.03) |
| 32 | 45.52 (44.82, 46.23) | 15.97 (15.71, 16.22) | 5.09 (4.95, 5.24) | 3.06 (2.98, 3.14) | 3.84 (3.78, 3.9) |
| 33 | 47.35 (46.56, 48.15) | 17.41 (17.1, 17.71) | 5.43 (5.25, 5.61) | 3.89 (3.79, 4) | 3.57 (3.51, 3.63) |
| 34 | 52.48 (51.59, 53.38) | 17.03 (16.71, 17.36) | 4.75 (4.56, 4.95) | 3.67 (3.57, 3.77) | 3.33 (3.27, 3.39) |
| 35 | 53.24 (52.33, 54.16) | 25.07 (24.64, 25.5) | 6.61 (6.34, 6.89) | 3.88 (3.77, 3.98) | 4 (3.93, 4.07) |
| 36 | 68.03 (66.99, 69.08) | 21.38 (21.02, 21.74) | 5.72 (5.46, 6) | 4.14 (4.03, 4.25) | 4.34 (4.27, 4.42) |
| 37 | 64.87 (63.96, 65.78) | 27.1 (26.71, 27.49) | 9.66 (9.28, 10.05) | 5.12 (5, 5.24) | 4.5 (4.42, 4.58) |
| 38 | 50.84 (50.1, 51.59) | 20.43 (20.13, 20.73) | 7.87 (7.58, 8.17) | 3.96 (3.87, 4.06) | 4.59 (4.51, 4.66) |
| 39 | 64.05 (63.18, 64.92) | 21.2 (20.9, 21.51) | 8.09 (7.81, 8.38) | 4.23 (4.13, 4.33) | 3.72 (3.66, 3.79) |
| 40 | 42.91 (42.25, 43.58) | 14.32 (14.08, 14.57) | 3.82 (3.63, 4) | 3.13 (3.05, 3.21) | 3.35 (3.28, 3.41) |
| 41 | 71.03 (70.07, 72.01) | 27.58 (27.17, 28) | 12.94 (12.48, 13.41) | 4.73 (4.62, 4.84) | 5.4 (5.3, 5.49) |
| 42 | 63.09 (62.29, 63.91) | 22.2 (21.88, 22.52) | 7.14 (6.89, 7.4) | 4.04 (3.95, 4.14) | 5.11 (5.03, 5.19) |
| 43 | 47.42 (46.76, 48.09) | 18.97 (18.68, 19.26) | 4.31 (4.11, 4.51) | 3.04 (2.96, 3.12) | 3.66 (3.6, 3.72) |
| 44 | 59.01 (58.21, 59.82) | 19.11 (18.81, 19.42) | 10.22 (9.83, 10.61) | 4.1 (3.99, 4.21) | 3.58 (3.52, 3.65) |
| 45 | 51.88 (51.16, 52.62) | 17.64 (17.34, 17.94) | 4.74 (4.53, 4.97) | 3.49 (3.39, 3.59) | 3.26 (3.2, 3.33) |
| 46 | 48.15 (47.43, 48.88) | 16.46 (16.15, 16.77) | 6.07 (5.77, 6.37) | 2.8 (2.7, 2.89) | 4.09 (4.01, 4.18) |
| 47 | 43.3 (42.58, 44.02) | 16.5 (16.16, 16.85) | 3.19 (2.96, 3.43) | 2.88 (2.77, 3) | 4.26 (4.17, 4.34) |
| 48 | 41.2 (40.43, 41.99) | 13.65 (13.31, 13.99) | 11.92 (11.28, 12.58) | 3.07 (2.94, 3.21) | 3.94 (3.86, 4.02) |
| 49 | 39.77 (38.92, 40.64) | 18.81 (18.34, 19.3) | 5.97 (5.63, 6.32) | 3.24 (3.09, 3.4) | 2.47 (2.4, 2.53) |
| 50 | 38.04 (37.1, 39) | 13.37 (12.96, 13.79) | 6.81 (6.42, 7.21) | 2.22 (2.08, 2.36) | 3.69 (3.59, 3.79) |
| 51 | 44.69 (43.52, 45.88) | 15.09 (14.58, 15.62) | 5.2 (4.86, 5.56) | 3.11 (2.91, 3.32) | 3.24 (3.14, 3.34) |
| 52 | 28.75 (27.77, 29.76) | 13.36 (12.83, 13.91) | 3.3 (2.99, 3.62) | 1.13 (1, 1.27) | 1.96 (1.88, 2.04) |
