# Supplementary material for: Hand, Foot, and Mouth Disease in China: Modeling Epidemic Dynamics of Enterovirus Serotypes and Implications for Vaccination
Source: PLoS Med. 2016 Feb 16;13(2):e1001958. doi: 10.1371/journal.pmed.1001958 (PMC4755668; doi:10.1371/journal.pmed.1001958)
Supplement: S3 Table — β^s and 95% CIs on each weekly βs value (row) for CV-A16 for each province (column) from the two-serotype model with α = 0.95 and province-specific maximum likelihood estimates of cross-protection. (DOCX) [file pmed.1001958.s040.docx]

**S3 Table. Spatial and temporal variation in** $\boldsymbol{R}_{\boldsymbol{0}}$ **of CV-A16 across provinces.** $\hat{\beta}_{s}$ and 95% CIs on each weekly $\beta_{s}$ value (row) for CV-A16 for each province (column) from the two-serotype model with $\alpha$ = 0.95 and province-specific maximum likelihood estimates of cross-protection.

|  | Beijing | Tianjin | Hebei | Shanxi | Inner Mongolia |
| --- | --- | --- | --- | --- | --- |
| 1 | 18.95 (17.56, 20.4) | 12.21 (11.07, 13.42) | 17.17 (16.69, 17.66) | 19.53 (18.64, 20.45) | 12.7 (12.01, 13.42) |
| 2 | 23.2 (21.36, 25.15) | 13.71 (12.23, 15.32) | 13.87 (13.36, 14.4) | 19.24 (18.13, 20.41) | 10.69 (9.86, 11.57) |
| 3 | 13.17 (11.69, 14.77) | 11.04 (9.51, 12.71) | 14.18 (13.51, 14.87) | 14.07 (12.88, 15.33) | 12.19 (10.94, 13.54) |
| 4 | 37.1 (33.56, 40.89) | 13.22 (11.09, 15.6) | 29.64 (28.42, 30.9) | 40.87 (37.91, 43.98) | 16.09 (14.19, 18.14) |
| 5 | 30.25 (27.52, 33.15) | 28.41 (24.71, 32.47) | 20.44 (19.54, 21.37) | 21.43 (19.6, 23.36) | 27.7 (24.84, 30.77) |
| 6 | 28.11 (25.61, 30.77) | 17.57 (15.21, 20.16) | 21.8 (20.81, 22.82) | 30.32 (27.78, 33.02) | 14.59 (12.77, 16.57) |
| 7 | 49.16 (45.9, 52.57) | 33.45 (30.06, 37.08) | 32.03 (30.8, 33.28) | 48.8 (45.61, 52.13) | 50.79 (46.67, 55.14) |
| 8 | 34.46 (32.44, 36.56) | 19.91 (17.97, 21.99) | 27.53 (26.57, 28.51) | 37.94 (35.75, 40.23) | 15.61 (14.14, 17.18) |
| 9 | 36.53 (34.69, 38.42) | 26.78 (24.63, 29.06) | 31.02 (30.09, 31.96) | 36.2 (34.3, 38.17) | 39.55 (36.8, 42.42) |
| 10 | 37.96 (36.34, 39.61) | 20.05 (18.5, 21.68) | 31.23 (30.42, 32.04) | 35.31 (33.59, 37.08) | 24.99 (23.38, 26.67) |
| 11 | 57.02 (55.33, 58.73) | 28.79 (27, 30.65) | 42.24 (41.43, 43.06) | 76.61 (74.27, 79.01) | 35.81 (34.01, 37.67) |
| 12 | 35.18 (34.25, 36.13) | 28.33 (26.91, 29.8) | 33.1 (32.56, 33.65) | 44.47 (43.31, 45.64) | 33.03 (31.67, 34.43) |
| 13 | 37.67 (36.8, 38.55) | 19.9 (18.92, 20.9) | 43.47 (42.93, 44) | 46.09 (45.08, 47.11) | 35.82 (34.64, 37.02) |
| 14 | 42.59 (41.78, 43.41) | 33.97 (32.73, 35.25) | 44.3 (43.89, 44.71) | 68.05 (67.02, 69.1) | 25.7 (24.89, 26.51) |
| 15 | 34.06 (33.46, 34.68) | 26.62 (25.79, 27.47) | 38.19 (37.9, 38.48) | 36.38 (35.84, 36.93) | 26.82 (26.04, 27.62) |
| 16 | 39.58 (38.97, 40.2) | 23.58 (22.91, 24.26) | 36.96 (36.72, 37.2) | 42.47 (41.91, 43.03) | 25.63 (24.93, 26.36) |
| 17 | 34.53 (34.03, 35.04) | 31.03 (30.33, 31.74) | 39.49 (39.28, 39.7) | 46.4 (45.87, 46.93) | 28.11 (27.4, 28.83) |
| 18 | 42.19 (41.67, 42.72) | 29.48 (28.92, 30.04) | 37.7 (37.52, 37.87) | 46.97 (46.52, 47.42) | 41.29 (40.51, 42.08) |
| 19 | 36.02 (35.6, 36.44) | 30.01 (29.54, 30.48) | 33.92 (33.77, 34.06) | 48.47 (48.07, 48.87) | 33.26 (32.73, 33.8) |
| 20 | 37.81 (37.4, 38.22) | 27.58 (27.2, 27.96) | 31.32 (31.19, 31.45) | 46.84 (46.51, 47.18) | 32.78 (32.33, 33.24) |
| 21 | 36.56 (36.19, 36.94) | 29.7 (29.35, 30.06) | 32.38 (32.25, 32.51) | 43.36 (43.08, 43.65) | 37.58 (37.16, 38) |
| 22 | 36.16 (35.8, 36.52) | 26.9 (26.61, 27.19) | 28.99 (28.86, 29.11) | 41.47 (41.22, 41.73) | 30.01 (29.7, 30.32) |
| 23 | 31.54 (31.21, 31.87) | 20.9 (20.67, 21.14) | 25.72 (25.6, 25.84) | 34.11 (33.89, 34.33) | 28.63 (28.35, 28.91) |
| 24 | 35.27 (34.9, 35.64) | 23.19 (22.93, 23.46) | 28.29 (28.15, 28.43) | 34.97 (34.73, 35.21) | 29.04 (28.77, 29.3) |
| 25 | 36.1 (35.72, 36.48) | 25.2 (24.92, 25.48) | 31.43 (31.27, 31.58) | 35.68 (35.43, 35.93) | 29.15 (28.9, 29.41) |
| 26 | 36.86 (36.47, 37.24) | 23.62 (23.36, 23.89) | 29.03 (28.89, 29.18) | 33.83 (33.57, 34.09) | 27.16 (26.93, 27.39) |
| 27 | 28.56 (28.23, 28.9) | 17.01 (16.78, 17.24) | 23.54 (23.4, 23.68) | 26.84 (26.59, 27.08) | 19.43 (19.24, 19.62) |
| 28 | 32.74 (32.33, 33.16) | 20.39 (20.1, 20.69) | 27.09 (26.92, 27.25) | 33.21 (32.89, 33.53) | 22.88 (22.64, 23.13) |
| 29 | 28.22 (27.81, 28.63) | 18.69 (18.38, 19) | 24.19 (24.03, 24.36) | 28.65 (28.33, 28.97) | 19.16 (18.92, 19.4) |
| 30 | 29.61 (29.13, 30.09) | 18.3 (17.95, 18.65) | 24.52 (24.34, 24.71) | 32.2 (31.82, 32.59) | 20.84 (20.56, 21.14) |
| 31 | 30.69 (30.16, 31.24) | 19.22 (18.82, 19.63) | 25.34 (25.14, 25.54) | 33 (32.58, 33.42) | 21.24 (20.92, 21.56) |
| 32 | 30.31 (29.73, 30.9) | 17.55 (17.13, 17.98) | 23.28 (23.07, 23.48) | 26.98 (26.58, 27.39) | 18.75 (18.43, 19.09) |
| 33 | 30.43 (29.81, 31.07) | 19.99 (19.46, 20.52) | 23.86 (23.63, 24.09) | 27.17 (26.7, 27.64) | 22.8 (22.39, 23.23) |
| 34 | 28.96 (28.3, 29.63) | 17.74 (17.21, 18.28) | 23.46 (23.21, 23.7) | 29.66 (29.11, 30.21) | 22.3 (21.87, 22.74) |
| 35 | 41.11 (40.24, 41.99) | 29.62 (28.85, 30.41) | 33.8 (33.48, 34.12) | 38.52 (37.84, 39.21) | 31.44 (30.9, 31.98) |
| 36 | 37.24 (36.47, 38.02) | 22.41 (21.81, 23.01) | 26.74 (26.49, 27) | 49.04 (48.31, 49.77) | 36.51 (36, 37.03) |
| 37 | 37.79 (37.04, 38.56) | 23.07 (22.46, 23.69) | 30.61 (30.34, 30.89) | 42.21 (41.64, 42.79) | 31.52 (31.13, 31.92) |
| 38 | 29.58 (28.93, 30.23) | 20.16 (19.6, 20.74) | 23.1 (22.88, 23.33) | 34.86 (34.38, 35.34) | 25.2 (24.88, 25.52) |
| 39 | 26.65 (25.99, 27.32) | 17.47 (16.91, 18.05) | 24.54 (24.29, 24.79) | 29.73 (29.29, 30.18) | 19.39 (19.11, 19.67) |
| 40 | 28.48 (27.71, 29.26) | 19.23 (18.56, 19.91) | 22.17 (21.92, 22.42) | 29.12 (28.64, 29.6) | 19.61 (19.28, 19.93) |
| 41 | 39.54 (38.56, 40.53) | 27.1 (26.25, 27.97) | 31.38 (31.05, 31.71) | 46.12 (45.47, 46.79) | 36.56 (36.07, 37.06) |
| 42 | 37.82 (36.95, 38.72) | 21.7 (21.01, 22.4) | 30.58 (30.28, 30.88) | 39.79 (39.26, 40.33) | 28.14 (27.78, 28.51) |
| 43 | 37.02 (36.2, 37.84) | 29.56 (28.75, 30.39) | 31.65 (31.36, 31.93) | 39.08 (38.58, 39.58) | 27.16 (26.82, 27.5) |
| 44 | 37.37 (36.6, 38.16) | 26.87 (26.2, 27.55) | 30.12 (29.86, 30.38) | 39.94 (39.46, 40.43) | 25.06 (24.74, 25.38) |
| 45 | 28.93 (28.29, 29.58) | 19.52 (18.99, 20.05) | 26.82 (26.59, 27.05) | 31.75 (31.34, 32.16) | 22.77 (22.46, 23.07) |
| 46 | 29.81 (29.12, 30.51) | 18.48 (17.94, 19.03) | 23.01 (22.79, 23.22) | 29.17 (28.76, 29.58) | 21 (20.69, 21.32) |
| 47 | 24.68 (24.03, 25.34) | 16.77 (16.22, 17.34) | 21.2 (20.98, 21.43) | 23.37 (22.97, 23.77) | 22.15 (21.8, 22.51) |
| 48 | 23.86 (23.14, 24.6) | 16.1 (15.48, 16.73) | 19.48 (19.23, 19.72) | 28.16 (27.63, 28.71) | 19.65 (19.29, 20.01) |
| 49 | 22.15 (21.37, 22.96) | 12.92 (12.3, 13.56) | 19.86 (19.58, 20.14) | 25.85 (25.29, 26.42) | 23.06 (22.63, 23.51) |
| 50 | 25.97 (24.98, 26.99) | 18.12 (17.21, 19.07) | 20.23 (19.91, 20.55) | 22.9 (22.3, 23.51) | 15.82 (15.44, 16.2) |
| 51 | 22.12 (21.15, 23.12) | 15.79 (14.89, 16.72) | 17.99 (17.65, 18.33) | 24.66 (23.92, 25.41) | 18.56 (18.05, 19.07) |
| 52 | 15.29 (14.38, 16.24) | 10.03 (9.24, 10.85) | 15.94 (15.57, 16.32) | 21.87 (21.09, 22.67) | 10.55 (10.13, 11) |

|  | Liaoning | Jilin | Heilongjiang |
| --- | --- | --- | --- |
| 1 | 11.42 (7.51, 16.51) | 29.86 (17.82, 46.38) | 26.15 (19.17, 34.66) |
| 2 | 17.76 (10.92, 26.98) | 36.77 (23.2, 54.82) | 57.4 (43.3, 74.26) |
| 3 | 169.47 (143.01, 199) | 366.19 (323.06, 412.99) | 229.98 (202.84, 259.43) |
| 4 | 43.69 (38.49, 49.33) | 29.86 (26.17, 33.89) | 54.63 (48.41, 61.35) |
| 5 | 14.56 (12.32, 17.06) | 10.89 (8.63, 13.5) | 35.36 (30.6, 40.58) |
| 6 | 36.59 (31.95, 41.66) | 66.06 (56.51, 76.62) | 37.08 (31.37, 43.45) |
| 7 | 24.26 (21.17, 27.63) | 21.9 (18.13, 26.14) | 61.4 (53.04, 70.6) |
| 8 | 26.39 (23.16, 29.9) | 44.41 (37.8, 51.75) | 16.21 (12.63, 20.4) |
| 9 | 30.56 (27.2, 34.19) | 39.6 (34.18, 45.56) | 124.28 (107.6, 142.6) |
| 10 | 26.03 (23.24, 29.04) | 18.88 (15.5, 22.73) | 35.23 (29.9, 41.15) |
| 11 | 77.96 (73.16, 82.96) | 188.9 (174.35, 204.23) | 121.57 (110.22, 133.67) |
| 12 | 29.25 (27.56, 31.01) | 44.63 (41.66, 47.74) | 52.6 (47.87, 57.63) |
| 13 | 33.27 (31.57, 35.02) | 36.99 (34.64, 39.43) | 124.3 (117.22, 131.65) |
| 14 | 40.4 (38.74, 42.1) | 46.98 (44.46, 49.6) | 65.01 (61.78, 68.34) |
| 15 | 46.45 (45, 47.93) | 95.48 (92.4, 98.63) | 101.99 (98.41, 105.66) |
| 16 | 32.45 (31.51, 33.4) | 49.94 (48.58, 51.33) | 84.45 (82.08, 86.87) |
| 17 | 36.24 (35.32, 37.17) | 40.6 (39.55, 41.67) | 59.89 (58.28, 61.52) |
| 18 | 48.29 (47.36, 49.24) | 62.31 (61.08, 63.57) | 101.73 (99.73, 103.74) |
| 19 | 45.45 (44.74, 46.16) | 52.15 (51.27, 53.04) | 83.04 (81.69, 84.4) |
| 20 | 42.76 (42.2, 43.32) | 49.28 (48.54, 50.03) | 71.17 (70.14, 72.21) |
| 21 | 43.25 (42.78, 43.72) | 55.46 (54.76, 56.16) | 74.34 (73.39, 75.3) |
| 22 | 41.28 (40.89, 41.66) | 50.9 (50.33, 51.47) | 72.51 (71.69, 73.34) |
| 23 | 33.89 (33.58, 34.2) | 47.5 (47, 48) | 77.94 (77.19, 78.69) |
| 24 | 41.85 (41.52, 42.18) | 44.36 (43.91, 44.81) | 69.68 (69.08, 70.29) |
| 25 | 39.36 (39.08, 39.65) | 51.46 (50.99, 51.93) | 65.6 (65.07, 66.13) |
| 26 | 38.88 (38.62, 39.15) | 47.72 (47.31, 48.13) | 66.97 (66.46, 67.48) |
| 27 | 33.2 (32.97, 33.43) | 37.82 (37.47, 38.17) | 64.46 (63.99, 64.94) |
| 28 | 31.91 (31.68, 32.14) | 39.55 (39.16, 39.94) | 58.93 (58.5, 59.37) |
| 29 | 30.74 (30.5, 30.98) | 37 (36.61, 37.39) | 55.31 (54.88, 55.74) |
| 30 | 28.4 (28.16, 28.64) | 35.98 (35.56, 36.41) | 49.61 (49.19, 50.04) |
| 31 | 30.08 (29.81, 30.36) | 39.58 (39.09, 40.06) | 63.82 (63.28, 64.36) |
| 32 | 26.19 (25.92, 26.47) | 35.02 (34.55, 35.49) | 46.23 (45.77, 46.68) |
| 33 | 23.32 (23.03, 23.61) | 35.84 (35.32, 36.37) | 49.37 (48.83, 49.91) |
| 34 | 23.84 (23.49, 24.18) | 43.55 (42.92, 44.18) | 55.45 (54.82, 56.09) |
| 35 | 26.66 (26.25, 27.08) | 40.07 (39.48, 40.67) | 46.3 (45.69, 46.92) |
| 36 | 25.65 (25.21, 26.1) | 38.01 (37.42, 38.61) | 56.51 (55.73, 57.29) |
| 37 | 30.89 (30.35, 31.43) | 44.65 (43.97, 45.33) | 60.55 (59.72, 61.4) |
| 38 | 23.12 (22.66, 23.6) | 29.56 (29.02, 30.11) | 49.7 (48.94, 50.48) |
| 39 | 24.73 (24.18, 25.29) | 33.36 (32.67, 34.05) | 55.24 (54.34, 56.14) |
| 40 | 20.65 (20.09, 21.22) | 33.31 (32.55, 34.08) | 38.34 (37.56, 39.14) |
| 41 | 42.5 (41.53, 43.48) | 47.74 (46.73, 48.76) | 73.05 (71.71, 74.41) |
| 42 | 33.08 (32.36, 33.8) | 38.66 (37.81, 39.52) | 56.28 (55.21, 57.36) |
| 43 | 21.75 (21.19, 22.31) | 29.09 (28.33, 29.86) | 40.84 (39.91, 41.79) |
| 44 | 27.37 (26.64, 28.12) | 35.27 (34.29, 36.28) | 54.93 (53.64, 56.25) |
| 45 | 24.78 (24.05, 25.51) | 34.52 (33.49, 35.58) | 50.18 (48.92, 51.46) |
| 46 | 26.09 (25.28, 26.91) | 30.64 (29.6, 31.71) | 46.17 (44.89, 47.48) |
| 47 | 11.34 (10.79, 11.91) | 16.62 (15.76, 17.51) | 13.66 (12.91, 14.45) |
| 48 | 13.64 (12.69, 14.64) | 28.86 (27.13, 30.66) | 35.58 (33.25, 38.03) |
| 49 | 22.35 (20.61, 24.18) | 29.02 (27.03, 31.1) | 52.97 (49.55, 56.53) |
| 50 | 21.87 (19.98, 23.88) | 33.88 (31.44, 36.45) | 28.06 (25.65, 30.61) |
| 51 | 6.19 (5.11, 7.42) | 1.59 (1.09, 2.23) | 20.94 (18.24, 23.9) |
| 52 | 12.55 (9.5, 16.21) | 17.63 (10.52, 27.38) | 21.03 (16.84, 25.85) |

|  | Shanghai | Jiangsu | Zhejiang | Anhui | Fujian | Jiangxi | Shandong |
| --- | --- | --- | --- | --- | --- | --- | --- |
| 1 | 17.65 (17.08, 18.23) | 21.25 (20.94, 21.56) | 15.11 (14.82, 15.4) | 16.89 (16.63, 17.15) | 18.06 (17.65, 18.48) | 13.09 (12.83, 13.34) | 7.06 (6.89, 7.23) |
| 2 | 16.73 (16.09, 17.4) | 18.8 (18.47, 19.13) | 15.74 (15.4, 16.08) | 16.51 (16.23, 16.8) | 18.2 (17.74, 18.67) | 13.1 (12.82, 13.38) | 6.92 (6.73, 7.12) |
| 3 | 25.18 (24.23, 26.15) | 27.06 (26.58, 27.54) | 20.84 (20.41, 21.28) | 26.22 (25.81, 26.63) | 23.99 (23.4, 24.59) | 16.97 (16.62, 17.31) | 8.73 (8.48, 8.98) |
| 4 | 20.17 (19.35, 21.02) | 22.99 (22.55, 23.44) | 18.18 (17.79, 18.58) | 21.07 (20.74, 21.4) | 23.39 (22.83, 23.97) | 15.78 (15.47, 16.1) | 9.36 (9.1, 9.63) |
| 5 | 21.34 (20.43, 22.28) | 23.4 (22.92, 23.89) | 15.27 (14.89, 15.65) | 21.55 (21.22, 21.88) | 18.29 (17.8, 18.79) | 12.46 (12.18, 12.74) | 8.39 (8.14, 8.64) |
| 6 | 30.67 (29.52, 31.85) | 28.27 (27.7, 28.85) | 23.27 (22.74, 23.8) | 21.96 (21.63, 22.3) | 25.85 (25.21, 26.5) | 23.33 (22.91, 23.76) | 12.98 (12.66, 13.31) |
| 7 | 32.58 (31.55, 33.64) | 35.92 (35.29, 36.55) | 22.65 (22.18, 23.14) | 27.13 (26.77, 27.49) | 27.92 (27.31, 28.55) | 17.28 (16.98, 17.58) | 12.75 (12.49, 13.02) |
| 8 | 40.55 (39.57, 41.55) | 40.83 (40.25, 41.41) | 26.74 (26.26, 27.22) | 32.51 (32.16, 32.87) | 31.26 (30.68, 31.85) | 20.53 (20.22, 20.84) | 16.35 (16.09, 16.61) |
| 9 | 35.01 (34.3, 35.73) | 40.71 (40.24, 41.19) | 32.3 (31.85, 32.75) | 28.25 (27.99, 28.52) | 30.26 (29.78, 30.75) | 23.89 (23.6, 24.18) | 12.44 (12.26, 12.61) |
| 10 | 25.84 (25.33, 26.36) | 31.52 (31.18, 31.87) | 22.75 (22.45, 23.06) | 23.81 (23.59, 24.02) | 26.19 (25.8, 26.59) | 18.83 (18.61, 19.04) | 11.13 (10.99, 11.28) |
| 11 | 33.6 (33.03, 34.18) | 37.77 (37.41, 38.13) | 29.99 (29.66, 30.32) | 33.49 (33.25, 33.74) | 31.4 (30.99, 31.81) | 27.02 (26.79, 27.26) | 15.39 (15.23, 15.55) |
| 12 | 30.04 (29.58, 30.52) | 36.34 (36.04, 36.65) | 27.45 (27.19, 27.71) | 25.45 (25.27, 25.63) | 28.63 (28.3, 28.97) | 20.15 (19.98, 20.31) | 11.9 (11.78, 12.01) |
| 13 | 28.67 (28.25, 29.09) | 33.48 (33.22, 33.75) | 25.53 (25.3, 25.75) | 29.55 (29.37, 29.73) | 24.6 (24.31, 24.88) | 18.57 (18.42, 18.71) | 12.86 (12.76, 12.97) |
| 14 | 31.55 (31.14, 31.98) | 33.65 (33.4, 33.9) | 25.25 (25.05, 25.45) | 26.24 (26.09, 26.39) | 29.18 (28.88, 29.49) | 23.41 (23.25, 23.56) | 14.72 (14.62, 14.83) |
| 15 | 25.56 (25.21, 25.9) | 31.59 (31.37, 31.82) | 24.45 (24.26, 24.63) | 23.76 (23.62, 23.89) | 29.66 (29.38, 29.94) | 20.15 (20.02, 20.27) | 11.12 (11.04, 11.19) |
| 16 | 29.97 (29.59, 30.35) | 35.47 (35.24, 35.7) | 25.59 (25.41, 25.77) | 25.79 (25.65, 25.93) | 28.11 (27.86, 28.36) | 19.7 (19.59, 19.81) | 14.66 (14.57, 14.74) |
| 17 | 25.61 (25.29, 25.94) | 31.1 (30.9, 31.3) | 22.72 (22.57, 22.88) | 23.88 (23.75, 24.01) | 26.23 (26, 26.45) | 16.14 (16.05, 16.24) | 13.34 (13.27, 13.41) |
| 18 | 31.37 (31, 31.74) | 37.68 (37.47, 37.9) | 25.2 (25.04, 25.36) | 25.89 (25.76, 26.03) | 28.81 (28.58, 29.04) | 18.92 (18.81, 19.03) | 14.32 (14.25, 14.38) |
| 19 | 31.4 (31.06, 31.75) | 33.39 (33.21, 33.56) | 23.68 (23.53, 23.83) | 24.67 (24.54, 24.8) | 30.61 (30.39, 30.84) | 16.02 (15.92, 16.12) | 12.79 (12.74, 12.85) |
| 20 | 27.54 (27.24, 27.85) | 29.99 (29.82, 30.15) | 22.44 (22.29, 22.58) | 22.55 (22.43, 22.67) | 27 (26.81, 27.19) | 15.41 (15.31, 15.52) | 12.94 (12.89, 12.99) |
| 21 | 26.77 (26.47, 27.08) | 30.25 (30.08, 30.42) | 20.81 (20.67, 20.95) | 22.88 (22.75, 23.01) | 24.31 (24.13, 24.49) | 14.58 (14.47, 14.69) | 12.81 (12.77, 12.86) |
| 22 | 28.56 (28.24, 28.89) | 30.89 (30.72, 31.07) | 24.06 (23.91, 24.22) | 21.6 (21.47, 21.73) | 27.15 (26.96, 27.35) | 16.89 (16.77, 17.02) | 11.45 (11.41, 11.5) |
| 23 | 30.76 (30.43, 31.09) | 32.49 (32.31, 32.67) | 24.4 (24.24, 24.56) | 23 (22.86, 23.15) | 25.13 (24.94, 25.32) | 16.55 (16.42, 16.68) | 10.23 (10.2, 10.27) |
| 24 | 29.67 (29.35, 29.98) | 30.21 (30.04, 30.38) | 22.77 (22.63, 22.92) | 22.99 (22.84, 23.14) | 25.22 (25.02, 25.41) | 16.01 (15.88, 16.14) | 10.71 (10.67, 10.75) |
| 25 | 28.81 (28.5, 29.11) | 29.81 (29.63, 29.98) | 24.39 (24.23, 24.54) | 21.48 (21.34, 21.63) | 24.64 (24.45, 24.84) | 16.72 (16.58, 16.86) | 11.01 (10.97, 11.06) |
| 26 | 24.34 (24.06, 24.63) | 27.36 (27.19, 27.53) | 18.91 (18.77, 19.04) | 21.19 (21.03, 21.34) | 21.08 (20.9, 21.27) | 13.76 (13.64, 13.89) | 11.3 (11.25, 11.35) |
| 27 | 21.15 (20.86, 21.44) | 21.87 (21.71, 22.04) | 15.81 (15.67, 15.94) | 17.81 (17.66, 17.97) | 20.77 (20.56, 20.98) | 13.13 (12.99, 13.27) | 9.84 (9.8, 9.89) |
| 28 | 20.69 (20.36, 21.02) | 22.49 (22.29, 22.69) | 16.35 (16.18, 16.52) | 19.12 (18.94, 19.3) | 19.87 (19.64, 20.09) | 15.02 (14.85, 15.2) | 9.81 (9.77, 9.86) |
| 29 | 19.89 (19.51, 20.26) | 22.12 (21.89, 22.35) | 17.63 (17.43, 17.83) | 18.95 (18.76, 19.16) | 23.67 (23.39, 23.96) | 14.81 (14.63, 14.99) | 9.89 (9.84, 9.94) |
| 30 | 18.31 (17.89, 18.73) | 21.16 (20.9, 21.43) | 17.5 (17.27, 17.73) | 17.63 (17.42, 17.85) | 23.03 (22.75, 23.32) | 14.19 (14, 14.38) | 9.1 (9.05, 9.15) |
| 31 | 22.11 (21.56, 22.67) | 24.76 (24.43, 25.09) | 18.1 (17.85, 18.36) | 19.72 (19.47, 19.97) | 20.2 (19.92, 20.48) | 16.07 (15.86, 16.29) | 8.94 (8.88, 8.99) |
| 32 | 22.93 (22.33, 23.55) | 25.22 (24.86, 25.58) | 18.85 (18.57, 19.14) | 19.22 (18.95, 19.48) | 24.61 (24.28, 24.95) | 15.66 (15.44, 15.87) | 8.69 (8.63, 8.75) |
| 33 | 25.27 (24.6, 25.95) | 26.67 (26.28, 27.07) | 20.42 (20.11, 20.73) | 20.14 (19.85, 20.43) | 24.03 (23.7, 24.36) | 15.94 (15.72, 16.16) | 7.93 (7.86, 8) |
| 34 | 27.14 (26.44, 27.85) | 30.5 (30.07, 30.93) | 21.7 (21.38, 22.03) | 22.42 (22.1, 22.74) | 27.37 (27.01, 27.73) | 19.49 (19.24, 19.74) | 8.62 (8.54, 8.7) |
| 35 | 26.72 (26.06, 27.4) | 32 (31.58, 32.43) | 22.19 (21.86, 22.51) | 21.77 (21.47, 22.09) | 28.73 (28.38, 29.08) | 18.06 (17.84, 18.29) | 9.04 (8.95, 9.13) |
| 36 | 33.69 (32.96, 34.42) | 38.28 (37.85, 38.72) | 28.41 (28.05, 28.77) | 26.82 (26.48, 27.16) | 34.81 (34.46, 35.17) | 21.42 (21.19, 21.66) | 10.08 (9.98, 10.18) |
| 37 | 28.02 (27.45, 28.61) | 30.99 (30.65, 31.33) | 21.89 (21.62, 22.16) | 25.67 (25.37, 25.97) | 27.06 (26.79, 27.32) | 16.78 (16.6, 16.97) | 9.92 (9.81, 10.02) |
| 38 | 20.94 (20.47, 21.42) | 24.69 (24.4, 24.99) | 20.46 (20.2, 20.72) | 17.49 (17.26, 17.72) | 25.08 (24.83, 25.34) | 14.1 (13.94, 14.27) | 8.04 (7.95, 8.14) |
| 39 | 25.82 (25.24, 26.41) | 33.1 (32.74, 33.46) | 20.81 (20.55, 21.08) | 27.06 (26.75, 27.38) | 22.25 (22.01, 22.49) | 16.98 (16.78, 17.18) | 11.01 (10.89, 11.14) |
| 40 | 21.31 (20.79, 21.83) | 22.97 (22.7, 23.25) | 16.05 (15.82, 16.29) | 16.99 (16.77, 17.22) | 17.36 (17.13, 17.59) | 14.49 (14.31, 14.68) | 8.16 (8.06, 8.26) |
| 41 | 31.2 (30.53, 31.88) | 37.09 (36.7, 37.49) | 24.98 (24.65, 25.31) | 26.72 (26.41, 27.04) | 26.39 (26.06, 26.72) | 18.3 (18.09, 18.52) | 11.86 (11.73, 12) |
| 42 | 26.77 (26.21, 27.34) | 30.02 (29.72, 30.33) | 19.27 (19.01, 19.54) | 22.96 (22.69, 23.22) | 21.61 (21.33, 21.9) | 15.32 (15.13, 15.51) | 11.42 (11.29, 11.54) |
| 43 | 26.71 (26.17, 27.26) | 30.34 (30.04, 30.64) | 22.39 (22.1, 22.69) | 21.67 (21.42, 21.92) | 24.29 (23.97, 24.62) | 17.97 (17.76, 18.18) | 9.86 (9.75, 9.97) |
| 44 | 33.37 (32.78, 33.97) | 34.63 (34.31, 34.94) | 25.18 (24.88, 25.48) | 27.24 (26.95, 27.52) | 25.1 (24.77, 25.43) | 17.34 (17.14, 17.54) | 11.94 (11.82, 12.06) |
| 45 | 25.95 (25.5, 26.41) | 29.4 (29.13, 29.66) | 21.4 (21.15, 21.66) | 25.36 (25.12, 25.61) | 26.07 (25.74, 26.41) | 17.55 (17.36, 17.75) | 10.25 (10.15, 10.35) |
| 46 | 28.53 (28.06, 29.01) | 31.81 (31.54, 32.08) | 23.52 (23.26, 23.78) | 23.81 (23.59, 24.03) | 24.72 (24.41, 25.03) | 16.45 (16.27, 16.64) | 9.74 (9.64, 9.84) |
| 47 | 19.35 (18.98, 19.73) | 21.95 (21.73, 22.16) | 16.44 (16.23, 16.65) | 18.37 (18.18, 18.56) | 17.69 (17.43, 17.95) | 12.32 (12.17, 12.48) | 6.94 (6.86, 7.03) |
| 48 | 24.96 (24.48, 25.45) | 28.06 (27.78, 28.34) | 19.62 (19.37, 19.87) | 21.74 (21.52, 21.96) | 23.39 (23.04, 23.73) | 14.78 (14.58, 14.98) | 8.46 (8.35, 8.58) |
| 49 | 25.2 (24.71, 25.7) | 28.88 (28.6, 29.17) | 19.58 (19.32, 19.84) | 21.35 (21.13, 21.57) | 21.13 (20.81, 21.46) | 14.88 (14.67, 15.09) | 8.81 (8.68, 8.94) |
| 50 | 21.11 (20.66, 21.56) | 24.45 (24.19, 24.71) | 17.82 (17.57, 18.08) | 19.53 (19.32, 19.75) | 21.57 (21.22, 21.92) | 14.47 (14.26, 14.68) | 8.01 (7.88, 8.14) |
| 51 | 22.28 (21.77, 22.79) | 25.33 (25.04, 25.61) | 18.98 (18.7, 19.27) | 19.74 (19.52, 19.97) | 23.87 (23.49, 24.25) | 15 (14.78, 15.22) | 7.72 (7.58, 7.86) |
| 52 | 14.74 (14.31, 15.19) | 19.78 (19.52, 20.05) | 13.03 (12.79, 13.27) | 14.58 (14.37, 14.78) | 13.07 (12.8, 13.36) | 10.33 (10.14, 10.52) | 5.92 (5.79, 6.06) |

|  | Henan | Hubei | Hunan | Guangdong | Guangxi | Hainan |
| --- | --- | --- | --- | --- | --- | --- |
| 1 | 19.95 (19.72, 20.19) | 19.4 (19.12, 19.69) | 19.22 (18.89, 19.56) | 24.2 (23.8, 24.61) | 12.59 (12.34, 12.85) | 17.23 (16.56, 17.93) |
| 2 | 17.06 (16.83, 17.3) | 18.63 (18.31, 18.97) | 16.3 (15.95, 16.66) | 21.53 (21.12, 21.95) | 11.19 (10.94, 11.46) | 22.27 (21.41, 23.14) |
| 3 | 24.39 (24.06, 24.73) | 23.57 (23.13, 24.01) | 23.56 (23.04, 24.08) | 24.48 (23.98, 24.98) | 12.07 (11.76, 12.38) | 14.36 (13.69, 15.05) |
| 4 | 27.69 (27.35, 28.04) | 20.52 (20.09, 20.95) | 24.29 (23.77, 24.83) | 31.65 (31.04, 32.27) | 17.51 (17.11, 17.91) | 27.37 (26.27, 28.51) |
| 5 | 22.75 (22.46, 23.05) | 24.57 (24.04, 25.1) | 17.92 (17.47, 18.38) | 23.62 (23.13, 24.12) | 12.07 (11.77, 12.38) | 19.26 (18.45, 20.09) |
| 6 | 26.17 (25.85, 26.49) | 31.19 (30.59, 31.8) | 27.65 (27, 28.3) | 30.01 (29.41, 30.62) | 14.66 (14.3, 15.03) | 18.98 (18.14, 19.83) |
| 7 | 26.64 (26.33, 26.95) | 31.37 (30.83, 31.93) | 25.67 (25.1, 26.25) | 43.45 (42.76, 44.16) | 20.09 (19.68, 20.51) | 38.72 (37.47, 39.99) |
| 8 | 39.46 (39.11, 39.82) | 40.41 (39.84, 40.98) | 34.55 (33.91, 35.2) | 40.31 (39.77, 40.86) | 18.49 (18.15, 18.83) | 28.93 (28.12, 29.75) |
| 9 | 29.44 (29.2, 29.68) | 35.97 (35.53, 36.41) | 30.65 (30.14, 31.16) | 35.95 (35.52, 36.39) | 19.49 (19.18, 19.8) | 27.07 (26.39, 27.77) |
| 10 | 27.79 (27.58, 28.01) | 30.34 (30, 30.69) | 29.57 (29.13, 30.01) | 37.04 (36.65, 37.44) | 18.33 (18.07, 18.59) | 27.51 (26.88, 28.14) |
| 11 | 38.48 (38.24, 38.71) | 43.18 (42.79, 43.57) | 40.85 (40.38, 41.33) | 36.85 (36.5, 37.2) | 21.46 (21.2, 21.71) | 23.16 (22.64, 23.69) |
| 12 | 29.96 (29.8, 30.13) | 33.32 (33.04, 33.6) | 32.47 (32.14, 32.8) | 40.1 (39.77, 40.43) | 21.89 (21.67, 22.1) | 26.54 (26, 27.09) |
| 13 | 32.72 (32.56, 32.88) | 34.84 (34.59, 35.1) | 36.43 (36.12, 36.73) | 33.06 (32.8, 33.32) | 21.87 (21.69, 22.05) | 23.05 (22.59, 23.52) |
| 14 | 31.46 (31.32, 31.6) | 33.11 (32.88, 33.33) | 39.53 (39.26, 39.8) | 40.52 (40.24, 40.79) | 24.52 (24.36, 24.68) | 23.7 (23.25, 24.16) |
| 15 | 26.23 (26.11, 26.35) | 30.6 (30.39, 30.8) | 32.31 (32.11, 32.51) | 35.71 (35.48, 35.93) | 18.97 (18.86, 19.09) | 20.95 (20.54, 21.36) |
| 16 | 32.05 (31.92, 32.18) | 32.15 (31.95, 32.36) | 32.3 (32.12, 32.48) | 37.88 (37.66, 38.1) | 19.44 (19.33, 19.55) | 23.06 (22.63, 23.5) |
| 17 | 26.02 (25.91, 26.13) | 27.45 (27.28, 27.63) | 30.14 (29.98, 30.3) | 34.74 (34.55, 34.93) | 16.52 (16.43, 16.62) | 21.93 (21.52, 22.35) |
| 18 | 28.53 (28.41, 28.65) | 31.08 (30.88, 31.27) | 31.76 (31.6, 31.92) | 38.03 (37.84, 38.23) | 18.27 (18.17, 18.37) | 20.44 (20.05, 20.84) |
| 19 | 23.57 (23.46, 23.68) | 26.09 (25.92, 26.27) | 26.77 (26.63, 26.91) | 40.77 (40.59, 40.95) | 17.99 (17.89, 18.08) | 23.88 (23.44, 24.32) |
| 20 | 27 (26.87, 27.12) | 27.53 (27.34, 27.72) | 25.81 (25.67, 25.95) | 34.3 (34.15, 34.45) | 15.6 (15.51, 15.69) | 20.28 (19.9, 20.67) |
| 21 | 25.65 (25.53, 25.78) | 27.16 (26.96, 27.35) | 26.29 (26.15, 26.44) | 30.91 (30.77, 31.05) | 15.85 (15.76, 15.95) | 20.06 (19.67, 20.46) |
| 22 | 23.96 (23.83, 24.08) | 27.99 (27.78, 28.19) | 28.62 (28.46, 28.78) | 33.8 (33.65, 33.95) | 17.47 (17.36, 17.57) | 20.63 (20.22, 21.05) |
| 23 | 24.84 (24.71, 24.98) | 28.29 (28.08, 28.5) | 29.35 (29.19, 29.52) | 34.82 (34.67, 34.97) | 18.31 (18.2, 18.42) | 23.47 (23.03, 23.92) |
| 24 | 23.41 (23.27, 23.55) | 25.41 (25.21, 25.61) | 27.85 (27.7, 28.01) | 30.88 (30.74, 31.02) | 15.21 (15.12, 15.31) | 21.3 (20.89, 21.71) |
| 25 | 25.62 (25.47, 25.78) | 27.63 (27.42, 27.85) | 26.59 (26.44, 26.75) | 33.65 (33.49, 33.8) | 16.3 (16.19, 16.41) | 23.13 (22.7, 23.56) |
| 26 | 24.68 (24.52, 24.83) | 23.81 (23.6, 24.02) | 24.05 (23.9, 24.2) | 33.51 (33.36, 33.67) | 15.87 (15.76, 15.98) | 19.94 (19.56, 20.33) |
| 27 | 22.54 (22.39, 22.69) | 20.74 (20.53, 20.95) | 20.59 (20.44, 20.74) | 26.97 (26.83, 27.11) | 12.77 (12.67, 12.87) | 16.93 (16.56, 17.3) |
| 28 | 22.44 (22.28, 22.6) | 21.4 (21.16, 21.65) | 21.9 (21.73, 22.08) | 26.96 (26.8, 27.12) | 12.7 (12.58, 12.81) | 20.03 (19.59, 20.49) |
| 29 | 22.33 (22.16, 22.51) | 22.17 (21.89, 22.45) | 22 (21.8, 22.2) | 30.54 (30.36, 30.73) | 14.27 (14.13, 14.42) | 19.96 (19.5, 20.42) |
| 30 | 21.62 (21.44, 21.8) | 18.93 (18.64, 19.22) | 21.09 (20.87, 21.3) | 31.1 (30.91, 31.3) | 15.09 (14.94, 15.25) | 20.99 (20.5, 21.48) |
| 31 | 22.2 (22, 22.4) | 20.38 (20.03, 20.73) | 20.68 (20.45, 20.92) | 30.16 (29.96, 30.36) | 14.79 (14.63, 14.95) | 19.25 (18.78, 19.73) |
| 32 | 20.6 (20.4, 20.81) | 21.78 (21.38, 22.19) | 22.88 (22.6, 23.16) | 29.59 (29.39, 29.8) | 15.21 (15.04, 15.38) | 20.39 (19.89, 20.9) |
| 33 | 21.25 (21.03, 21.48) | 23.04 (22.59, 23.5) | 22.84 (22.55, 23.14) | 32.54 (32.31, 32.77) | 15.79 (15.61, 15.96) | 21.04 (20.52, 21.56) |
| 34 | 22.45 (22.2, 22.69) | 27.83 (27.31, 28.36) | 30.07 (29.72, 30.43) | 32.73 (32.5, 32.95) | 17.14 (16.96, 17.33) | 22.64 (22.11, 23.19) |
| 35 | 33.51 (33.2, 33.83) | 35.3 (34.75, 35.87) | 43.02 (42.63, 43.41) | 36.51 (36.27, 36.75) | 19.24 (19.05, 19.43) | 26.4 (25.84, 26.96) |
| 36 | 23.58 (23.36, 23.81) | 33.19 (32.73, 33.66) | 30.23 (29.98, 30.49) | 36.44 (36.21, 36.67) | 19.32 (19.14, 19.49) | 30.54 (30, 31.08) |
| 37 | 29.2 (28.95, 29.45) | 34.98 (34.55, 35.4) | 25.95 (25.73, 26.18) | 33.88 (33.67, 34.09) | 18.01 (17.86, 18.17) | 28.04 (27.6, 28.48) |
| 38 | 18.86 (18.67, 19.04) | 23.76 (23.46, 24.06) | 23.49 (23.28, 23.71) | 27.65 (27.47, 27.83) | 14.21 (14.08, 14.34) | 23.69 (23.33, 24.05) |
| 39 | 27.14 (26.89, 27.39) | 27.22 (26.88, 27.56) | 28.13 (27.88, 28.37) | 30.3 (30.09, 30.51) | 14.51 (14.36, 14.65) | 20.64 (20.32, 20.97) |
| 40 | 20.55 (20.34, 20.76) | 24.89 (24.57, 25.21) | 25.47 (25.25, 25.7) | 26.86 (26.66, 27.06) | 15.86 (15.7, 16.01) | 19.59 (19.26, 19.92) |
| 41 | 28.85 (28.58, 29.12) | 35.69 (35.29, 36.08) | 29.3 (29.06, 29.55) | 33.81 (33.56, 34.06) | 16.42 (16.26, 16.58) | 23.71 (23.33, 24.1) |
| 42 | 26.11 (25.88, 26.35) | 28.34 (28.04, 28.65) | 26.96 (26.74, 27.19) | 24.99 (24.78, 25.19) | 14.12 (13.97, 14.27) | 20.9 (20.55, 21.26) |
| 43 | 25.23 (25.01, 25.46) | 31.15 (30.84, 31.46) | 29.74 (29.51, 29.97) | 33.8 (33.53, 34.07) | 16.1 (15.93, 16.27) | 21.48 (21.11, 21.86) |
| 44 | 31.44 (31.2, 31.68) | 35.31 (35, 35.62) | 30.43 (30.21, 30.65) | 31.41 (31.15, 31.66) | 19.28 (19.1, 19.47) | 22.05 (21.67, 22.44) |
| 45 | 27.43 (27.23, 27.63) | 30.77 (30.51, 31.02) | 27.92 (27.72, 28.12) | 33.44 (33.18, 33.71) | 15.49 (15.34, 15.64) | 21.41 (21.03, 21.8) |
| 46 | 26.91 (26.72, 27.1) | 30.06 (29.82, 30.3) | 27.51 (27.32, 27.7) | 32.09 (31.83, 32.34) | 15.82 (15.66, 15.97) | 22.3 (21.89, 22.71) |
| 47 | 22.24 (22.07, 22.41) | 23.67 (23.46, 23.87) | 22.25 (22.08, 22.42) | 22.47 (22.26, 22.69) | 13.96 (13.81, 14.11) | 15.68 (15.33, 16.03) |
| 48 | 24.13 (23.95, 24.32) | 27.93 (27.7, 28.18) | 24.96 (24.76, 25.16) | 28.53 (28.25, 28.81) | 16.59 (16.41, 16.76) | 18.74 (18.29, 19.2) |
| 49 | 25.51 (25.31, 25.7) | 24.74 (24.52, 24.97) | 24.25 (24.05, 24.45) | 26.84 (26.55, 27.13) | 14.09 (13.93, 14.25) | 18.43 (17.94, 18.92) |
| 50 | 23.7 (23.51, 23.88) | 22.49 (22.27, 22.72) | 22.42 (22.22, 22.62) | 25.7 (25.4, 26) | 14.97 (14.8, 15.14) | 17.57 (17.05, 18.1) |
| 51 | 18.83 (18.66, 19) | 22.95 (22.69, 23.2) | 20.01 (19.81, 20.22) | 26.62 (26.29, 26.95) | 15.39 (15.21, 15.57) | 18.85 (18.26, 19.46) |
| 52 | 16.8 (16.62, 16.98) | 19.28 (19.04, 19.53) | 13.35 (13.16, 13.54) | 20.26 (19.96, 20.57) | 8.83 (8.69, 8.97) | 16.52 (15.93, 17.12) |

|  | Chongqing | Sichuan | Guizhou | Yunnan | Tibet |
| --- | --- | --- | --- | --- | --- |
| 1 | 18.63 (18.29, 18.97) | 36.88 (36.54, 37.22) | 19.23 (18.83, 19.63) | 25.67 (25.36, 25.99) | 5.53 (4.75, 6.39) |
| 2 | 14.89 (14.57, 15.22) | 27.8 (27.5, 28.09) | 18.56 (18.13, 18.99) | 23.57 (23.26, 23.87) | 7.62 (6.19, 9.25) |
| 3 | 17.35 (16.93, 17.76) | 30.35 (30, 30.7) | 19.17 (18.68, 19.67) | 20.47 (20.18, 20.76) | 43.61 (38.92, 48.66) |
| 4 | 17.23 (16.79, 17.69) | 24.5 (24.16, 24.84) | 20.81 (20.25, 21.38) | 18.76 (18.45, 19.07) | 6.57 (5.52, 7.76) |
| 5 | 16.65 (16.17, 17.13) | 29.54 (29.1, 29.99) | 24.54 (23.9, 25.18) | 22.7 (22.31, 23.1) | 2.21 (1.32, 3.44) |
| 6 | 21.32 (20.72, 21.93) | 43.67 (43.08, 44.26) | 23.75 (23.15, 24.36) | 24.17 (23.75, 24.6) | 42.79 (32.37, 55.24) |
| 7 | 32.62 (31.9, 33.35) | 52.09 (51.52, 52.67) | 32.7 (32.01, 33.4) | 26.86 (26.41, 27.32) | 71.43 (63.17, 80.38) |
| 8 | 32.16 (31.58, 32.73) | 46.19 (45.74, 46.64) | 25.21 (24.7, 25.72) | 28.12 (27.68, 28.56) | 16 (14.2, 17.94) |
| 9 | 30.39 (29.93, 30.84) | 55.2 (54.77, 55.64) | 34.97 (34.4, 35.55) | 29.52 (29.11, 29.94) | 13.33 (11.78, 15.01) |
| 10 | 26.93 (26.58, 27.3) | 41.17 (40.86, 41.47) | 36.49 (36.01, 36.98) | 34.05 (33.64, 34.46) | 21.43 (19.38, 23.62) |
| 11 | 22.16 (21.87, 22.46) | 38.57 (38.29, 38.85) | 26.4 (26.06, 26.74) | 24.18 (23.89, 24.47) | 37.76 (35.49, 40.12) |
| 12 | 26.46 (26.14, 26.78) | 48.63 (48.32, 48.94) | 31.2 (30.85, 31.55) | 34.51 (34.16, 34.87) | 33.1 (31.76, 34.48) |
| 13 | 24.39 (24.11, 24.67) | 42.6 (42.34, 42.86) | 35.03 (34.7, 35.36) | 29.87 (29.59, 30.16) | 13.71 (13.12, 14.31) |
| 14 | 33.82 (33.5, 34.13) | 54.16 (53.88, 54.45) | 36.79 (36.5, 37.08) | 31.69 (31.43, 31.96) | 26.32 (25.45, 27.2) |
| 15 | 25.46 (25.24, 25.69) | 39.91 (39.71, 40.12) | 33.31 (33.08, 33.54) | 26.6 (26.38, 26.82) | 38 (37.2, 38.82) |
| 16 | 22.54 (22.33, 22.74) | 40.82 (40.61, 41.04) | 28.06 (27.88, 28.25) | 30.89 (30.66, 31.13) | 10.91 (10.62, 11.2) |
| 17 | 21.44 (21.24, 21.64) | 36.01 (35.81, 36.22) | 26.88 (26.7, 27.06) | 27.91 (27.71, 28.11) | 16.77 (16.32, 17.23) |
| 18 | 27.43 (27.19, 27.66) | 48.64 (48.39, 48.9) | 32.6 (32.4, 32.8) | 31.23 (31.02, 31.44) | 13.2 (12.8, 13.61) |
| 19 | 24.47 (24.26, 24.68) | 37.46 (37.25, 37.67) | 28 (27.83, 28.17) | 27.99 (27.81, 28.17) | 18.15 (17.63, 18.69) |
| 20 | 19.72 (19.53, 19.9) | 36.89 (36.66, 37.12) | 22.31 (22.17, 22.46) | 26.24 (26.07, 26.41) | 16.74 (16.26, 17.23) |
| 21 | 21.47 (21.25, 21.68) | 39.7 (39.45, 39.95) | 22.87 (22.7, 23.04) | 29.04 (28.85, 29.22) | 17.17 (16.68, 17.66) |
| 22 | 22.91 (22.68, 23.14) | 41.24 (40.97, 41.5) | 24.34 (24.15, 24.53) | 27.76 (27.59, 27.94) | 23.8 (23.23, 24.38) |
| 23 | 22.48 (22.24, 22.71) | 38.03 (37.77, 38.29) | 26.91 (26.69, 27.12) | 23.28 (23.12, 23.43) | 14.48 (14.1, 14.87) |
| 24 | 23.71 (23.47, 23.96) | 43.64 (43.34, 43.93) | 24.31 (24.1, 24.52) | 25.97 (25.78, 26.15) | 10.01 (9.66, 10.36) |
| 25 | 20.04 (19.82, 20.27) | 40.72 (40.43, 41) | 24.43 (24.21, 24.65) | 25.84 (25.65, 26.02) | 26.41 (25.68, 27.16) |
| 26 | 19.21 (18.97, 19.45) | 36.79 (36.52, 37.07) | 25.27 (25.03, 25.51) | 25.15 (24.95, 25.34) | 20.65 (20.12, 21.18) |
| 27 | 15.41 (15.18, 15.65) | 30.65 (30.38, 30.91) | 19.19 (18.97, 19.41) | 19.75 (19.57, 19.93) | 14.72 (14.31, 15.14) |
| 28 | 16.55 (16.26, 16.86) | 31.69 (31.38, 32.01) | 20.03 (19.76, 20.3) | 20.89 (20.67, 21.11) | 15.76 (15.29, 16.23) |
| 29 | 17.5 (17.15, 17.86) | 32.63 (32.27, 32.99) | 24.08 (23.75, 24.42) | 22.97 (22.71, 23.23) | 14.06 (13.6, 14.54) |
| 30 | 18.15 (17.74, 18.56) | 32.45 (32.06, 32.85) | 20.78 (20.46, 21.11) | 23.35 (23.07, 23.63) | 11.95 (11.47, 12.44) |
| 31 | 22.62 (22.12, 23.12) | 35.49 (35.04, 35.94) | 24.09 (23.7, 24.48) | 26.1 (25.78, 26.42) | 21.18 (20.42, 21.96) |
| 32 | 16.6 (16.19, 17.02) | 34.06 (33.6, 34.52) | 22.79 (22.39, 23.19) | 22.24 (21.94, 22.53) | 15.02 (14.44, 15.62) |
| 33 | 16.79 (16.32, 17.27) | 36.88 (36.38, 37.38) | 21.12 (20.72, 21.52) | 24.73 (24.4, 25.07) | 19.01 (18.31, 19.73) |
| 34 | 19.37 (18.81, 19.95) | 31.23 (30.77, 31.69) | 23.64 (23.18, 24.11) | 27.81 (27.44, 28.18) | 31.39 (30.53, 32.26) |
| 35 | 29.65 (28.92, 30.39) | 44.33 (43.74, 44.93) | 28.92 (28.4, 29.45) | 33.44 (33.05, 33.83) | 44.27 (43.49, 45.05) |
| 36 | 31.64 (31.01, 32.29) | 51.03 (50.45, 51.61) | 28.58 (28.09, 29.07) | 30.58 (30.24, 30.91) | 19.1 (18.76, 19.44) |
| 37 | 27.36 (26.87, 27.86) | 46.02 (45.55, 46.5) | 23.73 (23.31, 24.15) | 29.9 (29.59, 30.21) | 19.26 (18.91, 19.62) |
| 38 | 28.38 (27.94, 28.83) | 39.18 (38.78, 39.57) | 26.18 (25.73, 26.63) | 28.51 (28.23, 28.8) | 17.69 (17.34, 18.04) |
| 39 | 18.95 (18.63, 19.27) | 30.46 (30.12, 30.8) | 18.34 (17.96, 18.71) | 17.86 (17.64, 18.08) | 8.92 (8.65, 9.19) |
| 40 | 22.56 (22.19, 22.93) | 37.85 (37.44, 38.27) | 22.59 (22.12, 23.07) | 22.2 (21.9, 22.5) | 17.04 (16.5, 17.59) |
| 41 | 30.57 (30.15, 30.99) | 49.81 (49.35, 50.28) | 32.71 (32.13, 33.3) | 34.07 (33.67, 34.47) | 13.16 (12.66, 13.68) |
| 42 | 25.59 (25.26, 25.92) | 40.44 (40.08, 40.81) | 29.88 (29.41, 30.37) | 25.6 (25.3, 25.91) | 14.14 (13.53, 14.77) |
| 43 | 23.94 (23.65, 24.24) | 39.02 (38.67, 39.36) | 30.02 (29.58, 30.46) | 30.73 (30.39, 31.07) | 25.95 (25.02, 26.91) |
| 44 | 24.8 (24.5, 25.09) | 43.49 (43.14, 43.85) | 28.73 (28.34, 29.12) | 26.01 (25.73, 26.3) | 15.95 (15.33, 16.59) |
| 45 | 23.64 (23.36, 23.91) | 40.19 (39.87, 40.51) | 26.15 (25.81, 26.49) | 26.55 (26.26, 26.84) | 17.63 (16.92, 18.36) |
| 46 | 22.9 (22.64, 23.17) | 35.63 (35.34, 35.92) | 28.18 (27.84, 28.53) | 28.22 (27.92, 28.52) | 24.32 (23.47, 25.19) |
| 47 | 25.16 (24.88, 25.43) | 42.15 (41.83, 42.48) | 27.38 (27.06, 27.7) | 33.99 (33.67, 34.3) | 9.47 (9, 9.96) |
| 48 | 20.39 (20.16, 20.63) | 36.24 (35.95, 36.53) | 22.18 (21.91, 22.45) | 26.4 (26.16, 26.65) | 38.49 (37.16, 39.84) |
| 49 | 20.04 (19.8, 20.29) | 33.91 (33.62, 34.19) | 21.39 (21.11, 21.68) | 27.11 (26.85, 27.36) | 5.75 (5.39, 6.13) |
| 50 | 19.85 (19.59, 20.11) | 30.76 (30.47, 31.05) | 21.63 (21.33, 21.94) | 27.22 (26.97, 27.48) | 15.43 (14.38, 16.53) |
| 51 | 16.8 (16.54, 17.05) | 34.75 (34.42, 35.09) | 22.8 (22.47, 23.14) | 20.45 (20.23, 20.67) | 6.51 (5.78, 7.3) |
| 52 | 13.83 (13.57, 14.09) | 24.23 (23.94, 24.52) | 13.99 (13.72, 14.26) | 18.77 (18.54, 19) | 10.67 (9.22, 12.27) |

|  | Shaanxi | Gansu | Qinghai | Ningxia | Xinjiang |
| --- | --- | --- | --- | --- | --- |
| 1 | 23.8 (22.68, 24.96) | 17.14 (16.11, 18.22) | 2.24 (1.94, 2.57) | 10.28 (9.25, 11.38) | 14.55 (13.66, 15.49) |
| 2 | 29.06 (27.53, 30.65) | 24.61 (22.96, 26.34) | 1.89 (1.52, 2.31) | 6.03 (5.31, 6.81) | 18.12 (16.88, 19.41) |
| 3 | 11.07 (10.03, 12.18) | 5.44 (4.61, 6.36) | 1.51 (1.06, 2.07) | 3.29 (2.68, 4) | 4.46 (3.81, 5.19) |
| 4 | 33.85 (30.58, 37.35) | 20.77 (17.03, 25.02) | 0.42 (0.13, 0.98) | 8.32 (6.74, 10.13) | 20.56 (17.4, 24.08) |
| 5 | 33.07 (29.76, 36.63) | 15.05 (11.38, 19.43) | 3.15 (0.98, 7.31) | 3.37 (2.4, 4.58) | 7.79 (5.84, 10.13) |
| 6 | 50.46 (46.19, 54.97) | 105.08 (91.35, 120.13) | 33.02 (24.02, 44.04) | 7.99 (5.68, 10.84) | 59.53 (50.39, 69.72) |
| 7 | 178.39 (171.52, 185.43) | 181.93 (172.03, 192.19) | 11.65 (9.78, 13.76) | 66.59 (59.44, 74.29) | 149.3 (140.18, 158.8) |
| 8 | 44.44 (42.93, 45.98) | 32.54 (30.87, 34.26) | 2.1 (1.65, 2.62) | 6.19 (5.43, 7.02) | 23.72 (22.32, 25.18) |
| 9 | 54.24 (52.77, 55.74) | 24.6 (23.24, 26.02) | 54.21 (50.92, 57.63) | 27.11 (25.24, 29.06) | 23.92 (22.58, 25.31) |
| 10 | 51.34 (50.19, 52.52) | 44.36 (42.35, 46.43) | 10.81 (10.4, 11.23) | 7.09 (6.55, 7.65) | 18.19 (17.08, 19.35) |
| 11 | 46.47 (45.55, 47.41) | 42.31 (40.7, 43.97) | 4.71 (4.54, 4.89) | 7.88 (7.26, 8.54) | 29.42 (27.89, 31) |
| 12 | 46.12 (45.31, 46.95) | 39.2 (37.9, 40.54) | 5.8 (5.62, 5.99) | 13.15 (12.31, 14.02) | 30.99 (29.64, 32.38) |
| 13 | 55.58 (54.78, 56.39) | 32.57 (31.53, 33.64) | 5.93 (5.77, 6.1) | 7.92 (7.39, 8.47) | 41.37 (40.06, 42.71) |
| 14 | 72.48 (71.72, 73.24) | 53.66 (52.36, 54.98) | 4.07 (3.95, 4.19) | 12.73 (12.03, 13.45) | 33.95 (33.07, 34.86) |
| 15 | 51.82 (51.35, 52.3) | 47.34 (46.41, 48.28) | 7.34 (7.17, 7.51) | 18.07 (17.38, 18.78) | 44.29 (43.46, 45.14) |
| 16 | 53 (52.58, 53.42) | 46.33 (45.59, 47.09) | 6.7 (6.57, 6.83) | 12.66 (12.24, 13.08) | 34.44 (33.91, 34.98) |
| 17 | 46.2 (45.86, 46.55) | 37.68 (37.12, 38.24) | 4.22 (4.14, 4.31) | 12 (11.66, 12.35) | 36.45 (35.99, 36.91) |
| 18 | 49.92 (49.58, 50.26) | 53.5 (52.88, 54.11) | 7.29 (7.17, 7.4) | 12.48 (12.17, 12.79) | 34.55 (34.19, 34.91) |
| 19 | 51.1 (50.78, 51.41) | 46.91 (46.46, 47.37) | 5.53 (5.44, 5.61) | 15.21 (14.92, 15.51) | 36.83 (36.51, 37.15) |
| 20 | 42.35 (42.08, 42.61) | 42.38 (42.02, 42.74) | 5.76 (5.68, 5.84) | 12.62 (12.41, 12.84) | 35.76 (35.51, 36.02) |
| 21 | 45.79 (45.51, 46.07) | 43.22 (42.89, 43.55) | 5.58 (5.51, 5.65) | 13.75 (13.56, 13.96) | 30.51 (30.31, 30.72) |
| 22 | 42.74 (42.47, 43.01) | 43.99 (43.7, 44.29) | 5.32 (5.25, 5.39) | 11.19 (11.04, 11.35) | 32.27 (32.08, 32.47) |
| 23 | 34.25 (34, 34.49) | 31.96 (31.73, 32.19) | 4.13 (4.07, 4.18) | 9.31 (9.18, 9.45) | 24.5 (24.34, 24.65) |
| 24 | 34.46 (34.18, 34.74) | 36.42 (36.16, 36.67) | 4.4 (4.33, 4.46) | 10.03 (9.89, 10.18) | 27.97 (27.79, 28.15) |
| 25 | 43.25 (42.9, 43.61) | 38.43 (38.16, 38.69) | 4.23 (4.16, 4.3) | 10.53 (10.38, 10.69) | 27.42 (27.25, 27.6) |
| 26 | 41.42 (41.07, 41.78) | 36.84 (36.59, 37.1) | 5.37 (5.29, 5.46) | 10.49 (10.34, 10.64) | 21.6 (21.44, 21.77) |
| 27 | 38.19 (37.83, 38.54) | 30.65 (30.41, 30.89) | 5.03 (4.96, 5.11) | 9.76 (9.61, 9.9) | 24.52 (24.32, 24.73) |
| 28 | 37.08 (36.7, 37.46) | 26.95 (26.7, 27.2) | 4.59 (4.52, 4.66) | 7.66 (7.53, 7.79) | 23.93 (23.72, 24.15) |
| 29 | 33.79 (33.4, 34.18) | 25.89 (25.6, 26.19) | 3.19 (3.13, 3.25) | 9.07 (8.91, 9.24) | 20.32 (20.1, 20.54) |
| 30 | 36.31 (35.85, 36.78) | 32.09 (31.7, 32.48) | 3.47 (3.39, 3.55) | 8.96 (8.78, 9.14) | 20.19 (19.94, 20.45) |
| 31 | 30.19 (29.73, 30.66) | 23.61 (23.25, 23.97) | 4.42 (4.31, 4.53) | 6.72 (6.56, 6.89) | 19.37 (19.08, 19.67) |
| 32 | 39.95 (39.33, 40.59) | 30.79 (30.29, 31.31) | 3.73 (3.63, 3.83) | 8.57 (8.35, 8.8) | 25.23 (24.84, 25.62) |
| 33 | 33.29 (32.7, 33.88) | 29.03 (28.51, 29.57) | 3.26 (3.16, 3.37) | 9.11 (8.87, 9.36) | 23.08 (22.7, 23.46) |
| 34 | 38.95 (38.24, 39.66) | 29.48 (28.9, 30.07) | 2.7 (2.59, 2.82) | 9.34 (9.09, 9.6) | 20.58 (20.2, 20.96) |
| 35 | 50.78 (49.96, 51.62) | 55.27 (54.41, 56.15) | 4.92 (4.73, 5.13) | 12.32 (12.02, 12.62) | 30.05 (29.54, 30.57) |
| 36 | 58.54 (57.73, 59.35) | 40.16 (39.57, 40.76) | 4.13 (3.95, 4.3) | 11.99 (11.73, 12.25) | 29.52 (29.05, 30) |
| 37 | 45.14 (44.53, 45.75) | 44.34 (43.75, 44.93) | 7.1 (6.87, 7.34) | 12.2 (11.96, 12.44) | 28.5 (28.06, 28.93) |
| 38 | 40.25 (39.69, 40.81) | 39.01 (38.51, 39.51) | 4.87 (4.71, 5.03) | 10.87 (10.67, 11.08) | 27.43 (27.02, 27.84) |
| 39 | 46.73 (46.12, 47.35) | 36.67 (36.2, 37.14) | 4.93 (4.78, 5.09) | 9.93 (9.73, 10.12) | 24.36 (23.98, 24.74) |
| 40 | 32.46 (31.98, 32.95) | 23.57 (23.19, 23.95) | 2.38 (2.28, 2.49) | 7.86 (7.69, 8.03) | 24.24 (23.86, 24.63) |
| 41 | 51.6 (50.91, 52.3) | 46.59 (45.93, 47.25) | 8.16 (7.9, 8.42) | 11.67 (11.43, 11.91) | 31.64 (31.19, 32.11) |
| 42 | 48.96 (48.35, 49.58) | 42.49 (41.94, 43.05) | 4.18 (4.04, 4.32) | 9.92 (9.72, 10.13) | 28.88 (28.48, 29.29) |
| 43 | 39.83 (39.32, 40.35) | 37.67 (37.19, 38.16) | 2.75 (2.64, 2.87) | 8.64 (8.45, 8.83) | 28.25 (27.87, 28.64) |
| 44 | 41.79 (41.24, 42.33) | 30.97 (30.54, 31.41) | 5.45 (5.25, 5.67) | 9.43 (9.21, 9.64) | 20.9 (20.58, 21.22) |
| 45 | 40.04 (39.51, 40.58) | 29.23 (28.77, 29.69) | 2.82 (2.69, 2.96) | 7.76 (7.56, 7.97) | 21.34 (20.98, 21.7) |
| 46 | 36.59 (36.07, 37.11) | 27.6 (27.11, 28.09) | 4.06 (3.87, 4.27) | 6.87 (6.66, 7.09) | 24.47 (24.05, 24.9) |
| 47 | 32.11 (31.6, 32.63) | 25.47 (24.95, 26) | 1.62 (1.49, 1.76) | 6.62 (6.37, 6.87) | 19.51 (19.12, 19.9) |
| 48 | 26.74 (26.22, 27.27) | 21.94 (21.38, 22.51) | 7.93 (7.48, 8.4) | 7.36 (7.05, 7.68) | 19.49 (19.06, 19.93) |
| 49 | 30.16 (29.49, 30.84) | 32.74 (31.91, 33.6) | 4.15 (3.91, 4.4) | 7.63 (7.27, 8) | 15.35 (14.92, 15.79) |
| 50 | 28.04 (27.31, 28.78) | 22.3 (21.61, 23) | 4.58 (4.32, 4.84) | 5.63 (5.3, 5.99) | 23.52 (22.86, 24.21) |
| 51 | 29.24 (28.37, 30.12) | 21.93 (21.12, 22.77) | 2.08 (1.91, 2.26) | 7.68 (7.18, 8.2) | 16.94 (16.37, 17.52) |
| 52 | 24.1 (23.21, 25.01) | 22.96 (21.95, 24) | 2.88 (2.6, 3.17) | 3.24 (2.9, 3.61) | 11.69 (11.14, 12.26) |
