# Supplementary material for: Hand, Foot, and Mouth Disease in China: Modeling Epidemic Dynamics of Enterovirus Serotypes and Implications for Vaccination
Source: PLoS Med. 2016 Feb 16;13(2):e1001958. doi: 10.1371/journal.pmed.1001958 (PMC4755668; doi:10.1371/journal.pmed.1001958)
Supplement: S4 Table — We omit co-infection from this analysis, as co-infection of a single individual with both serotypes (EV-A71 and CV-A16) is rarer than expected by chance, given a reasonable sample size of EV-A71 and CV-A16 infections in a given year. We determined this using data from two published studies and χ2 tests. (DOCX) [file pmed.1001958.s041.docx]

**S4 Table. Omission of co-infection from the two-serotype model.** We omit co-infection from this analysis, as co-infection of a single individual with both serotypes (EV-A71 and CV-A16) is rarer than expected by chance, given a reasonable sample size of EV-A71 and CV-A16 infections in a given year. We determined this using data from two published studies and $\chi^{2}$ tests.

|  | | | | | | | |
| --- | --- | --- | --- | --- | --- | --- | --- |
| **Year** | **Observed**  **EV-A71** | **Observed**  **CV-A16** | **Observed**  **Co-infection** | **Total Cases** | **Expected**  **Co-infection** | $\boldsymbol{\chi}^{\boldsymbol{2}}$ | **p-value** |
| 2009 | 122 | 8 | 3 | 133 | 7.34 | 2.56 | < 0.15 |
| 2010 | 250 | 20 | 3 | 273 | 18.32 | 12.81 | < 0.0005 |
| Reference: [32] Yan et al 2012 | | | | | | | |
| **Year** | **Observed**  **EV-A71** | **Observed**  **CV-A16** | **Observed**  **Co-infection** | **Total Cases** | **Expected**  **Co-infection** | $\boldsymbol{\chi}^{\boldsymbol{2}}$ | **p-value** |
| 2007 | 2 | 43 | 0 | 45 | 1.91 | 1.91 | < 0.20 |
| 2008 | 117 | 24 | 0 | 141 | 19.91 | 19.91 | < 0.0005 |
| 2009 | 12 | 58 | 0 | 70 | 9.94 | 9.94 | < 0.0025 |

Reference: [33] Li et al 2011
