# Supplementary material for: Hand, Foot, and Mouth Disease in China: Modeling Epidemic Dynamics of Enterovirus Serotypes and Implications for Vaccination
Source: PLoS Med. 2016 Feb 16;13(2):e1001958. doi: 10.1371/journal.pmed.1001958 (PMC4755668; doi:10.1371/journal.pmed.1001958)
Supplement: S6 Table — Mean proportion of individuals that are susceptible to EV-A71 and CV-A16 (S¯), reporting rate of EV-A71 and CV-A16 (ρ), mean weekly estimated transmission rate of EV-A71 and CV-A16 (β¯), and coefficient of variation (CV) in estimated transmission rate of EV-A71 and CV-A16 by province. Calculated with α = 0.95 and province-specific maximum likelihood estimates of cross-protection from the two-serotype model. The 95% CIs for S¯ are derived from the profile likelihood using the χ 2 distribution with 1 degree of freedom; the 95% CIs for ρ are derived from the standard errors for the coefficient 1/ρ in the OLS regression of cumulative births and cumulative cases; the maximum standard errors (se) of the log(βs) terms across weekly β^s values are from the log-linear regression, representing variability around estimated βs. (DOCX) [file pmed.1001958.s043.docx]

**S6 Table. Three-serotype TSIR model estimates for EV-A71 and CV-A16.** Mean proportion of individuals that are susceptible to EV-A71 and CV-A16 ($\bar{S}$), reporting rate of EV-A71 and CV-A16 ($\rho$), mean weekly estimated transmission rate of EV-A71 and CV-A16 ($\bar{\beta}$), and coefficient of variation (CV) in estimated transmission rate of EV-A71 and CV-A16 by province. Calculated with $\alpha$ = 0.95 and province-specific maximum likelihood estimates of cross-protection from the two-serotype model. The 95% CIs for $\bar{S}$ are derived from the profile likelihood using the $\chi^{2}$ distribution with 1 degree of freedom; the 95% CIs for $\rho$ are derived from the standard errors for the coefficient $1/\rho$ in the OLS regression of cumulative births and cumulative cases; the maximum standard errors (se) of the $\log\left( \beta_{s} \right)$ terms across weekly $\hat{\beta}_{s}$ values are from the log-linear regression, representing variability around estimated $\beta_{s}$.

| Province | $\bar{\boldsymbol{S}}$ of  EV-A71  (CI) | $\bar{\boldsymbol{S}}$ of  CV-A16  (CI) | $\boldsymbol{\rho}$ of  EV-A71  (CI) | $\boldsymbol{\rho}$ of  CV-A16  (CI) | $\bar{\boldsymbol{\beta}}$ of  EV-A71  (se) | $\bar{\boldsymbol{\beta}}$ of  CV-A16 (se) | CV of $\boldsymbol{\beta}_{\mathbf{s}}$, EV-A71 | CV of $\boldsymbol{\beta}_{\mathbf{s}}$, CV-A16 |
| --- | --- | --- | --- | --- | --- | --- | --- | --- |
| Beijing | 0.065 (0.063, 0.068) | 0.050 (0.049, 0.051) | 0.065 (0.059, 0.071) | 0.058 (0.053, 0.064) | 22.80 (0.07) | 29.93 (0.06) | 0.22 | 0.24 |
| Tianjin | 0.074 (0.070, 0.079) | 0.047 (0.045, 0.048) | 0.052 (0.047, 0.058) | 0.043 (0.039, 0.047) | 19.63 (0.10) | 31.34 (0.09) | 0.27 | 0.26 |
| Hebei | 0.059 (0.058, 0.059) | 0.058 (0.058, 0.059) | 0.019 (0.018, 0.019) | 0.017 (0.016, 0.018) | 27.67 (0.02) | 28.16 (0.02) | 0.25 | 0.26 |
| Shanxi | 0.044 (0.042, 0.046) | 0.044 (0.044, 0.045) | 0.018 (0.018, 0.019) | 0.016 (0.016, 0.016) | 35.32 (0.04) | 35.84 (0.04) | 0.27 | 0.32 |
| Inner Mongolia | 0.548 (0.267, 1.000) | 0.060 (0.056, 0.064) | 0.020 (0.019, 0.020) | 0.016 (0.016, 0.017) | 2.81 (0.07) | 25.93 (0.07) | 0.31 | 0.33 |
| Liaoning | 0.045 (0.044, 0.046) | 0.051 (0.049, 0.052) | 0.029 (0.028, 0.031) | 0.035 (0.033, 0.037) | 34.02 (0.06) | 32.25 (0.23) | 0.34 | 0.67 |
| Jilin | 0.281 (0.214, 0.411) | 0.034 (0.033, 0.036) | 0.026 (0.025, 0.027) | 0.024 (0.024, 0.025) | 5.50 (0.07) | 53.71 (0.24) | 0.40 | 1.07 |
| Heilongjiang | 0.087 (0.081, 0.095) | 0.028 (0.028, 0.029) | 0.009 (0.009, 0.010) | 0.012 (0.011, 0.012) | 17.61 (0.05) | 59.93 (0.15) | 0.33 | 0.56 |
| Shanghai | 0.065 (0.062, 0.068) | 0.056 (0.054, 0.058) | 0.070 (0.067, 0.073) | 0.048 (0.046, 0.050) | 23.23 (0.03) | 27.06 (0.02) | 0.20 | 0.20 |
| Jiangsu | 0.056 (0.055, 0.057) | 0.053 (0.052, 0.054) | 0.043 (0.042, 0.044) | 0.028 (0.028, 0.029) | 28.91 (0.01) | 30.50 (0.01) | 0.19 | 0.19 |
| Zhejiang | 0.059 (0.058, 0.060) | 0.078 (0.076, 0.081) | 0.062 (0.061, 0.064) | 0.044 (0.042, 0.046) | 26.88 (0.01) | 20.37 (0.01) | 0.19 | 0.19 |
| Anhui | 0.055 (0.054, 0.055) | 0.071 (0.070, 0.072) | 0.035 (0.035, 0.036) | 0.024 (0.023, 0.025) | 29.43 (0.01) | 22.74 (0.01) | 0.17 | 0.17 |
| Fujian | 0.046 (0.046, 0.048) | 0.065 (0.064, 0.067) | 0.048 (0.047, 0.049) | 0.033 (0.032, 0.034) | 34.20 (0.02) | 24.22 (0.01) | 0.17 | 0.17 |
| Jiangxi | 0.080 (0.078, 0.083) | 0.095 (0.092, 0.097) | 0.020 (0.019, 0.020) | 0.014 (0.014, 0.015) | 19.98 (0.01) | 16.85 (0.01) | 0.19 | 0.19 |
| Shandong | 0.207 (0.196, 0.220) | 0.137 (0.131, 0.145) | 0.028 (0.027, 0.029) | 0.017 (0.016, 0.017) | 7.87 (0.02) | 11.91 (0.02) | 0.22 | 0.22 |
| Henan | 0.077 (0.075, 0.078) | 0.078 (0.078, 0.079) | 0.021 (0.021, 0.022) | 0.009 (0.009, 0.009) | 21.40 (0.01) | 21.32 (0.01) | 0.20 | 0.18 |
| Hubei | 0.087 (0.082, 0.094) | 0.058 (0.057, 0.059) | 0.041 (0.040, 0.041) | 0.019 (0.018, 0.019) | 18.45 (0.01) | 27.74 (0.01) | 0.23 | 0.21 |
| Hunan | 0.064 (0.063, 0.065) | 0.064 (0.063, 0.064) | 0.054 (0.052, 0.055) | 0.022 (0.022, 0.023) | 25.58 (0.02) | 25.60 (0.01) | 0.23 | 0.22 |
| Guangdong | 0.034 (0.033, 0.034) | 0.048 (0.048, 0.048) | 0.087 (0.083, 0.091) | 0.048 (0.046, 0.051) | 48.15 (0.01) | 34.26 (0.01) | 0.16 | 0.16 |
| Guangxi | 0.059 (0.058, 0.059) | 0.100 (0.098, 0.101) | 0.101 (0.097, 0.106) | 0.048 (0.046, 0.050) | 27.06 (0.01) | 16.14 (0.01) | 0.19 | 0.18 |
| Hainan | 0.131 (0.121, 0.145) | 0.068 (0.065, 0.072) | 0.094 (0.092, 0.097) | 0.061 (0.060, 0.063) | 11.32 (0.02) | 22.02 (0.02) | 0.16 | 0.19 |
| Chongqing | 0.018 (0.018, 0.018) | 0.054 (0.052, 0.056) | 0.017 (0.017, 0.017) | 0.015 (0.015, 0.015) | 86.53 (0.02) | 28.82 (0.02) | 0.20 | 0.22 |
| Sichuan | 0.042 (0.042, 0.042) | 0.036 (0.035, 0.036) | 0.012 (0.012, 0.013) | 0.012 (0.011, 0.012) | 38.82 (0.01) | 45.17 (0.01) | 0.18 | 0.18 |
| Guizhou | 0.063 (0.062, 0.064) | 0.063 (0.062, 0.063) | 0.019 (0.019, 0.020) | 0.018 (0.018, 0.019) | 24.99 (0.02) | 25.06 (0.01) | 0.21 | 0.20 |
| Yunnan | 0.040 (0.039, 0.040) | 0.059 (0.058, 0.061) | 0.020 (0.019, 0.020) | 0.017 (0.017, 0.018) | 39.89 (0.01) | 27.30 (0.01) | 0.15 | 0.16 |
| Tibet | 0.053 (0.052, 0.054) | 0.060 (0.058, 0.062) | 0.004 (0.004, 0.004) | 0.005 (0.004, 0.005) | 30.28 (0.18) | 27.84 (0.24) | 0.53 | 0.61 |
| Shaanxi | 0.029 (0.029, 0.030) | 0.038 (0.037, 0.038) | 0.037 (0.036, 0.037) | 0.030 (0.029, 0.031) | 54.27 (0.03) | 43.39 (0.05) | 0.26 | 0.50 |
| Gansu | 0.076 (0.071, 0.082) | 0.045 (0.044, 0.046) | 0.010 (0.010, 0.010) | 0.008 (0.008, 0.008) | 20.66 (0.05) | 37.96 (0.14) | 0.34 | 0.66 |
| Qinghai | 0.204 (0.189, 0.221) | 0.327 (0.295, 0.367) | 0.006 (0.005, 0.006) | 0.005 (0.005, 0.006) | 8.11 (0.19) | 5.89 (0.50) | 0.70 | 1.34 |
| Ningxia | 0.045 (0.041, 0.049) | 0.089 (0.080, 0.099) | 0.025 (0.025, 0.026) | 0.019 (0.018, 0.019) | 32.87 (0.11) | 18.04 (0.16) | 0.29 | 0.78 |
| Xinjiang | 0.111 (0.101, 0.123) | 0.048 (0.047, 0.049) | 0.006 (0.006, 0.006) | 0.004 (0.004, 0.005) | 14.11 (0.06) | 35.06 (0.14) | 0.32 | 0.68 |
