# Supplementary material for: Hand, Foot, and Mouth Disease in China: Modeling Epidemic Dynamics of Enterovirus Serotypes and Implications for Vaccination
Source: PLoS Med. 2016 Feb 16;13(2):e1001958. doi: 10.1371/journal.pmed.1001958 (PMC4755668; doi:10.1371/journal.pmed.1001958)
Supplement: S7 Table — Shown for both EV-A71 and CV-A16 from 2010 to 2013, for the one-, two-, and three-serotype models with α = 0.95 and province-specific maximum likelihood estimates of cross-protection from the two-serotype model. Values with asterisks indicate lower PE for the serotype in the two-serotype model compared to the one-serotype model, and values with carets indicate lower PE for the serotype in the three-serotype model compared to the two-serotype model. (DOCX) [file pmed.1001958.s044.docx]

**S7 Table. Within-province estimates of mean absolute yearly prediction error between observed and simulated weekly incidence.** Shown for both EV-A71 and CV-A16 from 2010 to 2013, for the one-, two-, and three-serotype models with $\alpha$ = 0.95 and province-specific maximum likelihood estimates of cross-protection from the two-serotype model. Values with asterisks indicate lower PE for the serotype in the two-serotype model compared to the one-serotype model, and values with carets indicate lower PE for the serotype in the three-serotype model compared to the two-serotype model.

| Province | 1-serotype  PE of EV-A71 | 2-serotype  PE of EV-A71 | 3-serotype  PE of EV-A71 | 1-serotype  PE of CV-A16 | 2-  serotype PE of  CV-A16 | 3-  serotype PE of  CV-A16 |
| --- | --- | --- | --- | --- | --- | --- |
| Beijing | 0.290 | 0.206* | 0.217 | 0.312 | 0.292* | 0.309 |
| Tianjin | 0.434 | 0.365* | 0.267^ | 0.741 | 0.666* | 0.303^ |
| Hebei | 0.236 | 0.138* | 0.105^ | 0.849 | 0.760* | 0.754^ |
| Shanxi | 0.353 | 0.353* | 0.353^ | 0.744 | 0.744* | 0.744^ |
| Inner Mongolia | 0.174 | 0.174* | 0.174^ | 0.280 | 0.281 | 0.282 |
| Liaoning | 0.282 | 0.368 | 0.328^ | 1.750 | 1.763 | 1.687^ |
| Jilin | 1.366 | 1.478 | 1.375^ | 1.334 | 1.322* | 1.079^ |
| Heilongjiang | 0.747 | 0.939 | 1.168 | 2.038 | 1.002* | 0.815^ |
| Shanghai | 0.435 | 0.365* | 0.374 | 0.429 | 0.397* | 0.369^ |
| Jiangsu | 0.264 | 0.262* | 0.291 | 0.371 | 0.347* | 0.344^ |
| Zhejiang | 0.198 | 0.223 | 0.223^ | 0.473 | 0.465* | 0.461^ |
| Anhui | 0.194 | 0.187* | 0.184^ | 0.451 | 0.422* | 0.419^ |
| Fujian | 0.186 | 0.194 | 0.198 | 0.393 | 0.417 | 0.421 |
| Jiangxi | 0.394 | 0.394* | 0.394^ | 0.556 | 0.556* | 0.556^ |
| Shandong | 0.437 | 0.402* | 0.406 | 0.431 | 0.414* | 0.387^ |
| Henan | 0.355 | 0.319* | 0.311^ | 0.121 | 0.119* | 0.193 |
| Hubei | 0.352 | 0.350* | 0.341^ | 0.195 | 0.196 | 0.193^ |
| Hunan | 0.580 | 0.495* | 0.499 | 0.226 | 0.234 | 0.217^ |
| Guangdong | 0.864 | 0.431* | 0.482 | 0.146 | 0.423 | 0.379^ |
| Guangxi | 0.927 | 0.188* | 0.290 | 0.142 | 0.235 | 0.154^ |
| Hainan | 0.882 | 0.882* | 0.882^ | 0.283 | 0.283* | 0.283^ |
| Chongqing | 0.138 | 0.151 | 0.137^ | 0.443 | 0.417* | 0.396^ |
| Sichuan | 0.107 | 0.119 | 0.094^ | 0.408 | 0.302* | 0.344 |
| Guizhou | 0.354 | 0.198* | 0.244 | 1.084 | 1.081* | 1.229 |
| Yunnan | 0.020 | 0.039 | 0.021^ | 0.651 | 0.584* | 0.599 |
| Tibet | 0.349 | 0.800 | 0.644^ | 1.908 | 1.475* | 1.866 |
| Shaanxi | 0.228 | 0.281 | 0.268^ | 0.507 | 0.503* | 0.492^ |
| Gansu | 1.149 | 1.149* | 1.149^ | 1.211 | 1.211* | 1.211^ |
| Qinghai | 1.419 | 1.398* | 1.387^ | 3.128 | 3.139 | 3.149 |
| Ningxia | 0.375 | 0.370* | 0.194^ | 0.788 | 0.743* | 0.596^ |
| Xinjiang | 0.460 | 0.479 | 0.828 | 1.301 | 1.271* | 0.915^ |
