# Supplementary material for: Hand, Foot, and Mouth Disease in China: Modeling Epidemic Dynamics of Enterovirus Serotypes and Implications for Vaccination
Source: PLoS Med. 2016 Feb 16;13(2):e1001958. doi: 10.1371/journal.pmed.1001958 (PMC4755668; doi:10.1371/journal.pmed.1001958)
Supplement: S8 Table — Shown for both EV-A71 and CV-A16 from 2010 to 2013, for the one-, two-, and three-serotype models with α = 0.95 and province-specific maximum likelihood estimates of cross-protection from the two-serotype model. (DOCX) [file pmed.1001958.s045.docx]

**S8 Table.** **Within-province estimates of the coefficient of determination (**$\boldsymbol{R}^{\boldsymbol{2}}$**) between observed and simulated weekly incidence.** Shown for both EV-A71 and CV-A16 from 2010 to 2013, for the one-, two-, and three-serotype models with $\alpha$ = 0.95 and province-specific maximum likelihood estimates of cross-protection from the two-serotype model.

| Province | 1-serotype  $\boldsymbol{R}^{\boldsymbol{2}}$ of EV-A71 | 2-serotype  $\boldsymbol{R}^{\boldsymbol{2}}$ of EV-A71 | 3-serotype  $\boldsymbol{R}^{\boldsymbol{2}}$ of EV-A71 | 1-serotype  $\boldsymbol{R}^{\boldsymbol{2}}$ of CV-A16 | 2-serotype  $\boldsymbol{R}^{\boldsymbol{2}}$ of CV-A16 | 3-serotype  $\boldsymbol{R}^{\boldsymbol{2}}$ of CV-A16 |
| --- | --- | --- | --- | --- | --- | --- |
| Beijing | 0.787 | 0.787 | 0.834 | 0.806 | 0.750 | 0.691 |
| Tianjin | 0.745 | 0.781 | 0.859 | 0.507 | 0.547 | 0.715 |
| Hebei | 0.709 | 0.785 | 0.816 | 0.146 | 0.224 | 0.284 |
| Shanxi | 0.682 | 0.682 | 0.682 | 0.258 | 0.258 | 0.258 |
| Inner Mongolia | 0.901 | 0.901 | 0.902 | 0.635 | 0.635 | 0.632 |
| Liaoning | 0.891 | 0.881 | 0.884 | 0.067 | 0.053 | 0.082 |
| Jilin | 0.759 | 0.721 | 0.759 | 0.502 | 0.549 | 0.663 |
| Heilongjiang | 0.617 | 0.522 | 0.401 | 0.021 | 0.010 | 0.000 |
| Shanghai | 0.586 | 0.644 | 0.662 | 0.501 | 0.452 | 0.576 |
| Jiangsu | 0.715 | 0.732 | 0.720 | 0.513 | 0.564 | 0.584 |
| Zhejiang | 0.803 | 0.769 | 0.767 | 0.517 | 0.516 | 0.549 |
| Anhui | 0.667 | 0.686 | 0.714 | 0.358 | 0.407 | 0.440 |
| Fujian | 0.785 | 0.782 | 0.775 | 0.518 | 0.473 | 0.504 |
| Jiangxi | 0.576 | 0.576 | 0.576 | 0.458 | 0.458 | 0.458 |
| Shandong | 0.831 | 0.860 | 0.862 | 0.767 | 0.774 | 0.798 |
| Henan | 0.832 | 0.854 | 0.914 | 0.567 | 0.564 | 0.500 |
| Hubei | 0.738 | 0.741 | 0.767 | 0.519 | 0.533 | 0.538 |
| Hunan | 0.549 | 0.676 | 0.678 | 0.286 | 0.327 | 0.39 |
| Guangdong | 0.505 | 0.865 | 0.812 | 0.313 | 0.542 | 0.506 |
| Guangxi | 0.612 | 0.918 | 0.893 | 0.459 | 0.544 | 0.628 |
| Hainan | 0.201 | 0.201 | 0.201 | 0.329 | 0.329 | 0.329 |
| Chongqing | 0.679 | 0.671 | 0.604 | 0.536 | 0.586 | 0.649 |
| Sichuan | 0.797 | 0.666 | 0.651 | 0.735 | 0.870 | 0.853 |
| Guizhou | 0.439 | 0.583 | 0.576 | 0.132 | 0.119 | 0.061 |
| Yunnan | 0.848 | 0.804 | 0.827 | 0.476 | 0.582 | 0.585 |
| Tibet | 0.207 | 0.126 | 0.211 | 0.579 | 0.483 | 0.303 |
| Shaanxi | 0.287 | 0.295 | 0.314 | 0.157 | 0.178 | 0.193 |
| Gansu | 0.142 | 0.142 | 0.142 | 0.081 | 0.081 | 0.081 |
| Qinghai | 0.081 | 0.082 | 0.081 | 0.012 | 0.012 | 0.012 |
| Ningxia | 0.734 | 0.745 | 0.615 | 0.249 | 0.290 | 0.476 |
| Xinjiang | 0.696 | 0.710 | 0.352 | 0.059 | 0.054 | 0.051 |
